# Ir(III) Half-Sandwich Photosensitizers with a π-Expansive Ligand for Efficient Anticancer Photodynamic Therapy

**DOI:** 10.1021/acs.jmedchem.3c01276

**Published:** 2024-01-31

**Authors:** Carlos Gonzalo-Navarro, Elisenda Zafon, Juan Angel Organero, Félix A. Jalón, Joao Carlos Lima, Gustavo Espino, Ana María Rodríguez, Lucía Santos, Artur J. Moro, Sílvia Barrabés, Jessica Castro, Javier Camacho-Aguayo, Anna Massaguer, Blanca R. Manzano, Gema Durá

**Affiliations:** † Departamento de Química Inorgánica, Orgánica y Bioquímica- IRICA, Facultad de Ciencias y Tecnologías Químicas, Universidad de Castilla-La Mancha, Avda. C. J. Cela, 10, 13071 Ciudad Real, Spain; ‡ Departament de Biologia, Facultat de Ciències, Universitat de Girona, Maria Aurèlia Capmany 40, 17003 Girona, Spain; § Departamento de Química Física, Facultad de Ciencias Ambientales y Bioquímicas and INAMOL, Universidad de Castilla-La Mancha, 45071 Toledo, Spain; ∥ LAQV-REQUIMTE, Departamento de Química, Faculdade de Ciências e Tecnologia, Universidade NOVA de Lisboa, 2829-516 Caparica, Portugal; ⊥ Departamento de Química, Facultad de Ciencias, Universidad de Burgos, Pza. Misael Bañuelos, s/n, 09001 Burgos, Spain; # Departamento de Química Inorgánica, Orgánica y Bioquímica- IRICA, Escuela Técnica Superior de Ingenieros Industriales, Universidad de Castilla-La Mancha, Avda. C. J. Cela, 3, 13071 Ciudad Real, Spain; ∇ Departamento de Química Física, Facultad de Ciencias y Tecnologías Químicas, Universidad de Castilla-La Mancha, Avda. C. J. Cela, s/n, 13071 Ciudad Real, Spain; ○ Analytical Chemistry Department, Analytic Biosensors Group, Instituto de Nanociencia y Nanomateriales de Aragon, Faculty of Sciences, University of Zaragoza, 50009 Zaragoza, Spain

## Abstract

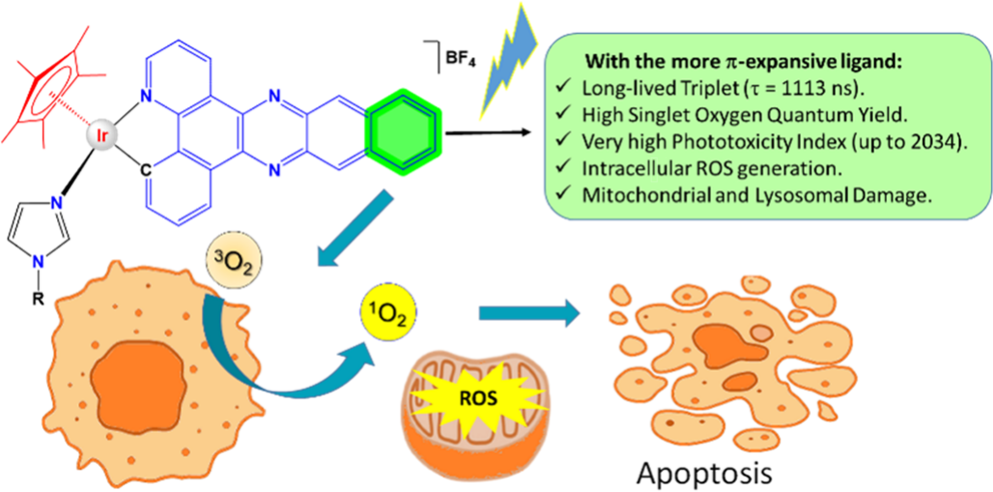

One approach to reduce the side effects of chemotherapy in cancer treatment is photodynamic therapy (PDT), which allows spatiotemporal control of the cytotoxicity. We have used the strategy of coordinating π-expansive ligands to increase the excited state lifetimes of Ir(III) half-sandwich complexes in order to facilitate the generation of ^1^O_2_. We have obtained derivatives of formulas [Cp*Ir(C^∧^N)Cl] and [Cp*Ir(C^∧^N)L]BF_4_ with different degrees of π-expansion in the C^∧^N ligands. Complexes with the more π-expansive ligand are very effective photosensitizers with phototoxic indexes PI > 2000. Furthermore, PI values of 63 were achieved with red light. Time-dependent density functional theory (TD-DFT) calculations nicely explain the effect of the π-expansion. The complexes produce reactive oxygen species (ROS) at the cellular level, causing mitochondrial membrane depolarization, cleavage of DNA, nicotinamide adenine dinucleotide (NADH) oxidation, as well as lysosomal damage. Consequently, cell death by apoptosis and secondary necrosis is activated. Thus, we describe the first class of half-sandwich iridium cyclometalated complexes active in PDT.

## Introduction

Cancer has a great incidence in modern societies, and one of the most successful strategies for the treatment of this disease is chemotherapy. In this field, platinum derivatives^1^ are commonly used although they have some important limitations, such as resistance and side effects. As a consequence, researchers are searching for alternatives, and these include other platinum derivatives^2^ with improved properties or complexes of other metals such as Ru, Ir, Au, Rh, Os, and Re.^3−7^

One approach to reduce the side effects of chemotherapy is photodynamic therapy (PDT), which is a noninvasive treatment that allows spatiotemporal control of cytotoxic activity.^8−11^ This treatment involves the synergistic action of three components: a photosensitizer (PS), light and cellular O_2_. The PS is a chromophore that ideally lacks biological activity in its ground state (dark conditions) and can be administered to the patient intravenously. The area to be treated is irradiated with light of a precise wavelength, and this promotes the PS to its excited state, PS*. The excited photosensitizer, PS*, will react with cellular ^3^O_2_ either through an electron transfer process (type I mechanism) that will generate O_2_^•–^ and other reactive oxygen species (ROS) or through an energy transfer process (type II mechanism) that produces singlet oxygen, ^1^O_2_, a very reactive and toxic species. The lifetime of ^1^O_2_ in a biological environment is very short, and this implies that the damage will be reduced to regions in very close proximity to the irradiated zone.^12^ A very important requirement for the clinical application of a PS is strong photocytotoxicity, which is measured by the phototoxic index (PI), a parameter defined as the ratio of the toxicity in the dark and after light irradiation (PI = IC_50,dark_/IC_50,light_). Very efficient ^1^O_2_ generation is also a crucial requirement for a PDT active compound. FDA-approved PSs mainly contain a tetrapyrrolic core,^13^ although their clinical use is constrained by the short lifetime of their excited state, their low solubility in aqueous solutions, and their significant side effects.^13^ Different transition metal complexes are emerging species of high interest for PDT applications.^14−16^ Octahedral polypyridine or related ruthenium(II) complexes have been extensively studied,^17,18^ even with π-extended ligands,^19^ and it is worth highlighting that the Ru(II) polypyridyl complex TLD-1433, prepared by McFarland et al., is under clinical trials for nonmuscle invasive bladder cancer.^20^ Octahedral tris(chelate) bis-cyclometaled iridium(III) complexes are also attracting interest in this field and also as diagnostic agents due to their exceptional photophysical properties,^21−26^ excellent photostability, and cell permeability.^4,21,27−30^

Although there are numerous cases of cyclopentadienyl iridium derivatives,^31^ to our knowledge, only three examples of this kind of derivatives with PDT activity have been reported so far and all of them contain very sophisticated and hard-to-synthesize ligands that could even be the origin of the PDT activity.^32−34^ With the exception of one complex with PI values >278 or >417,^34^ in the rest of the cases, the PI values are rather low.^32,33^ Reports on half-sandwich cyclometalated iridium complexes with PDT activity have not been published. However, this kind of compounds has been described as being quite active chemotherapeutic agents.^31,35,36^ Sadler et al. reported several interesting examples in this field using 2-phenylpyridinate (ppy).^37−41^ Modifications of the ppy ligand,^38,42−45^ the cyclopentadienyl ring,^45^ or the use of other C^∧^N ligands^46−50^ have been described.

The extremely scarce use of half-sandwich iridium complexes in PDT is surely due to their poor photophysical properties. Derivatives of the type [Cp*Ir(C^∧^N)L]^+^ or [Cp*Ir(N^∧^N)L]^2+^ are usually nonemissive at room temperature and their lifetimes are short.^51,52^ It has been demonstrated that compounds with long excited state lifetimes are more efficient ROS producers, have large ^1^O_2_ quantum yields, and show high in vitro photocytotoxicities.^53−55^ It has been proposed^51^ that one reason for the short lifetimes of half-sandwich Ir compounds is their relatively weak ligand field, even with C^∧^N ligands, and this situates the nonradiative d–d state (MC) at an energy close to that of the emissive state, thus reducing the lifetime of the latter. One approach to solve this problem is to increase the energy gap between the MC and ^3^MLCT states.^52^ However, in the present work, it was decided to evaluate a different strategy in the search for half-sandwich iridium cyclometalated complexes with long-lived triplet excited states that could be active in PDT.

Our rationale was based on the following points: (i) The more the ^3^ππ* character of the triplet excited state, the longer the T_1_ lifetime;^56^ (ii) the use of ligands with extended π-systems lowers the energy of the ligand-centered (LC) ^3^ππ* state to below, or close to, that of the ^3^MLCT state notably increasing the ^3^ππ* character of the T_1_ state.^27,53,57,58^ Indeed, it has been reported that extending the π-conjugation of some ligands (N-donor or cyclometalated C^∧^N) on tris(chelate) or bis(tridentate) Ru(II)^53,54,59−64^ or Ir(III)^27,56,57,65,66^ complexes has led to increased ππ* character of the lowest-lying triplet excited states. In particular, iridium derivatives of the type [IrCl(C^∧^N)(terpy)]^+^ or ruthenium complexes of stoichiometry [Ru(bpy)_2_(C^∧^N)]^+^ with the C^∧^N ligands used in this work (pbpz, 4,9,14-triazadibenzo[*a*,*c*]anthracene or pbpn, 4,9,16-triazadibenzo[*a*,*c*]naphthacene) have been reported^19,67^ exhibiting moderate or good PDT efficacy. However, the strategy of using ligands with π-extension has never been applied to half-sandwich iridium complexes.

**Chart 1:**
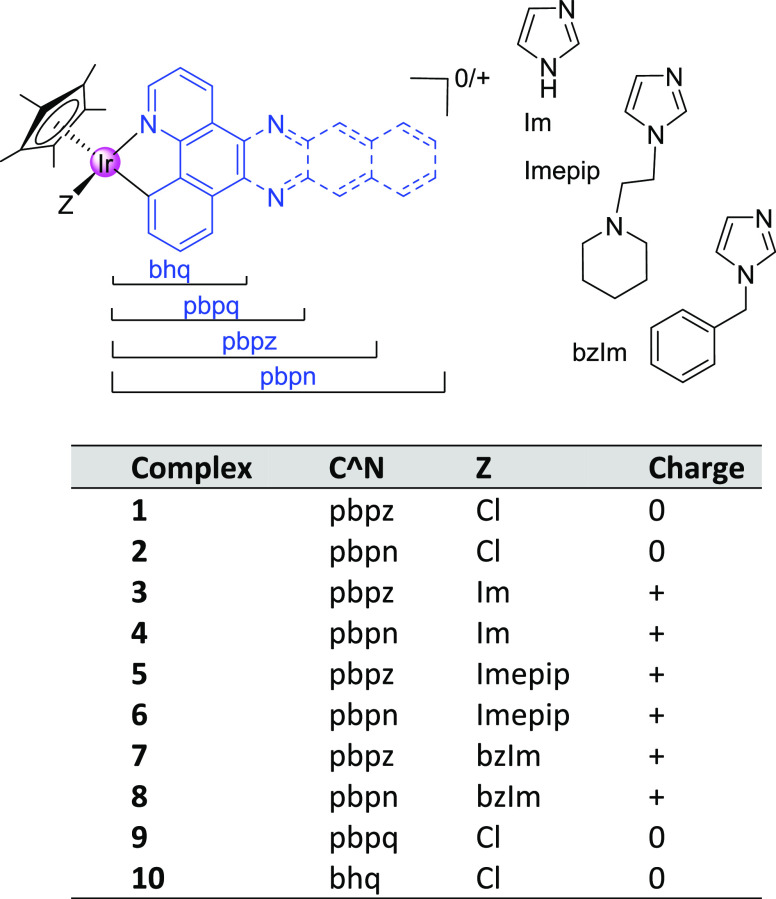
Iridium(III) Complexes Synthesized in This Worka

We present here the synthesis, characterization, and biological activity of a set of 10 pentamethylcyclopentadienyl iridium complexes with C^∧^N ligands with a gradual expansion in their π-conjugation (see Chart 1). In the case of the highest π-extension (pbpn ligand), the formation of an excited triplet state with lifetimes in the microsecond range is observed. This leads to remarkable ^1^O_2_ generation and outstanding phototoxicity indexes higher than 2000 against cancer cells. The results of time-dependent density functional theory (TD-DFT) studies nicely explain the special characteristics of the complexes containing this ligand due to a high ^3^ππ* character of the T_1_ state. Notably, the present [Cp*IrL(pbpn)]^+^ complexes are the first class of half-sandwich cyclometalated Ir derivatives with PDT behavior.

a Cationic complexes **3**–**8** were isolated as tetrafluoroborate salts.

## Results and Discussion

### Synthesis and Characterization

A new family of eight Ir(III) complexes of general formulas [Cp*Ir(C^∧^N)Cl] and [Cp*Ir(C^∧^N)L]BF_4_ (**series A**: C^∧^N = pbpz, **series B**: pbpn; in both series: L = imidazole, Im; (*N*-ethylpiperidyl)imidazole, Imepip; *N*-benzyl-imidazole, bzIm) has been prepared (Chart 1). For the sake of comparison of the effect of the π-extended conjugation in the C^∧^N ligand on the biological properties, the chlorido complexes of formula [Cp*Ir(C^∧^N)Cl], with shorter C^∧^N ligands (C^∧^N = pbpq, benzo[*f*]pyrido[2,3-*h*]quinoxaline, complex **9**, and bhq, benzo[*h*]quinoline, complex **10**), have been prepared as well. Complex **10** was previously reported.^68^ The proligands Hpbpz, Hpbpn, and Hpbpq were synthesized using a literature procedure (Scheme S1).^19^ Complexes **1**, **2**, **9**, and **10** were synthesized from the dimeric starting material [Cp*IrCl_2_]_2_ by a nonreported procedure through the addition of AgOCOCF_3_ and Na_2_CO_3_ (Scheme S1). Subsequently, the cationic complexes (**3**–**8**) were obtained from **1** and **2** using one equivalent of silver tetrafluoroborate, in the presence of the N-donor monodentate ligands. The complexes were obtained in moderate to very good yields (63–98%). The 10 complexes were fully characterized by elemental analysis, ^1^H and ^13^C{^1^H} NMR spectroscopy (Tables S1 and S2, Figures S1–S27) including ^1^H–^1^H COSY, ^1^H–^13^C gHSQC and ^1^H–^13^C gHMBC experiments and mass spectrometry (Figures S28–S37). The high-performance liquid chromatography (HPLC) traces were also obtained (Figures S38–S46). **1**, **6**, and **9** were also characterized by a single-crystal X-ray diffraction study.

The complexes exhibited good solubility in polar solvents such as dimethyl sulfoxide (DMSO) and chlorinated solvents, as well as in methanol or tetrahydrofuran (THF). The chloride complexes were poorly soluble in aqueous media. However, the cationic complexes were soluble in water provided that a small amount of another solvent, such as DMSO, CH_3_OH, or CH_3_CN was added. Thus, nontoxic amounts of DMSO were used in the biological experiments to assist dissolution. The complexes were fully soluble at the maximum concentration used during the biological assays.

The p*K*_a_ values of complexes **5** and **6**, along with that of the free Imepip ligand, were determined in order to find out if the Imepip ligand is protonated under the conditions of the biological studies (Figures S47–S49). The values were determined by recording the absorbance spectra at different pH values in DMSO/H_2_O mixtures (2/98, v/v) with Bu_4_NBF_4_ at 5 mM. Both **5** and **6** exhibited a single equilibrium with p*K*_a_ = 7.2 and 7.8, respectively (Table S2). The p*K*_a_ of the Imepip ligand is 7.1. It is proposed that all of these p*K*_a_ values correspond to the protonation–deprotonation equilibrium of the piperidine ring. Thus, the Imepip ligand in complexes **5** and **6** is partially protonated under the conditions of the biological studies (pH: 7.4).

### X-ray Crystal Structures

**Figure 1:**
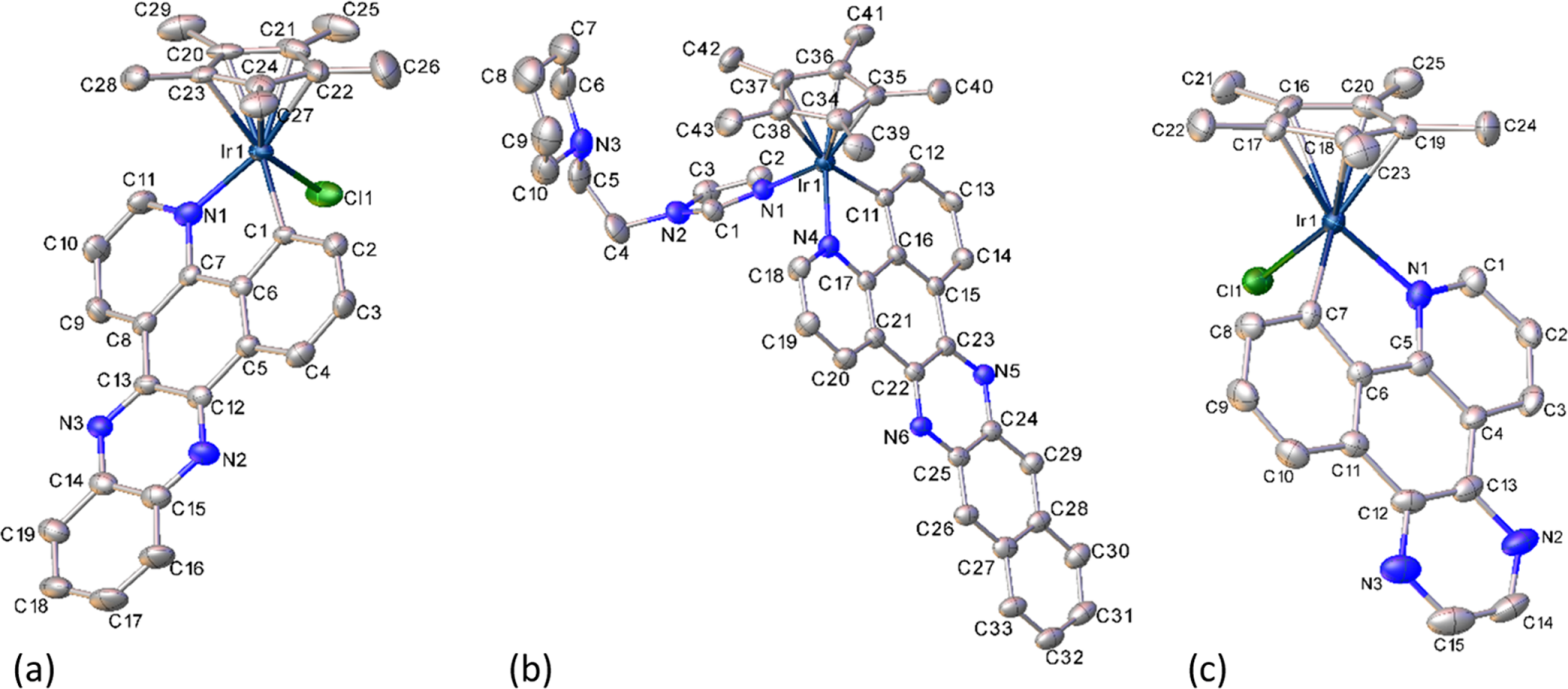
ORTEP diagrams of complex S_Ir_**1** (a), cation of S_Ir_**6** (b), and complex R_Ir_**9** (c). Ellipsoids are at the 30% probability level. Hydrogen atoms and BF_4_^–^ anions have been omitted for clarity.

The molecular and crystal structures of complexes **1**, **6 × 0**.**75C**_**3**_**H**_**6**_**O**, and **9 × 0**.**5C**_**4**_**H**_**10**_**O** were determined by X-ray diffraction. Selected bond distances and angles are gathered in Table S4, the crystallographic data are provided in Table S3, and the corresponding ORTEP diagrams are shown in Figure 1. The unit cells of the complexes contain both enantiomers, R_Ir_ and S_Ir_, arising from the stereogenic nature of the metal center.

The three metallic derivatives exhibit the expected pseudo-octahedral half-sandwich geometry with the Cp* ring adopting an η^5^-coordination mode and occupying three coordination sites, while the C^∧^N ligand exhibits a bidentate-chelate coordination mode (κ^2^-C,N). A chloride anion (**1** and **9**) or an Imepip ligand (**6**) completes the coordination sphere around the metal center. The distances involving the Ir atom (Table S4) are as one would expect.^38,42^ The *N*-ethylpiperidine (in **6**) fragment is oriented in the opposite direction to the C^∧^N ligand (see Figure 1).

### Stability and Photostability of **1**–**8**

The possible hydrolysis process of the chlorido complexes **1** and **2** was studied by ^1^H NMR spectroscopy. Sadler observed this process in different half-sandwich cyclometalated chlorido iridium compounds.^38,42,69^ The evolution of **1** and **2** in the dark was studied in a DMSO-*d*_6_/D_2_O (9:1) solvent mixture (Figures S50–S52 and Table S5) and in DMSO-*d*_6_ (Figures S53–S56 and Tables S6–S7). Thus, the formation of the aqua adducts [Cp*Ir(C^∧^N)(D_2_O)]^+^ (**1w** and **2w**) was deduced in both cases although at a slower rate in the case of **2**. The reversibility of the process was confirmed by addition of an excess of Bu_4_NCl to a solution of **1w** (see Figures S57–S58).

We also studied, by means of UV–vis, the stability of complexes **1**–**8**, in the presence of serum (Dulbecco’s modified Eagle’s medium (DMEM) without phenol red, Corning), to simulate biological experiments and evaluate the effect of the different species present in this medium, including nucleophiles. In the dark, no changes were observed (48 h, Figure S59), (a minor change in the case of **1** was observed after 24 or 48 h) while in the presence of blue light (470 nm), some modifications were observed both in complexes of **series A** and **B** (Figure S60). More specifically, a decrease of the visible band was observed. In the case of **6**, we decided to perform more experiments. On the one hand, we registered the UV–vis spectra in water under blue light irradiation, and a similar effect was observed (Figure S61). This kind of transformation is typical of the formation of aggregates.^70^ We also performed a ^1^H NMR experiment (although in a higher concentration range and lower proportion of water) in order to evaluate the possible dissociation of the imidazolyl ligands in the cationic complexes and the release of the N-donor ligand was not observed in any case (in the dark or under blue, green or red light irradiation) (Figures S62–S65). Considering these facts, we propose that the observed effect is due to an aggregation process favored by the hydrophobicity of the pbpn or pbpz ligands and the polarity of water, a process that takes place, at least for the concentration studied, only in the presence of light. The effect of the light should imply that the formation of aggregates takes place in the excited state. In fact, considering theoretical calculations (see below), aggregates formation is not very favorable for the complexes in their ground states since charge distribution of their highest occupied molecular orbital (HOMO) is located on Ir+Cp* fragments (Table S11), but upon photoexcitation, a significant metal to ligand charge transfer process causes the population of their lowest unoccupied molecular orbitals (LUMO) centered on the quinoxaline or benzo-quinoxaline fragments, for complexes of **series A** and **B**, respectively.

### Singlet Oxygen Production

**Figure 2:**
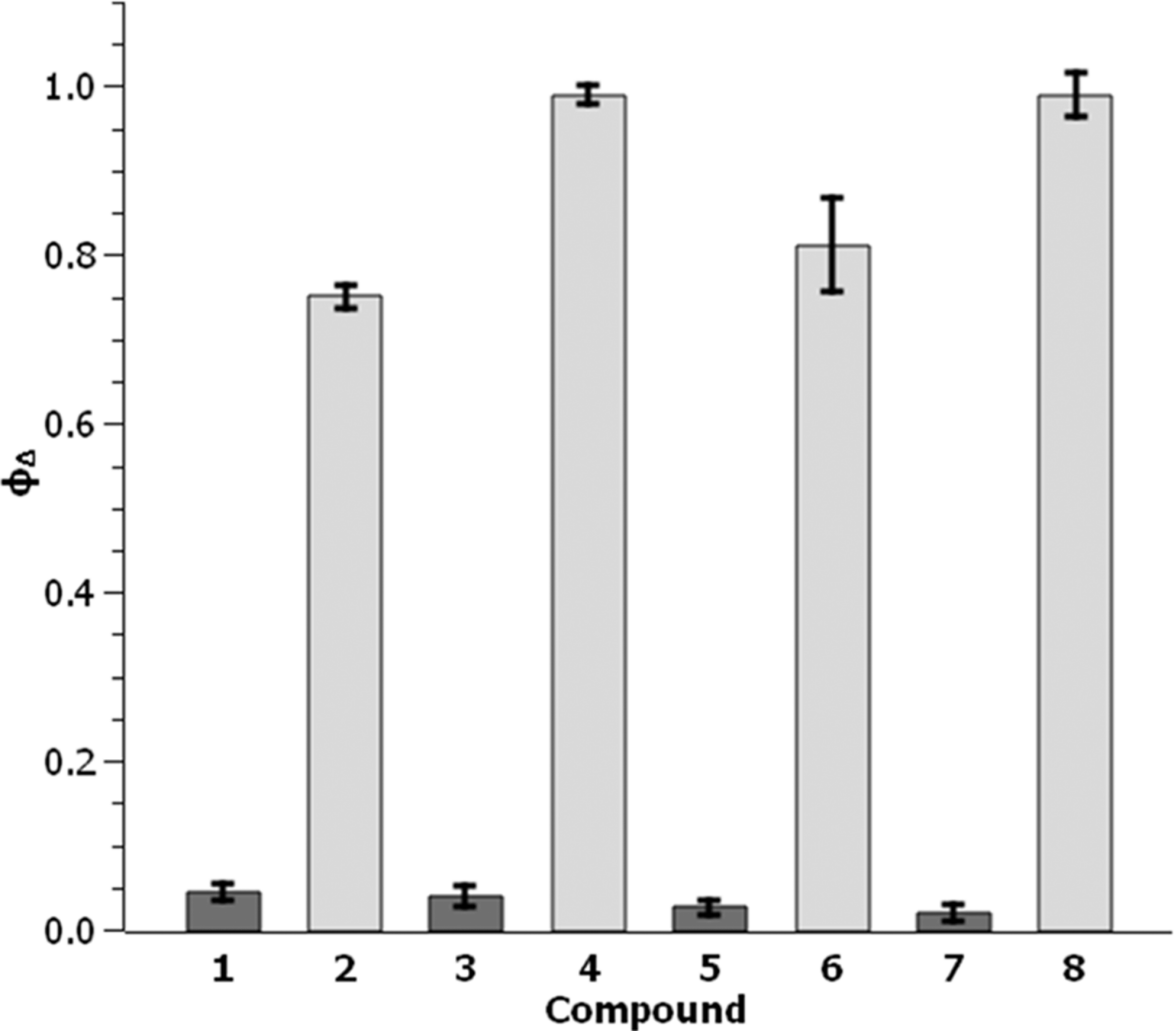
^1^O_2_ generation quantum yields of complexes of **series A** (dark gray) or **series B** (light gray) in CH_3_CN. Ir(III) complexes were excited at 470 nm by a light-emitting diode (LED) (2.3 W).

Singlet oxygen is considered the main cytotoxic species in PDT processes. Therefore, the ability of the PSs reported here to generate singlet oxygen (^1^O_2_) was evaluated by monitoring the oxidation of 1,3-diphenylisobenzofuran (DPBF, yellow, 5 × 10^–5^ M) to 1,2-dibenzoylbenzene (colorless) in acetonitrile in the presence of **1**–**8** (1 × 10^–6^ M) under blue light (470 nm) irradiation by UV–vis spectroscopy. The intensity of the DPBF bands (e.g., λ_max_ = 410 nm) decreased over time in all cases (Figures S66–S69 and Table S8). Control experiments in the absence of either O_2_, light, or PSs were performed in an effort to elucidate the nature of this transformation and the role of the new PSs. Thus, the oxidation of DPBF was not observed in the absence of either O_2_ or light. However, in the absence of PSs, partial oxidation of DPBF (25%) was observed after 20 s in aerated acetonitrile under blue light irradiation (Figure S70). Therefore, the corresponding correction was applied to the calculations. These results indicate that (a) DPBF tends to decompose under blue light irradiation in the presence of O_2_ and (b) complexes **1**–**8** accelerate the photooxidation of DPBF through a photocatalytic process. Furthermore, the formation of ^1^O_2_ was confirmed in an experiment performed in the presence of **6** and NaN_3_ (Figure S71). As one would expect, this singlet oxygen quencher inhibited the oxidation of DPBF. More specifically, complexes of **series B** (bearing pbpn, i.e., **2**, **4**, **6**, and **8**) showed a higher photocatalytic activity (Figure S72) than their analogues of **series A** (bearing pbpz, i.e., **1**, **3**, **5**, and **7**). Indeed, near complete photodegradation of DPBF was observed after only 25 s for **4**, **6**, and **8**. The ^1^O_2_ generation quantum yields (ϕ_Δ_) were obtained and are provided in Table S10 (see also Figure 2). Very high ϕ_Δ_ values were obtained for complexes of **series B**, thus confirming the enormous potential of these compounds as singlet oxygen photosensitizers. In particular, for complex **8**, ϕ_Δ_ = 0.99. By contrast, the four derivatives with pbpz exhibited low ϕ_Δ_ values and this highlights the significant influence of the extra ring in the C^∧^N ligand on the photophysical properties. Similar experiments were carried out on complexes of **series B** under green light irradiation. In this case, the consumption of DPBF was much slower relative to that observed with blue light (Figures S73–S74).

Although DPBF is the probe normally used to detect ^1^O_2_ and has very high sensitivity, it has the drawback that it is not specific to ^1^O_2_, as may react with other ROS as superoxide anion and H_2_O_2_.^71,72^ In order to unambiguously conclude about the formation of ^1^O_2_, another probe, ABDA, 9,10-anthracenediyl-bis(methylene)dimalonic acid, was used. ABDA has a much lower reactivity and a much smaller spectroscopic response, but it is specific for ^1^O_2_, allowing for discrimination from other ROS.^73^ It is quite water-soluble and thus, the formation of ^1^O_2_ was determined in H_2_O/DMSO (95:5) mixtures. This determination was carried out with two pairs of complexes, the neutral **1** and **2** and the cationic **7** and **8** (see Figures S75–S78 and Table S9). The formation of ^1^O_2_ was verified in all cases and, as it was found with DPBF, higher yields were obtained with complexes containing the pbpn ligand. As expected, the values were lower than those obtained with DPBF in acetonitrile.^73^ Besides, the high concentration of the complexes that was necessary to be used (10^–3^ M) probably leads to aggregation phenomena that may influence the specific values. The high values in acetonitrile reflect that the complexes could produce a high amount of ^1^O_2_ when located in lipophilic cell regions.

It was subsequently decided to study the excited states of the two series of PSs by different techniques in conjunction with theoretical calculations in an effort to understand their nature and the photophysical mechanism involved in the production of singlet oxygen.

### Photophysical Properties

#### UV–Vis Absorption Spectra

The UV–vis spectra of complexes **1**–**10** were recorded in acetonitrile solutions (10^–5^ M) at 25 °C (Figure S79). The spectra, grouped by families, are shown in Figure S80 (the four chlorido complexes are also compared) and the absorption spectra of the free ligands are shown in Figure S81. All of the spectra contain two very intense, high-energy bands below 340–350 nm with a red shift for the complexes of **series B** (C^∧^N = pbpn). Moreover, the spectra exhibit a third band of lower intensity centered at around 380 nm (**9** and **10**), 400 nm (**series A**, C^∧^N = pbpz), and 420 nm (**series B**) with featureless and weak absorption tails that extend well into the visible region for families of **series A** and **B** (above λ = 450 or 500 nm for complexes of **series A** and **B**, respectively) (see Table S10). The simulated TD-DFT absorption spectra for compounds **5**–**8** and their calculated electronic transitions (Figures S90–S91 and Tables S12–S15, see below for the theoretical calculations) suggest that the highest-intensity bands mainly correspond to a mixture of metal to C^∧^N ligand (d → π*, ^1^MLCT) charge transfer and C^∧^N ligand to ligand (π → π*, ^1^LC) transitions, while the lowest-intensity bands mainly correspond to ^1^MLCT transitions in both series. The red shift observed in the absorption spectra of complexes of **series B** reflects the strong electron-withdrawing ability and extended π-conjugation of the benzo-quinoxaline ligand enabling larger oscillator strength values. Moreover, an additional C^∧^N ligand-centered transition (π → π*) was exclusively observed in the lower-intensity bands of these compounds, which contribute to their broadening. The spectra recorded for members of each family (**A** or **B**) are very similar among them. However, the stated red-shifted absorption exhibited by PSs of **series B**, relative to their congeners of **series A** and also relative to **9** and **10**, improves their light-harvesting ability and makes them sensitive to light sources with deeper tissue penetration capacity.

#### Emission Spectra

The photoluminescence spectra of **1**–**8** were recorded in solutions of dry deoxygenated acetonitrile (10^–5^ M) at 25 °C (Figure S82 and Table S10). Complexes of **series A** are not emissive at this concentration while the spectra recorded for derivatives of **series B** show a relatively weak emission at about 550 nm upon excitation at λ = 420 nm.

Considering the low emissive character of these complexes and the high ability to generate ^1^O_2_ of complexes of **series B** we decided to evaluate the presence of long-lived states that could be quenched by O_2_. Thus, the lifetime of possible triplet states was measured by Transient Absorption Spectroscopy (TAS) in complexes of series **A** and **B**.

#### Transient Absorption Spectroscopy

TAS measurements were performed with two pairs of complexes **5**/**6** and **7**/**8**. According to our expectations, TAS of **6** and **8** show the presence of an excited state with very long lifetimes (τ = 2430 ns for **6**; τ = 976 ns for **8**) in the absence of O_2_. The observed values for the lifetimes decreased markedly in the presence of O_2_ (τ = 176 ns for **6**; τ = 174 ns for **8**) (Table 1, Figures S83 and S84). This led to the conclusion that these excited states must have a triplet nature (T_1_) and are responsible for the generation of ^1^O_2_. We found no evidence for similar long-lived states in **5** and **7**. The transients of **5** and **7** show decay times in the range 12–20 ns and they do not show quenching by oxygen (Table 1, Figures S85 and S86).

**Table 1:** Summary of Recorded Lifetimes of the Iridium Complexes in Acetonitrile (λ_exc_ = 355 nm) from TAS Measurements^a^

	lifetimes at 270/300 nm (μs)		lifetimes at 540 nm (μs)	
comp.	with O_2_	without O_2_	with O_2_	without O_2_
**5**	0.015	0.012	0.020	0.020
**7**	0.015	0.015	0.022	0.020
**6**	0.176	2.430	0.187	2.520
**8**	0.174	0.976	0.185	1.250

a Lifetimes were recorded at the wavelengths corresponding to the bleaching of the ground state (270 nm for **5** and **7**, 300 nm for **6** and **8**) and at 540 nm (absorption of the transient state). *A* ∼ 0.2.

It is important to stress that the observed transients of **6** and **8** in the presence of oxygen display total recovery of the bleaching within the time scale of the measurements, which allows the assignment of the quenching by oxygen to energy transfer, compatible with singlet oxygen formation. The absorption spectra recorded before and after flash photolysis experiments for all complexes were the same, thus indicating that photodegradation did not occur (Figure S87).

### Theoretical Calculations

In order to obtain a better understanding of the important differences in the photophysical properties of complexes of **series A** and **B** and give further support to the outstanding ability of complexes of **series B** for the generation of ^1^O_2_ generation, TD-DFT calculations were performed for singlet and triplet transitions of complexes **5**–**8**. Compounds **5** and **6**, on the one hand, and **7** and **8** on the other hand share the same monodentate ligand L but differ in the C^∧^N cyclometalating fragment.

**Figure 3:**
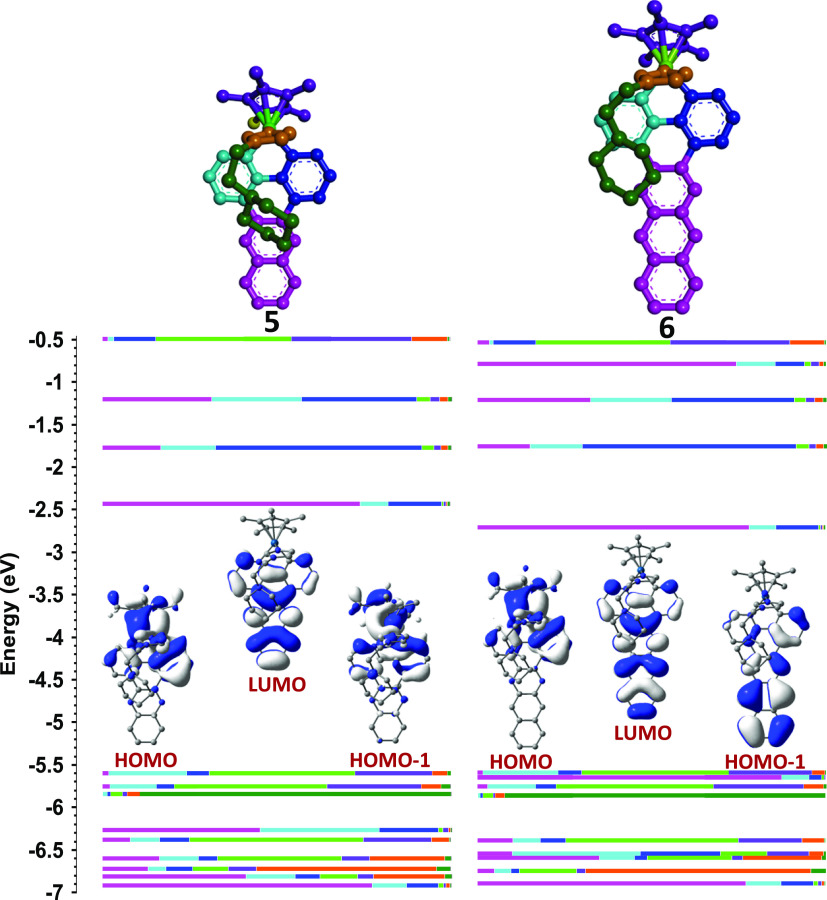
Optimized ground state structures for **5** and **6** obtained from TD-DFT [(B3LYP/SDD for Ir(III)) and (6-31 g** for C,H,N)] with SMD (CH_3_CN). For the sake of clarity, the hydrogen atoms are not shown and each molecular fragment has been assigned a color code. Calculated fragmental contributions and energies for some selected molecular orbitals are provided along with the topologies of HOMO–1, HOMO, and LUMO for **5** and **6**. The length of each color bar is proportional to the percentage contribution of the corresponding colored moiety to each molecular orbital. Color codes for bars and molecular fragments: Cp* (purple), iridium (light green), quinoxaline or benzo-quinoxaline (pink), benzene (blue), pyridine (navy blue), imidazole (brown), ethylpiperidine (dark green).

The minimum energy conformations found for **5** and **6** were those in which the ethylpiperidine fragment exhibited CH−π interactions^74^ with the quinoxaline and benzo-quinoxaline units, respectively (Figure 3). A similar behavior was observed for **7** and **8**, in which the phenyl ring of the benzyl group exhibited π–π stacking interactions with the quinoxaline and benzo-quinoxaline fragments, respectively (Figure S88). The geometry optimizations of **6** and **7** present a parallel orientation of the piperidine and benzyl ligands regarding the C^∧^N ligand, whereas the X-ray structure of **6** exhibits an antiparallel orientation. Therefore, the experimental structures do not correspond to the minima of potential energy surfaces of the single molecules in solution, because free from the crystal packing forces, they are probably on a slope of their potential energy surface and not in their minima. Therefore, the geometries would be mainly affected by the rotation of bonds and ligands with low rotational barriers as Cp*^75^ toward lower potential surfaces by DFT optimizations, revealing the energetic stress involved in the crystal structure. A comprehensive manner to compare the X-ray and the theoretical geometries is to superimpose both structures and calculate the root-mean-square deviation (RMSD) of atomic positions. Thus, Figure S89 shows that the RMSD value corresponding to the overlapping of the C^∧^N moiety for **6** is 0.025. This reflects in almost identical experimental and theoretical geometries for the fragment, which is not affected by the crystal packing effect.

The respective fragmental contributions of molecular orbitals corresponding to complexes **5** and **6** are shown in Figure 3 and those for complexes **7** and **8** are provided in Figure S88. In the cases of **6** and **8** the HOMO and HOMO–1 orbitals are almost isoenergetic since they have negligible energy differences of 0.05 and 0.08 eV, respectively. The major contributions to the HOMO in **6** and **8** come from the atomic orbitals of the iridium atoms and the molecular orbitals of the Cp* ligands, with relative values Ir+Cp* of 42 + 24% and 43 + 27%, respectively (Table S11). In contrast, the largest contribution to the HOMO–1 orbitals of **6** and **8** comes from the benzo-quinoxaline moieties (87%) and these orbitals have a π nature. Furthermore, the fragmental contributions of the HOMO and HOMO–1 orbitals in **5** and **7** are very similar to those of the HOMO of **6** and **8**, but the energy differences between these orbitals, 0.15 and 0.17 eV, respectively, are significantly larger than those calculated for **6** or **8**. It is worth highlighting that the energy values and the fragmental contributions calculated for HOMO and HOMO–2 of **6** and **8** are very similar to those calculated for HOMO and HOMO–1 of **5** and **7** (see Figures 3 and S88). The topologies involve d orbitals of the iridium atom in combination with π-electron clouds of the Cp* moiety. Regarding the LUMO, these are mainly located on the benzo-quinoxaline fragments in **6** and **8** and on the quinoxaline fragments in **5** and **7**, with equivalent energy values of −2.71 and −2.43 eV for each pair, respectively. Therefore, the presence of the extra fused benzene ring in **6** and **8**, has two significant effects: (1) it lowers the LUMO level, which leads to a red shift in the absorption spectra with regard to those of **5** and **7**, and (2) it generates an additional π molecular orbital (HOMO–1), which is mainly located on the benzo-quinoxaline fragment. Indeed, this orbital does not have an equivalent in complexes **5** and **7**. The low energy gap between HOMO and HOMO–1 in **6** and **8** prevents the selective excitation of an electron from these orbitals and this is expected to have relevant effects on the photophysical properties of these compounds.

To obtain information about the behavior of the studied compounds in their electronic excited states, the vertical excitation energies were calculated along with the orbitals involved in these excitations and their relative contributions to S_1_, S_2_, T_1_, T_2_, and T_3_ states (Tables 2 and S12–S15). In compounds **6** and **8** the differences in energy between S_0_ → S_1_ and S_0_ → S_2_ transitions are only 0.07 and 0.11 eV, respectively. These results suggest the roughly isoenergetic character of the vertical excitations to S_1_ and S_2_, which arise from d_H_ → π_L_* (^1^MLCT) and π_H-1_ → π_L_* (^1^LC) transitions (Table 2 and Figure 3). As far as compounds **5** and **7** are concerned, these excitations mainly correspond to d_H_ → π*_L_ (^1^MLCT) and d_H-1_ → π*_L_ (^1^MLCT) electronic structures, with energy differences of 0.17 and 0.18 eV, respectively. Excitations to higher singlet excited states will lead to ultrafast internal conversion processes to populate their lowest singlet excited states as it has been reported for other iridium complexes.^76^

**Table 2:** Vertical Excitation Energies (eV) of S_1_, S_2_, T_1_, T_2_, and T_3_ States and Percentages (Values in Parentheses) of Dominant Contributions to the Calculated Transitions for **5**, **6**, **7**, and **8** Obtained at the TD-DFT(SMD, Acetonitrile)/6-31G(d,p)//SDD Level

	Energy/eV		Electronic Structure	
State	**5**	**6**	**5**	**6**
S_1_	2.66	2.40		
S_2_	2.83	2.47		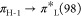
T_1_	2.31	1.50		
T_2_	2.45	2.23		
T_3_	2.73	2.46		

	Energy/eV		Electronic Structure	
State	**7**	**8**	**7**	**8**
S_1_	2.66	2.39		
S_2_	2.84	2.50		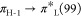
T_1_*	2.31	1.51		
T_2_	2.45	2.23		
T_3_	2.73	2.48		

The excitation energies for T_1_ states of **6** (1.50 eV) and **8** (1.51 eV) are low and very similar and they mainly originate from a high contribution (92%) of the π_H-1_ → π*_L_ transition for both compounds (Table 2). It is worth highlighting that the major fragmental contribution of the orbitals involved in these transitions is from the benzo-quinoxaline moieties (Figures 3 and S88), which indicates the ^3^LC/^3^ππ* character of these states. Both the rigidity of that scaffold and the large ^3^ππ* character of the observed transitions suggest the existence of long lifetimes for the T_1_ states, which is a desirable property in singlet oxygen photosensitizers.^56,77^ Furthermore, at such low energy, the nonradiative decay to the ground state makes it difficult to observe phosphorescence at room temperature. However, the ^3^LC/^3^ππ* character makes possible the microsecond lifetimes observed by TAS under these conditions. The reduction of the lifetime (about 10 times) of **6** and **8** by quenching with oxygen relative to the values under inert atmosphere indicates around 90% efficiency for energy transfer from the triplet state of the molecule to oxygen. Regarding vertically T_2_, T_3_ (^3^MLCT/^3^dπ*) states, they are much closer in energy to S_1_ (^1^MLCT/^1^dπ*) and S_2_ (^1^LC/^1^ππ*) states than T_1_ states (^3^LC/^3^ππ*). Therefore, an efficient spin–orbit coupling (SOC) due to the presence of Ir atom and the involvement of vibronic interactions between S_1_/S_2_ and T_2_/T_3_ are expected to produce an efficient intersystem crossing (ISC) in a nonadiabatic regime followed by a fast internal conversion (IC) to populate T_1_ state, as it has been observed in other [Ir(C^∧^N)_2_(N^∧^N)]^+^ compounds.^78−80^ It is worthy of note that the ISC between S_2_ (^1^LC/^1^ππ) and T_2_/T_3_ (^3^MLCT/^3^dπ*) states are expected to be very efficient because, in addition to the presence of the metal atom, the change from singlet to triplet excited states involves different molecular orbital configurations to achieve an effective overlapping, according to El-Sayed’s rule.^81^

Concerning T_1_ states of **5** and **7**, their excitation energies are the same (2.31 eV) and they have significantly larger multiconfigurational character than those of **6** and **8** (Tables 2 and S11; Figures 3 and S88). Moreover, they show the participation of nonrigid molecular fragments in the electronic structures of T_1_ states of **5** and **7** (Cp* and imidazole ligands). Thus, the rotational motions of these scaffolds facilitate the energy dissipation by nonradiative channels. In addition to this, the percentage of their main transitions (Table 2) suggests a lower ^3^ππ* character for **5** and **7** than for **6** and **8**. All of these factors will lead to a dramatic decrease in the lifetimes of **5** and **7**. This fact will have a negative effect on the utility of these compounds as singlet oxygen photosensitizers, which require a relatively long lifetime to allow the triplet–triplet energy transfer to oxygen. On the other hand, vertical T_2_ and T_3_ states show a clear ^3^MLCT/ ^3^dπ* nature and they are similar in composition and close in energy to the S_1_ and S_2_ (^1^MLCT/^1^dπ) states, respectively (Table 2). Thus, it is foreseeable that the large metal contribution on these states provokes a strong iridium-induced SOC followed by fast ISC and IC processes to populate the T_1_ state. Despite this, the absence of a change in orbital angular momentum during ISC processes between S_1_/S_2_ and T_2_/T_3_ states, as it was observed in compounds **6** and **8**, suggests that this process will be less effective than in the latter compounds, according to El-Sayed’s rule.

The adiabatic excitation energies (eV) calculated at the optimized excited states are provided in Table S16. It is important to note that the tiny energy difference between adiabatic states S_1_ and S_2_ of **6** (0.06 eV) indicates that these states are almost degenerate. Consequently, the electronic transitions associated with the luminescence emission will have π_L_* → d_H_ and π_L_* → π_H-1_ character for this compound. According to the existence of this ligand-based transition (ππ character) a relatively large luminescence intensity is expected as most of the luminescence emission in the transition metal complexes is due to the ligand-based luminescence.^82^ A similar behavior is expected for **8** since both compounds have similar orbitals and electronic transitions (Tables 2 and S13).

In the cases of compounds **5** and **7**, the adiabatic S_1_ and S_2_ states are clearly degenerate with a fairly ^1^MLCT/^1^dπ* character (Table S16). The absence of luminescence (at 10^–5^ M) observed for these compounds is related to the lack of a ligand-based radiative transition toward the ground state. Besides, the small energy gap between the lowest excited singlet and triplet states in these compounds suggests the existence of a direct S_1_/S_2_ (^1^dπ*) → T_1_ (^3^ππ*) ISC mechanism, but despite the change in the type of molecular orbital during this process, it is not expected to be very efficient since the multiconfigurational nature of the T_1_ states in **5** and **7** causes the involvement of a large number of orbitals and molecular fragments, with the resulting decrease in the efficiency of SOC. By way of conclusion, a comparative diagram showing the theoretical energy values for the lowest vertically excited states (S*_n_* and T*_n_*) and their experimental lifetimes for **7** and **8** is given in Scheme S2. The long lifetime determined for T_1_ of **8**, mainly due to its ^3^LC/^3^ππ* character, explains the outstanding ability of the pbpn derivatives (**series B**) for the generation of singlet oxygen.

### Biological Studies

#### Effect on Cell Viability

The potential of the complexes for the PDT of cancer was first evaluated by analyzing their activity against human lung cancer A549 cells in the dark and after photoactivation with blue (λ_irr_: 460 nm), green (λ_irr_: 530 nm), and red (λ_irr_: 655 nm) light. For each set of conditions, the concentration of compound required to reduce the number of viable cells by 50% (IC_50_) was initially established by MTT assays. All complexes were found to be active in the absence of light. Complexes **1**, **2**, **3**, and **4** exhibited IC_50,dark_ values between 2.4 and 8.0 μM (Table 3), and these are within the range of the anticancer agent cisplatin assayed under the same conditions (IC_50_ = 8.9 ± 2 μM). It should be noted that complexes with Imepip (**5** and **6**) and bzlm (**7** and **8**) ligands were the most active against A549 cells. In particular, the two complexes with bzlm exhibited IC_50_ values in the nanomolar range, thus demonstrating a high anticancer activity in dark conditions. The Imepip group alone was also tested, and it showed very low activity (IC_50_ > 50 μM). Therefore, the strong effect of **5** and **6** on cancer cell viability is almost certainly due to the complexes rather than to the release of the Imepip ligand.

**Table 3:** Effect of Complexes on A549 Cancer Cells Viability

	dark	blue light		green light		red light	
complex	IC_50_ (μM)	IC_50_ (μM)	PI^a^	IC_50_ (μM)	PI^a^	IC_50_ (μM)	PI^a^
**1**	8.0 ± 1	2.9 ± 0.4	2.8	5.8 ± 0.1	1.4	n.m.	n.m.
**2**	2.2 ± 0.9	0.0069 ± 0.001	323	0.032 ± 0.01	68.7	0.60 ± 0.04	3.7
**3**	4.9 ± 0.7	1.2 ± 0.1	4.1	2.5 ± 0.3	2.0	n.m.	n.m.
**4**	2.9 ± 1	0.0032 ± 0.002	932	0.010 ± 0.003	294	0.12 ± 0.05	25
**5**	0.83 ± 0.02	0.39 ± 0.1	2.1	0.52 ± 0.02	1.6	n.m.	n.m.
**6**	2.0 ± 0.1	0.0015 ± 0.0005	1317	0.011 ± 0.004	179	0.032 ± 0.01	63
**7**	0.77 ± 0.2	0.65 ± 0.2	1.2	0.47 ± 0.04	1.7	n.m.	n.m.
**8**	0.15 ± 0.05	0.00020 ± 0.0001	764	0.0021 ± 0.0004	71	0.030 ± 0.02	5.0
**9**	8.7 ± 1	5.3± 0.4	1.6	n.m.	n.m.	n.m.	n.m.
**10**	10 ± 0.3	5.6± 0.4	1.8	n.m.	n.m.	n.m.	n.m.
**Imepip**	68 ± 2	n.m.	n.m.	n.m.	n.m.	n.m.	n.m.
**Cisplatin**	8.9 ± 2	n.m.	n.m.	n.m.	n.m.	n.m.	n.m.

a A549 cells were incubated with the compounds for 4 h at 37 °C and then kept in the dark or exposed to blue (460 nm), green (530 nm), and red (655 nm) light for 1 h (24.1 J cm^–2^). Cell viability was assessed after 48 h of treatment by MTT assays. Data represents mean ± standard deviation (SD) of at least three independent experiments, each performed in triplicate. PI = IC_50,dark_/IC_50,light_. n.m.: not measured.

**Figure 4:**
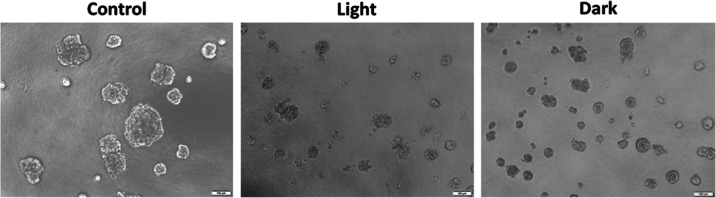
Representative microscopy images of A549 spheroids treated with complex **6** at 2 μM in the dark or at 0.01 μM with blue light irradiation. Untreated A549 cells seeded on Geltrex formed round mass spheroids (control). Images show the growth-suppressing effect of complex **6** on spheroids after 48 h of treatment. Scale bar: 100 μm.

The anticancer effect of complex **6** was also tested in multicellular A549 tumor spheroids. This three-dimensional (3D) cell culture model offers a closer representation of in vivo conditions of solid tumors, providing an approximation of the tumor microenvironment and cell–cell interactions. Consequently, it enables a more reliable assessment of the complex’s anticancer activity.^83^ Spheroids were treated for 48 h with different concentrations of complex **6**, either in the dark or with blue light photoactivation. Remarkably, an IC_50,dark_ of 1.2 ± 0.2 μM was obtained, which is similar to the value obtained in cell monolayers (Table 3). Upon photoactivation, the IC_50,light_ value determined in the 3D cultures (0.005 ± 0.001 μM) was only slightly higher than that obtained in the monolayer cultures, resulting in a PI of 240. Accordingly, as shown in Figure 4, a clear decrease in the spheroids size was observed both under dark and light conditions when exposed to concentrations close to the IC_50,dark_ (2 μM) or IC_50,light_ (0.01 μM). Moreover, spheroids were less refringent and showed a darker appearance than those untreated, which is characteristic of spheroids composed of dead cells. These data clearly demonstrate the growth-suppressing effect of complex **6** on 3D cultures, suggesting that complex **6** may exert antitumor activity in vivo.

The possible relationship between the biological activity and lipophilicity was analyzed by determining the log *P* values for complexes **5**, **6**, **7**, and **8** using the “shake flash” method^37,84,85^ (see the Experimental Section and Table S17). Regarding lipophilicity, it is considered that a value of log *P* < 4 means that a compound is suitable to be used as a drug.^86^ Thus, the values are adequate. However, there is no clear correlation between this value and the anticancer activity in the dark. Therefore, it appears that other factors influence the biological behavior of the compounds.

To assess the photodynamic behavior of the complexes, A549 cells were incubated with the complexes for 4 h to allow their internalization and then irradiated for 1 h with light of different wavelengths at a dose of 24.1 J cm^–2^. The light dose applied was comparable to those employed for the activation of other metal-based photocytotoxic compounds.^14,87^ Photoactivation with blue light led to a moderate enhancement of the activity of the complexes of the **series A** (**1**, **3**, **5**, and **7**), resulting in low phototoxicity indexes (PI = IC_50,dark_/IC_50,light_) ranging from 1.2 to 4.1. Conversely, photoactivation of the complexes with the pbpn ligand (**series B**) (**2**, **4**, **6**, and **8**) resulted in a marked reduction in cell viability, yielding PIs between 323 and 1317 (Table 3). Notably, the IC_50,light_ values for these complexes were in the very low nanomolar range (ranging from 6.9 nM for complex **2** to 0.20 nM for complex **8**), underscoring the exceptional anticancer properties of these photoactivated complexes. The high ability of these complexes to generate ^1^O_2_ is probably the reason for this outstanding photocytotoxicity.

To further elucidate the impact of π-expansion on the photodynamic activity of the complexes, the PIs of the four chloride complexes differing only in the extension of the C^∧^N ligands (**10**, **9**, **1**, and **2**) were compared (Table 3). Among them, only the pbpn derivative exhibited a remarkable photodynamic behavior, thus confirming the definitive influence of the presence of this ligand with the highest π-expansion on the complex activity.

Complexes with the pbpn ligand also showed a remarkable increase in their anticancer activity upon green light irradiation, with PI values between 71 and 294 along with very low IC_50,light_ values (from 32 to 2.1 nM) (Table 3). Photoactivation with red light, which has deeper tissue penetration,^88^ also increased their activity, especially in the case of complexes **4** and **6**, with PI values of 25 and 63, respectively. Although the photoactivation of complex **8** was lower upon red light irradiation, the resulting IC_50,light_ was still in the low nanomolar range (30 ± 19 nM).

To determine whether the anticancer activity was predominantly attributed to antiproliferative or to cytotoxic effects, A549 cells were treated with the complexes at their respective IC_50_ in the dark. Subsequently, a trypan blue exclusion test was performed to assess cellular viability. A significant proportion of cells (24, 54, 53, and 23% for complexes **2**, **4**, **6**, and **8**, respectively) exhibited uptake of trypan blue dye (Figure S92), indicating compromised cell membrane integrity and loss of cellular viability. The remaining cells predominantly exhibited cell shrinkage, detachment, reduced refractivity, and alterations in their morphology, which are distinctive attributes of cells undergoing death processes. Overall, these observations support that the complexes exert a cytotoxic effect on cancer cells.

The phototoxic activities of complexes **2**, **4**, **6**, and **8** were further evaluated against HeLa cervical carcinoma cells and PC-3 prostate adenocarcinoma cells, as well as nonmalignant fibroblast (1BR.3.G and MRC-5). For these experiments, a viability assay based on cellular DNA quantification was utilized, as studies on the mechanism of action of the complexes revealed that the compounds primarily target mitochondria (see below). Consequently, the results obtained using the MTT assay, which relies on mitochondrial metabolism, could reflect mitochondrial dysfunction rather than a decrease in cell viability. Specifically, the CyQUANT Direct Cell Proliferation Assay Kit (Molecular Probes) was employed.^89,90^ This assay incorporates a fluorescent DNA-binding dye along with a background suppression reagent. The background suppression reagent prevents the staining of dead cells or cells with compromised cell membranes, thereby enabling precise estimation of the viable cell population after treatments. The IC_50_ values obtained from the CyQUANT assay in A549 cells were compared to those obtained from the MTT assay (Tables 3 and 4), revealing a high level of consistency between the two assays, as statistically significant differences were observed only between complexes **6** and **8** under dark conditions. Overall, the results demonstrated that all of the compounds exhibited high photocytotoxicity against the three cancer cell lines, with IC_50,light_ values in the low nanomolar range. For complexes **4**, **6**, and **8**, phototoxicity indexes of approximately 1000 were obtained. Of particular significance are the PI values of 2034 and 2022 observed for complex **4** in A549 and PC-3 cells, respectively. Viability assays conducted on nontumor cells yielded comparable IC_50_ values, indicating limited selectivity of the compounds toward cancer cells. However, a comparison between the activity of the nonirradiated compounds against fibroblasts and their activity against cancer cells upon irradiation revealed significant photoselectivity. For instance, the IC_50,light_ values of complexes **2**, **4**, **6**, and **8** in A549 lung cancer cells were 980, 2480, 1750, and 1700 times lower, respectively, than the IC_50,dark_ values obtained in MRC-5 lung fibroblasts. Therefore, the doses that induce toxicity on cancer cells upon photoactivation are not toxic to nonirradiated healthy cells, offering the potential for highly specific light-controlled anticancer treatments.

**Table 4:** Cytotoxic Effect of Complexes against Human Cancer and Nonmalignant Cells

		cell line				
complex		A549	HeLa	PC-3	1BR.3.G	MRC-5
**2**	IC_50,dark_ (μM)	2.6 ± 0.8	2.7 ± 0.3	2.7 ± 0.2	3.2 ± 0.6	3.9 ± 0.8
	IC_50,light_ (μM)	0.0040 ± 0.002	0.037 ± 0.01	0.0089 ± 0.001	0.0062 ± 0.003	0.016 ± 0.004
	PI^a^	639	260	307	522	245
**4**	IC_50,dark_ (μM)	2.1 ± 0.6	1.55 ± 0.3	2.28 ± 0.7	2.00 ± 0.4	2.48 ± 0.2
	IC_50,light_ (μM)	0.0010 ± 0.0004	0.0042 ± 0.0009	0.0011 ± 0.0006	0.00080 ± 0.0004	0.0031 ± 0.001
	PI^a^	2034	370	2022	2451	790
**6**	IC_50,dark_ (μM)	0.33 ± 0.2	0.70 ± 0.27	1.16 ± 0.16	0.57 ± 0.06	0.70 ± 0.08
	IC_50,light_ (μM)	0.00040 ± 0.0002	0.00070 ± 0.0002	0.0012 ± 0.001	0.00090 ± 0.0003	0.0008 ± 0.0002
	PI^a^	911	1031	961	644	929
**8**	IC_50,dark_ (μM)	0.50 ± 0.2	0.51 ± 0.1	0.64 ± 0.1	0.50 ± 0.04	0.68 ± 0.1
	IC_50,light_ (μM)	0.00040 ± 0.0003	0.00080 ± 0.0002	0.00050 ± 0.0003	0.00050 ± 0.0002	0.00080 ± 0.0001
	PI^a^	1260	622	1179	942	863

a Cells were incubated with the compounds for 4 h at 37 °C and then kept in the dark or exposed to blue light (460 nm) for 1 h (24.1 J cm^–2^). Cell viability was assessed after 48 h of treatment by CyQUANT assays. Data represents mean ± SD of at least three independent experiments, each performed in triplicate. PI = IC_50,dark_/IC_50,light_.

Considering the results outlined above, it can be concluded that complexes of **series B**, in particular complexes **4**, **6**, and **8**, have great potential as PDT agents under irradiation with light sources of different wavelengths. Complex **6** provided the best PI and PI/dose values reported upon red light irradiation in the field of iridium chemistry (Table S18). Several values of PI/dose between 43 and 52 have been found for complexes **6** and **8** (A549, HeLa, and PC-3 cell lines) under blue light, which are among the highest reported for iridium complexes. Furthermore, a PI/dose value of 84 was obtained upon photoactivation of complex **4** with blue light in two different cancer cell lines (A549 and PC-3). To our knowledge, this is the third highest value reported to date for iridium derivatives (see Table S18 and references therein). Higher PI/dose values were obtained for the complex [Ir(N^∧^N)(ppy)_2_]PF_6_ (N^∧^N = 2-(quinoline-2-yl)1*H*-naphto[2,3-*d*]imidazole, 397 and 268 on HeLa and A459 cells, respectively),^24^ although the wavelength used was 425 nm, i.e., higher energy radiation and lower tissue penetrability than that used in this work. The other example is [Ir(R_1_-tpy)(R_2_-tpy)]^3+^ with R_1_ = Ph and R_2_ = pyren-1-yl (tpy = 2,2′;6′,2″-terpyridine) using visible light. If we consider complexes of other transition metals, three papers of ruthenium complexes^19,59,64^ and another with platinum derivatives^91^ have reported PI/dose values between 123 and 189 (Table S18). The outstanding results with very high PI values of McFarland work with tris(bidentate) derivatives of Ru and Os containing N^∧^N donor ligands that include several thiophene rings deserve special mention.^92−94^ We consider that our results are excellent and support the potential use of **4** and **6** as versatile PDT photosensitizers. Thus, they could be used to treat deep-seated tumors when activated with red light, but they could also be employed to tackle superficial malignancies under irradiation with blue light. In other words, these PSs offer the possibility of fine-tuning the precision of their cytotoxic action in terms of depth by simply changing the wavelength of the light source.

Next, we investigated whether the activity of complexes of **series B** could generate toxicity against red blood cells (RBC) as a result of their interaction with the cell membrane. The hemolytic activity of these compounds was assessed by determining the hemoglobin release from RBC exposed to different concentrations of the complexes under dark conditions. None of the four complexes exhibited any capacity to destabilize RBC membranes (hemolysis < 5%) at concentrations up to 10 μM (Table S19). These results indicate that all of the complexes have good blood compatibility at the photocytotoxic dose (IC_50,light_), which would prevent anemia in an eventual clinical treatment.

#### Intracellular ROS Production

**Figure 5:**
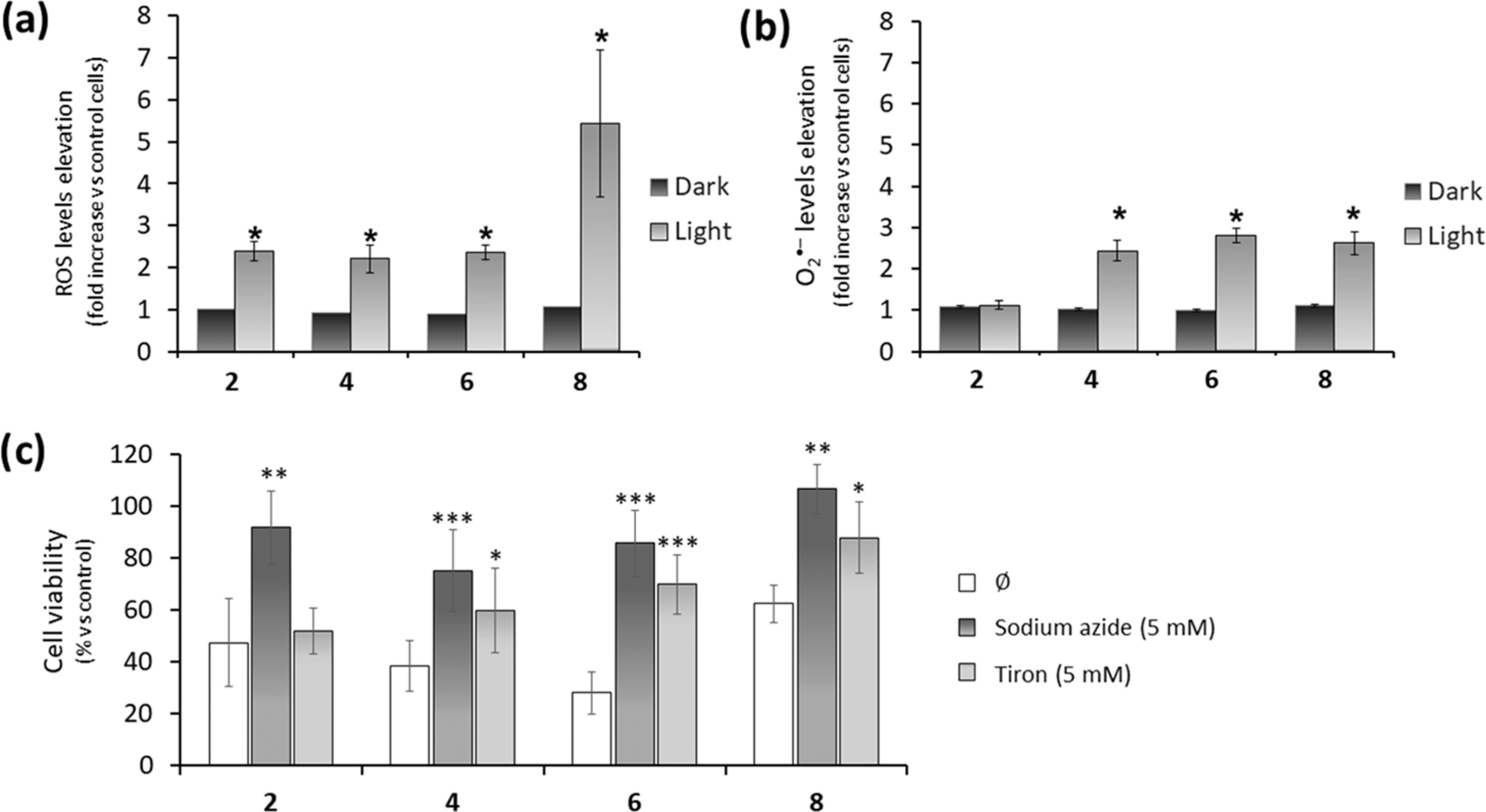
(a, b) Cellular ROS generation. A549 cells were treated with complexes **2**, **4**, **6**, and **8** at 10 nM for 4 h, followed by incubation in the dark or exposure to blue light (460 nm, 24.1 J cm^–2^) for an additional hour. General ROS (a) and superoxide anion (b) levels were measured with specific fluorescent probes by flow cytometry. Bars represent the mean fold increase (±standard deviation) relative to untreated control cells. * *p* < 0.05 versus control cells. (c) Effect of ROS on cell viability. A549 cells were treated with the complexes at their IC_50,light_ for 4 h, followed by blue light irradiation in the presence or absence of ROS scavengers (sodium azide for ^1^O_2_ or tiron for O_2_^•–^). Bars represent the mean percentage of viable cells 48 h later relative to the corresponding untreated control cells (±standard deviation). * *p* < 0.05, ** *p* < 0.01, and *** *p* < 0.001 versus cells exposed to the complexes without ROS scavenger (⌀). All conditions were tested by triplicate in three independent experiments.

The promising photocytotoxic properties of complexes **2**, **4**, **6**, and **8** encouraged us to investigate their mechanism of action. First, it was determined whether their photodynamic activity leads to a general elevation of ROS at the cellular level. To compare their ability to generate ROS, A549 cells were treated with the complexes at an equimolar concentration (10 nM). The cells were incubated with the complexes for 4 h and then maintained in the dark or exposed to blue light irradiation for 1 h. ROS generation was immediately determined by flow cytometry using the cell-permeable 2′,7′-dichlorodihydrofluorescein diacetate (CM-H_2_DCFDA) dye, which generates a derivative inside the cells that is oxidized by different ROS to fluorescent 2′,7′-dichlorodihydrofluorescein (DFC). Exposure of the cells to complexes in the dark did not modify the cellular ROS content. In contrast, after photoactivation, complexes **2**, **4**, and **6** generated 2.4-, 2.2-, and 2.4-fold elevations of ROS levels, respectively, as determined by the increase of the median fluorescence intensity of cells (Figure 5a). In the case of complex **8**, ROS levels increased by 5.6-fold, and this was the highest pro-oxidant activity observed in this study. This finding is consistent with the outstanding cytotoxicity of **8** after activation with blue light and its high level of ^1^O_2_ generation. Furthermore, additional studies were conducted to elucidate the specific types of ROS generated. In addition to singlet oxygen (see Figures 2 and 10), various ROS species such as superoxide anions (O_2_^•-^), hydroxyl radicals (^•^OH), hydrogen peroxide (H_2_O_2_), and peroxyl radicals (ROO^•^) are generated by PDT.^21,95^ Among these species, the superoxide anions are one of the primary and most cytotoxic ROS, capable of inducing irreversible damage to cellular components. Superoxide anion also contributes to the formation of hydrogen peroxide within cells, either spontaneously or facilitated by superoxide dismutase.^96^ Hydrogen peroxide exhibits long half-life and the ability to traverse biological membranes, causing damage in diverse cellular compartments.^97^ Consequently, the generation of superoxide anion radicals was assessed using a specific probe that produces an orange fluorescent product upon reaction with O_2_^•–^. Treatment with photoactivated complexes **4**, **6**, and **8** resulted in a significant increase in the mean fluorescence of the cells (by 2.4-, 2.8-, and 2.6-fold, respectively) indicating their capability to generate O_2_^•–^ (Figure 5b). However, O_2_^•–^ intracellular levels were not elevated by the treatments in the dark. Furthermore, upon light irradiation, complexes **4**, **6**, and **8** also induced a 1.7- to 2-fold increase in intracellular H_2_O_2_ levels (Figure S93). In contrast, no generation of either ROS was detected in the case of complex **2**, both in the light and in the dark.

To corroborate the impact of the ROS on cell death, A549 cells were incubated with complexes **2**, **4**, **6**, and **8** at their respective IC_50,light_ and subsequently activated with blue light in the absence or presence of NaN_3_ as selective ^1^O_2_ scavenger or tiron as O_2_^•–^ scavenger.^98^ Cell viability was determined after 48 h of treatment. As shown in Figure 5c, the phototoxicities of all of the complexes were significantly suppressed in the presence of NaN_3_, confirming the active role of ^1^O_2_ in cell death. Furthermore, the impact of complexes **4**, **6**, and **8** on cell viability was partially reversed by tiron, which is in agreement with their capacity to generate O_2_^•–^.

These findings collectively confirm the prominent role of type II photochemical processes in the photocytotoxicity of all of the complexes. Moreover, they demonstrate the involvement of O_2_^•–^ in the activity of complexes **4**, **6**, and **8**. This type I mechanism is less dependent on oxygen, making these complexes valuable candidates for targeting cancer cells within hypoxic microenvironments.^29^

Since the complexes also exhibited cytotoxic activity in the absence of irradiation, albeit at much higher concentrations, it was determined whether ROS generation was also involved in their mechanism of action in the dark. Treatment of the cells with complexes **2**, **4**, **6**, and **8** at their corresponding IC_50,dark_ generated a similar effect and led to a 2.6-, 2.8-, 2.6-, and 1.7-fold increase in cellular ROS content, respectively. Therefore, in the dark, the cytotoxic activity of compounds is also associated with a pro-oxidant activity that can effectively alter the redox balance of cells (Figure S94). This activity in the dark has also been observed for other [Cp*Ir(C^∧^N)L]^+^ complexes.^31,35^

#### Cellular Uptake and Distribution

ROS have a very short half-life and a radius of action that is limited to nearby molecules and structures.^12^ Thus, in addition to the amount of ROS generated, the efficacy of PDT is highly dependent on the specific cellular location in which they are produced.^99^ Compound **6** was selected to investigate the intracellular fate of the complexes since it showed a very good photodynamic behavior. First, its cellular internalization was analyzed, which may occur passively either by diffusion through the membrane or via channels, or alternatively by energy-dependent processes, such as active transport or endocytosis.^100^ The uptake mechanism was determined by specifically blocking the processes that require metabolic energy. To this end, the cells were treated for 1 h with the complex at low temperature (4 °C) or with a previous incubation with carbonyl cyanide *m*-chlorophenyl hydrazone (CCCP), a potent mitochondrial uncoupling agent that dissipates membrane potential, thus inhibiting adenosine triphosphate (ATP) production. Control cells were incubated at 37 °C without any inhibition to enable all types of internalization mechanisms. It was determined that the iridium content in control cells (87.6 ± 4 ng/10^6^ cells) was reduced by approximately 80% when cells were incubated both at 4 °C (18.0 ± 3 ng/10^6^ cells) or with CCCP pretreatment (17.6 ± 2 ng/10^6^ cells) (Figure S95), thus revealing that the internalization of the complex occurs mainly through an energy-dependent mechanism. However, it must be taken into account that these inhibition conditions may also affect the ability of cells to maintain the mitochondrial membrane potential (MMP) either by a reduction in metabolic activity at 4 °C, which inhibits the mitochondrial electron transport chain, or by the direct depolarization of the inner mitochondrial membrane by CCCP. Therefore, the low cellular accumulation of the complex could also be influenced by a reduced ability of mitochondria to retain cationic compounds.

**Figure 6:**
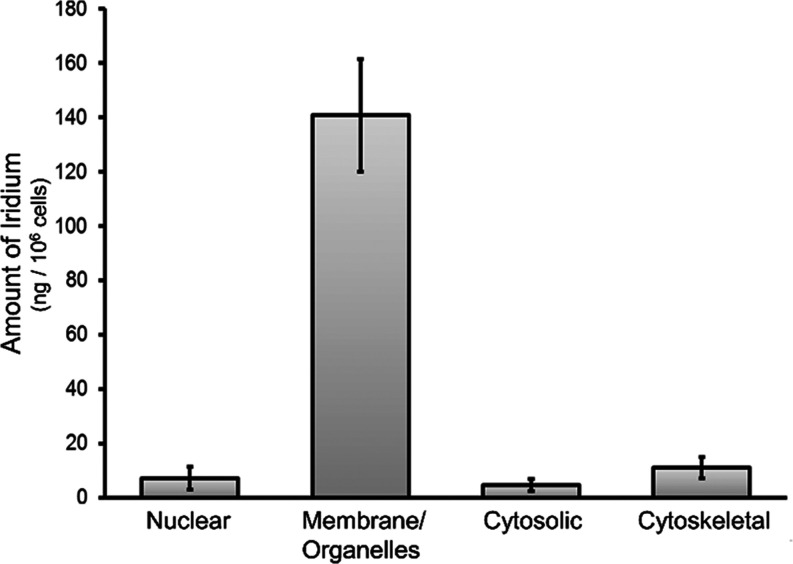
Subcellular distribution of complex **6**. The nuclear, membrane/organelles, cytosolic, and cytoskeletal fractions of A549 cells treated for 4 h with complex **6** at 10 μM were isolated, and the iridium content in each fraction was measured by ICP-MS. The mean values and standard deviation obtained in two independent experiments are shown.

The distribution of complex **6** inside the cells was investigated next. DNA^39,43,46^ as well as the mitochondria,^39,41,44,45,47,50,101,102^ endoplasmic reticulum (ER),^103,104^ or lysosomes^44,105^ have been described to be targets of iridium cyclometalated complexes. The cells were incubated with the complex at 10 μM for 4 h at 37 °C to allow its maximum accumulation and the major cellular compartments (nuclear, membrane/particulate, cytosolic, and cytoskeletal) were isolated. The amount of iridium in each fraction was determined by inductively coupled plasma mass spectrometry (ICP-MS). As represented in Figure 6, most of the iridium (140.8 ± 20 ng Ir/10^6^ cells) was found in the fraction containing the cell membranes and organelles. In contrast, only very small amounts of iridium were detected in the nuclear, cytosolic, and cytoskeletal fractions (7.1 ± 4 ng Ir/10^6^ cells, 4.6 ± 2 ng Ir/10^6^ cells, and 11.0 ± 4 ng Ir/10^6^ cells, respectively). Since complex **6** showed low interaction with the cell membrane in hemolysis experiments, its most likely cellular localization was membranous cell organelles. Specifically, as described for other cationic iridium-based compounds with lipophilic ligands, the complex was expected to accumulate in the mitochondria due to the negative potential across the inner mitochondrial membrane.^106^

As the complexes do not exhibit sufficient luminescence it was not possible to characterize their precise localization by fluorescence microscopy imaging. Therefore, to unravel their mechanism of action, the specific damage they exert on organelles was evaluated.

#### Mitochondrial Damage

##### Effect on the Mitochondrial Membrane Potential

**Figure 7:**
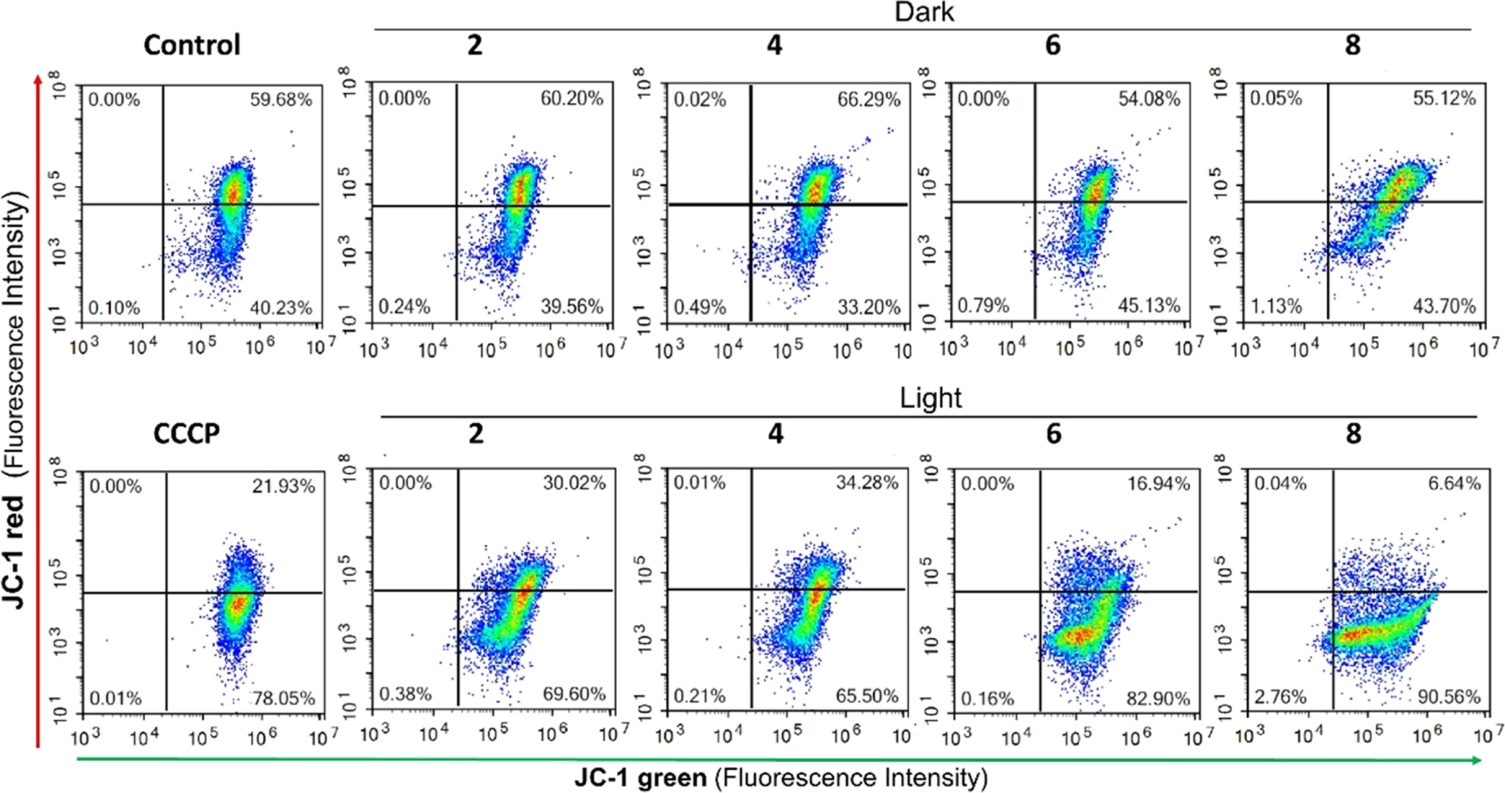
Flow cytometry analysis of the mitochondrial membrane potential. A549 cells were treated with complexes **2**, **4**, **6**, and **8** at a concentration of 10 nM for 4 h, followed by incubation in the dark or exposure to blue light irradiation for 1 h (460 nm, 24.1 J cm^–2^). The mitochondrial membrane uncoupler CCCP (5 μM) was used as a positive control to induce membrane depolarization. Cells incubated with medium alone were used as negative control. Plots represent fluorescence intensities of green JC-1 monomers and red JC-1 aggregates inside the cells. 10,000 cells were analyzed for each sample. The percentage of population in each quadrant is indicated. Loss of MMP can be detected by the reduction of red fluorescence within the cells.

MMP is generated by the process of electron transport to drive ATP production during oxidative phosphorylation, and it is considered a key indicator of mitochondrial activity. Beyond ATP generation, other processes including transport of molecules across the mitochondrial membrane, cell death signaling, and redox balance are dependent on the MMP. Therefore, disruption of the MMP can effectively compromise the cell viability.^107^ To determine the impact of the complexes on the MMP, A549 cells were treated with **2**, **4**, **6**, and **8** at 10 nM in the dark or under blue light irradiation and the mitochondrial membrane polarization was monitored by flow cytometry using the JC-1 cationic dye, which specifically accumulates inside healthy mitochondria and forms aggregates that emit red fluorescence. However, if the mitochondrial membrane is depolarized, JC-1 diffuses to the cytosol in the form of monomeric molecules that emit green fluorescence. As shown in Figure 7, red emission from JC-1 aggregates was detected in 59.7% of control untreated cells while 40.2% of the cells only emitted green fluorescence corresponding to JC-1 monomers. A very similar fluorescence pattern was observed in cells exposed to the complexes in the dark, thus indicating that the MMP was not altered under these conditions. In contrast, upon irradiation, cell fluorescence emission shifted to green and the percentage of cells with red fluorescence was reduced to 30.0% (**2**), 34.3% (**4**), 16.9% (**6**), and 6.6% (**8**), indicating a loss of MMP. Similar results were observed after the treatment of the cells with the CCCP mitochondrial uncoupler, which decreased the percentage of cells with red fluorescence to 21.9%. These results demonstrate that the photodynamic activity of complexes under investigation generates rapid inner membrane depolarization that potentially impairs mitochondrial functions. This effect was especially strong in cells exposed to complexes **6** and **8** and correlates well with their higher cytotoxicity upon photoactivation.

**Figure 8:**
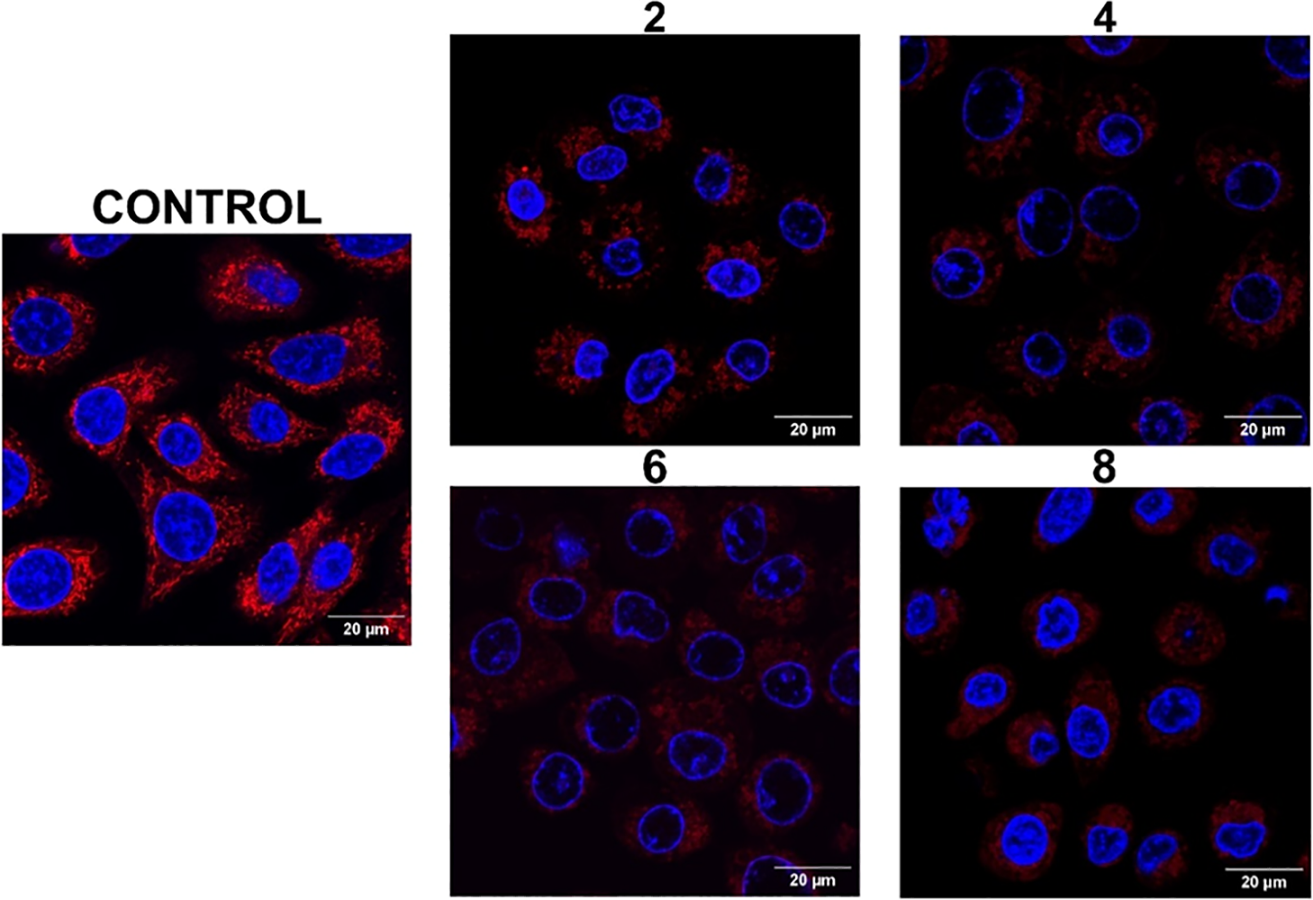
Effect of the photoactivated complexes on mitochondria. A549 cells were incubated with complexes **2**, **4**, **6**, and **8** for 4 h at the corresponding IC_50,light_ followed by photoactivation with a blue light (1 h, 460 nm, 24.1 J cm^–2^) or incubated with medium alone as a control. Mitochondria were stained with MitoTracker Red CMX and visualized in red (λ_ex_ = 554 nm; λ_em_ = 576 nm) by confocal microscopy. Nuclei of living cells were counterstained with Hoechst 33342 (λ_ex_ = 350 nm; λ_em_ = 461 nm) and visualized in blue.

The impact of the complexes on mitochondrial function was further confirmed using the MitoTracker Red CMX dye, which passively diffuses across the plasma membrane and accumulates in active mitochondria due to the MMP.^108^ A549 cells were incubated with complexes **2**, **4**, **6**, and **8** at a dose corresponding to the IC_50,light_, and the effect on mitochondria after photoactivation was immediately analyzed by confocal microscopy (Figure 8). The cells were localized by blue nuclear counterstain with Hoechst 33342. In untreated control cells, mitochondria showed a characteristic tubular morphology forming an interconnected network throughout the cytoplasm. In contrast, the number of fluorescent mitochondria was notably reduced in cells treated with the complexes and the remaining mitochondria mostly displayed a vesicular rounded shape, which is an indicator of mitochondrial damage.^108,109^ These results are consistent with those obtained with the JC-1 dye, further confirming that mitochondria are prime targets of the complexes and that mitochondrial dysfunction is a major mechanism by which they induce a cytotoxic effect.

##### Nicotinamide Adenine Dinucleotide (NAD) Oxidation

Nicotinamide adenine dinucleotide is a coenzyme involved in a wide range of oxidation/reduction reactions associated with cell metabolism.^110,111^ The reduced form of NAD (NADH) is an important electron source in the mitochondrial transport chain.^112^ Thus, oxidation of NADH in the mitochondria by exogenous causes may disrupt oxidative phosphorylation and contribute to mitochondrial dysfunction. During the past decade, several examples of the photocatalytic oxidation of NADH by iridium catalysts have been reported.^40,42,111,113^ Considering these reports, the Ir-photocatalyzed oxidation of NADH was studied for complexes **1**–**8** by UV–vis. The evolution of NADH (2.5 μM, complex/NADH ratio = 1:40) in the presence of the complexes (100 μM) was monitored in a H_2_O/DMSO (97.5:2.5 v/v) solvent mixture for **1**–**2** or H_2_O/MeOH (97.5:2.5 v/v) solvent mixture for **3**–**8** over a period of 8 h, at room temperature, in the dark and under blue light (470 nm). The decrease in intensity of the band at 340 nm was monitored as this corresponds to the disappearance of NADH (Figures S96–S104). In all cases, a very low level of NADH oxidation was observed in the dark. However, under blue light irradiation, there was a significant decrease in the intensity of the 340 nm band.

The reaction proceeded with first-order kinetics with respect to NADH. The TONs (Turnover Number, number of conversions per unit of catalyst) were measured for all complexes (Figure S105). In general, those PSs with pbpn ligands were more active photocatalysts than the corresponding complexes with pbpz ligands, except for the pair **1** and **2**, because **1** showed the highest activity in the photooxidation of NADH. The TON values are similar to those of other complexes reported in the literature.^40,42,114^ However, the values reported previously were not obtained under light irradiation. To our knowledge, the complexes reported here are the first half-sandwich iridium compounds to show a remarkable photocatalytic oxidation of NADH.

The photooxidation of NADH catalyzed by complexes **1**, **2**, **5**, and **6** was also monitored by ^1^H NMR spectroscopy both in the dark and under blue light (470 nm) irradiation, at room temperature (Figures S106–S114). The concentration of complexes was 1 mM and NADH was added (3.5 equiv) in a DMSO/D_2_O (9:1) solvent mixture for chlorido complexes due to their low solubility in aqueous media and in a DMSO/D_2_O (1:1) mixture for complexes **5** and **6**. In the absence of light, significant changes in the resonances were not observed. However, under blue light irradiation a new set of peaks was detected, and these were assigned to the oxidized form of NAD, that is, NAD^+^ (8.43 and 8.37 ppm). Nevertheless, the peak corresponding to the “Ir–H” species was not observed at any time—in contrast to other similar Ir complexes described previously.^40,42,114^ It is possible that in our case the hydrido derivative is either too reactive to be detected or the mechanism followed by these complexes is different from that observed by Sadler et al.^40^ Overall, these results revealed that oxidation of NADH after the treatment of the cells with the photoactivated complexes might contribute to the loss of the MMP and the subsequent mitochondrial dysfunction. However, there is not a perfect correlation between the photocatalytic activity and the cytotoxicity. In relation with that, it has been recently reported that the photocatalytic oxidation of NADH by certain complexes may be modified by interactions with other biomolecules.^115^

##### DNA Damage

Mitochondria are the only organelles outside the cell nucleus that possess their own genetic material. Mitochondrial DNA (mtDNA) is a small, circular, multicopy DNA molecule that encodes 13 polypeptide subunits of the complexes of the respiratory chain as well as tRNAs (tRNAs) and rRNAs (rRNAs) to translate them.^116^ As a consequence, mtDNA is essential for mitochondrial function. Importantly, mtDNA is not protected by histones and is more vulnerable to oxidative damage than nuclear DNA. Therefore, ROS overproduction by the complexes in close proximity to mtDNA could generate genomic aberrations that would contribute to mitochondrial dysfunction. The capacity of our complexes to interact with or damage DNA was evaluated by two different approaches.

**Figure 9:**
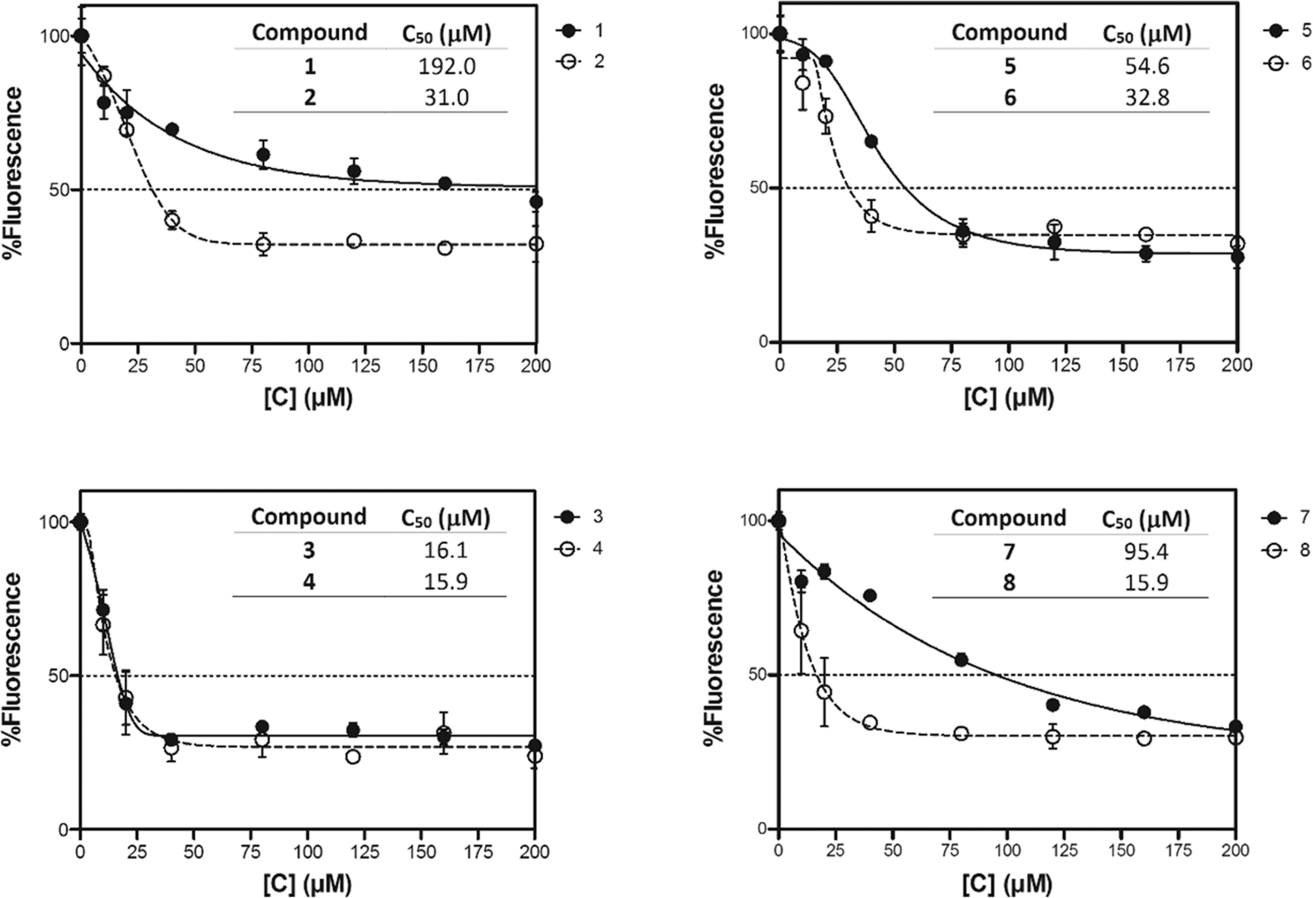
Competitive EtBr displacement assay with CT-DNA. Percentage fluorescence of EtBr interacting with CT-DNA treated with different compound concentrations. Each graph presents the mean and standard deviation of three independent experiments on pairs of compounds that differ in the C^∧^N ligand (pbpz or pbpn). C_50_ values obtained for each compound are indicated in the graph.

First, the capacity of the compounds to intercalate into DNA was assessed by testing their ability to displace the fluorescent dye ethidium bromide (EtBr) previously bound to double-stranded DNA. In this respect, a calf thymus DNA (CT-DNA) sample was saturated with an excess of EtBr. The samples were then exposed to a range of concentrations of complexes **1**–**8**. The fluorescence spectra showed that all complexes generated a concentration-dependent decline in the fluorescence of EtBr bound to CT-DNA (Figure S115). The concentration of each complex required to achieve a 50% reduction of the initial fluorescence (C_50_ value) was determined. All of the complexes intercalated into DNA with C_50_ values between 192 and 15.9 μM (Figure 9). It should be noted that the higher π-extension resulted in an increase of the intercalating properties for the pbpn derivatives, except for the complexes bearing the imidazole ligand (**3** and **4**), which displayed very similar C_50_ values (16.1 and 15.9 μM, respectively). These two compounds had the best DNA intercalating abilities, together with complex **8** (C_50_: 15.9 μM).

The interactions of the compounds that contain pbpn ligands with DNA in the dark and under irradiation were further analyzed by electrophoretic mobility assays using the circular plasmid pUC18, as described previously.^87^ This assay analyses the mobility shift of a plasmid DNA from a supercoiled (SC) conformation (with high electrophoretic mobility) to an open circular (OC) conformation (with reduced electrophoretic mobility due to single-strand breaks performed by damage-inducing agents, such as ROS). When the damage is severe, the DNA can become highly fractionated, and fractions elute from the gel. First, pUC18 plasmid was treated with serial dilutions of the complexes (from 25 to 0.5 μM) in order to find the concentration that generated a clear effect on the DNA for all compounds (data not shown). Based on the results, a concentration of 25 μM was chosen for the study. Doxorubicin (25 μM) was included in the experiment as a positive control of a classical DNA intercalator and treatment with 1.7% H_2_O_2_ and 20 μM FeCl_2_ was used as a positive control for ROS generation and nuclease activity.

**Figure 10:**
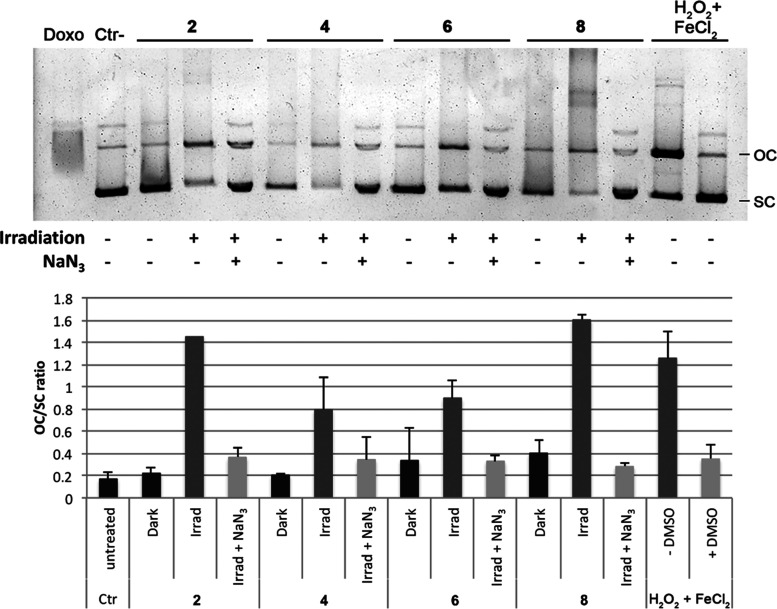
Electrophoretic mobility of pUC18 plasmid exposed to compounds **2**, **4**, **6**, and **8** (25 μM) for 1 h in dark conditions, under irradiation (irrad) (1 h, 460 nm, 24.1 J cm^–2^) or under irradiation in the presence of sodium azide (NaN_3_) as a ROS scavenger, as indicated under each lane. Doxorubicin (Doxo) was included as a reference DNA intercalator. Untreated pUC18 was used as a control (Ctr). H_2_O_2_ + FeCl_2_ were used to induce ROS-mediated nuclease activity on DNA, and DMSO was used as a hydroxyl radical scavenger. Supercoiled plasmid conformation (SC) and open circular conformation (OC) bands of two independent experiments were quantified by densitometry and the OC/SC ratio is shown in the graph.

As shown in Figure 10, in the presence of doxorubicin the plasmid electrophoretic mobility was greatly reduced and gave rise to a diffuse DNA band of lower mobility than the SC form of the untreated plasmid. Regarding the samples exposed to the compounds, the proportion of SC and OC forms of pUC18 treated in the dark were similar to those observed for the untreated sample, thus revealing that the compounds are not active against DNA without irradiation. In contrast, all compounds clearly induced a shift in the proportion of OC/SC forms of pUC18 under irradiation, demonstrating a nuclease activity. In addition, the photodynamic effect of the complexes was almost blocked by the presence of a singlet oxygen scavenger (sodium azide). Similar changes were observed on the treatment of pUC18 with H_2_O_2_ in the presence of FeCl_2_, which was also blocked by the hydroxyl radical scavenger DMSO.

The results outlined above indicate that the complexes can intercalate into DNA and induce oxidative DNA damage through oxygen singlet generation after photoactivation.

#### Lysosome Damage

Since the complexes were shown to accumulate in membranous organelles, their ability to cause lysosomal damage was also analyzed. The integrity of lysosomes was evaluated with Acridine Orange (AO), a cationic fluorescent dye that emits red fluorescence when accumulated in acidic compartments, such as lysosomes, and green fluorescence when localized in the cytosol and the cell nucleus.^117^ In control untreated cells and in cells treated with complexes **2**, **4**, **6**, and **8** at 10 nM in dark conditions, a dotted red fluorescent staining, corresponding to healthy lysosomes, was observed (Figure S116). However, upon blue light irradiation, the red fluorescent particles completely disappeared in cells exposed to complexes **4**, **6**, and **8**, indicating massive lysosomal damage, while they were only reduced in cells exposed to complex **2**, possibly due to the higher IC_50,light_ value of this complex. It should be noted that lysosomal photodamage could not be detected when the cells were treated with any of the complexes in the same conditions at the IC_50,light_ (data not shown), in contrast to that observed at the mitochondrial level. This finding suggests that lysosomes are not the main cellular target of the compounds, although they can also be damaged by their phototoxic activity. Nevertheless, it has been reported that lysosomal dysfunction, even at a low level, can significantly contribute to the cytotoxic effect of PSs directed at mitochondria.^118^

#### Cell Death Mechanism

**Figure 11:**
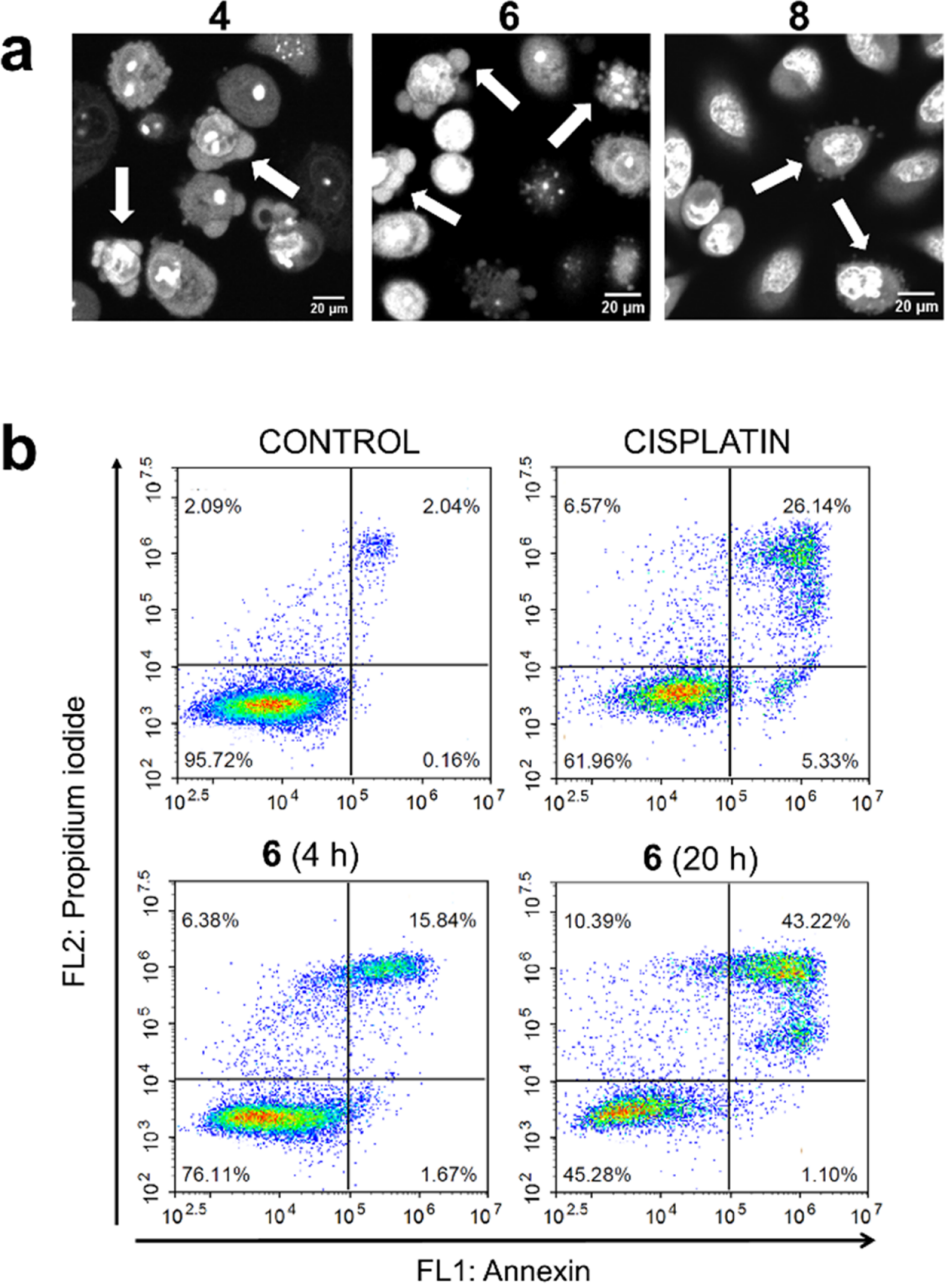
Cell death mechanism. (a) High-magnification microscopy images of A549 cells stained with AO after treatment with photoactivated complexes **4**, **6**, and **8** at 10 nM. Nuclear condensation and membrane blebbing, which are typical features of apoptosis, are indicated by white arrows. (b) Flow cytometry analysis of propidium iodide (FL2) and Annexin V-FITC (FL1) double staining after 4 and 20 h of treatment with photoactivated complex **6** at the IC_50,light_ × 5. Cisplatin (50 μM for 24 h) was included as positive control. The percentages of cells in each quadrant are indicated.

PDT triggers different modes of death that can be mainly classified into necrosis, apoptosis, and autophagy, although these mechanisms can coexist.^119^ In the microscopy images of the cells stained with AO it was apparent that, shortly after the treatments, some cells showed nuclear condensation and/or membrane blebbing, which are characteristic features of apoptosis (Figure 11a). The cell death pathway induced was further characterized for complex **6** by determining phosphatidylserine externalization to the outer cell membrane (indicative of apoptosis) and plasma membrane disruption (indicative of late apoptosis or necrosis) by annexin V-FITC (AN) and propidium iodide staining, respectively. The cells were treated with the complex at a concentration corresponding to 5 times the IC_50,light_ to induce death in a significant number of cells shortly after the photoactivation. Cisplatin was used as a positive control. As shown in Figure 11b, 95.7% of untreated cells (control) were negative for both propidium iodide and annexin V-FITC staining. After treatment with the photoactivated complex **6**, the proportion of dead cells increased in a time-dependent manner. After 4 h, 1.7% of the cells were found in early apoptosis (AN+; IP−), 6.4% in necrosis (AN–; IP+), and 15.8% in late apoptosis, which is also considered secondary necrosis (AN+; IP+).^119,120^ At 20 h, the populations in late apoptosis rose to 43.2% and the necrotic cells to 10.4%. In the case of cells treated with cisplatin for 24 h, 5.3% of the cells were in early apoptosis, 6.6% in necrosis, and 26.1% in late apoptosis.

This cell death mechanism involving both apoptosis and secondary necrosis is consistent with the proposed mechanism of action of the complexes. The prooxidative activity of the complexes has been found to generate rapid DNA damage as well as mitochondrial membrane permeabilization, as confirmed by MMP dissipation. This situation can promote cytochrome c release into the cytoplasm, where it triggers the activation of caspases and the subsequent intrinsic apoptosis pathway. Low levels of mitochondrial photodamage normally activate autophagy, in particular mitophagy, to recycle damaged mitochondria and protect cells from the release of proapoptotic proteins and mitochondrial ROS.^121^ However, the permeabilization of the mitochondrial membrane facilitates the redistribution of the complexes to other subcellular compartments, in particular to lysosomes, which are involved in autophagy, compromising this mechanism of cellular protection. The accumulation of ROS within the mitochondria can also cause necrosis as a result of a massive oxidative damage and depletion of ATP levels,^99^ an effect that intensifies with longer treatments. In addition, lysosomal damage can also activate necrosis by the release of lysosomal enzymes (such as cathepsins B, D, L) into the cytoplasm.^122^ Overall, the multitarget photocytotoxic activity of the complexes is highly effective in triggering cell death through both apoptosis and necrosis.

## Conclusions

Photodynamic therapy (PDT) is an approach that may reduce the side effects of chemotherapy by a spatiotemporal control of the cytotoxic activity. We have designed an approach involving the use of π-expansive ligands to obtain the first class of half-sandwich iridium cyclometalated derivatives that are active in PDT. A set of eight complexes with formulas [Cp*Ir(C^∧^N)Cl] and [Cp*Ir(C^∧^N)L]BF_4_ (L = imidazolyl-derived ligands, C^∧^N = 4,9,14-triazadibenzo[*a*,*c*]anthracene, pbpz, **series A**, 4,9,16-triazadibenzo[*a*,*c*]naphthacene, pbpn, **series B**) and also two other chloride complexes with progressive less π-expansion in the C^∧^N ligand were synthesized and characterized. The highest π-expansion present in pbpn was crucial to achieve a marked change in their photophysical properties and PDT behavior. A bathochromic shift in the absorption profile was observed for complexes with this ligand. Complexes of **series B**, and not those of **series A**, exhibited a long-lived triplet excited state (τ ∼ 1–2.5 μs) as verified by TAS. This state was strongly quenched by O_2_ and must be responsible for the high quantum yield in the generation of ^1^O_2_ (up to 99%) observed for complexes of **series B**. A rationale for the special behavior of the complexes of **series B** was obtained by TD-DFT calculations. Thus, complexes with the most π-expansive C^∧^N ligand possess nearly isoenergetic HOMO–1 and HOMO, with the former centered on the C^∧^N ligand, a situation that imparts ^3^LC/^3^ππ* character to the T_1_ state and increases the respective lifetime.

Regarding the activity of the complexes at the cellular level, it was observed that the more π-extended C^∧^N ligand (pbpn) confers outstanding properties to the complexes for cancer PDT, particularly for complexes **4**, **6**, and **8**. Notably, the photocytotoxic activity of complex **6** also was confirmed using spheroids, which provide a closer model of in vivo conditions of solid tumors. This behavior must be due to the existence of the microsecond T_1_ state that is able to generate ^1^O_2_ very efficiently. It was found that the cytotoxic activity of the complexes increases by up to 2034 times upon irradiation with blue light. Furthermore, the compounds partially retain their photoactivation properties on using green or red light, which have a greater capacity to penetrate tissues and allow the photocontrolled treatment of tumors located in deeper tissues. To the best of our knowledge, complex [Cp*Ir(pbpn)(Im)]BF_4_ (**4**), under activation with blue light (λ = 460 nm), provided the third highest PI/dose value reported for iridium derivatives and complexes **6** and **8** also gave very high PI and PI/dose values. Complex **6** also gave the best PI and PI/dose values reported with red light in the field of iridium chemistry. It was demonstrated that the cellular uptake of the complexes requires an active metabolism and functional mitochondria and that, once internalized, the compounds preferentially accumulate in these cell organelles. In particular, the compounds exerted rapid photodamage at the mitochondrial level, and this generated mitochondrial membrane depolarization, NADH photocatalytic oxidation, and oxidative cleavage of DNA. Interestingly, it was observed that complexes can also generate severe lysosomal dysfunction. As a consequence, the compounds activate a multitarget cell death mechanism that involves both apoptosis and necrosis and compromises cell survival processes such as autophagy.

To our knowledge, this is the first report on half-sandwich iridium cyclometalated derivatives that are active in PDT and have outstanding PI values. The extra fused ring of the π-expansive pbpn ligand is crucial for the photocytotoxic activity. Although the cytotoxicity of the compounds in the dark is relatively high, this drawback could be circumvented by administering low doses, corresponding to the IC_50,light_ of the complexes, ensuring that they are only active in tumors after controlled light irradiation. In any case, work is ongoing to improve this aspect and we believe that this work opens a new avenue for the generation of promising chemotherapeutic agents for the treatment of human cancers.

## Experimental Section

### Materials

IrCl_3_·*x*H_2_O was purchased from Johnson Matthey and used as received. The starting dimer [(η^5^-C_5_Me_5_)Ir(μ-Cl)Cl]_2_ was prepared according to the reported procedure.^123^ Proligands H-pbpq, H-pbpz, and H-pbpn were prepared by reaction of benzo[*h*]quinoline-5,6-dione with ethylene diamine (H-pbpq), *o-*phenylenediamine (H-pbpz), or 2,3-diaminonaphthalene (H-pbpn) according to the literature methods.^19^ Benzo[*h*]quinoline-5,6-dione was prepared as reported.^19^ The reagent 1,2,3,4,5-pentamethylcyclopentadiene (>93%) was purchased from Tokyo Chemical Industry (TCI); benzo[*h*]quinoline, iodopentoxide, 2,3-diaminonaphthalene and silver trifluoroacetate were purchased from Alfa Aesar; *o*-phenylendiamine from Sigma-Aldrich; imidazole (Im), *N*-benzylimidazole (bzIm), and silver tetrafluoroborate from Acros; and sodium carbonate from Panreac. All of them were used without further purification. (*N*-Ethylpiperidyl)imidazole (Imepip) was prepared according to the literature.^124^ Deuterated solvents were purchased from Eurisotop. Conventional solvents such as diethyl ether (Fisher Scientific), acetone (Fisher Scientific), dimethyl sulfoxide (Scharlau), *N*,*N*-dimethylformamide (Scharlau), ethanol (Scharlau), methanol (Scharlau), dichloromethane (Scharlau), toluene (Across Organics), and acetic acid (Scharlau) were degassed and in some cases distilled prior to use. Tetrabutylammonium hexafluorophosphate ([*n*Bu_4_N][PF_6_]) was purchased from Acros. Sodium hydroxide and hydrochloride acid were purchased from Panreac.

### Experimental Details

All synthetic manipulations were performed under an inert, oxygen-free, dry nitrogen atmosphere using standard Schlenk techniques. All metal complexes were synthesized in dark conditions and protected from light using aluminum foil throughout each step of synthesis, isolation, and characterization. Solvents were dried and distilled under nitrogen atmosphere before use. Elemental analyses were performed on a FlashEA112 microanalyzer (ThermoFinnigan). FAB+ and ESI+ MS (mass spectrometry) data were recorded on a Thermo MAT95XP mass spectrometer using dichloromethane (DCM) or methanol as the sample solvent. UV–vis absorption spectra were recorded on a Secomam Uvikon XS spectrophotometer using the LabPower Junior program. Fluorescence excitation and emission spectra were recorded on a PTI Quanta Master TM spectrofluorometer from Photon Technology International (PTI) equipped with a 75 W xenon short arc lamp and a model 814PTM detection system. Multinuclear nuclear magnetic resonance (NMR) spectra were recorded at 298 K on a Varian Unity Inova 400 or on a Varian Inova 500 spectrometer. Chemical shift values (δ) are reported in ppm (parts per million) and coupling constants (*J*) in hertz. The splitting of proton resonances is defined as follows: s = singlet, d = doublet, t = triplet, q = quadruplet, m = multiplet, bs = broad singlet. 2D-NMR spectra such as ^1^H–^1^H gCOSY, ^1^H–^13^C gHSQC and ^1^H–^13^C gHMBC were recorded using standard pulse–pulse sequences. ^1^H NMR chemical shifts were internally referenced to C*H*Cl_3_ (7.26 ppm) for chloroform-*d*_1_, to (C*H*D_2_)(CD_3_)SO (2.50 ppm) for DMSO-*d*_6_ or to (C*H*D_2_)(CD_3_)CO (2.05 ppm) for acetone-*d*_6_ via the residual proton solvent resonances. The probe temperature (±1 K) was controlled by a standard unit calibrated with a methanol reference. All NMR data processing was performed using MestReNova version 12.0.0. pH measurements were carried out with a CRISON 522 conductimeter, connected to a conductivity cell CRISON 52 92 with platinum electrodes.

Unless otherwise stated, reagents and solvents were of reagent quality and commercially available.

The purity of the final compounds was determined by reversed-phase high-performance liquid chromatography (HPLC) analyses on a LiquidPurple ODS C18 column (250 × 4.6 mm, 5 μm, flow rate: 1 mL min^–1^) using linear gradients of 0.1% formic acid in Milli-Q H_2_O (A) and 0.1% formic acid in MeCN (B) and coupled to a UV–vis detector. The HPLC column was maintained at 25 °C. The detection wavelength was 260 nm. All final compounds were >95% pure by this method.

#### Atom Numbering

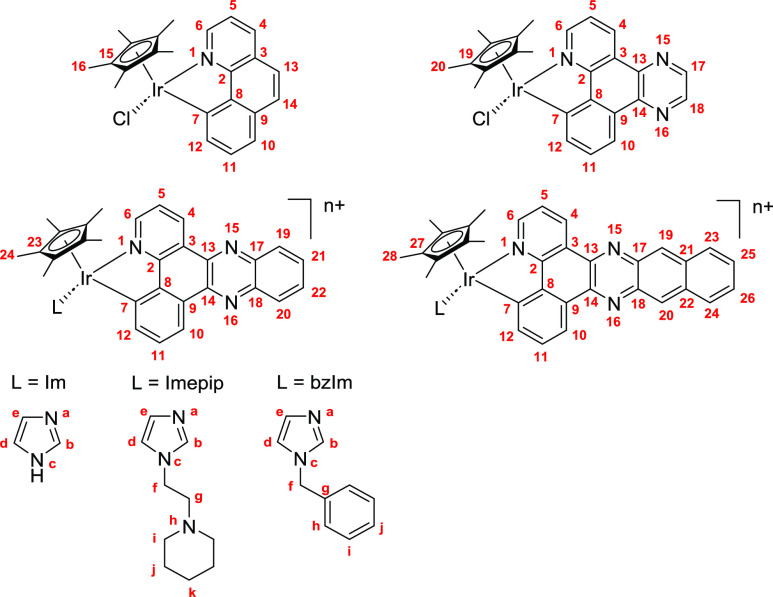



##### [(η^5^-C_5_Me_5_)IrCl(pbpz)] (**1**)

**1** was synthesized using a procedure adapted from the literature.^125^ In a 50 mL Schlenk flask, a solution of [(η^5^-C_5_Me_5_)Ir(μ-Cl)Cl]_2_ (0.50 g, 0.6 mmol, 1.0 equiv), 4,9,14-triazadibenzo[*a*,*c*]anthracene (H-pbpz, 0.36 g, 1.2 mmol, 2.0 equiv), Na_2_CO_3_ (0.13 g, 1.3 mmol, 2.1 equiv), and AgOCOCF_3_ (0.28 g, 1.3 mmol, 2.1 equiv) in degassed DCM (30 mL) was stirred at ambient temperature for 4.5 h. The resulting precipitate (AgCl) was removed by filtration over Celite. To recover all of the product, the residue was washed with degassed DCM until the filtrate was colorless. The filtrate was concentrated in vacuo until dryness. Then, the red solid was washed with diethyl ether (3 × 10 mL). The product was purified by flash chromatography with Celite as stationary phase and DCM as mobile phase. The filtrate was concentrated in vacuo until dryness to give a red solid (0.67 g, 1.0 mmol, 83%). ^1^H RMN (500 MHz, CDCl_3_): δ 9.42 (dd, ^3^*J* = 7.9 Hz, ^4^*J* = 1.4 Hz, 1H, H^4^), 9.04 (dd, ^3^*J* = 5.4 Hz, ^4^*J* = 1.4 Hz, 1H, H^6^), 8.80 (dd, ^3^*J* = 7.8 Hz, ^4^*J* = 0.9 Hz, 1H, H^10^), 8.37 (dd, *J* = 8.5, 1.8 Hz, 1H, H^19^), 8.31 (dd, *J* = 8.2, 1.8 Hz, 1H, H^20^), 8.19 (dd, ^3^*J* = 7.4 Hz, ^4^*J* = 0.9 Hz, 1H, H^12^), 7.88 (qd, *J* = 6.7, 1.7 Hz, 2H, H^21^ and H^22^), 7.71 (t, ^3^*J* = 7.6 Hz, 1H, H^11^), 7.59 (dd, ^3^*J* = 7.9, 5.4 Hz, 1H, H^5^), 1.78 (s, 15H, H^24^). ^13^C{^1^H} RMN (126 MHz, CDCl_3_): δ 161.4 (C, C^7^), 161.1 (C, C^2^), 152.1 (CH, C^6^), 144.1 (C, C^14^), 142.6 (C, C^17^ or C^18^), 142.2 (C, C^8^), 141.7 (C, C^17^ or C^18^), 141.1 (C, C^13^), 136.7 (CH, C^12^), 134.4 (CH, C^4^), 131.7 (C and CH, C^9^ and C^11^), 130.5 (CH, C^21^ or C^22^), 130.0 (CH, C^21^ or C^22^), 129.8 (CH, C^19^), 129.5 (CH, C^20^), 126.5 (C, C^3^), 122.7 (CH, C^5^), 118.9 (CH, C^10^), 88.8 (5C, C^23^), 9.2 (5CH_3_, C^24^). Elemental Analysis calculated for C_29_H_25_ClIrN_3_ (643.20 g/mol): C 54.16, H 3.92, N 6.53; found: C 54.01, H 3.90, N 6.52. MS (FAB+, DCM): *m*/*z* calculated for [(η^5^-C_5_Me_5_)_2_Ir_2_Cl(pbpz)_2_]^+^: 1249.30, found: 1249.40; *m*/*z* calculated for [(η^5^-C_5_Me_5_)IrCl(pbpz)]^+^: 643.14, found: 643.18; *m*/*z* calculated for [(η^5^-C_5_Me_5_)Ir(pbpz)]^+^: 608.17, found: 608.39.

##### [(η^5^-C_5_Me_5_)IrCl(pbpn)] (**2**)

**2** was prepared following a similar procedure to that used for **1**. Amounts were as follows: [(η^5^-C_5_Me_5_)Ir(μ-Cl)Cl]_2_ (0.52 g, 0.7 mmol, 1.0 equiv), 4,9,16-triazadibenzo[*a*,*c*]naphthacene (H-pbpn, 0.44 g, 1.3 mmol, 2.0 equiv), Na_2_CO_3_ (0.14 g, 1.3 mmol, 2.0 equiv), and AgOCOCF_3_ (0.28 g, 1.3 mmol, 2.0 equiv) in degassed DCM (30 mL). The product was obtained as a dark red solid (0.81 g, 1.1 mmol, 90%). ^1^H RMN (400 MHz, CDCl_3_): δ 9.38 (dd, ^3^*J* = 7.9 Hz, ^4^*J* = 1.4 Hz, 1H, H^4^), 9.01 (dd, ^3^*J* = 5.5 Hz, ^4^*J* = 1.4 Hz, 1H, H^6^), 8.90 (s, 1H, H^19^), 8.86 (s, 1H, H^20^), 8.79 (dd, ^3^*J* = 7.8 Hz, ^4^*J* = 1.0 Hz, 1H, H^10^), 8.18 (dd, ^3^*J* = 7.5 Hz, ^4^*J* = 1.0 Hz, 1H, H^12^), 8.14–8.17 (m, 2H, H^23^ and H^24^), 7.69 (t, ^3^*J* = 7.6 Hz, 1H, H^11^), 7.58–7.55 (m, 3H, H^5^, H^25^ and H^26^), 1.78 (s, 15H, H^28^). ^13^C{^1^H} RMN (101 MHz, CDCl_3_): δ 161.8 (C, C^2^), 161.7 (C, C^7^), 152.3 (CH, C^6^), 145.0 (C, C^14^), 142.4 (C, C^13^), 142.3 (C, C^8^), 139.0 (C, C^17^ or C^18^), 138.4 (C, C^17^ or C^18^), 137.2 (CH, C^12^), 134.5 (C, C^21^ or C^22^), 134.4 (CH, C^4^), 134.1 (C, C^21^ or C^22^), 131.9 (C, C^9^), 131.8 (CH, C^11^), 128.67 (CH, C^23^ or C^24^), 128.62 (CH, C^23^ or C^24^), 127.8 (CH, C^19^), 127.7 (CH, C^20^), 127.0–126.8 (3C, CH and C, C^25^, C^26^ and C^3^), 122.8 (CH, C^5^), 119.3 (CH, C^10^), 88.8 (5C, C^27^), 9.2 (5CH_3_, C^28^). Elemental Analysis calculated for C_33_H_27_ClIrN_3_ (693,26 g/mol): C 55.17, H 3.93, N 6.06; found: C 55.11, H 3.92, N 6.06. MS (FAB+, DCM): *m*/*z* calculated for [(η^5^-C_5_Me_5_)Ir(pbpn)]^+^: 658.18, found: 658.25.

##### [(η^5^-C_5_Me_5_)Ir(Im)(pbpz)]BF_4_ (**3**)

In a 50 mL Schlenk flask, a solution of **1** (0.1241 g, 0.2 mmol, 1.0 equiv), imidazole (Im, 0.0128 g, 0.2 mmol, 1.0 equiv), and AgBF_4_ (0.0404 g, 0.2 mmol, 1.1 equiv) in degassed DCM (15 mL) was stirred at ambient temperature for 4 h. The resulting precipitate (AgCl) was removed by filtration over Celite. To recover all of the product, the residue was washed with degassed DCM until the filtrate was colorless. The filtrate was concentrated in vacuo until dryness. Then, the yellow solid was washed with diethyl ether/pentane (50/50, v/v) (3 × 15 mL). The product was purified by flash chromatography with Celite as stationary phase and DCM as mobile phase. The filtrate was concentrated in vacuo until dryness to give a yellow solid (0.0968 g, 0.12 mmol, 68%). ^1^H RMN (400 MHz, CDCl_3_): δ 10.93 (s, 1H, H^c^), 9.48 (d, ^3^*J* = 7.8 Hz, 1H, H^4^), 9.25 (d, ^3^*J* = 4.6 Hz, 1H, H^6^), 8.90 (dd, ^3^*J* = 7.9 Hz, ^4^*J* = 0.8 Hz, 1H, H^10^), 8.34 (m, 1H, H^19^), 8.29 (m, 1H, H^20^), 8.26 (dd, ^3^*J* = 7.3 Hz, ^4^*J* = 1.0 Hz, 1H, H^12^), 7.86 (qd, *J* = 6.8, 1.7 Hz, 2H, H^21^ and H^22^), 7.84–7.76 (m, 3H, H^5^, H^11^ and H^b^), 6.73 (s, 1H, H^d^), 6.49 (s, 1H, H^e^), 1.70 (s, 15H, H^24^). ^13^C{^1^H} RMN (101 MHz, CDCl_3_): δ 160.6 (C, C^2^), 158.8 (C, C^7^), 152.5 (CH, C^6^), 143.2 (C, C^14^), 142.53 (C, C^17^ or C^18^), 142.46 (C, C^8^), 141.9 (C, C^17^ or C^18^), 140.5 (C, C^13^), 138.5 (CH, C^b^), 135.7 (CH, C^12^), 135.6 (CH, C^4^), 132.1 (CH, C^11^), 132.0 (C, C^9^), 131.0 (CH, C^21^ or C^22^), 130.5 (CH, C^21^ or C^22^), 129.63 (CH, C^19^), 129.59 (CH, C^20^), 128.3 (CH, C^e^), 126.8 (C, C^3^), 124.3 (CH, C^5^), 120.1 (CH, C^10^), 118.2 (CH, C^d^), 90.0 (5C, C^23^), 8.9 (5CH_3_, C^24^). Elemental Analysis calculated for C_32_H_29_BF_4_IrN_5_ (762.63 g/mol): C 50.40, H 3.83, N 9.18; found: C 50.38, H 3.82, N 9.16. MS (FAB+, MeOH): *m*/*z* calculated for [(η^5^-C_5_Me_5_)Ir(Im)(pbpz)]^+^: 676.21, found: 676.32; *m*/*z* calculated for [(η^5^-C_5_Me_5_)Ir(pbpz)]^+^: 608.17, found: 608.39.

##### [(η^5^-C_5_Me_5_)Ir(Im)(pbpn)]BF_4_ (**4**)

Following the procedure to synthesize complex **3**, complex **4** was prepared from a solution of **2** (0.0972 g, 0.1 mmol, 1.0 equiv), imidazole (Im, 0.0090 g, 0.1 mmol, 1.0 equiv), and AgBF_4_ (0.0362 g, 0.1 mmol, 1.1 equiv) in degassed DCM (12 mL). The product was obtained as a dark red solid (0.0782 g, 0.094 mmol, 69%). ^1^H RMN (500 MHz, (CD_3_)_2_CO): δ 11.84 (s, 1H, H^c^), 9.61 (dd, ^3^*J* = 5.4 Hz, ^4^*J* = 1.2 Hz, 1H, H^6^), 9.33 (dd, ^3^*J* = 7.9 Hz, ^4^*J* = 1.1 Hz, 1H, H^4^), 8.78 (s, 2H, H^19^ and H^20^), 8.71 (d, ^3^*J* = 7.5 Hz, 1H, H^10^), 8.48 (dd, ^3^*J* = 7.3 Hz, ^4^*J* = 0.7 Hz, 1H, H^12^), 8.16 (m, 2H, H^23^ and H^24^), 7.93 (m, 2H, H^5^ and H^b^), 7.80 (t, ^3^*J* = 7.6 Hz, 1H, H^11^), 7.53 (m, 2H, H^25^ and H^26^), 7.13 (s, 1H, H^d^), 7.03 (s, 1H, H^e^), 1.82 (s, 15H, H^28^). ^13^C{^1^H} RMN (126 MHz, (CD_3_)_2_CO): δ 161.9 (C, C^2^), 160.5 (C, C^7^), 154.7 (CH, C^6^), 144.7 (C, C^14^), 143.5 (C, C^8^), 142.4 (C, C^13^), 139.4 (C, C^21^ or C^22^), 139.3 (CH, C^b^), 138.9 (C, C^17^ or C^18^), 137.6 (CH, C^12^), 136.0 (CH, C^4^), 135.4 (C, C^17^ or C^18^), 135.0 (C, C^21^ or C^22^), 132.8 (CH, C^11^), 132.7 (C, C^9^), 129.9 (CH, C^e^), 129.33 (CH, C^23^ or C^24^), 129.26 (CH, C^23^ or C^24^), 128.5 (CH, C^19^ or C^20^), 128.4 (CH, C^19^ or C^20^), 128.1 (CH, C^25^ or C^26^), 127.9 (CH, C^25^ or C^26^), 125.5 (CH, C^5^), 120.6 (CH, C^10^), 119.6 (C, C^3^), 119.4 (CH, C^d^), 91.0 (5C, C^27^), 9.0 (5CH_3_, C^28^). Elemental analysis calculated for C_36_H_31_BF_4_IrN_5_ (812.69 g/mol): C 53.21, H 3.84, N 8.62; found: C 53.12, H 3.83, N 8.60. MS (FAB+, MeOH): *m*/*z* calculated for [(η^5^-C_5_Me_5_)Ir(Im)(pbpn)]^+^: 725.22, found: 726.48; *m*/*z* calculated for [(η^5^-C_5_Me_5_)Ir(pbpn)]^+^: 658.18, found: 658.20.

##### [(η^5^-C_5_Me_5_)Ir(Imepip)(pbpz)]BF_4_ (**5**)

Following the procedure to synthesize complex **3**, complex **5** was prepared from a solution of **1** (0.1323 g, 0.21 mmol, 1.0 equiv), (*N*-ethylpiperidyl)imidazole (Imepip, 0.0411 g, 0.22 mmol, 1.0 equiv), and AgBF_4_ (0.0462 g, 0.24 mmol, 1.1 equiv) in degassed DCM (15 mL). The product was obtained as a yellow solid (0.1530 g, 0.051 mmol, 85%). ^1^H RMN (500 MHz, CDCl_3_): δ 9.42 (d, ^3^*J* = 7.8 Hz, 1H, H^4^), 9.38 (m, 1H, H^6^), 8.83 (d, ^3^*J* = 8.0 Hz, ^4^*J* = 0.8 Hz, 1H, H^10^), 8.23 (m, 2H, H^19^ and H^20^), 8.17 (d, ^3^*J* = 6.0 Hz, 1H, H^12^), 8.08 (s, 1H, H^b^), 7.83 (m, 3H, H^5^, H^21^ and H^22^), 7.73 (t, ^3^*J* = 6.8 Hz, 1H, H^11^), 6.83 (s, 1H, H^d^), 6.43 (s, 1H, H^e^), 4.39 (m, 2H, H^f^), 3.09 (m, 2H, H^g^), 2.85 (m, 4H, H^i^), 1.72 (s, 19H, H^24^ and H^j^), 1.52 (m, 2H, H^k^). ^13^C{^1^H} RMN (126 MHz, CDCl_3_): δ 160.4 (C, C^2^), 158.8 (C, C^7^), 153.5 (CH, C^6^), 143.3 (C, C^14^), 142.7 (C, C^17^ or C^18^), 142.6 (C, C^17^ or C^18^), 142.0 (C, C^8^), 140.6 (C, C^13^), 140.1 (CH, C^b^), 135.7 (CH, C^12^), 135.5 (CH, C^4^), 132.0 (C and CH, C^9^ and C^11^), 130.9 (CH, C^21^ or C^22^), 130.5 (CH, C^21^ or C^22^), 129.8 (2C, C^19^ and C^20^), 129.6 (CH, C^e^), 126.7 (C, C^3^), 124.7 (CH, C^5^), 121.3 (CH, C^d^), 120.0 (CH, C^10^), 90.1 (5C, C^23^), 56.9 (CH_2_, C^g^), 53.7 (2CH_2_, C^i^), 43.1 (CH_2_, C^f^), 23.7 (2CH_2_, C^j^), 22.2 (CH_2_, C^k^), 9.0 (5CH_3_, C^24^). Elemental Analysis calculated for C_39_H_42_BF_4_IrN_6_ (873.82 g/mol): C 53.61, H 4.84, N 9.62; found: C 53.53, H 4.83, N 9.61. MS (FAB+, MeOH): *m*/*z* calculated for [(η^5^-C_5_Me_5_)Ir(Imepip)(pbpz)]^+^: 787.31, found: 787.80; *m*/*z* calculated for [(η^5^-C_5_Me_5_)Ir(pbpz)]^+^: 608.17, found: 608.41.

##### [(η^5^-C_5_Me_5_) Ir(Imepip)(pbpn)]BF_4_ (**6**)

Following the procedure to synthesize complex **3**, complex **6** was prepared from a solution of **2** (0.1305 g, 0.2 mmol, 1.0 equiv), (*N*-ethylpiperidyl)imidazole (Imepip, 0.0342 g, 0.2 mmol, 1.0 equiv), and AgBF_4_ (0.0396 g, 0.2 mmol, 1.1 equiv) in degassed DCM (15 mL). The product was obtained as a dark red solid (0.1099 g, 0.12 mmol, 63%). ^1^H RMN (400 MHz, CDCl_3_): δ 9.49 (dd, ^3^*J* = 5.5 Hz, ^4^*J* = 1.3 Hz, 1H, H^6^), 9.37 (dd, ^3^*J* = 8.0 Hz, ^4^*J* = 1.3 Hz, 1H, H^4^), 8.85 (s, 2H, H^19^ and H^20^), 8.82 (d, ^3^*J* = 8.0 Hz, 1H, H^10^), 8.24 (dd, ^3^*J* = 7.3 Hz, ^4^*J* = 1.0 Hz, 1H, H^12^), 8.21–8.11 (m, 3H, H^23^, H^24^ and H^b^), 7.88 (dd, ^3^*J* = 8.0, 5.5 Hz, 1H, H^5^), 7.78 (t, ^3^*J* = 7.6 Hz, 1H, H^11^), 7.60 (m, 2H, H^25^ and H^26^), 6.93 (t, *J* = 1.3 Hz, 1H, H^d^), 6.53 (t, *J* = 1.3 Hz, 1H, H^e^), 4.19–4.03 (m, 2H, H^f^), 2.67 (m, 2H, H^g^), 2.43 (m, 4H, H^i^), 1.73 (s, 19H, H^28^ and H^j^), 1.44 (m, 2H, H^k^). ^13^C{^1^H} RMN (101 MHz, CDCl_3_): δ 160.9 (C, C^2^), 159.3 (C, C^7^), 154.1 (CH, C^6^), 144.1 (C, C^14^), 142.9 (C, C^8^), 141.8 (C, C^13^), 140.3 (CH, C^b^), 138.9 (C, C^17^ or C^18^), 138.4 (C, C^17^ or C^18^), 136.3 (CH, C^12^), 135.4 (CH, C^4^), 134.6 (C, C^21^ or C^22^), 134.3 (C, C^21^ or C^22^), 132.0 (C, C^9^), 131.9 (CH, C^11^), 129.4 (CH, C^e^), 128.7 (CH, C^23^ or C^24^), 128.6 (CH, C^23^ or C^24^), 127.9 (CH, C^19^ or C^20^), 127.8 (CH, C^19^ or C^20^), 127.2 (CH, C^25^ or C^26^), 127.1 (C, C^3^), 125.1 (CH, C^5^), 121.1 (CH, C^d^), 120.2 (CH, C^10^), 90.1 (5C, C^27^), 57.7 (CH_2_, C^g^), 54.1 (2CH_2_, C^i^), 44.7 (CH_2_, C^f^), 25.2 (2CH_2_, C^j^), 23.5 (CH_2_, C^k^), 9.0 (5CH_3_, C^28^). Elemental Analysis calculated for C_43_H_44_BF_4_IrN_6_ (923.88 g/mol): C 55.90, H 4.80, N 9.10; found: C 55.89, H 4.79, N 9.08. MS (FAB+, MeOH): *m*/*z* calculated for [(η^5^-C_5_Me_5_)Ir(Imepip)(pbpn)]^+^: 837.33, found: 837.67; *m*/*z* calculated for [(η^5^-C_5_Me_5_)Ir(pbpn)]^+^: 658.18, found: 658.19.

##### [(η^5^-C_5_Me_5_)Ir(bzIm)(pbpz)]BF_4_ (**7**)

Following the procedure to synthesize complex **3**, complex **7** was prepared from a solution of **1** (0.1284 g, 0.2 mmol, 1.0 equiv), *N*-benzylimidazole (bzIm, 0.0313 g, 0.2 mmol, 1.0 equiv), and AgBF_4_ (0.0446 g, 0.2 mmol, 1.1 equiv) in degassed DCM (15 mL). The product was obtained as a dark yellow solid (0.1135 g, 0.13 mmol, 66.7%). ^1^H RMN (400 MHz, CDCl_3_): δ 9.62 (dd, ^3^*J* = 5.3 Hz, ^4^*J* = 1.0 Hz, 1H, H^6^), 9.47 (dd, ^3^*J* = 8.0 Hz, ^4^*J* = 1.0 Hz, 1H, H^4^), 8.92 (dd, ^3^*J* = 7.9 Hz, ^4^*J* = 0.9 Hz, 1H, H^10^), 8.38 (m, 1H, H^19^), 8.33 (m, 2H, H^20^ and H^b^), 8.23 (dd, ^3^*J* = 7.3 Hz, ^4^*J* = 0.9 Hz, 1H, H^12^), 8.00 (dd, ^3^*J* = 8.0, 5.4 Hz, 1H, H^5^), 7.90 (qd, *J* = 6.8, 2.2 Hz, 2H, H^21^ and H^22^), 7.77 (t, ^3^*J* = 7.6 Hz, 1H, H^11^), 7.18 (m, 3H, H^i^ and H^j^), 7.09 (m, 2H, H^h^), 6.49 (s, 1H, H^d^), 6.43 (s, 1H, H^e^), 5.20 (d, *J* = 14.6 Hz, 1H, H^f^), 4.91 (d, *J* = 14.6 Hz, 1H, H^f^), 1.72 (s, 15H, H^24^). ^13^C{^1^H} RMN (101 MHz, CDCl_3_): δ 160.1 (C, C^2^), 159.1 (C, C^7^), 154.3 (CH, C^6^), 142.8 (2C, C^8^ and C^14^), 141.9 (C, C^17^ and C^18^), 140.8 (C, C^13^), 140.4 (CH, C^b^), 135.9 (CH, C^12^), 135.4 (CH, C^4^), 135.1 (C, C^g^), 131.7 (C and CH, C^9^ and C^11^), 130.9 (CH, C^21^ or C^22^), 130.5 (CH, C^21^ or C^22^), 129.6 (CH, C^e^), 129.5 (CH, C^20^), 129.0 (CH, C^19^), 128.5 (2CH, C^i^ and C^j^), 128.4 (CH, C^h^), 126.4 (C, C^3^), 125.1 (CH, C^5^), 119.9 (CH, C^10^), 119.8 (CH, C^d^), 90.2 (5C, C^23^), 9.0 (5CH_3_, C^24^). Elemental Analysis calculated for C_39_H_35_BF_4_IrN_5_ (852.75 g/mol): C 54.93, H 4.14, N 8.21; found: C 54.82, H 4.13, N 8.20. MS (FAB+, MeOH): *m*/*z* calculated for [(η^5^-C_5_Me_5_)Ir(bzIm)(pbpz)]^+^: 766.25, found: 766.65; *m*/*z* calculated for [(η^5^-C_5_Me_5_)Ir(pbpz)]^+^: 608.17, found: 608.39.

##### [(η^5^-C_5_Me_5_)Ir(bzIm)(pbpn)]BF_4_ (**8**)

Following the procedure to synthesize complex **3**, complex **8** was prepared from a solution of **2** (0.1396 g, 0.2 mmol, 1.0 equiv), *N*-benzylimidazole (bzIm, 0.0290 g, 0.2 mmol, 1.0 equiv), and AgBF_4_ (0.0450 g, 0.2 mmol, 1.1 equiv) in degassed DCM (15 mL). The product was obtained as a dark red solid (0.1606 g, 0.17 mmol, 88.3%). ^1^H RMN (400 MHz, (CD_3_)_2_CO): δ 9.59 (dd, ^3^*J* = 5.5 Hz, ^4^*J* = 1.3 Hz, 1H, H^6^), 9.34 (dd, ^3^*J* = 8.0 Hz, ^4^*J* = 1.3 Hz, 1H, H^4^), 8.82 (s, 2H, H^19^ and H^20^), 8.72 (dd, ^3^*J* = 7.8 Hz, ^4^*J* = 0.9 Hz, 1H, H^10^), 8.45 (dd, ^3^*J* = 7.3 Hz, ^4^*J* = 0.9 Hz, 1H, H^12^), 8.21 (m, 2H, H^23^ and H^24^), 8.13 (s, 1H, H^b^), 7.93 (dd, ^3^*J* = 8.0, 5.5 Hz, 1H, H^5^), 7.79 (t, ^3^*J* = 7.5 Hz, 1H, H^11^), 7.59 (m, 2H, H^25^ and H^26^), 7.14 (m, 3H, H^i^ and H^j^), 7.07 (m, 3H, H^d^ and H^h^), 6.93 (s, 1H, H^e^), 5.16 (d, *J* = 2.9 Hz, 2H, H^f^), 1.81 (s, 15H, H^28^). ^13^C{^1^H} RMN (101 MHz, (CD_3_)_2_CO): δ 161.8 (C, C^2^), 160.4 (C, C^7^), 154.8 (CH, C^6^), 144.7 (C, C^14^), 143.5 (C, C^8^), 142.4 (C, C^13^), 140.8 (CH, C^b^), 139.5 (C, C^17^ or C^18^), 138.9 (C, C^17^ or C^18^), 137.6 (CH, C^12^), 137.0 (C, C^g^), 136.0 (CH, C^4^), 135.4 (C, C^21^ or C^22^), 135.1 (C, C^21^ or C^22^), 132.8 (CH, C^11^), 132.6 (C, C^9^), 130.7 (CH, C^e^), 129.6 (CH, C^i^ or C^j^), 129.4 (CH, C^23^ or C^24^), 129.3 (CH, C^23^ or C^24^), 129.1 (CH, C^i^ or C^j^), 128.52 (CH, C^19^ or C^20^), 128.46 (CH, C^h^), 128.39 (CH, C^19^ or C^20^), 128.1 (CH, C^25^ or C^26^), 128.0 (CH, C^25^ or C^26^), 127.5 (C, C^3^), 125.6 (CH, C^5^), 122.4 (CH, C^d^), 120.6 (CH, C^10^), 91.0 (5C, C^27^), 52.0 (CH_2_, C^f^), 9.0 (5CH_3_, C^28^). Elemental Analysis calculated for C_43_H_37_BF_4_IrN_5_ (902.81 g/mol): C 55.21, H 4.13, N 7.76; found: C 55.12, H 4.13, N 7.75. MS (FAB+, MeOH): *m*/*z* calculated for [(η^5^-C_5_Me_5_)Ir(bzIm)(pbpn)]^+^: 816.27, found: 816.65; *m*/*z* calculated for [(η^5^-C_5_Me_5_)Ir(pbpn)]^+^: 658.18, found: 658.18.

##### [(η^5^-C_5_Me_5_)IrCl(pbpq)] (**9**)

**9** was synthesized using a procedure adapted from the literature.^68^ In a 50 mL Schlenk flask, a solution of [(η^5^-C_5_Me_5_)Ir(μ-Cl)Cl]_2_ (0.0885 g, 0.11 mmol, 1.0 equiv), benzo[*f*]pyrido[2,3-*h*]quinoxaline (H-pbpq, 0.0512 g, 0.22 mmol, 2.0 equiv), Na_2_CO_3_ (0.0261 g, 0.25 mmol, 2.3 equiv), and AgOCOCF_3_ (0.0496 g, 0.22 mmol, 2.0 equiv) in degassed DCM (25 mL) was stirred at ambient temperature for 30 h. The resulting precipitate (AgCl) was removed by filtration over Celite. To recover all of the product, the residue was washed with degassed DCM until the filtrate was colorless. The filtrate was concentrated in vacuo until dryness. Then, the red solid was purified by neutral alumina chromatography with EtOAc/hexane (1:1) to yield a yellow solid (0.0439 g, 0.07 mmol, 33%). ^1^H RMN (500 MHz, CDCl_3_): δ 9.27 (dd, ^3^*J* = 7.9 Hz, ^4^*J* = 1.2 Hz, 1H, H^4^), 9.06 (dd, ^3^*J* = 5.3 Hz, ^4^*J* = 1.2 Hz, 1H, H^6^), 8.96 (d, ^3^*J* = 2.0 Hz, 1H, H^17^), 8.87 (d, ^3^*J* = 2.1 Hz, 1H, H^18^), 8.64 (d, ^3^*J* = 7.9 Hz, 1H, H^10^), 8.20 (d, ^3^*J* = 7.3 Hz, 1H, H^12^), 7.73 (t, ^3^*J* = 7.6 Hz, 1H, H^11^), 7.60 (dd, ^3^*J* = 7.9, 5.5 Hz, 1H, H^5^), 1.77 (s, 15H, H^20^). ^13^C{^1^H} RMN (126 MHz, CDCl_3_): δ 161.0 (C, C^7^), 159.4 (C, C^2^), 151.6 (CH, C^6^), 144.5 (CH, C^17^), 143.7 (C, C^13^ and C^14^), 143.0 (CH, C^18^), 141.9 (C, C^8^), 140.0 (C, C^13^), 135.7 (CH, C^12^), 133.8 (CH, C^4^), 131.5 (CH, C^11^), 122.4 (CH, C^5^), 117.8 (CH, C^10^), 88.7 (5C, C^19^), 9.2 (5CH_3_, C^20^). Elemental Analysis calculated for C_25_H_23_ClIrN_3_·(CH_2_Cl_2_)_0.6_ (644.10 g/mol): C 47.74, H 3.79, N 6.52; found: C 47.43, H 3.48, N 6.17. MS (ESI+, DCM): *m*/*z* calculated for [(η^5^-C_5_Me_5_)_2_Ir_2_Cl(pbpq)_2_+2H]^+^: 1151.29, found: 1151.28; *m*/*z* calculated for [(η^5^-C_5_Me_5_)Ir(pbpq)]^+^: 558.15, found: 558.15.

##### [(η^5^-C_5_Me_5_)Ir(bhq)Cl] (**10**)

**10** was synthesized using a procedure adapted from the literature.^68^ In a 50 mL Schlenk flask, a solution of [(η^5^-C_5_Me_5_)Ir(μ-Cl)Cl]_2_ (0.1711 g, 0.21 mmol, 1.0 equiv), benzo[*h*]quinoline (H-bhq, 0.0775 g, 0.43 mmol, 2.0 equiv), Na_2_CO_3_ (0.0492 g, 0.46 mmol, 2.0 equiv), and AgOCOCF_3_ (0.0993 g, 0.45 mmol, 2.0 equiv) in degassed DCM (25 mL) was stirred at ambient temperature for 4.5 h. The resulting precipitate (AgCl) was removed by filtration over Celite. To recover all of the product, the residue was washed with degassed DCM until the filtrate was colorless. The filtrate was concentrated in vacuo until dryness. Then, the yellow solid was purified by flash chromatography with Celite as stationary phase and DCM as mobile phase. The filtrate was concentrated in vacuo until dryness to give a yellow solid (0.2197 g, 0.41 mmol, 94%). The NMR data are given because the assignment was not included in the manuscript where the preparation was reported.^68^^1^H RMN (500 MHz, CDCl_3_): δ 8.96 (dd, ^3^*J* = 5.3 Hz, ^4^*J* = 1.3 Hz, 1H, H^6^), 8.14 (dd, ^3^*J* = 8.0 Hz, ^4^*J* = 1.3 Hz, 1H, H^4^), 8.06 (d, ^3^*J* = 7.0 Hz, 1H, H^10^), 7.82 (d, ^3^*J* = 8.7 Hz, 1H, H^12^), 7.62 (dd, ^3^*J* = 7.8, 7.2 Hz, 1H, H^11^), 7.56 (m, 2H, H^13^ and H^14^), 7.47 (dd, ^3^*J* = 7.9, 5.3 Hz, 1H, H^5^), 1.74 (s, 15H, H^16^). ^13^C{^1^H} RMN (126 MHz, CDCl_3_): δ 160.9 (C, C^7^), 157.4 (C, C^2^), 149.0 (CH, C^6^), 141.9 (C, C^8^), 135.7 (CH, C^4^), 134.0 (C, C^9^), 132.5 (CH, C^10^), 130.7 (CH, C^11^), 130.0 (CH, C^12^), 127.1 (C, C^3^), 123.1 (CH, C^13^ or C^14^), 121.5 (CH, C^5^), 120.1 (CH, C^13^ or C^14^), 88.4 (5C, C^15^), 9.1 (5CH_3_, C^16^). Elemental Analysis calculated for C_23_H_23_ClIrN·(CH_2_Cl_2_)_0.4_ (575.07 g/mol): C 48.87, H 4.17, N 2.44; found: C 48.71, H 3.74, N 4.20. Mass data were not previously given.^68^ MS (ESI+, DCM): *m*/*z* calculated for [(η^5^-C_5_Me_5_)_2_Ir_2_(bhq)_2_+H]^+^: 1011.30, found: 1011.30; *m*/*z* calculated for [(η^5^-C_5_Me_5_)Ir(bhq)]^+^: 506.15, found: 506.15.

### Methods and Instrumentation

#### Stability and Photostability Studies in DMSO-*d*_6_ and DMSO-*d*_6_/D_2_O (NMR Spectroscopy)

Solutions of complexes **1** and **6** at 3.2 mM and **2** at 0.6 mM in DMSO-*d*_6_ and DMSO-*d*_6_/D_2_O mixture (9:1, v/v) were prepared. The lower concentration of **2** was due to the lower solubility of the complex. ^1^H NMR spectra were recorded at different times until 48 h in dark conditions and under blue (470 nm), green (530 nm), and red (655 nm) light irradiation to evaluate the stability of the complexes.

#### Reactivity of **1w** with *n*-Bu_4_NCl

A solution of **1w** (3.0 mM) with 100 equiv of *n*-Bu_4_NCl (300 mM) in a DMSO-*d*_6_/D_2_O (9:1, v/v) mixture was monitored by NMR spectroscopy at room temperature and at 60 °C. The presence of DMSO ensured the solubility of the complexes. ^1^H NMR spectra were recorded in dark conditions at ambient temperature over 4 days or at 60 °C for 15 min.

#### Photostability Studies in DMEM

The stability of the complexes in biological media was studied by UV–vis spectroscopy. Complexes **1** and **2** were dissolved in DMSO and complexes **3**–**8** were dissolved in MeOH at 1.0 × 10^–3^ M and then diluted to 1.0 × 10^–5^ M with Dulbecco’s modified Eagle’s medium (DMEM) without phenol red (Corning) (the final concentration of DMSO or MeOH was 1%). The solutions were studied in the dark and under blue light irradiation (470 nm).

#### Photostability Studies in Water

The stability of complex **6** in aqueous solution was studied by UV–vis spectroscopy. Complex **6** was dissolved in MeOH at 1.0 × 10^–3^ M and then diluted to 1.0 × 10^–5^ M with water (the final concentration of MeOH was 1%). The solution was studied in the dark and under blue light irradiation (470 nm).

#### X-ray Crystallographic Structure Determination

Data collection and refinement parameters for **1**, **6** × **0**.**75C**_**2**_**H**_**6**_**O**, and **9** × **0**.**5C**_**4**_**H**_**10**_**O** are given in Table S3. Single crystals were transferred to a Bruker APEX II CCD-based diffractometer equipped with a graphite-monochromated Mo Kα radiation source (λ = 0.71073 Å). The data sets were integrated with Saint^126^ and corrected for Lorentzian and polarization effects. A semiempirical absorption correction was applied to the diffraction data.^127,128^ The software package Wingx^129,130^ was used for space group determination, structure solution, and refinement by full-matrix least-squares methods based on *F*^2^. A successful solution by direct methods provided most non-hydrogen atoms from the E map. The remaining non-hydrogen atoms were located in an alternating series of least-squares cycles and difference Fourier maps. The non-hydrogen atoms were refined with anisotropic displacement coefficients and hydrogen atoms were placed by using a riding model and included in the refinement at calculated positions.

Deposition numbers in the Cambridge database are 2223319 for **1**, 2223321 for **6**, and 2262621 for **9**.

##### 1

Exceptions and special features: For compound , several plate and block crystals were chosen, and all were found to be twinned. The selected crystals were indexed as a two-component non-merohedral twin related by a rotation of 180°.^131^ The reflections from the two domains were simultaneously integrated and Twinabs^128^ was used for scaling, empirical absorption corrections, and the generation of two different data files, one with detwinned data for structure solution and a second one for structure refinement against total integrated intensities. Compounds **6** and **9** show disorder atoms (BF_4_^–^ counteranion for compound **6** and ethyl ether for **9**) so it was necessary to apply several restraints (DELU, SIMU, and DFIX) in order to improve refinement stability.

Suitable single crystals for the X-ray diffraction study were obtained in the following way. **1**: by slow diffusion of an acetone/pentane mixture into a solution of **1** in dimethylformamide. **6**: by slow diffusion of pentane into a solution of **6** in acetone. **9**: slow diffusion of diethyl ether into a chloroform solution of **9** with pentane in the interface. The complexes crystallize in the space group *P*1̅ of the triclinic system (**1**), in the *P*2_1_/*n* of the monoclinic system (**6**), and in the *P*1̅ of the triclinic system (**9**).

#### ^1^O_2_ Quantum Yield Determination

Singlet oxygen (^1^O_2_) generation was studied using two methods.

##### (a) DPBF

It was performed for complexes **1**–**8** in acetonitrile according to a relative procedure adapted from the literature^132−134^ based on monitoring the oxidation of 1,3-diphenylisobenzofuran (DPBF, yellow) to 1,2-dibenzoylbenzene (colorless) photosensitized by the Ir(III) complexes. DPBF was used as an ^1^O_2_ scavenger due to its fast reaction with this molecule. Fresh stock air-equilibrated solutions of photosensitizers (6.00 × 10^–6^ M) and DPBF (1.50 × 10^–4^ M) in acetonitrile were prepared. In a quartz cell, 1 mL of DPBF solution, 0.5 mL of complex solution, and 1.5 mL of acetonitrile were mixed to prepare a final solution with 5.00 × 10^–5^ M DPBF and 1.00 × 10^–6^ M complex. The final solutions were studied by recording UV–vis absorption spectra at room temperature in dark conditions (over a period of 6 min with time intervals of 2 min), under blue light irradiation (λ_exc_ = 470 nm, “Medusa” photoreactor) and under green light irradiation (λ_exc_ = 530 nm, “Medusa” photoreactor) for 5 s irradiation intervals during a total exposure period of 20 s. Absorption UV–vis spectra were recorded after every irradiation interval.

The consumption of DPBF probe was measured by monitoring its absorbance intensity decrease at 410 nm with irradiation time. ^1^O_2_ generation quantum yield (ϕ_Δ_) was calculated from eq 1 using Rose Bengal or [Ru(bpy)_3_]^2+^ as reference for complexes with pbpz and pbpn ligands, respectively:
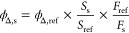
1where *s* and ref subscripts correspond to the complex and the reference, ϕ_Δ_ is the ^1^O_2_ generation quantum yield (0.53 for Rose Bengal^135^ and 0.56 for [Ru(bpy)_3_]Cl_2_^132^ in degassed acetonitrile), *S* is the slope of the plots of the −ln(normalized absorbance) of DPBF at 410 nm vs the irradiation time, and *F* is the correction factor of absorption, which is given by *F* = 1–10^–O.D.^ (where O.D. is the optical density at 470 nm of solutions of complexes and references). The reproducibility of the results was confirmed by performing each experiment three times.

The stability of DPBF probe was studied in dark conditions and under light irradiation. In dark conditions and under green light irradiation the probe remains unaltered. However, under blue light irradiation, a photobleaching of DPBF was observed. Probe consumption was approximately 6.5% every 5 s. Therefore, a working interval of 20 s was chosen, where 75% of the probe remains stable. To calculate the ϕ_Δ_, a correction factor was applied to the absorbance at 410 nm: a 6.5% of the previous value was added to each absorbance to counteract self-consumption of the probe (i.e., to the absorbance at 10 s, a 6.5% of the absorbance at 5 s was added).

Also, a degassed solution of complex **6** and DPBF was studied to evaluate the dependence of photosensitizers on oxygen. In addition, a solution of complex **6** was studied with sodium azide as ^1^O_2_ scavenger. For this experiment, 1 mL of DPBF solution, 0.5 mL of complex solution, 10 μL of NaN_3_ at 1.80 × 10^–2^ M in H_2_O, and 1.5 mL of acetonitrile were mixed to prepare a final solution with 5.00 × 10^–5^ M DPBF, 1.00 × 10^–6^ M complex, and 6.00 × 10^–5^ M NaN_3_.

##### (b). ABDA

Fresh stock air-equilibrated solutions of photosensitizers **1**–**2** and **7**–**8** (1.00 × 10^–3^ M) and ABDA (6.00 × 10^–4^ M) in H_2_O/DMSO (95:5) were prepared. In a quartz cell, 0.50 mL of ABDA solution, 0.03 mL of complex solution, and 2.47 mL of H_2_O/DMSO (95:5) were mixed to prepare a final solution with 1.00 × 10^–4^ M ABDA and 1.00 × 10^–5^ M complex. The final solutions were studied by recording UV–vis absorption spectra at room temperature under blue light irradiation (λ_exc_ = 470 nm, “Medusa” photoreactor) for 30 s irradiation intervals during a total exposure period of 5 min. Absorption UV–vis spectra were recorded after every irradiation interval.

The consumption of ABDA probe was measured by monitoring its absorbance intensity decrease at 402 nm with irradiation time. ^1^O_2_ generation quantum yield (ϕ_Δ_) was calculated from eq 1 using Rose Bengal or [Ru(bpy)_3_]^2+^ as reference. In this case, the ϕ_Δ_ values of the references in H_2_O/DMSO (95:5) are 0.76 for Rose Bengal^73^ and 0.18 for [Ru(bpy)_3_]Cl_2_.^136^ The reproducibility of the results was confirmed by performing each experiment three times.

The stability of ABDA probe was studied in dark conditions and under light irradiation. In dark conditions and under light irradiation, the probe remains unaltered.

#### Photophysical Properties

UV–vis absorption spectra were recorded on a Secomam Uvikon XS spectrophotometer using the LabPower Junior program. Quartz cuvettes with 1 or 0.1 cm optical path length were used for the measurements. Photoluminescence excitation and emission spectra were recorded on a PTI Quanta Master TM spectrofluorometer from Photon Technology International (PTI) equipped with a Xenon short arc lamp (75 W) and an 814PTM detector. Hellma quartz cuvettes with 1 cm optical path length were used. Felix32 software was used to collect and process fluorescence data.

For all optical measurements in degassed solvents, the compounds were dissolved at 1.00 × 10^–5^ M in a glovebox under a nitrogen atmosphere and the solutions were kept under inert atmosphere in 1 cm closed cuvettes equipped with Teflon septum screw caps. All optical measurements were recorded at room temperature. The luminescence emission spectra were recorded from 450 to 800 nm at a rate of 1 nm/s with an optical length of 5 mm by exciting at 420 nm with a xenon arc lamp.

#### Transient Absorption Spectroscopy (TAS)

Transient absorption spectra were measured using a setup composed of a LKS 60 ns laser photolysis spectrometer from Applied Photophysics, with a Brilliant Q-Switch Nd:YAG laser from Quantel, using the third harmonics (λ_exc_ = 355 nm, laser pulse half-width equal to 4 ns). Measurements were repeated in degassed and aerated samples. Absorbance was adjusted to 0.2.

#### Photoactivation Protocol in Nonbiological Studies

Photoactivation in nonbiological studies was carried out in a 16-compartment “Medusa” photomultireactor built by Microbeam, equipped with 2.3 W light-emitting diodes (LED) (Luxeon Rebel, Philips Lumileds) situated below each compartment (Figure S117). The LEDS were driven at 700 mA. Every reactor position is composed of a 40 mL cylindrical glass flask provided by Scharlau. All reaction positions were irradiated from the bottom using monochromatic light provided by LEDs at different wavelengths (470, 530, 655 nm, radiant power: 910 mW). The spectral width at half of the peak intensity was 20 nm for the LED at 470 nm, 30 nm for the LED at 530, and 20 nm for the LED at 655 nm. All reactions in the system could be stirred using an orbital stirrer, which was set at 50 rpm.

#### TD-DFT Calculations

The ground state and excited state calculations were performed using the DFT method with B3LYP functional^137,138^ as well as the time-dependent (TD)-DFT^139−141^ method using Gaussian 09 software,^142^ respectively. D3 Grimme dispersion^143^ was added including “ultrafine integration grid” to obtain a better description. The 6-31G(d,p) basis set was used for C, N, and H atoms and the SDD effective core potential basis set for Ir atom. Solvent effects were considered within the self-consistent reaction field (SCRF) theory using the solvation model SMD.^144^ The geometries of the lowest-lying triplet states were calculated at the spin-unrestricted UB3LYP level with a spin multiplicity of 3. Chemissian software 4.43v was used to generate fragmental contributions and energies for each molecular orbital as well as the electronic structure for each electronic state.^145^

#### p*K*_a_ Determination of **5**, **6**, and Imepip

Solutions of complexes **5** and **6** (2.00 × 10^–5^ M) and Imepip ligand (2.00 × 10^–3^ M) in H_2_O/DMSO (98/2, v/v) mixture with *n*-Bu_4_NBF_4_ (5 mM) were prepared. The presence of the salt ensured the conductivity of the media. Evolution of the peaks was studied at different pH by recording the absorbance spectrum. Aliquots of 10 μL of HCl (0.002–0.020 M) or NaOH (0.002–0.020 M) were added to a solution of 2.4 mL of complex to vary the pH. The absorbance versus pH plot enables us to determine one constant according to the equation from the Wilson and Lester method (eq 2):^146^
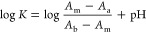
2where *A*_a_ is the absorbance for the acid species, *A*_b_ is the absorbance for the basic species, and *A*_m_ is the absorbance of the mixture. The intersection obtained by representing log(*A*_m_ – *A*_a_)/(*A*_b_ – *A*_m_) versus pH provides the p*K*_a_.

#### Lipophilicity Determination by the “Shake Flash” Method

The partition coefficients of complexes **5**, **6**, **7**, and **8** were determined by the standard “shake flask” method.^37,84,85^ Octanol-saturated water (OSW) and water-saturated octanol (WSO) were prepared using analytical grade 1-octanol and ultrapure water. The complexes were dissolved in WSO and molar extinction coefficient was determined. Then, 2 mL of 25 μg/mL complex in WSO was shaken with 2 mL of OSW at 1500 rpm for 6 h at room temperature. To separate the phases, centrifugation was done at 4000 rpm for 10 min. The absorbance of WSO phase before and after shaking was measured. The absorbance of WSO before mixing minus the absorbance of WSO after mixing corresponds to the absorbance of the complex in OSW. The distribution coefficients (log *D*_o/w_) of the complexes were obtained from the ratio of the complexes present in 1-octanol and the aqueous phase (eq 3). Each experiment was repeated three times. To avoid protonation in complexes **5** and **6**, a carbonate–bicarbonate buffer at pH 9.2 in OSW was used.
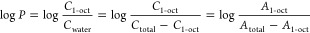
3where *A*_1-oct_ corresponds to the absorbance of WSO phase and *A*_total_ is the actual concentration of the WSO before shaking.

#### Cell Lines

The human A549 lung cancer, HeLa cervical cancer, PC-3 prostate adenocarcinoma, and MRC-5 lung fibroblasts cell lines were purchased from the American Type Culture Collection (ATCC). The 1BR.3.G skin fibroblasts cell line was obtained from the European Collection of Authenticated Cell Cultures (ECACC). All cell lines were cultured in Dulbecco’s modified Eagle’s medium (DMEM) (Corning) supplemented with 10% fetal bovine serum (FBS) (Gibco-BRL), 1% l-glutamine (Corning), and 1% penicillin-streptomycin (Corning) at 37 °C in a humidified atmosphere containing 5% CO_2_. The cells were maintained by successive trypsinization and seeding. Possible contamination with mycoplasma was routinely checked using VenorH GeM Mycoplasma Detection Kit (Minerva Biolabs).

#### Cell Viability Experiments

Cells were cultured in 96-well plates at a density of 3000 (A549), 2500 (HeLa), or 5000 (PC-3, 1BR.3.G, and MRC-5) cells per well in cell culture medium and allowed to attach for 24 h. Solutions of the complexes at different concentrations were prepared for cell treatments. In all cases, the solid compounds were weighed using an analytical balance (Mettler Toledo AX205DR) to ensure precise measurements. Subsequently, 5 mM solutions were prepared using DMSO. Solutions were then diluted in water to reach a concentration of 1 mM and then, aliquots were taken and diluted in culture medium to obtain solutions ranging from 0 to 50 μM. The final concentration of DMSO in the solutions did not exceed 1%. The cells were treated with these solutions in dark conditions or following the photoactivation protocol (see below). 48 h later the treatments were removed, and cells were washed with phosphate-buffered saline (PBS).

Initially, the effect of the complexes on the cell viability was determined by the 3-(4,5-dimethylthiazol-2-yl)-2,5-diphenyltetrazolium bromide (MTT) assay.^147^ The cells were incubated for 2 h with 100 μL of culture medium together with 10 μL of MTT (Sigma-Aldrich) at 0.5 mg/mL. After discarding the medium, DMSO was added to each well to dissolve the purple formazan crystals. The absorbance of each well was determined on a Multiscan Plate Reader (Synergy 4, Biotek, Winooski) at a wavelength of 570 nm. Cell viability was also assessed using the CyQUANT Direct Cell Proliferation Assay (Thermo Fisher Scientific), following the manufacturer’s instructions. Briefly, after the treatments, the cells were washed with PBS, and an equal volume of culture medium and CyQUANT mix was added to each well. The plates were then incubated for 1 h at 37 °C. Subsequently, the fluorescence intensity was bottom read using a Multiscan Plate Reader (Synergy 4, Biotek, Winooski) with excitation and emission filters of 485 and 528 nm, respectively. In both assays, at least three replicates were measured for each treatment. The concentration that reduces the cell viability by 50% (IC_50_) was established using the Gen5 software (BioTek). Three independent experiments were carried out for each compound. Compounds with IC_50_ values greater than 50 μM were considered to be inactive. The phototoxicity index (PI = IC_50,dark_/IC_50,light_) was determined.

To assess the cytotoxicity of the complexes, A549 cells were seeded in 96-well plates and treated with the complexes at the respective IC_50,dark_ value. After 24 h of incubation, 10 μL of Trypan Blue (0.4% solution; Sigma) was added directly to the wells to avoid loss of dead cells in the treatment washes. After 10 min, images of the cells were captured using an Olympus CKX41 Microscope equipped with LCmicro software (Olympus).

#### Photoactivation Protocol

To assess the photodynamic activity of the complexes, cells were incubated with the complexes for 4 h to enable their intracellular accumulation. The cells were then irradiated with light with a wavelength of 460 nm (blue), 530 nm (green), or 655 nm (red) for 1 h using a LED system (LuxLight) with an effective power of 6.7 mW cm^–2^, which provides a total light dose of 24.1 J cm^–2^. The LED system was positioned at 25 mm from the cell culture plates.

#### Cytotoxic Activity against Spheroids

For the evaluation of the cytotoxic activity of complex **6** in 3D cultures, 96-well plates coated with a thin solidified layer of Geltrex reduced growth factor basement membrane matrix (Gibco) were used to form A549 spheroids. A density of 1500 single cells per well was seeded in culture medium supplemented with 2% Geltrex. Treatments with concentrations of complex **6** ranging from 5 to 0.001 μM in culture medium containing 2% Geltrex were performed at day 6. The cells were then kept in the dark or photoactivated with blue light, as described. After 48 h, the treatments were removed, the cells were washed with PBS and cell viability was determined by adding 100 μL of medium and 100 μL of CellTiter-Glo 3D reagent (Promega) to each well. Following the manufacturer’s protocol, the cells were kept in agitation for 5 min and incubated for 25 min at room temperature. Then, the luminescence was determined using a Multiscan Plate Reader (Synergy 4, Biotek, Winooski). The IC_50_ values were calculated with the Gen5 Data Analysis Software (BioTeck) for two independent experiments, each with duplicate samples. Nontreated cells were used as control.

#### Hemolytic Activity

The hemolytic activities of the complexes were determined by measuring hemoglobin release from porcine red blood cells (RBC). First, fresh porcine blood commercially obtained was diluted to 5% v/v in PBS solution and washed three times with PBS by centrifugation at 1000 rcf for 10 min. Then, RBC suspension (150 μL) was mixed with 150 μL of each complex at concentrations ranging from 1 to 50 μM. A solution of 0.2% Triton X-100 in PBS was used as positive control to induce complete hemolysis. PBS alone was used as negative control. Samples were incubated at 37 °C for 1 h in an orbital shaker (50 rpm) and then centrifuged at 3500 rcf for 10 min. 80 μL aliquots of the supernatant were transferred to a 96-well plate and diluted with 80 μL of Milli-Q water. Hemolysis was evaluated by measuring the absorbance of the samples at 540 nm with a Synergy 4 plate reader (BioTek, Winooski). The percentage of hemolysis (*H*) was calculated using eq 4:
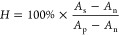
4where *A*_s_ is the absorbance for a given sample, *A*_n_ is the absorbance for the negative control, and *A*_p_ is the absorbance for the positive control. Each sample was tested in triplicate.

#### Intracellular ROS Production

A549 cells were seeded onto 24-well plates (5 × 10^4^ cells/well) 24 h before the experiment. The cells were incubated for 4 h with complexes **2**, **4**, **6**, and **8** at 10 nM and then photoactivated or kept in the dark for an additional hour. In addition, the cells were treated for 5 h with the complexes at the corresponding IC_50,dark_ without irradiation. The cells exposed for 30 min to 0.5 μM FeCl_2_ and 0.04% H_2_O_2_, as ROS inducers, were used as positive control. After the treatments, the cells were washed with PBS and harvested by trypsinization. Intracellular ROS were detected by incubation with the chloromethyl 2′,7′-dichlorodihydrofluorescein diacetate (CM-H_2_DCFDA; Invitrogen) probe at 2.5 μM for 30 min at 37 °C. After washing with PBS, the median fluorescence of 1 × 10^4^ cells was measured with a NovoCyte flow cytometer (Agilent Technologies) equipped with the NovoExpress software. The fold increase versus control untreated cells was determined for each treatment in three independent experiments.

The types of ROS generated by the treatments were also investigated. Superoxide anion (O_2_^•–^) production was monitored using the ROS-ID Superoxide Detection Kit (Enzo Life Sciences), which contains a cell-permeable probe that selectively emits orange fluorescence upon reacting with O_2_^•–^. Following the treatments, cells were harvested and incubated with the probe for 30 min at 37 °C. Fluorescence emission from 1 × 10^4^ cells was measured by flow cytometry (Ex/Em: 550/620 nm) and compared to that of control untreated cells. In addition, hydrogen peroxide (H_2_O_2_) generation was measured using the Hydrogen Peroxide Assay Kit (Cell-based) (Abcam). Treated cells were collected and exposed to the AbGreen Indicator, a cell-permeable probe that exhibits green fluorescence upon interaction with H_2_O_2_. After 1 h of incubation at 37 °C, the median fluorescence of 1 × 10^4^ cells was determined by flow cytometry (Ex/Em = 490/520 nm) and compared to that of control cells. All experiments were conducted in triplicate.

To assess the impact of ROS on cell death caused by the complexes, A549 cells were incubated with complexes **2**, **4**, **6** and **8** at their respective IC_50,light_ for 4 h, followed by irradiation with blue light (460 nm) for 1 h in the absence or presence of selective ROS scavengers: sodium azide (Sigma-Aldrich) for ^1^O_2_ or tiron (4,5-dihydroxy-1,3-benzenedisulfonic acid) for O_2_^•–^, both at a final concentration of 5 mM. After 48 h of treatment, cell viability was measured by MTT assays. The percentage of viable cells was established in comparison to untreated cells exposed to the corresponding scavengers or to medium alone. Each condition was tested by triplicate, in three independent experiments.

#### Cell Internalization Experiments

To assess the cell internalization mechanism, 8 × 10^6^ A549 cells were incubated with complex **6** at 10 μM for 1 h in the dark under different conditions: (i) at 37 °C (positive control cells), (ii) at 37 °C in the presence of CCCP (50 μM) (to inhibit mitochondrial ATP production) or (iii) at 4 °C (to inhibit cellular metabolism). The cells incubated with medium alone were included as negative control. Then, the cells were washed twice with PBS, detached with trypsin, and centrifuged to obtain the whole cell pellet. By trypan blue staining, it was verified that in all samples, the cells displayed more than 95% viability. The iridium amount in the samples was then determined by inductively coupled plasma mass spectrometry (ICP-MS) analysis. To this end, the cell pellets were dissolved in 400 μL of 69% v/v nitric acid (PanReac Applichem) and heated in a water bath at 100 °C for 18 h. Samples were then allowed to cool and diluted with Milli-Q water to a final volume of 10 mL. To analyze the amount of iridium, cell pellets were dissolved in 400 μL of 69% v/v concentrated nitric acid and heated at 100 °C for 18 h. The vials were allowed to cool, and then the digested samples were diluted 10-fold with Milli-Q. Iridium content was measured on an ICP-MS Agilent 7500c instrument at the Serveis Tècnics de Recerca, Universitat de Girona. The solvent used for all ICP-MS experiments was Milli-Q water with 2% superpure grade HNO_3_. The iridium standard (high purity standards, 1000 μg/mL ± 2 μg/mL in 5% hydrochloric acid in low TOC water (<50 ppb)) was diluted with 2% HNO_3_ to 400 ppb. Iridium standards were freshly prepared in Milli-Q water with 2% HNO_3_ before each experiment. The concentrations used for the calibration curve were 0, 1, 2, 5, 10, and 20 ppb. The isotope detected was 193Ir and readings were done in triplicate. Rhodium was added as an internal standard at a concentration of 10 ppb to all samples. All of the experiments were performed by duplicate.

#### Subcellular Distribution

The intracellular distribution of complex **6** was explored by measuring the iridium content in different cellular compartments: nucleus, membrane/particulate (which includes cellular organelles and organelle’s membrane proteins), cytosol, and cytoskeleton. To this end, 8 × 10^6^ A549 cells were incubated with **6** at 10 μM for 4 h in the dark at 37 °C. Then, the cells were washed twice with PBS, detached with trypsin, and centrifuged to obtain the whole cell pellet. The viability of the cells was assessed through Trypan blue staining, which confirmed that over 95% of the cells were viable. The different cell fractions were then isolated using the FractionPREP Cell Fractionation kit (BioVision) and the total iridium content in each fraction was measured by ICP-MS in two independent experiments, as described above.

##### Evaluation of Mitochondrial Membrane Potential

A549 cells were seeded onto 24-well plates. 24 h later, cells were incubated for 4 h with complexes **2**, **4**, **6**, and **8** at 10 nM and then photoactivated or kept in the dark for an additional hour. Next, the cells were washed with PBS and harvested by trypsinization. Mitochondrial membrane potential changes were immediately measured by the JC-1 (5,5′,6,6′-tetrachloro-1,1′,3,3′-tetraethylbenzimidazolocarbo-cyanine iodide) Mitochondrial Membrane Potential Detection Kit (Biotium), according to the manufacturer’s instructions. As positive control, the cells were coincubated with CCCP (Sigma) at 5 μM for 30 min to disrupt the mitochondrial membrane potential. For each treatment, the fluorescence of 1 × 10^4^ cells was analyzed by a Novocyte flow cytometer. JC-1 red aggregates were detected at a wavelength of 590 nm (FL2) and JC-1 green monomers at 529 nm (FL1).

#### NADH Oxidation

##### UV–Vis Spectroscopy

Fresh stock solutions of the complexes at 7.50 μM in H_2_O/MeOH mixture (97.5/2.5, v/v) and NADH at 300 μM in H_2_O/MeOH mixture (97.5/2.5, v/v) were prepared. In a quartz cell, 1 mL of complex solution, 1 mL of NADH solution, and 1 mL of solvent mixture were mixed to prepare a final solution with 2.50 μM complex and 100 μM NADH (complex/NADH rate = 1/40). The solutions were studied by recording UV–vis absorption spectra in dark conditions and under blue light irradiation (λ_exc_ = 470 nm, “Medusa” photoreactor) at ambient temperature and various time intervals for 8 h. In addition, a solution of NADH at 100 μM was studied under blue light irradiation to confirm its photostability. The photocatalytic rate of the photosensitizers was evaluated by monitoring its absorbance decrease at 340 nm, which correspond to NADH oxidation.

The turnover numbers (TONs) of complexes were calculated by measuring the absorption difference at 340 nm after 1 h of reaction. TON was calculated from the difference in NADH concentration after 1 h divided by the concentration of photocatalyst (eq 5), where b is the optical path length (1 cm). The concentration of NADH was obtained using the extinction coefficient ε_340_ = 5751 M^–1^ cm^–1^.^40,148^
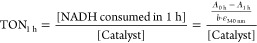
5

#### NADH Oxidation

##### NMR Spectroscopy

Solutions of complexes **1**, **2**, **5**, and **6** (1.0 mM) and NADH (3.5 mM) (complex/NADH rate = 1/3.5) in a DMSO-*d*_6_/D_2_O mixture (9:1, v/v) for complexes **1** and **2** and a DMSO-*d*_6_/D_2_O mixture (1/1, v/v) for complexes **5** and **6** were studied by ^1^H NMR at ambient temperature in dark conditions and under blue light irradiation (λ_exc_ = 470 nm, “Medusa” photoreactor). ^1^H NMR spectra were recorded at ambient temperature at different time intervals until 22 h. The photooxidation of NADH was deduced by the decrease of the 8.48 ppm resonance of NADH and the increase of the 8.43 ppm signal of NAD^+^ in DMSO-*d*_6_/D_2_O (9:1, v/v) and the decrease of the 8.55 ppm resonance of NADH and the increase of the 8.51 ppm signal of NAD^+^ in DMSO-*d*_6_/D_2_O (1/1, v/v).

#### Confocal Microscopy Experiments

For confocal microscopy experiments, A549 cells were seeded onto glass-bottom 8-well chamber slides (Ibidi) (5 × 10^4^ cells per well) and allowed to attach overnight. In each experiment, the cells were treated with the complexes at the indicated conditions and washed with PBS before staining. For mitochondria visualization, the cells were incubated with the fluorescent dye MitoTracker Red CMXRos (Molecular Probes) at 200 nM in serum-free culture medium for 30 min at 37 °C. Cell nuclei were counterstained in blue with Hoechst 33342 (Invitrogen) diluted 1:4000 in DMEM without phenol red. To analyze lysosomal damage, the cells were incubated with AO at 5 μM at 37 °C for 15 min. Before observation, the cells were washed twice with PBS and fresh growth medium without phenol red was added. Cell images were immediately obtained by a Nikon A1R confocal microscope and images were analyzed using the NIS-Elements AR (Nikon, Japan) and the Fiji/ImageJ software.

#### EtBr Displacement Assay

To investigate the capacity of the complexes to intercalate into DNA, the competitive ethidium displacement assay was carried out as previously described^149^ with minor modifications. Stock solutions of 20 μM calf thymus DNA (CT-DNA) and 25 μM Ethidium bromide (EtBr) were prepared in saline TE (50 mM NaCl, 10 mM tris(hydroxymethyl) aminomethane–HCl, 0.1 mM ethylenediaminetetracetic acid (EDTA), pH 7.4). Serial dilutions of metal complexes were prepared at concentrations ranging from 20 to 400 μM from a 1 mM stock solution containing 20% DMSO. The assay was carried out by mixing 25 μL of CT-DNA and 25 μL of EtBr with 50 μL of each compound dilution, on a black 96-well plate. The plate was incubated at room temperature for 1 h and the fluorescence was measured on a Synergy 4 microplate reader (Biotek, Winooski), with excitation wavelength set to 500 nm, and emission wavelength set to 530–800 nm. The fluorescence emission at 590 nm was used to measure the C_50_, corresponding to the concentration of compound required to induce a 50% decrease in the fluorescence of the EtBr. Experiments were performed in triplicate.

#### Electrophoretic Mobility Assay

Experiments were carried out by mixing 250 ng of pUC18 plasmid DNA (Thermo Scientific; stock solution at 0.5 μg/μL, final concentration at 37.8 μM in nucleotides) and compounds solutions at up to 25 μM, on a final total volume of 20 μL in saline TE buffer. The maximum final DMSO concentration in the treatments was 0.5% (in the 25 μM solutions). Samples were incubated at room temperature for 1 h, either in the dark or under photoactivation. A treatment with 1.75% H_2_O_2_ and 20 μM FeCl_2_ was used as a positive control to generate ROS. To inhibit ROS generation during the treatment, 0.4 M sodium azide (in saline TE buffer) as an oxygen singlet scavenger was added to the reaction mixtures, and DMSO (15%) was added to the positive control as a hydroxyl radical scavenger. Reactions were quenched by adding 4 μL of loading buffer (0.25% w/v bromo-phenol blue, 0.25% w/v xylenecyanole, and 30% v/v glycerol). Samples were then subjected to electrophoresis on 0.8% agarose gel in 0.5 × TBE buffer (0.045 M Tris, 0.045 M boric acid, and 1 mM EDTA) at 90 V for 1 h and 40 min. Finally, DNA was dyed with EtBr (0.5 μg/mL in 0.5× TBE buffer) for 20 min and the DNA bands were visualized on a capturing system (ProgRes CapturePro 2.7). Images were cropped and inverted to better appreciate plasmid bands using Adobe Photoshop. Densitometry analyses of the bands were carried out using the imaging system Fluorochem SP (AlphaInnotech).

#### Cell Death Mechanism

The cell death mechanism was analyzed with the Vybrant Apoptosis Assay Kit (Molecular Probes). A549 cells seeded in 12-well plates at a density of 1 × 10^5^ cells per well were treated with complex **6** at the corresponding IC_50,light_ × 5 under photoactivation conditions. Cisplatin at 50 μM was used as positive control. 4 and 20 h after the irradiation, cells were collected by trypsinization and immediately stained with Annexin V-FITC and propidium iodide according to the manufacturer’s instructions. The median fluorescence of 10,000 cells of each sample was analyzed by flow cytometry. Annexin-FITC staining was detected at a wavelength of 520 nm (FL1) and propidium iodide was detected at 617 nm (FL2).

#### Statistics

The statistical analysis was performed with the GraphPad Prism software. Quantitative variables were expressed as mean or median and standard deviation (SD). Statistical differences were analyzed by the Mann–Whitney nonparametric test. *A* value of *p* < 0.05 was considered significant.

## Supporting Information Available

Scheme of synthesis, NMR spectra, FAB+ and ESI+ spectra, HPLC traces, p*K*_a_ determination, X-ray crystallographic information, stability and photostability studies, ^1^O_2_ generation, photophysical properties, TD-DFT calculations, biological properties, and NADH oxidation (PDF)
Molecular Formula Strings (CSV)

## Abbreviations Used

AN: annexin
AO: acridine orange
ATP: adenosine triphosphate
bhq: benzo[*h*]quinoline
bzIm: *N*-benzylimidazole
CCCP: carbonyl cyanide *m*-chlorophenyl hydrazone
CM-H_2_DCFDA: 2′,7′-dichlorodihydrofluorescein diacetate
Cp*: 1,2,3,4,5-pentamethylcyclopentadienyl
CT-DNA: Calf Thymus DNA
DFC: 2′,7′-dichlorodihydrofluorescein
DMEM: Dulbecco’s modified Eagle’s medium
Doxo: Doxorubicin
DPBF: 1,3-diphenylisobenzofuran
EDTA: ethylenediaminetetracetic Acid
ER: endoplasmic reticulum
EtBr: ethidium bromide
FBS: fetal bovine serum
HOMO: highest occupied molecular orbital
IC: internal conversion
ICP-MS: inductively coupled plasma mass spectrometry
Im: imidazole
Imepip: (*N*-ethylpiperidyl)imidazole
ISC: intersystem crossing
JC-1: 5,5′,6,6′-tetrachloro-1,1′,3,3′-tetraethylbenzimidazolocarbo-cyanine iodide
LC: ligand-centered
LUMO: lowest occupied molecular orbital
MC: metal-centered
MLCT: metal to ligand charge transfer
MMP: mitochondrial membrane potential
mtDNA: mitochondrial DNA
MTT: 3-(4,5-dimethylthiazol-2-yl)–2,5-diphenyltetrazolium bromide
NaAz: sodium azide
NADH: nicotinamide adenine dinucleotide
OC: open circular
OSW: octanol-saturated water
pbpn: 4,9,16-triazadibenzo[*a*,*c*]naphthacene
pbpq: benzo[*f*]pyrido[2,3-*h*]quinoxaline
pbpz: 4,9,14-triazadibenzo[*a*,*c*]anthracene
PBS: phosphate-buffered saline
PDT: photodynamic therapy
PI: phototoxic index
ppy: 2-phenylpyridinate
PS: photosensitizer
RBC: red blood cells
RMSD: root-mean-square deviation
ROS: reactive oxygen species
rRNA: ribosomal RNA
SC: supercoiled
SMD: solvation model
SOC: spin–orbit coupling
TAS: transient absorption spectroscopy
TD-DFT: time-dependent density functional theory
terpy: 2,2′:6′,2″-terpyridine
TON: turnover number
tRNA: transfer RNA
WSO: water-saturated octanol
