# Supplementary material for: Ir(III) Half-Sandwich Photosensitizers with a π-Expansive Ligand for Efficient Anticancer Photodynamic Therapy
Source: J Med Chem. 2024 Jan 31;67(3):1783–811. doi: 10.1021/acs.jmedchem.3c01276 (PMC10859961; doi:10.1021/acs.jmedchem.3c01276)
Supplement: Supplementary file 1 — jm3c01276_si_001.pdf [file jm3c01276_si_001.pdf]

SUPPORTING INFORMATION

Ir(III) Half-sandwich Photosensitizers with a π-expansive ligand for efficient Anticancer Photodynamic Therapy

Carlos Gonzalo-Navarro,[1] Elisenda Zafon,[2] Juan Angel Organero,[3] Félix A. Jalón,[1] Joao Carlos Lima,[4] Gustavo Espino,[5] Ana María Rodríguez,[6] Lucía Santos,[7] Artur Moro,[4] Sílvia Barrabés,[2] Jessica Castro,[2] Javier Camacho-Aguayo,[8] Anna Massaguer,*,[2] Blanca R. Manzano,*,[1] Gema Durá*,[1]

[1] Universidad de Castilla-La Mancha, Departamento de Química Inorgánica, Orgánica y Bioquímica-IRICA, Facultad de Ciencias y Tecnologías Químicas, Avda. C. J. Cela, 10, 13071 Ciudad Real, Spain.

[2] Universitat de Girona, Departament de Biologia, Facultat de Ciències, Maria Aurèlia Capmany 40, 17003 Girona, Spain.

[3] Universidad de Castilla-La Mancha, Departamento de Química Física, Facultad de Ciencias Ambientales y Bioquímicas and INAMOL, 45071 Toledo, Spain.

[4] Universidade NOVA de Lisboa, LAQV-REQUIMTE, Departamento de Química, Faculdade de Ciências e Tecnologia, 2829-516 Caparica, Portugal.

[5] Universidad de Burgos, Departamento de Química, Facultad de Ciencias, Pza. Misael Bañuelos, s/n, 09001 Burgos, Spain.

[6] Universidad de Castilla-La Mancha, Departamento de Química Inorgánica, Orgánica y Bioquímica-IRICA, Escuela Técnica Superior de Ingenieros Industriales, Avda. C. J. Cela, 3, 13071 Ciudad Real, Spain.

[7] Universidad de Castilla-La Mancha, Departamento de Química Física, Facultad de Ciencias y Tecnologías Químicas, Avda. C. J. Cela, s/n, 13071 Ciudad Real, Spain.

[8] Analytical Chemistry Department, Analytic Biosensors Group, Instituto de Nanociencia y Nanomateriales de Aragon, Faculty of Sciences, University of Zaragoza, 50009-Zaragoza, Spain.

Index

Scheme of synthesis: p. S3

NMR spectroscopy: p. S3-S16.

Mass spectrometry: p. S17-S21.

HPLC traces: p. S22-S26

pKa determination: S26-S27

X-ray diffraction: p. S27-S28.

Stability and photostability studies: p. S28-S36.

$^1O_2$ generation: p. S37-S41.

Photophysical properties (UV-vis absorption, Emission, Transient Absorption Spectroscopy (TAS)): p.S42-S46.

TD-DTF calculations: p. S47-S50.

Biological properties (lipophilicity, cytotoxic activity, hemolytic activity, intracellular ROS production, cellular uptake, NADH oxidation, DNA intercalation, lysosomal damage): p. S51-S64.

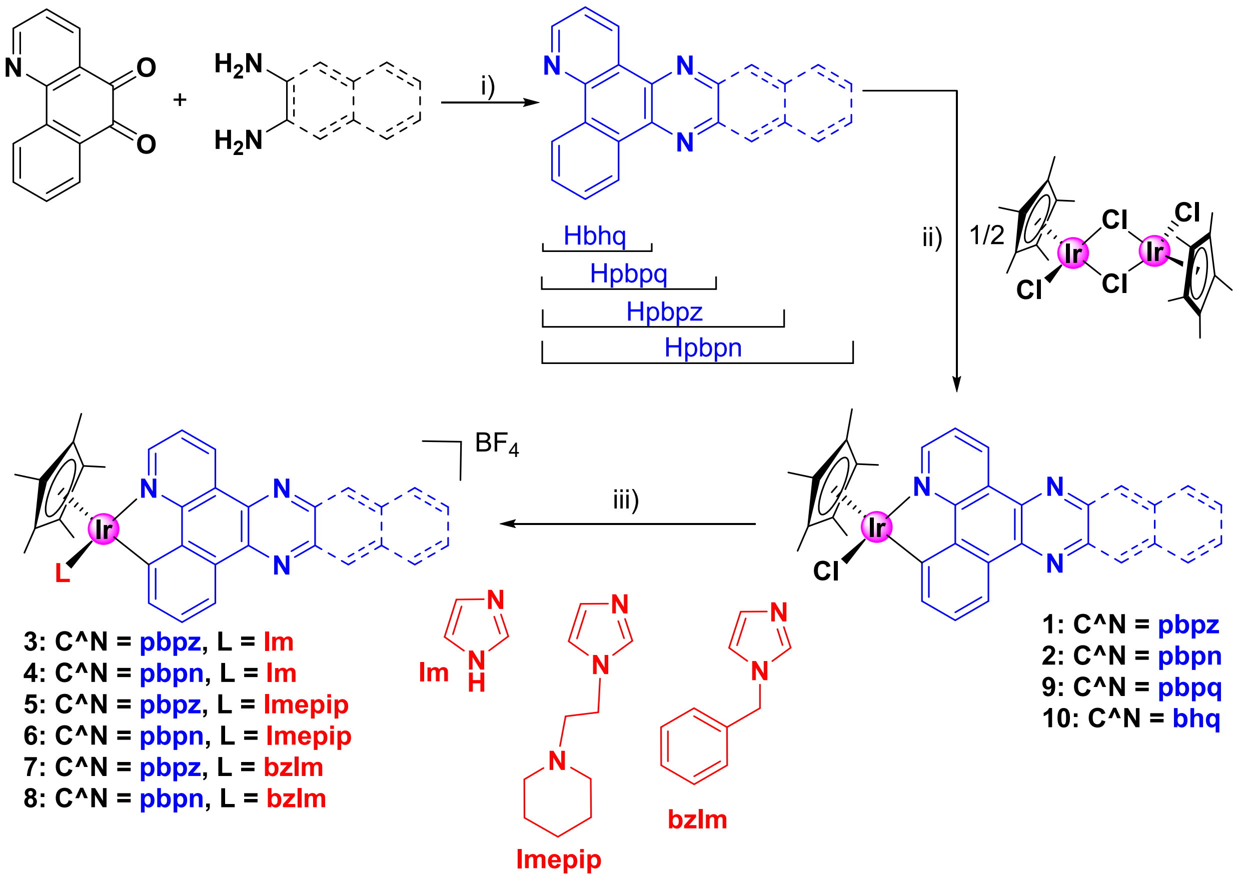

Scheme S1. Synthesis of ligands and complexes presented in this paper. i) EtOH, reflux, 4 h. ii) Na_2CO_3, $AgOCOCF_3$ in DCM, at room temperature for 4.5 h. iii) 1.1 eq of $AgBF_4$, 1 eq of Im, Imepip or bzIm, respectively, in DCM at room temperature for 4 h.

NMR spectroscopy

Table S1. ^{1}H NMR shift of proligands and complexes in $CDCl_3$ (proligands, **1**-**3** and **5**-**7**) or $(CD_3)_2CO$ (**4** and **8**). The resonance of ligands L are omitted for clarity.

Section a: Hpbpz and its complexes

Complex	H^4	H^5	H^6	H^7	H^{10}	H^{11}	H^{12}	H^{19}	H^{20}	H^{21}	H^{22}	Cp*
Hpbpz	9.08	7.68	9.60	9.38	9.24	7.92-7.84	7.92-7.84	7.92-7.84	7.92-7.84	8.36	8.36	-
**1**	9.42	7.59	9.04	-	8.80	7.71	8.19	8.37	8.31	7.88	7.88	1.78
**3**	9.48	7.84-7.76	9.25	-	8.90	7.84-7.76	8.26	8.34	8.29	7.86	7.86	1.70
**5**	9.42	7.83	9.38	-	8.83	7.73	8.17	8.23	8.23	7.83	7.83	1.72
**7**	9.47	8.00	9.62	-	8.92	7.77	8.23	8.38	8.33	7.90	7.90	1.72

Section b: Hpbpn and its complexes

Complex	H^4	H^5	H^6	H^7	H^{10}	H^{11}	H^{12}	H^{19}	H^{20}	H^{23}	H^{24}	H^{25}	H^{26}	Cp*
Hpbpn	9.05	7.68	9.60	9.38	9.18	7.87	7.87	8.96	8.93	8.20	8.20	7.61	7.61	-
**2**	9.38	7.57	9.01	-	8.79	7.69	8.18	8.90	8.86	8.16	8.16	7.57	7.57	1.78
**4**	9.33	7.93	9.61	-	8.71	7.80	8.48	8.78	8.78	8.16	8.16	7.53	7.53	1.82
**6**	9.37	7.88	9.49	-	8.82	7.78	8.24	8.85	8.85	8.16	8.16	7.60	7.60	1.73
**8**	9.34	7.93	9.59	-	8.72	7.79	8.45	8.82	8.82	8.21	8.21	7.59	7.59	1.81

Table S2. ^{1}H NMR shift of Hpbpq and complex **9**, Hbhq and complex **10** in $CDCl_3$

Complex	H^4	H^5	H^6	H^7	H^{10}	H^{11}	H^{12}	H^{13}	H^{14}	H^{17}	H^{18}	Cp*
Hpbpq	9.42	7.67	9.09	9.28	9.18	7.87	7.87	-	-	8.95	8.92	-
**9**	9.27	7.60	9.06	-	8.64	7.73	8.20	-	-	8.96	8.87	1.77
Hbhq	9.30	7.53	9.01	8.18	7.92	7.78-7.67	7.78-7.67	7.82	7.78-7.67	-	-	-
**10**	8.14	7.47	8.96	-	8.06	7.62	7.82	7.56	7.56	-	-	1.74

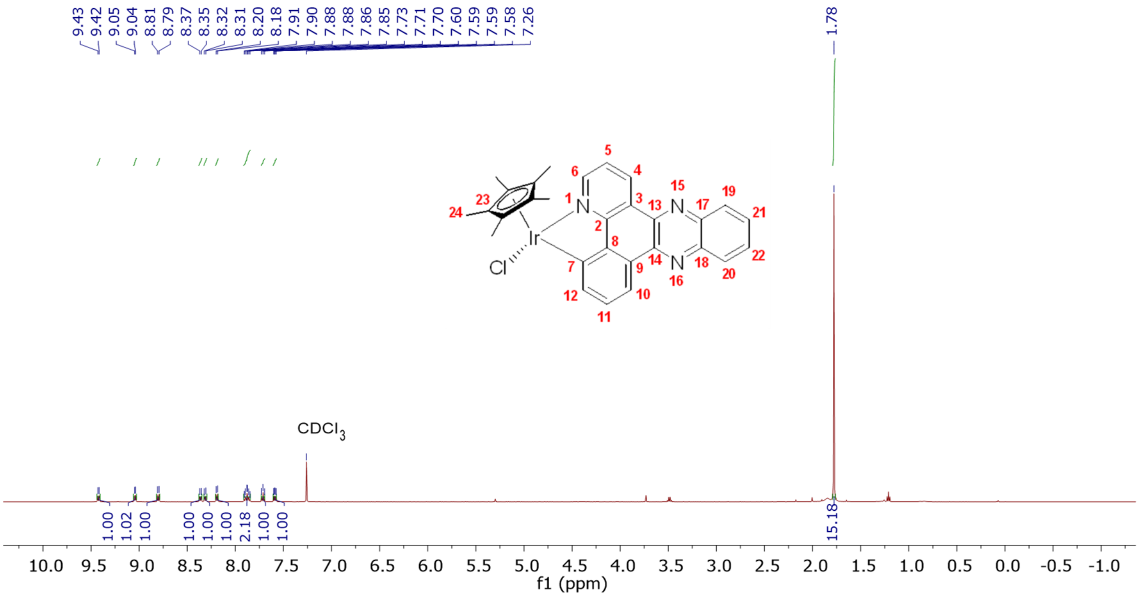

Figure S1. ^{1}H NMR (500 MHz, $CDCl_3$, 298 K) spectrum of **1**.

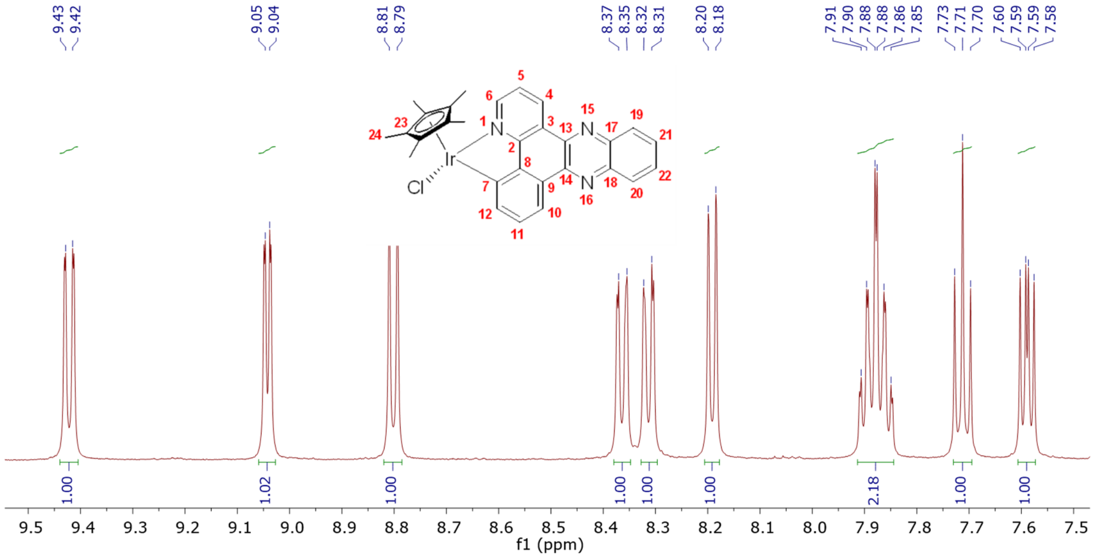

Figure S2. ^{1}H NMR (500 MHz, $CDCl_3$, 298 K) spectrum of **1** in the aromatic region.

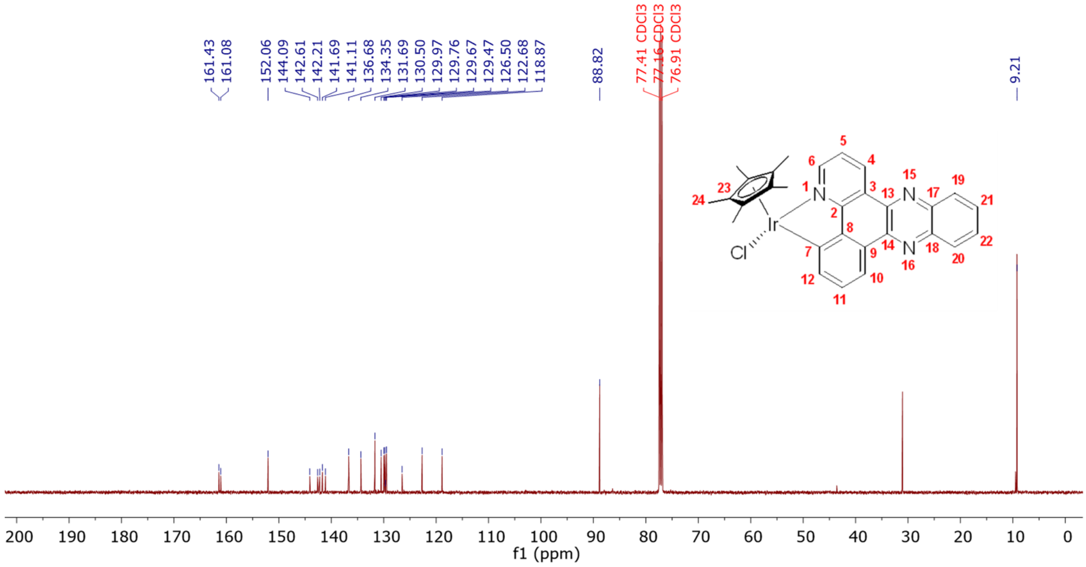

Figure S3. $^{13}C\{^1H\}$ NMR (126 MHz, $CDCl_3$, 298 K) spectrum of **1**.

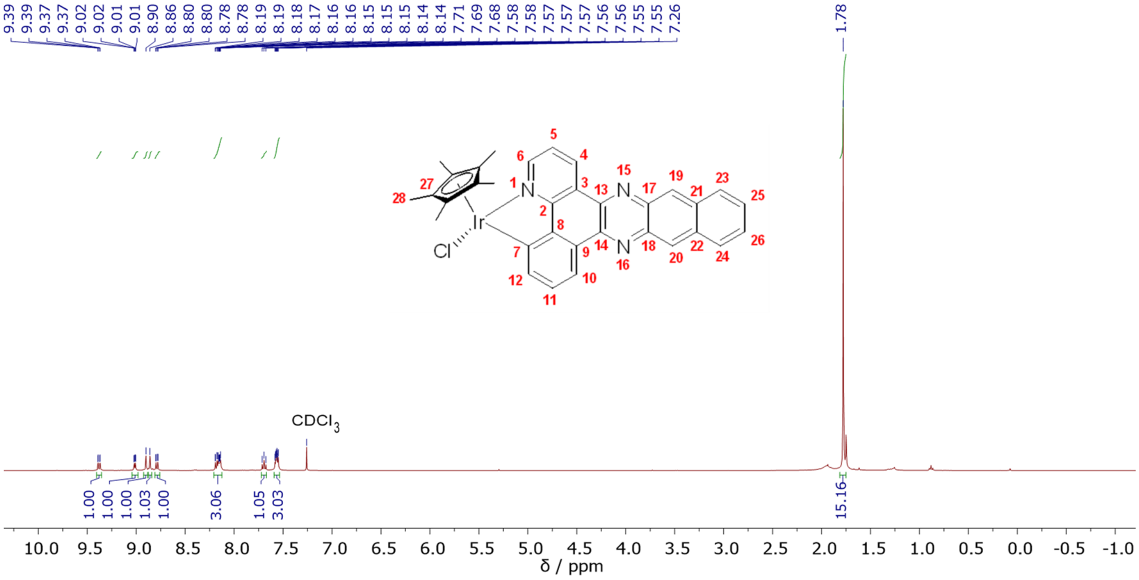

Figure S4. ^1H NMR (400 MHz, $CDCl_3$, 298 K) spectrum of **2**.

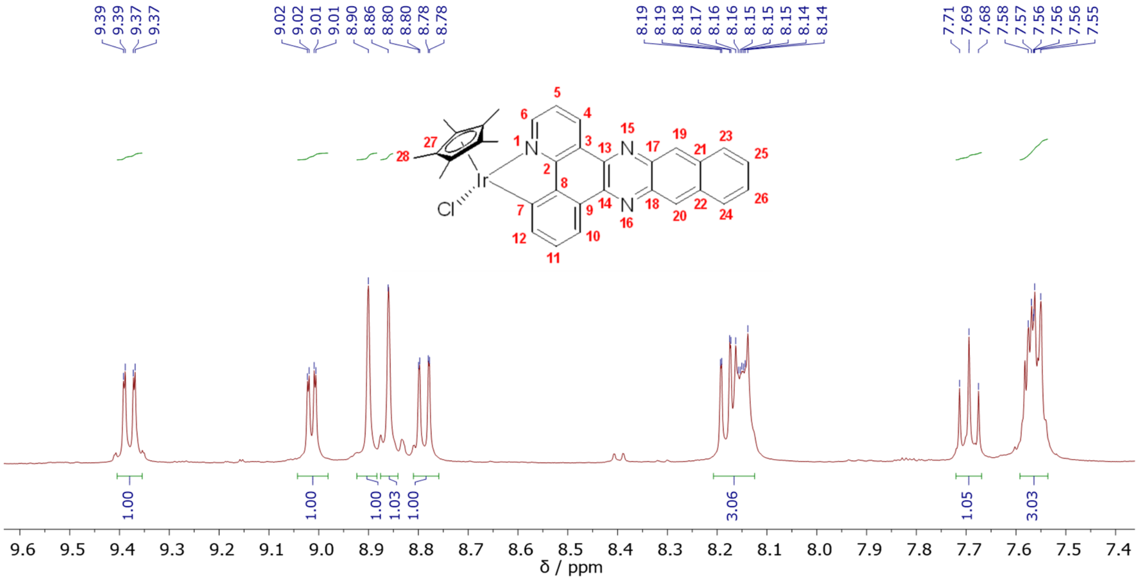

Figure S5. ^{1}H NMR (400 MHz, $CDCl_3$, 298 K) spectrum of **2** in the aromatic region.

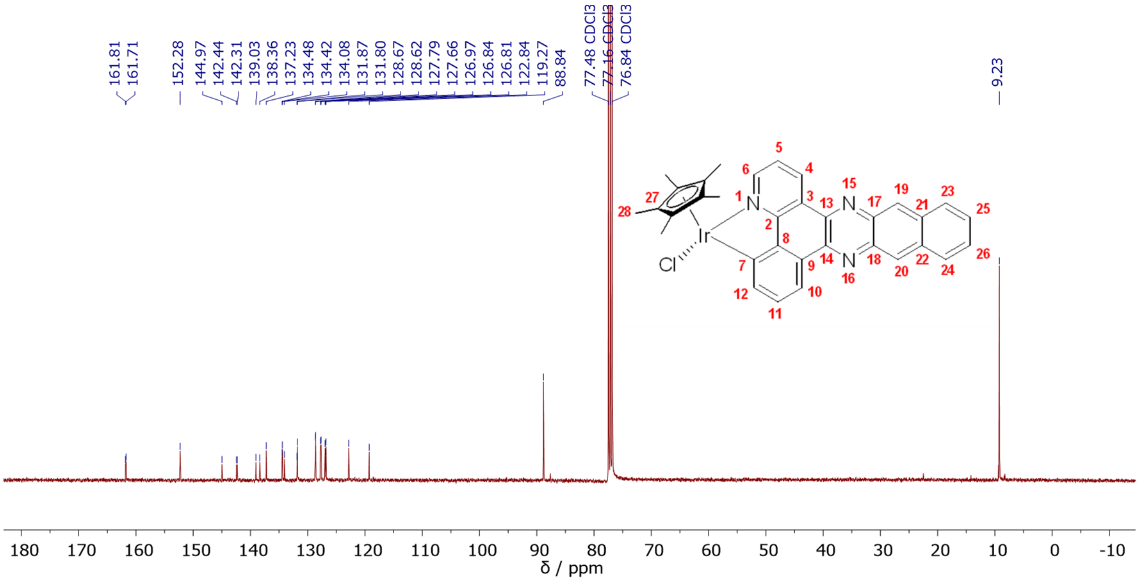

Figure S6. $^{13}C\{^{1}H\}$ NMR (101 MHz, $CDCl_3$, 298 K) spectrum of **2**.

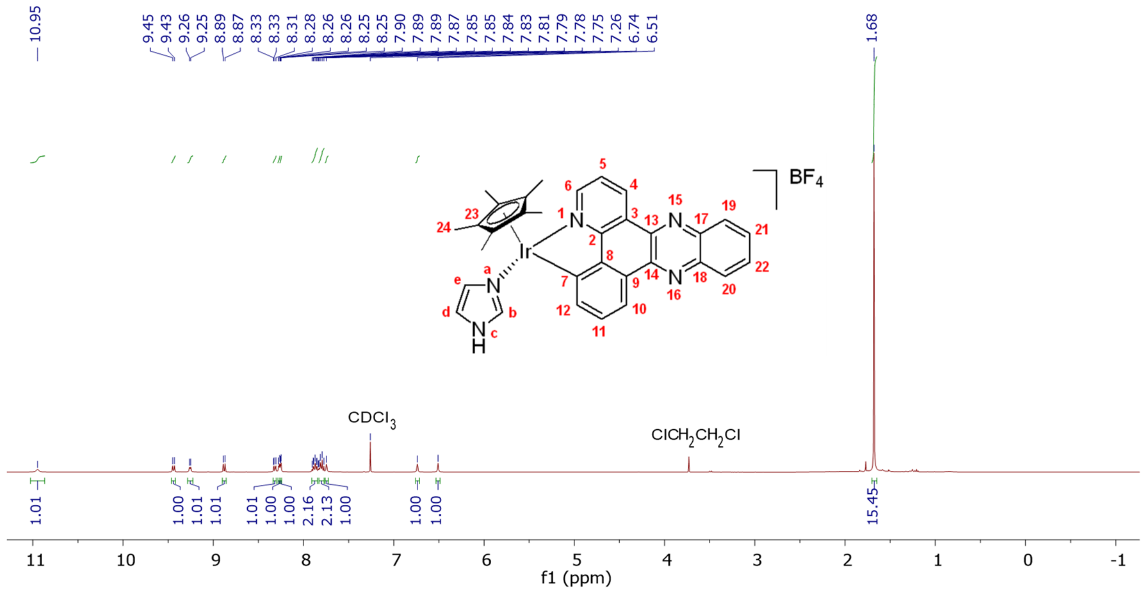

Figure S7. ^{1}H NMR (400 MHz, $CDCl_3$, 298 K) spectrum of **3**.

.

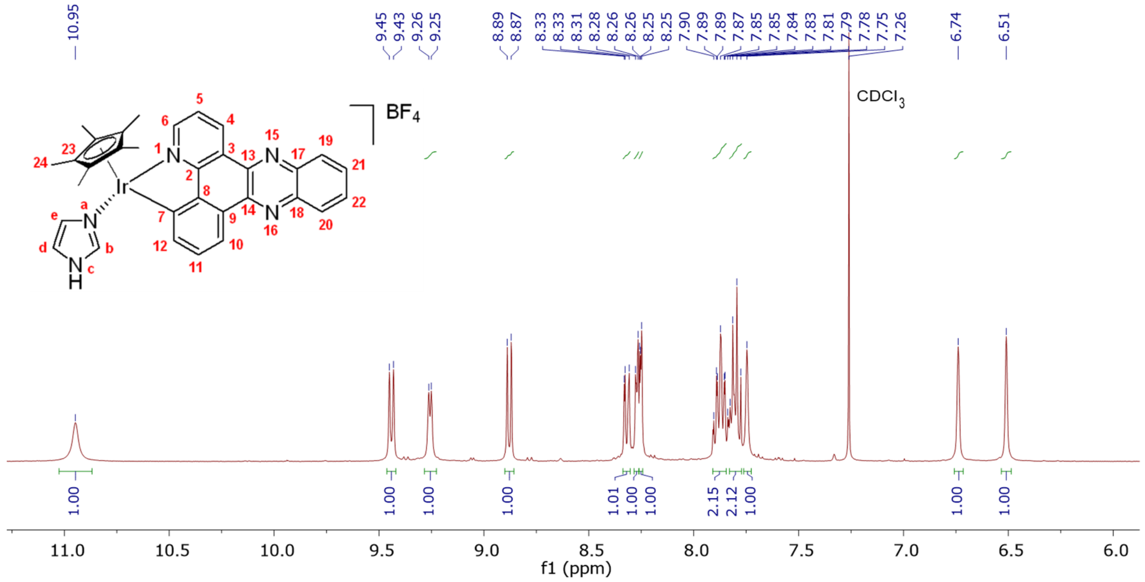

Figure S8. ^{1}H NMR (400 MHz, $CDCl_3$, 298 K) spectrum of **3** in the aromatic region.

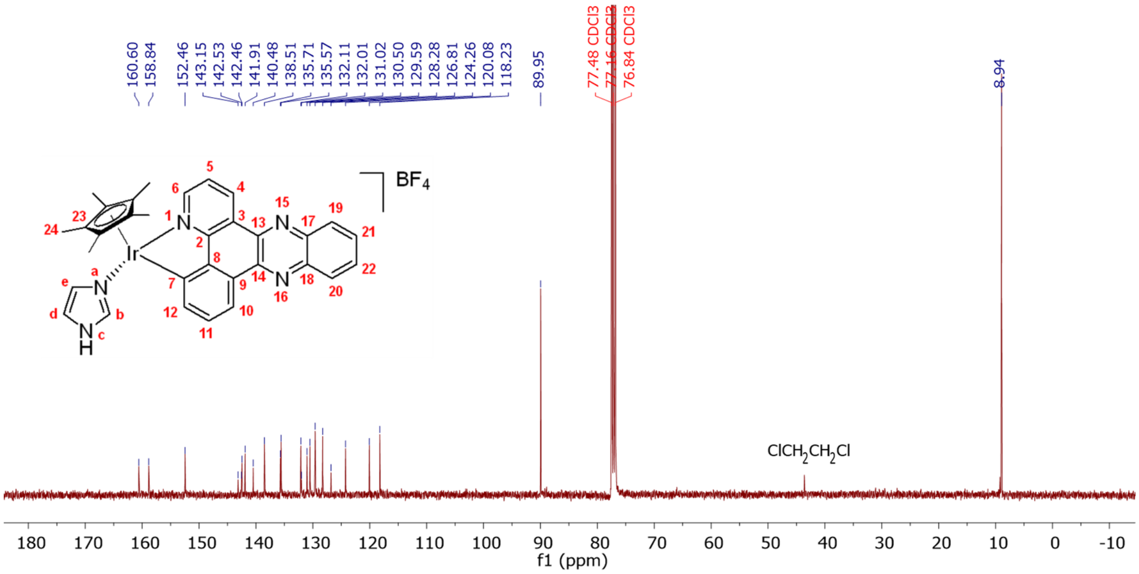

Figure S9. $^{13}C\{^{1}H\}$ NMR (101 MHz, $CDCl_3$, 298 K) spectrum of **3**.

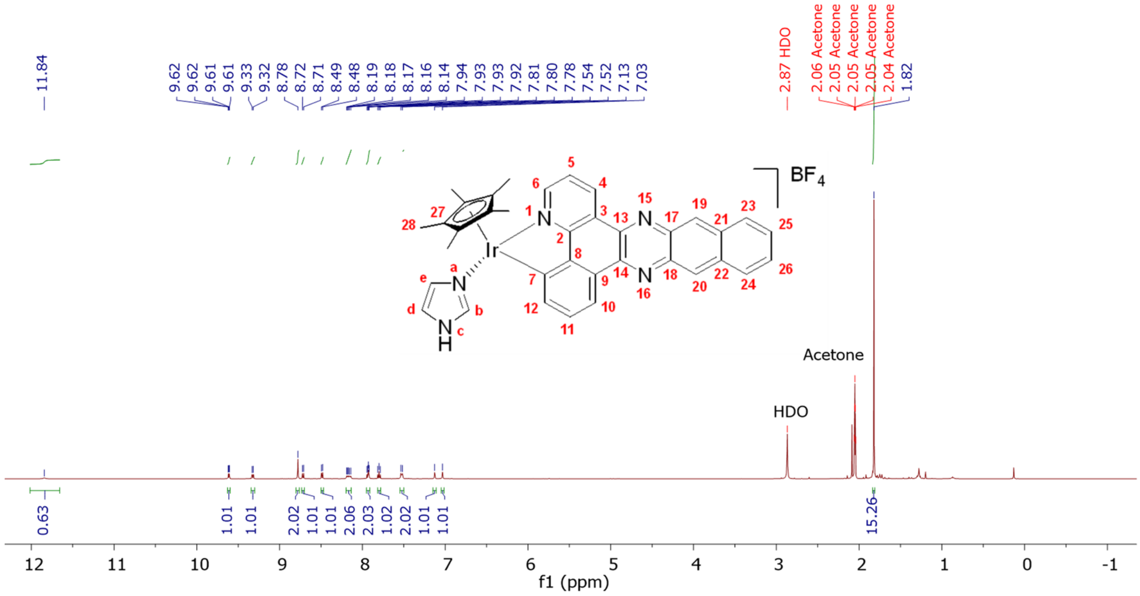

Figure S10. ^{1}H NMR (500 MHz, $(CD_3)_2CO$, 298 K) spectrum of **4**.

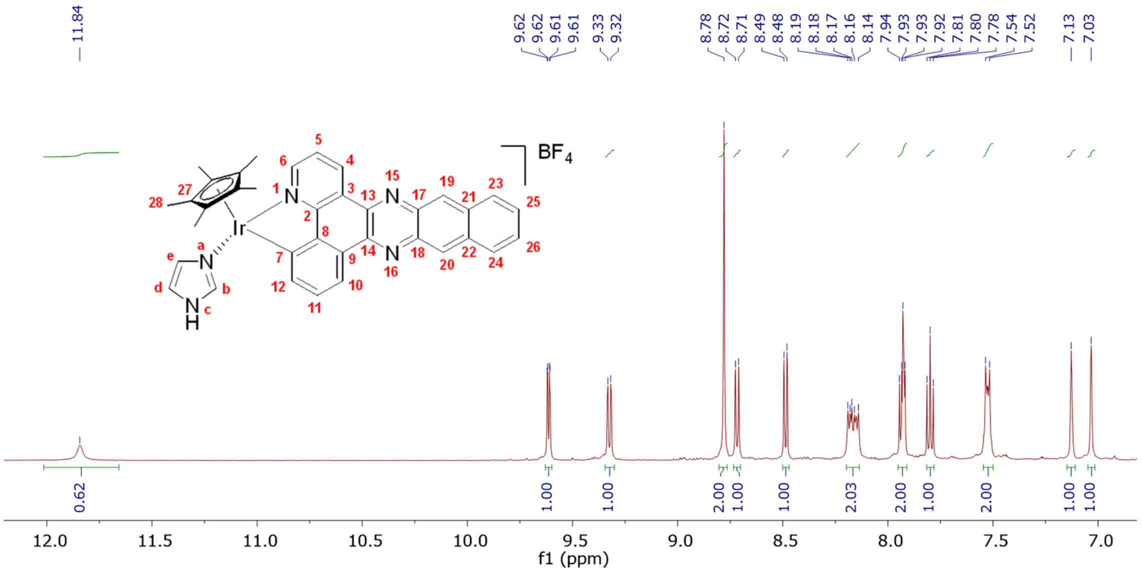

Figure S11. ^{1}H NMR (500 MHz, $(CD_3)_2CO$, 298 K) spectrum of **4** in the aromatic region.

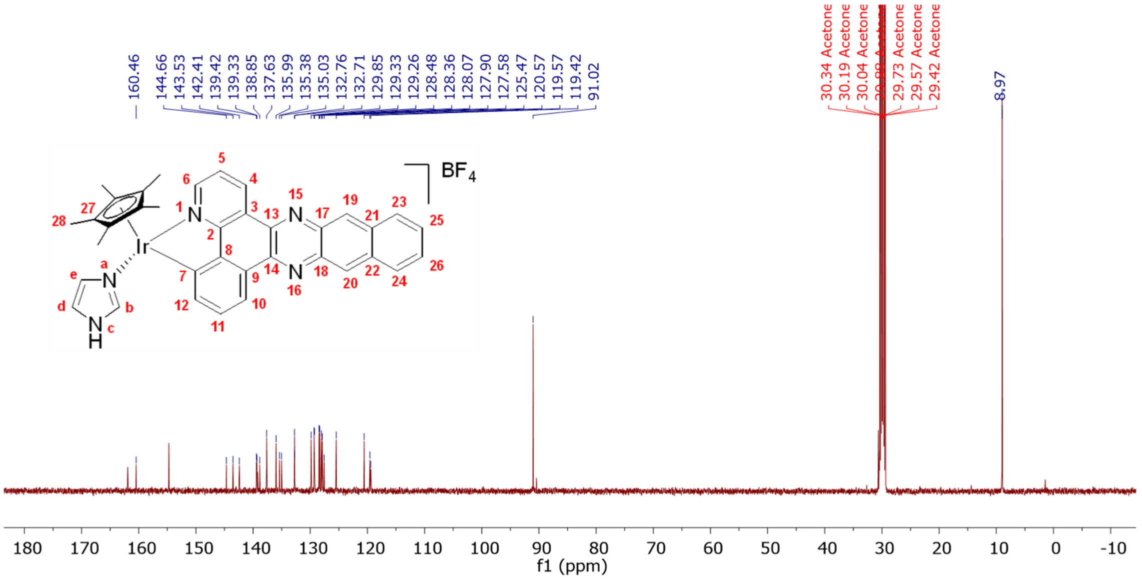

Figure S12. $^{13}C\{^1H\}$ NMR (126 MHz, $(CD_3)_2CO$, 298 K) spectrum of **4**.

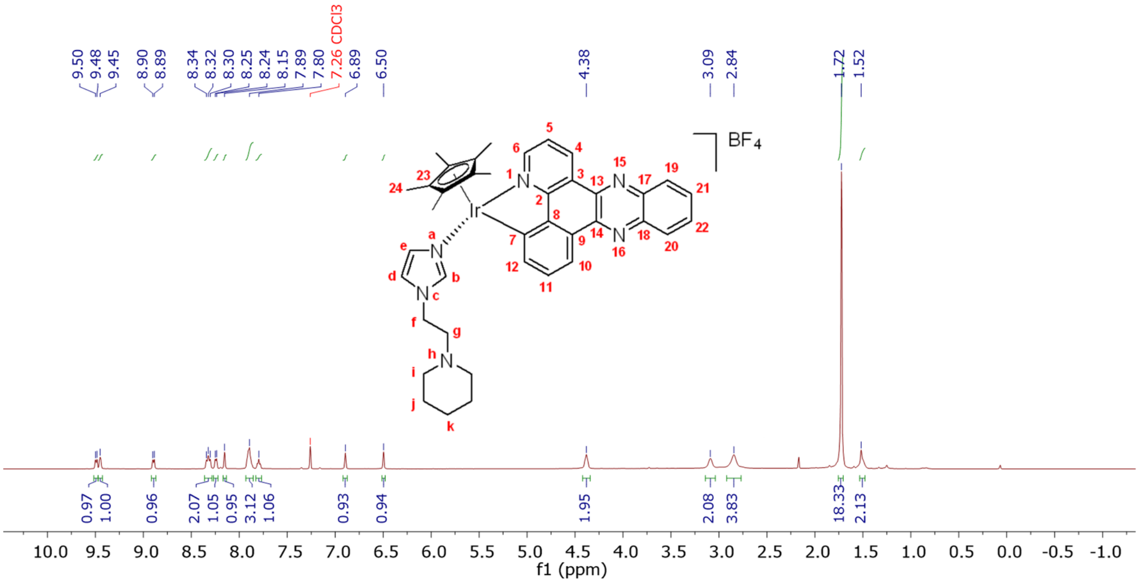

Figure S13. ^{1}H NMR (500 MHz, $CDCl_3$, 298 K) spectrum of **5**.

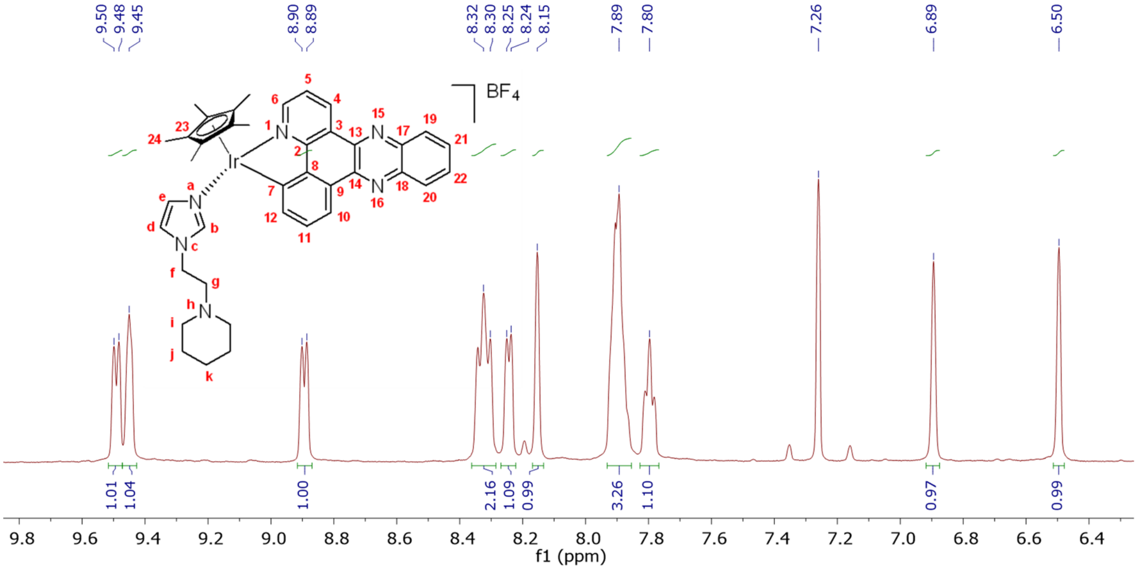

Figure S14. ^{1}H NMR (500 MHz, $CDCl_3$, 298 K) spectrum of **5** in the aromatic region.

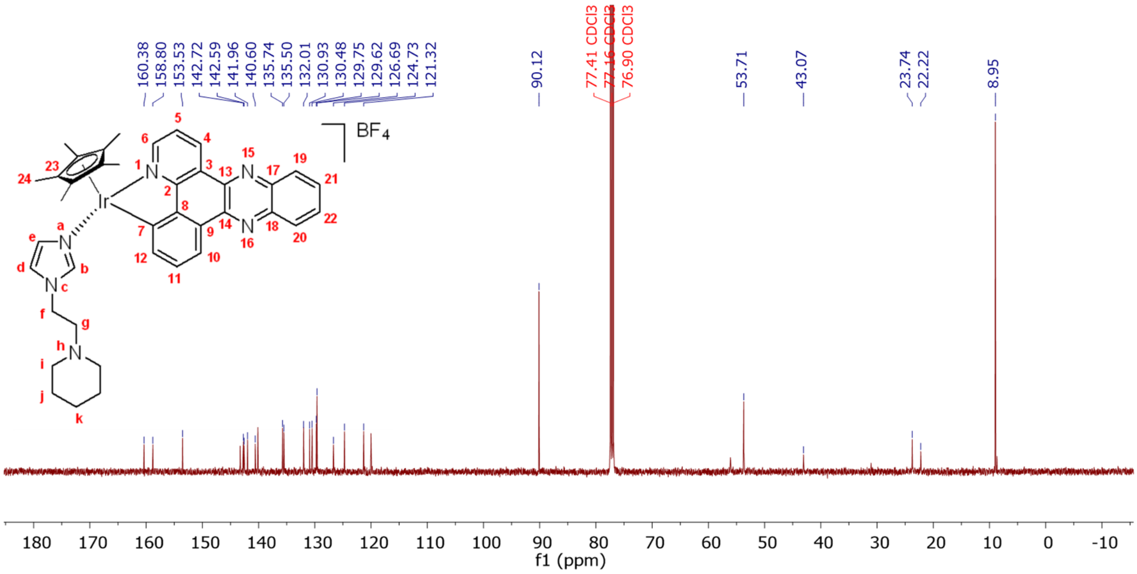

Figure S15. $^{13}C\{^1H\}$ NMR (126 MHz, $CDCl_3$, 298 K) spectrum of **5**.

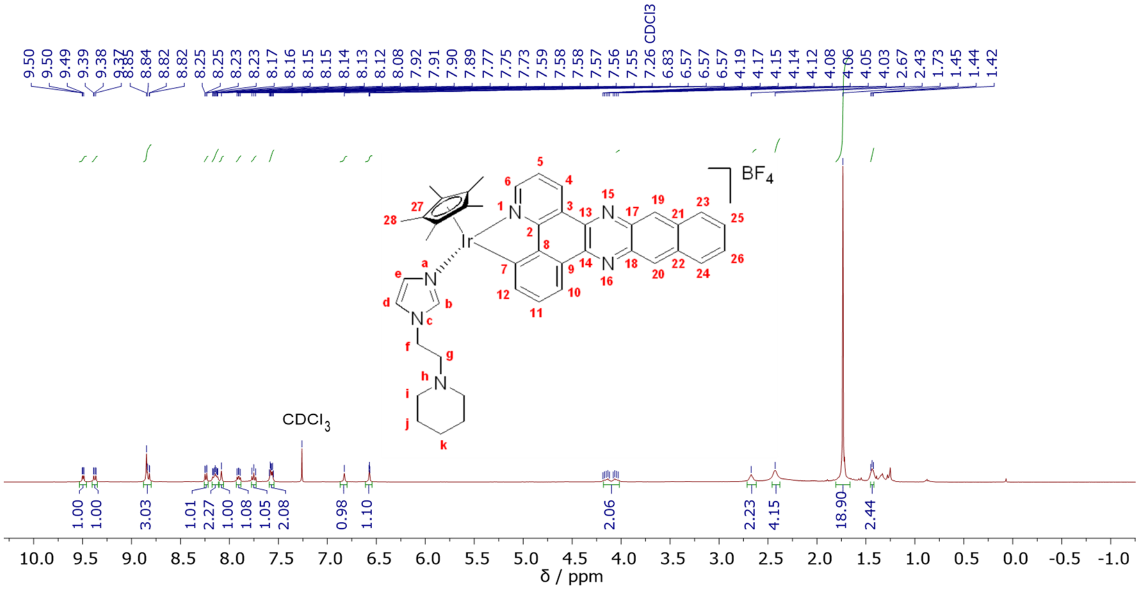

Figure S16. ^1H NMR (400 MHz, $CDCl_3$, 298 K) spectrum of **6**.

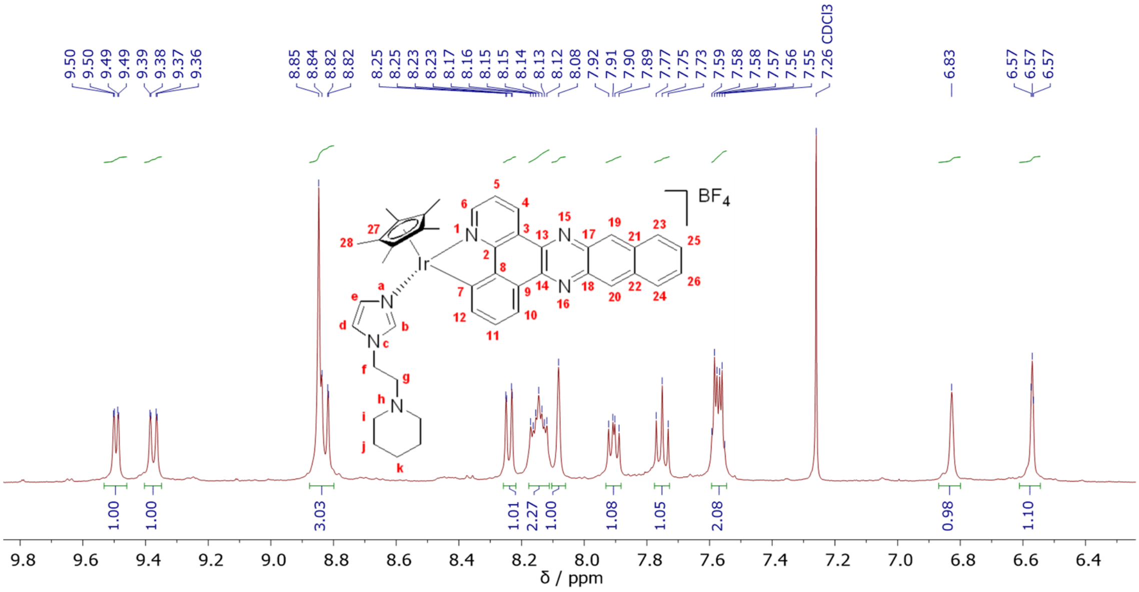

Figure S17. ^{1}H NMR (400 MHz, $CDCl_3$, 298 K) spectrum of **6** in the aromatic region.

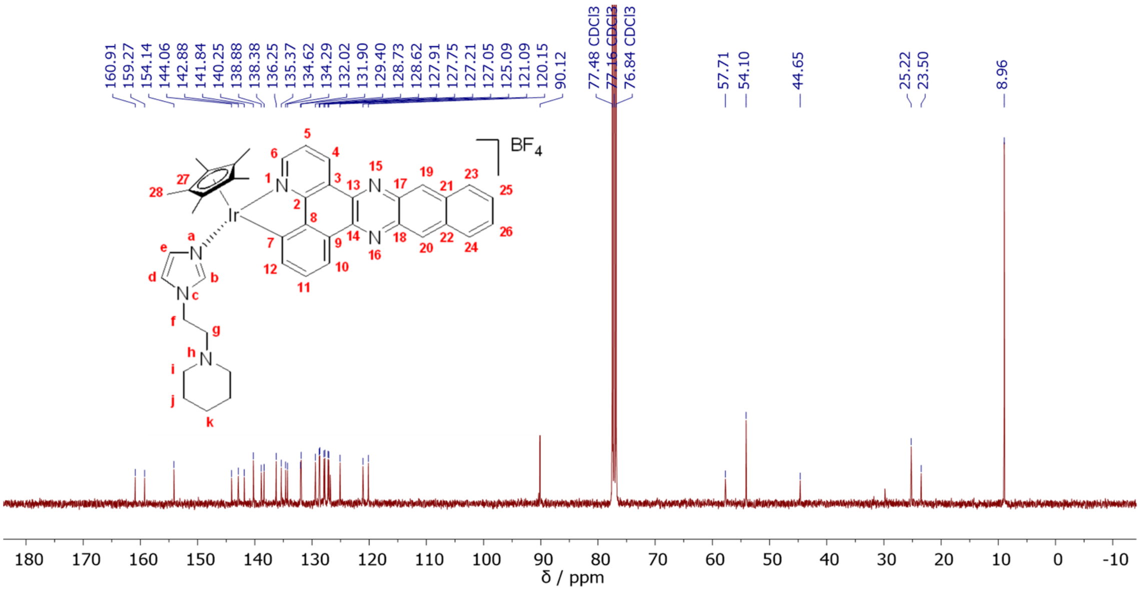

Figure S18. $^{13}C\{^{1}H\}$ NMR (101 MHz, $CDCl_3$, 298 K) spectrum of **6**.

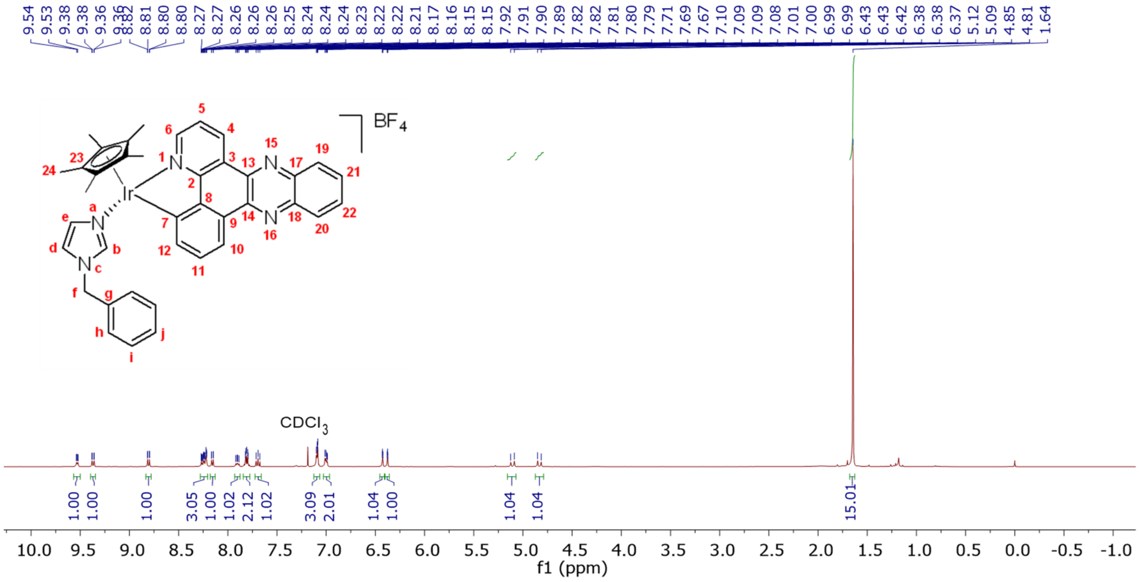

Figure S19. ^{1}H NMR (400 MHz, $CDCl_3$, 298 K) spectrum of **7**.

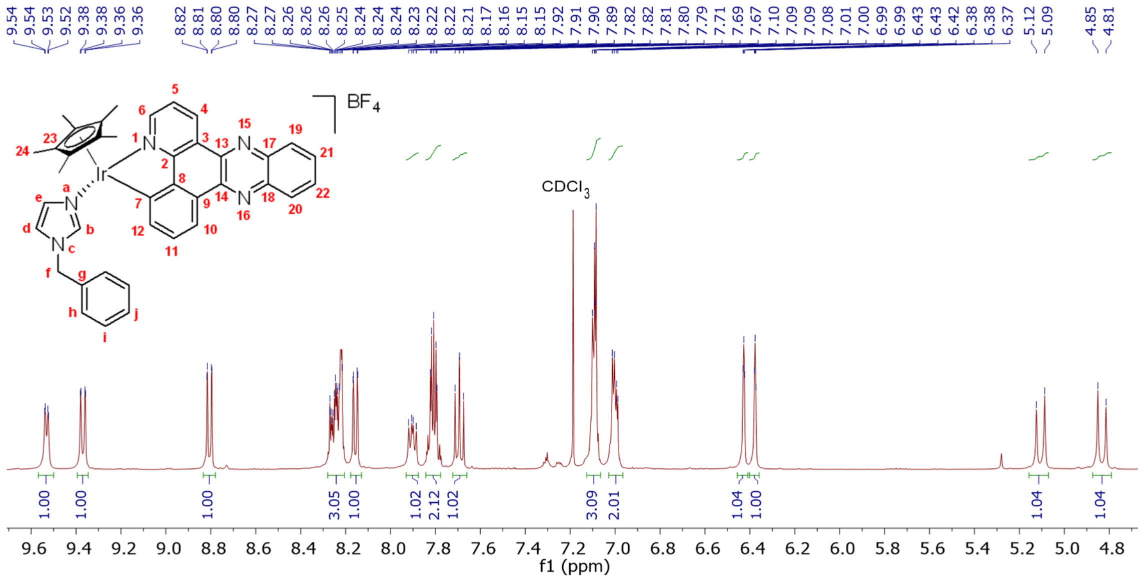

Figure S20. ^{1}H NMR (400 MHz, $CDCl_3$, 298 K) spectrum of **7** in the aromatic region.

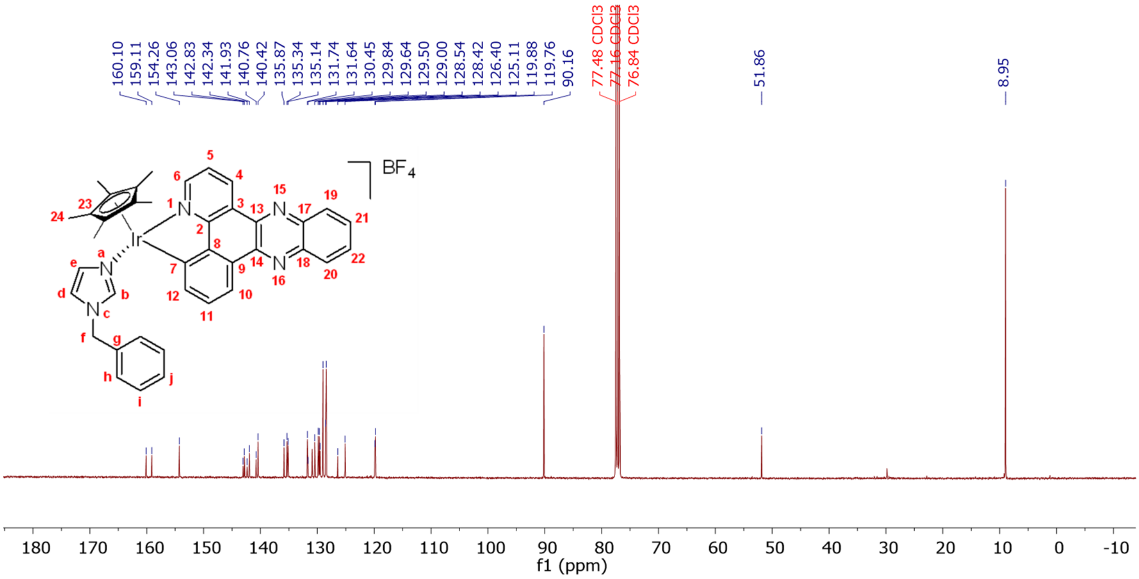

Figure S21. $^{13}C\{^{1}H\}$ NMR (101 MHz, $CDCl_3$, 298 K) spectrum of **7**.

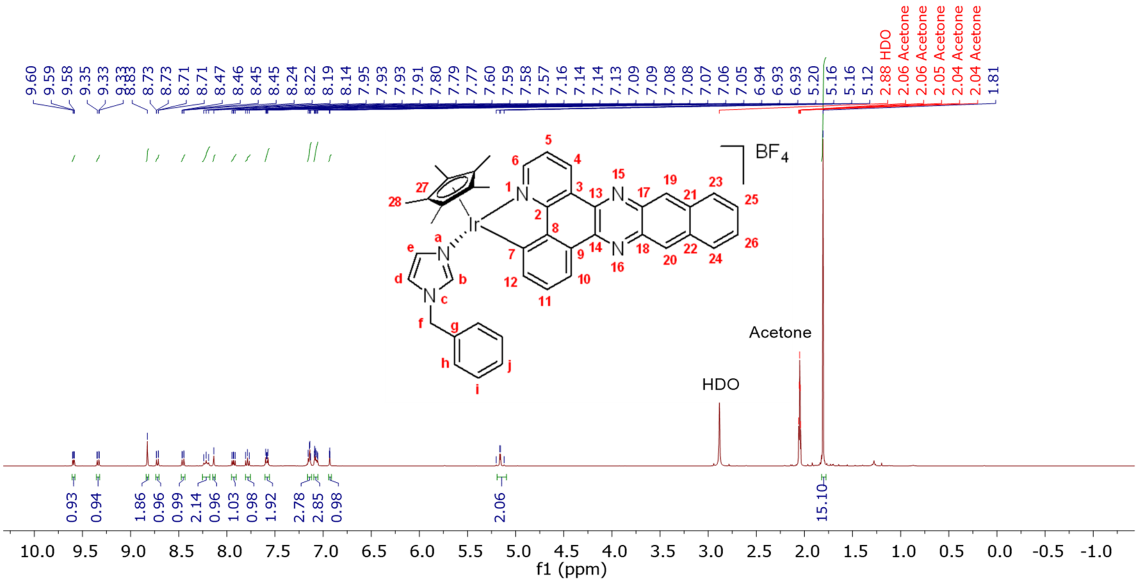

Figure S22. ^{1}H NMR (400 MHz, $(CD_3)_2CO$, 298 K) spectrum of **8**.

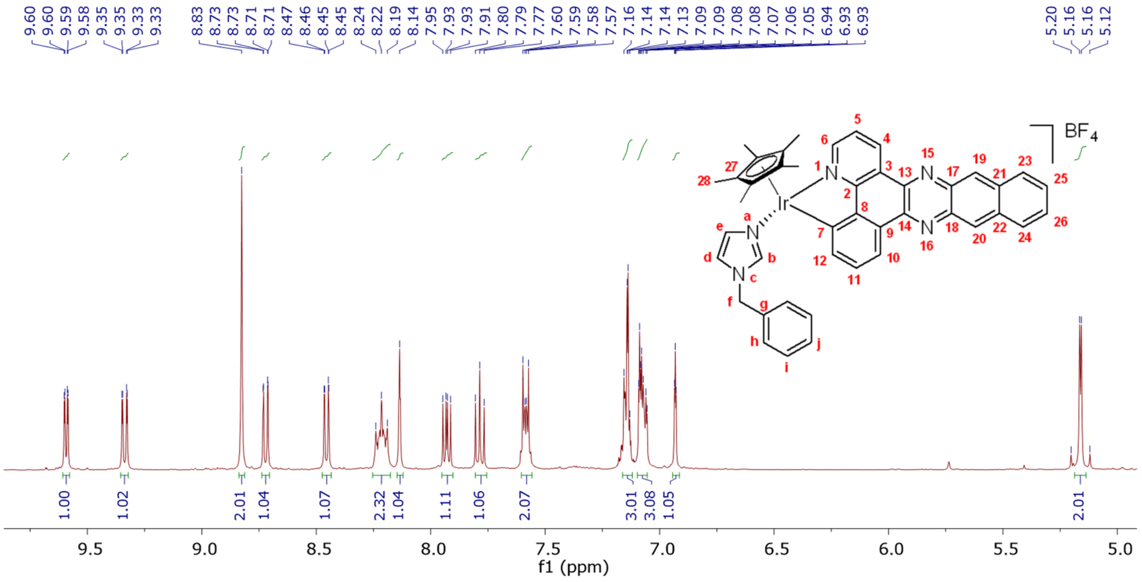

Figure S23. ^{1}H NMR (400 MHz, $(CD_3)_2CO$, 298 K) spectrum of **8** in the aromatic region.

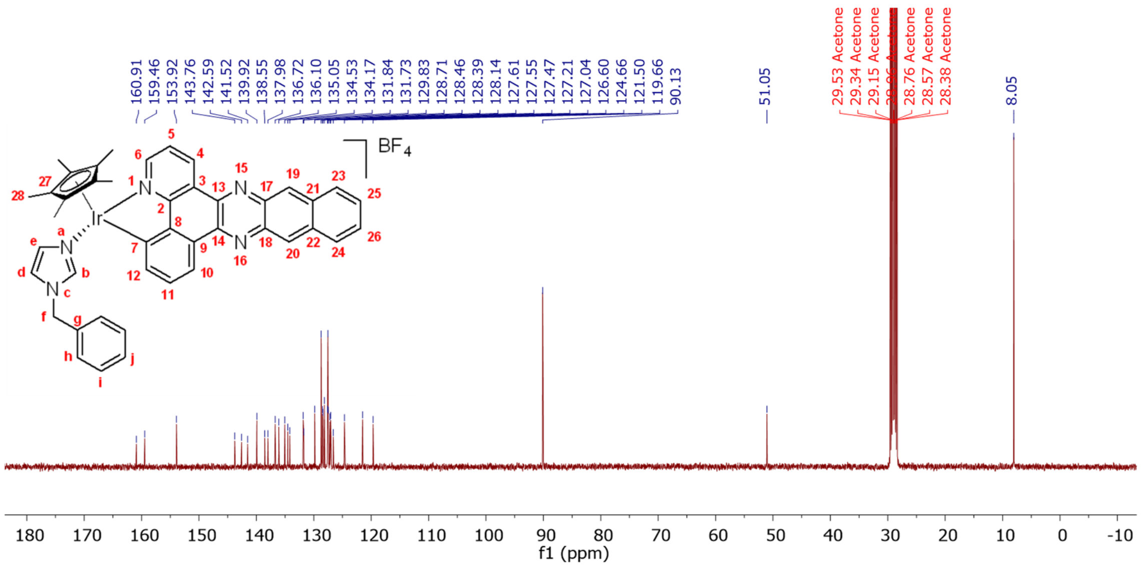

Figure S24. $^{13}C\{^{1}H\}$ NMR (101 MHz, $(CD_3)_2CO$, 298 K) spectrum of **8**.

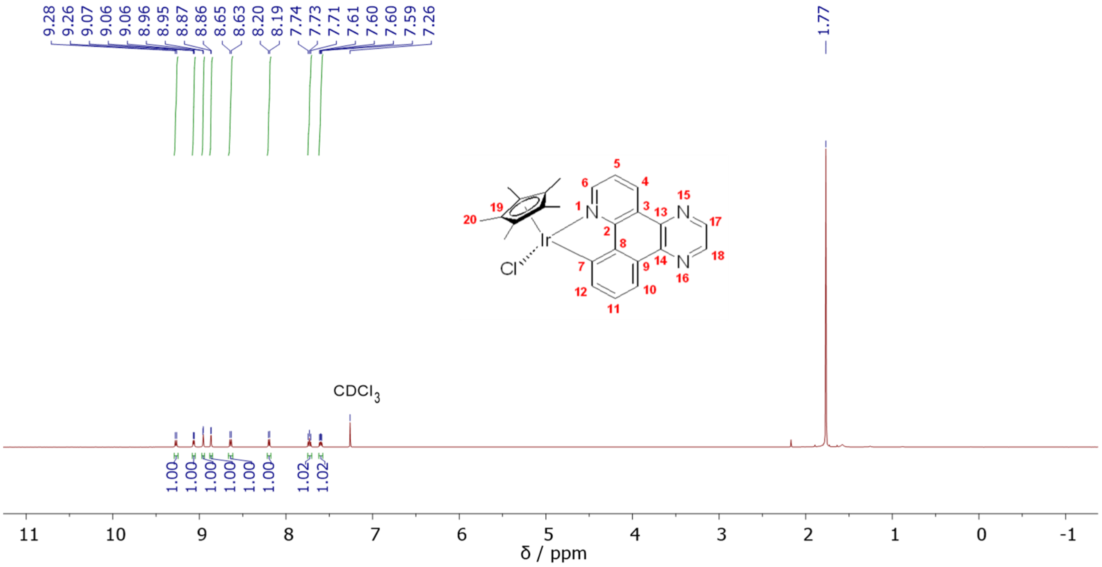

Figure S25. ^{1}H NMR (500 MHz, $CDCl_3$, 298 K) spectrum of **9**.

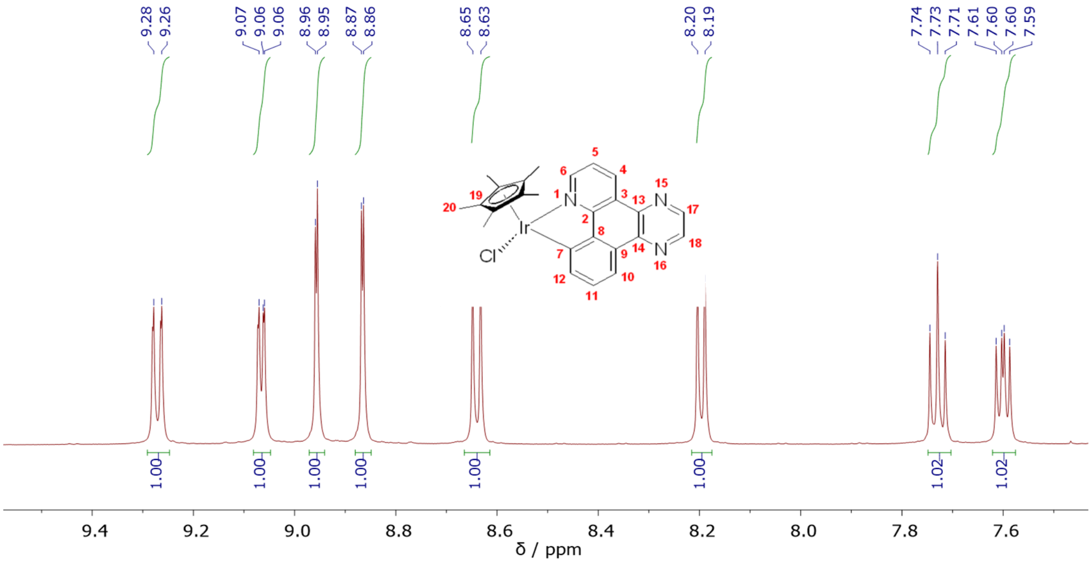

Figure S26. ^{1}H NMR (500 MHz, $CDCl_3$, 298 K) spectrum of **9** in the aromatic region.

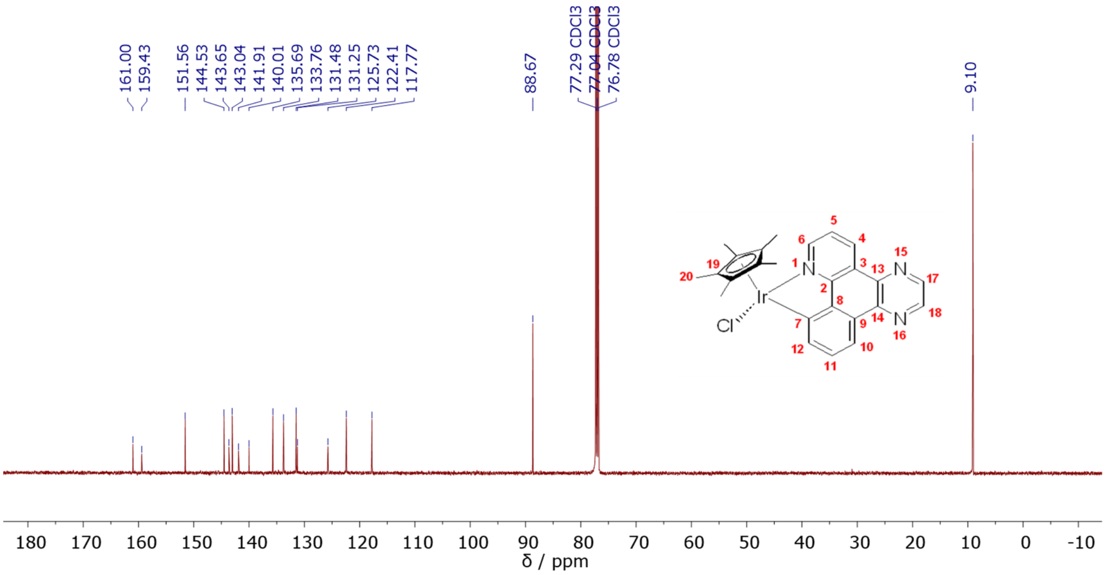

Figure S27. $^{13}C\{^{1}H\}$ NMR (126 MHz, $CDCl_3$, 298 K) spectrum of **9**.

Mass spectrometry

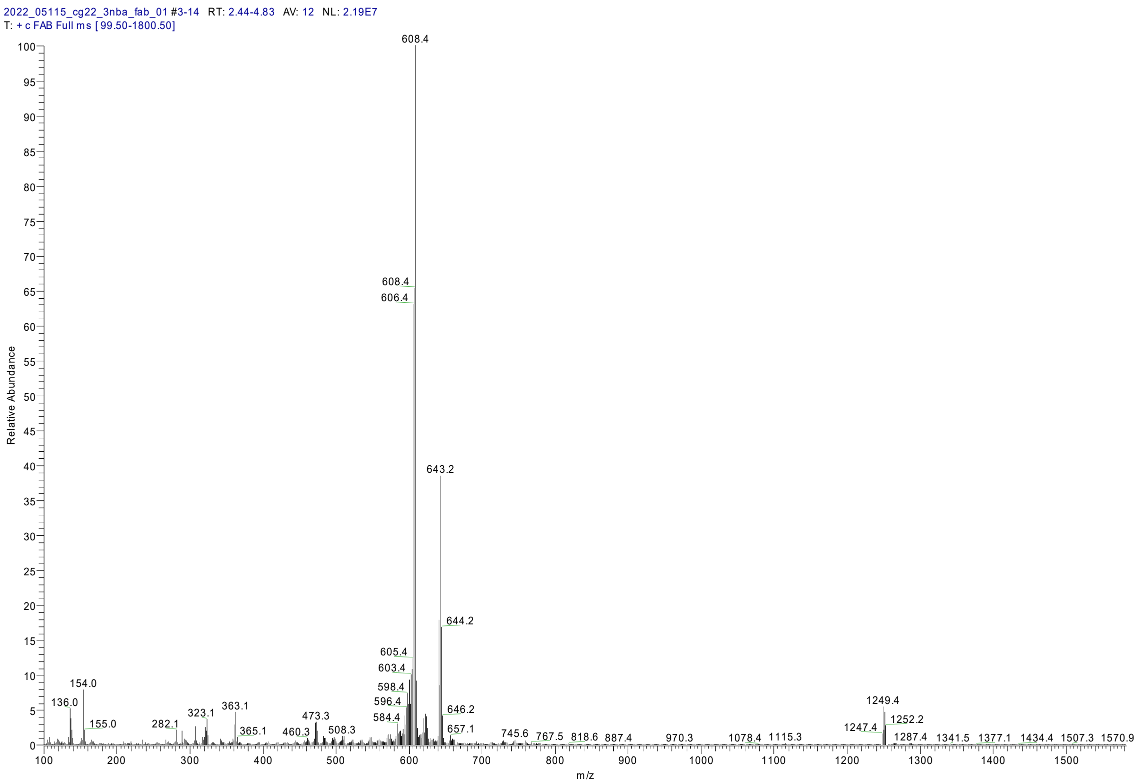

Figure S28. FAB+ mass of complex **1**.

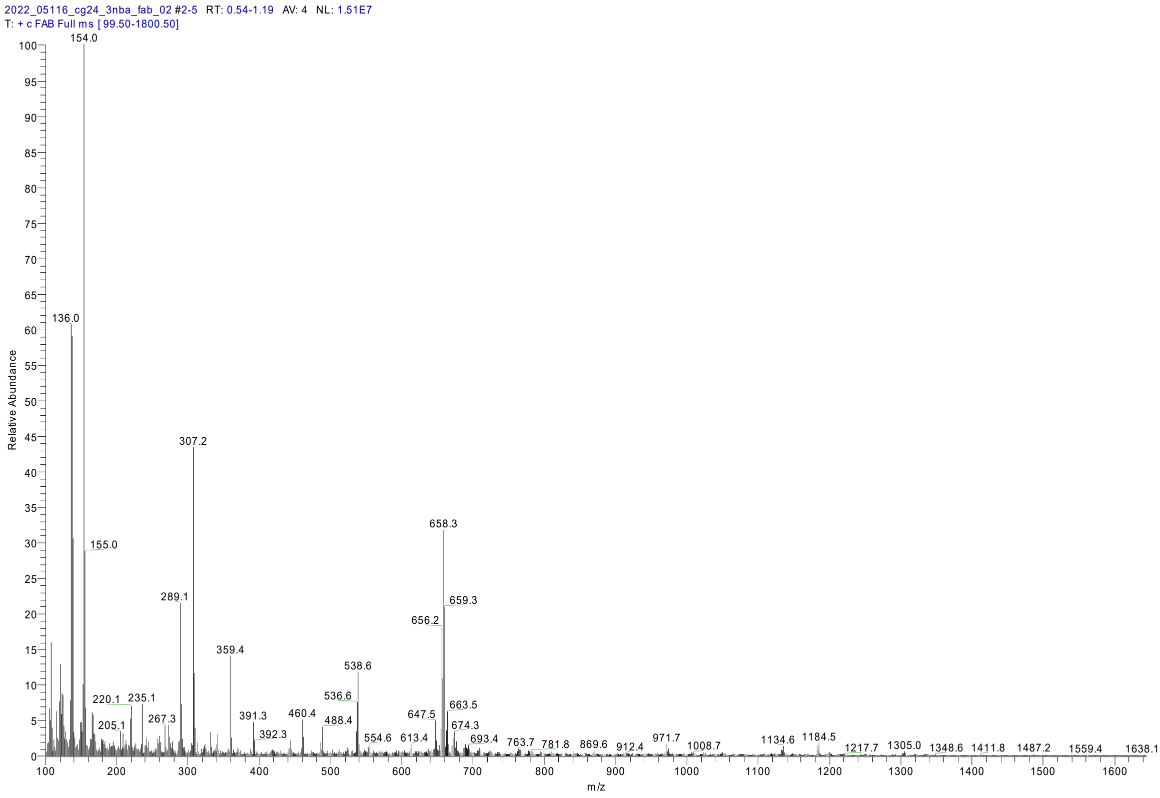

Figure S29. FAB+ mass of complex **2**.

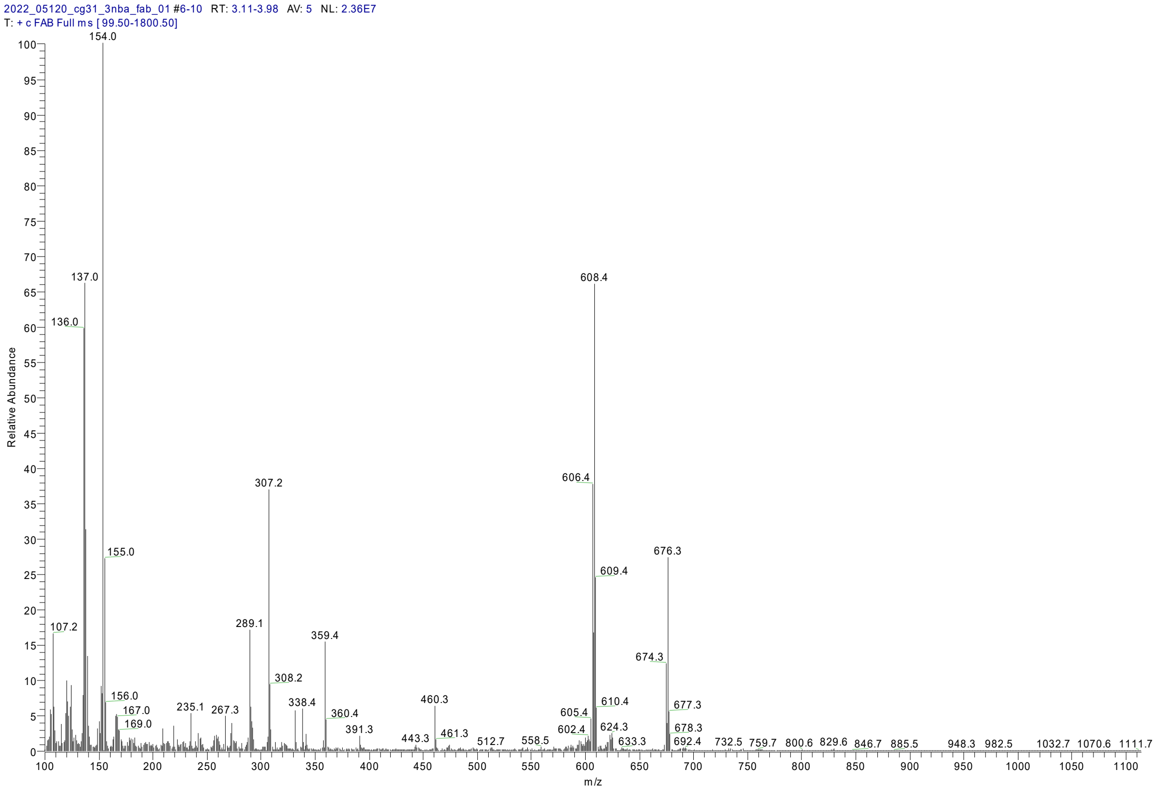

Figure S30. FAB+ mass of complex **3**.

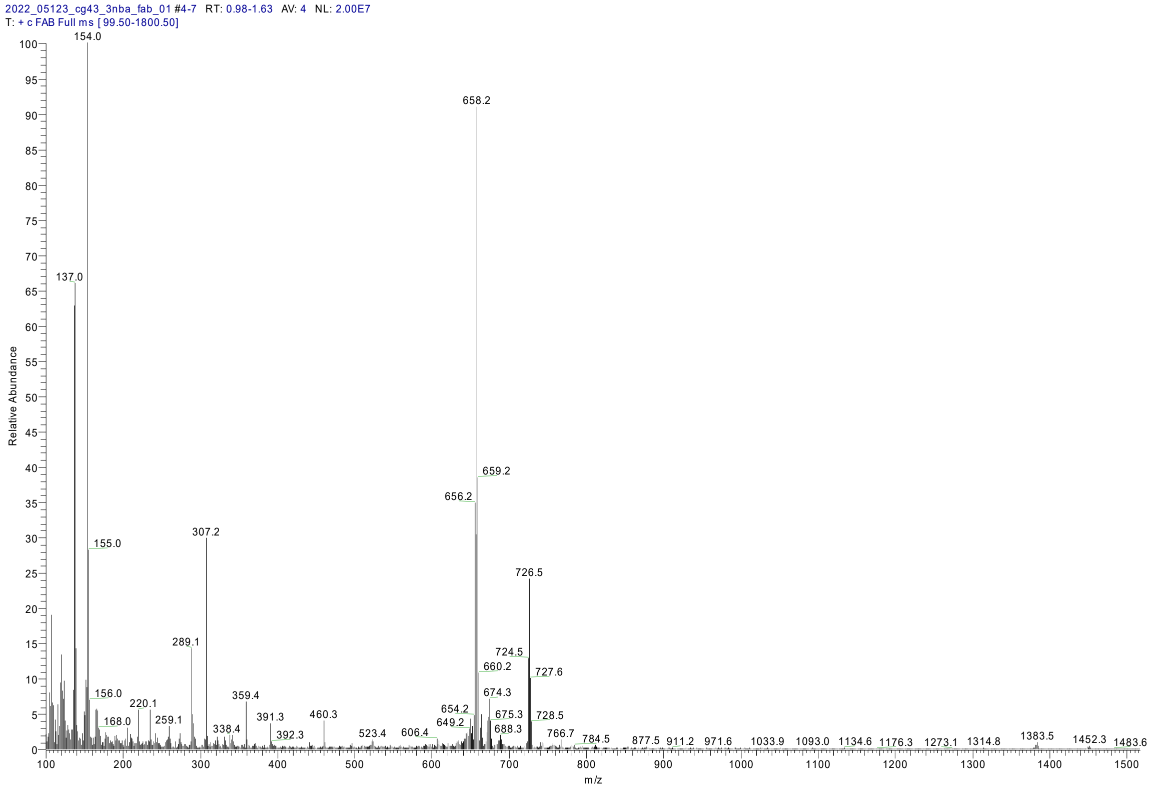

Figure S31. FAB+ mass of complex **4**.

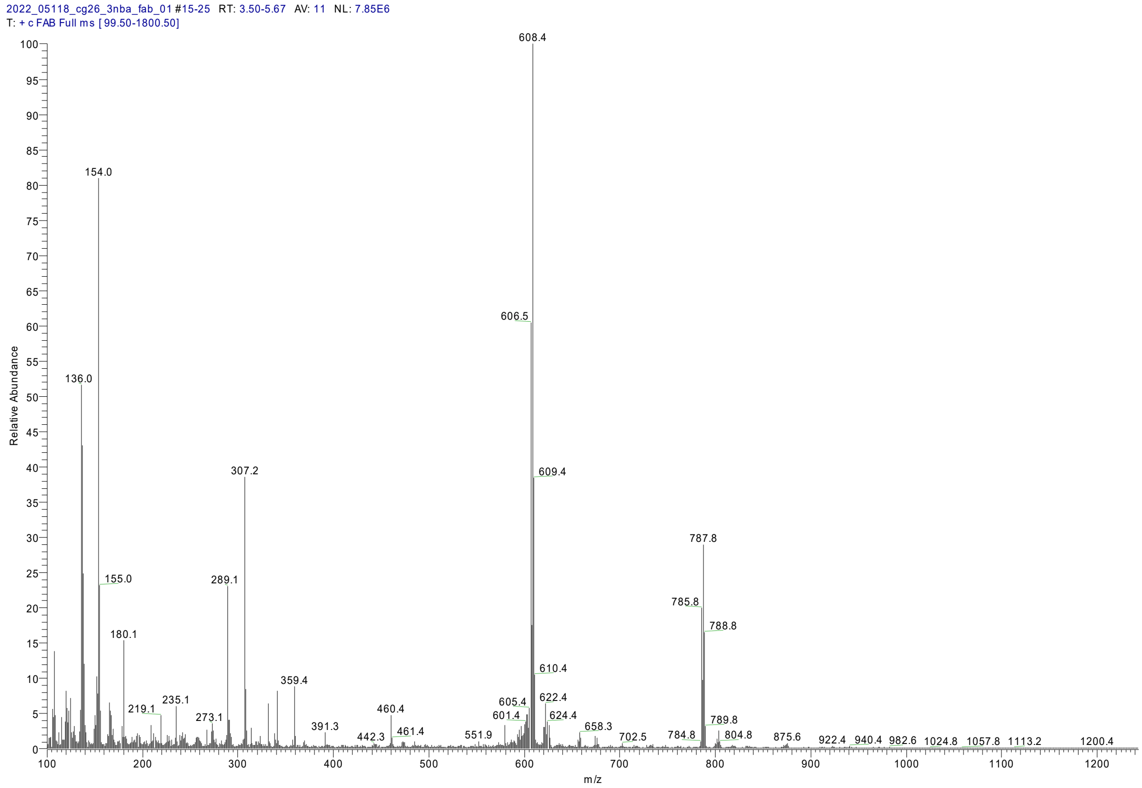

Figure S32. FAB+ mass of complex **5**.

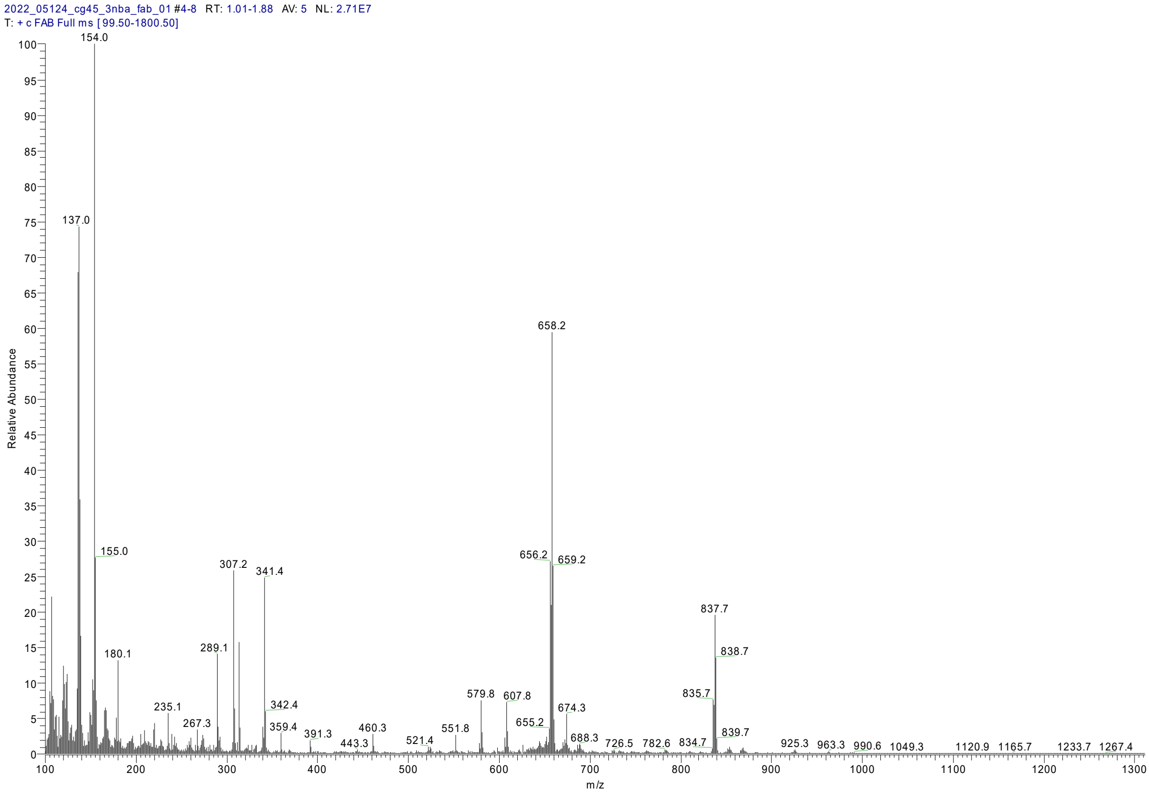

Figure S33. FAB+ mass of complex **6**.

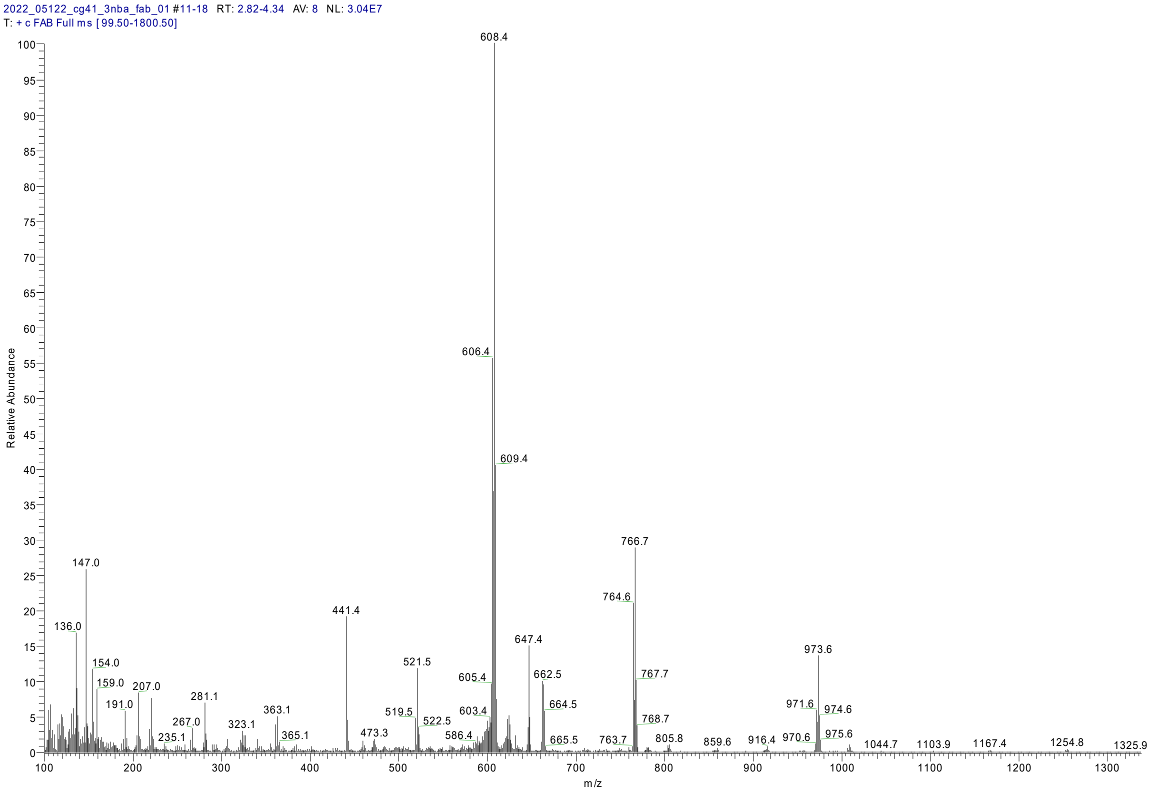

Figure S34. FAB+ mass of complex **7**.

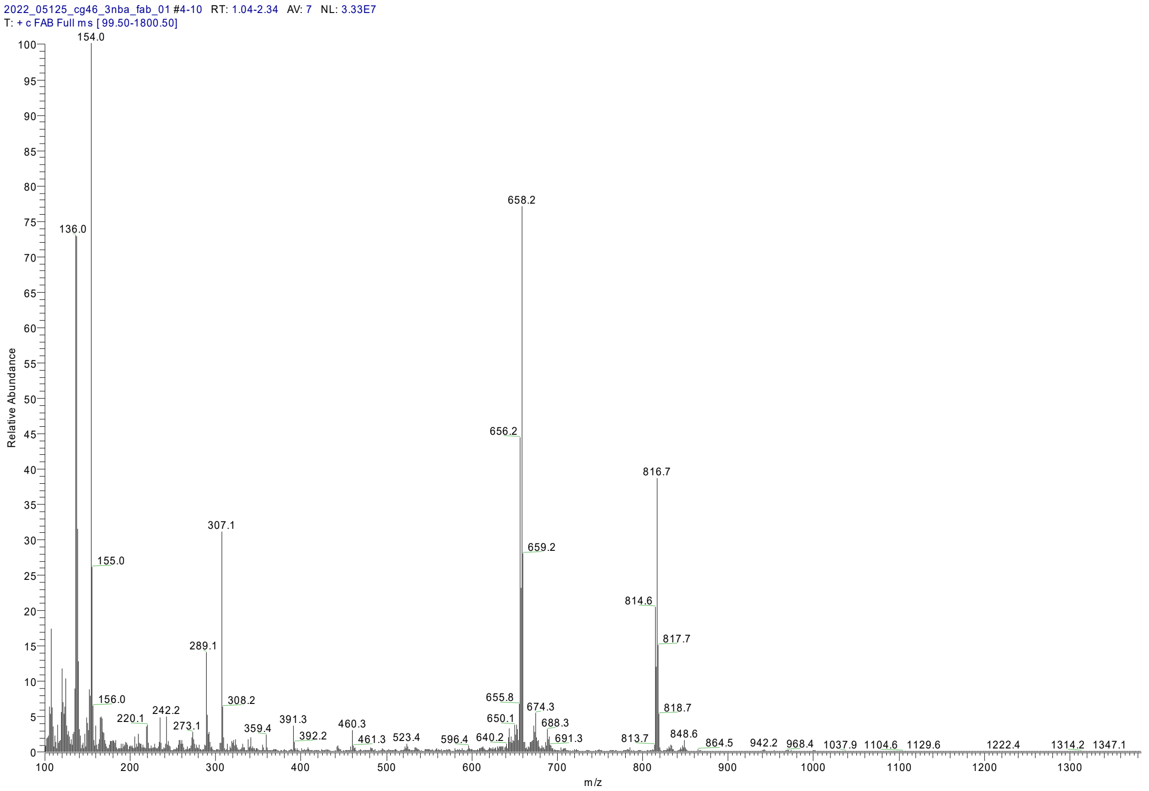

Figure S35. FAB+ mass of complex **8**.

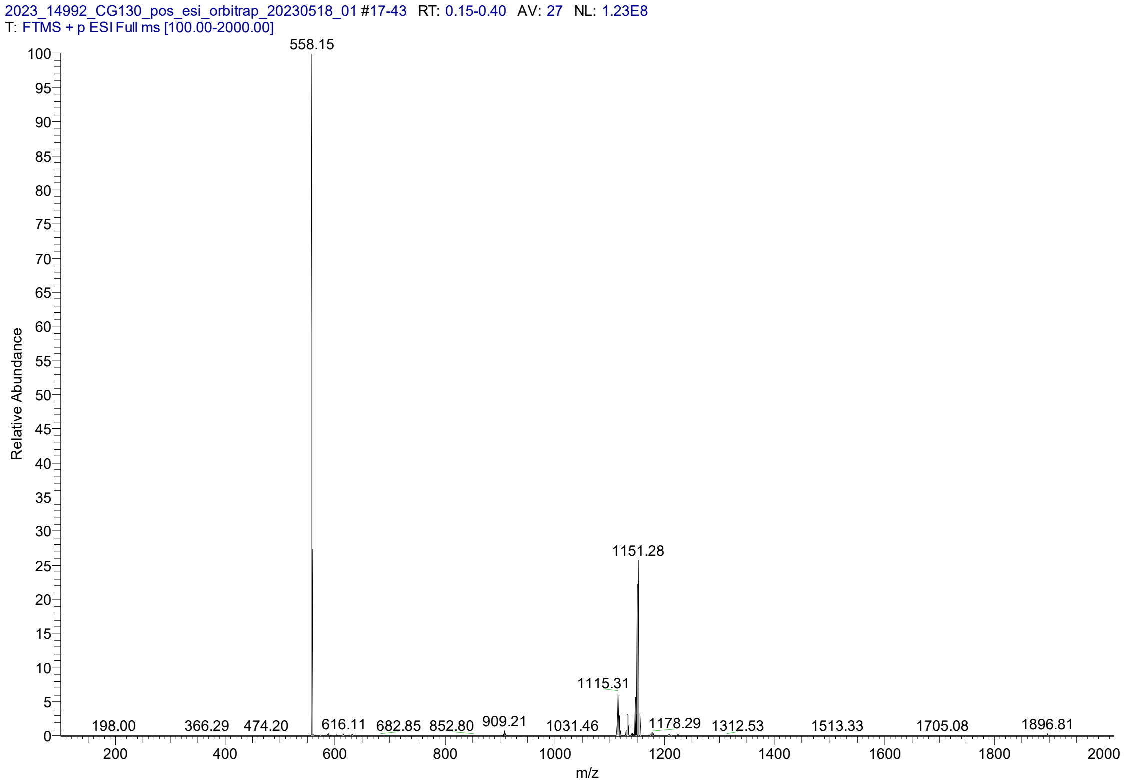

Figure S36. ESI+ mass of complex **9**.

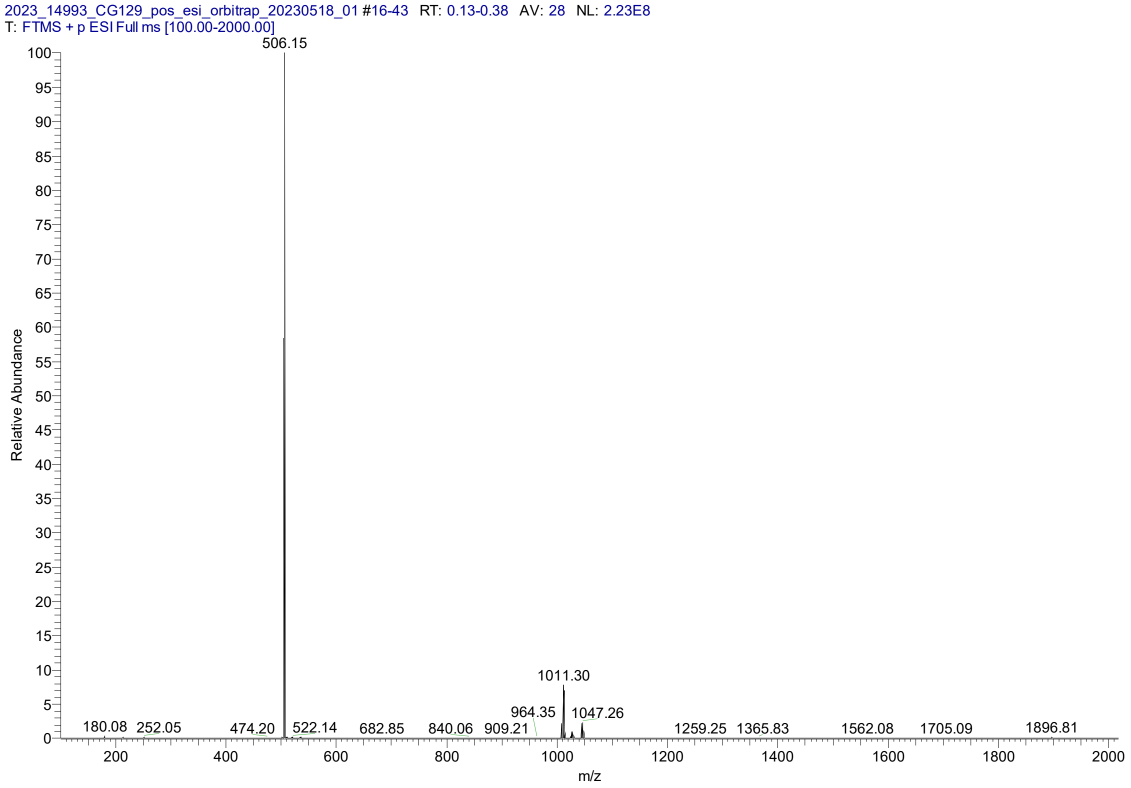

Figure S37. ESI+ mass of complex **10**.

HPLC traces

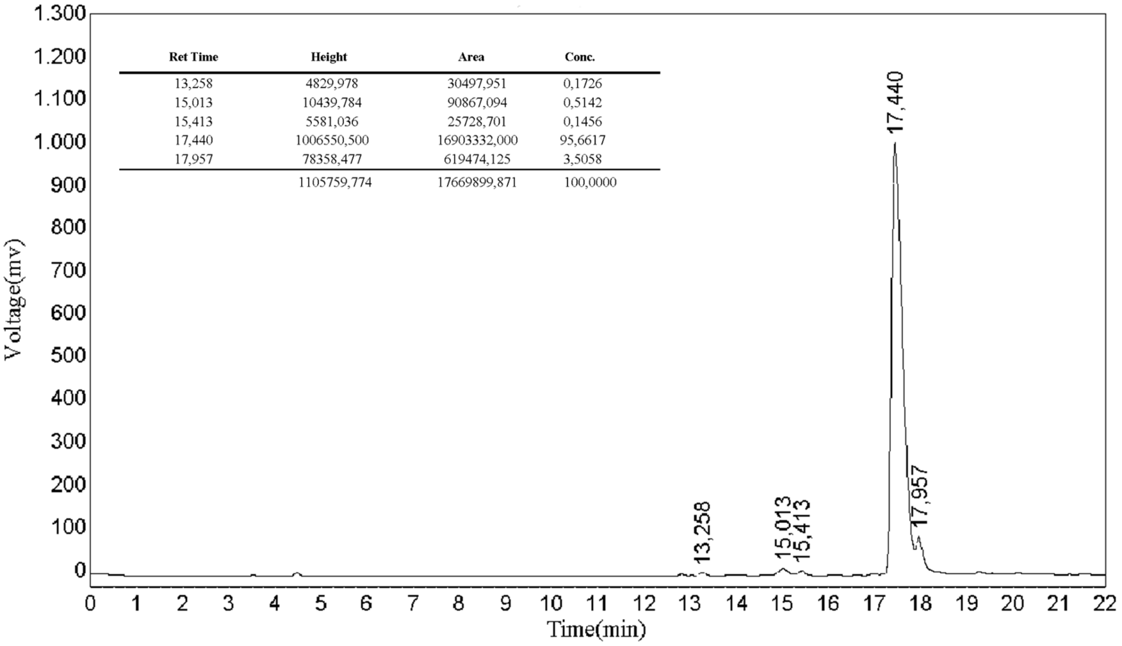

Ret Time	Height	Area	Conc.
13,258	4829,978	30497,951	0,1726
15,013	10439,784	90867,094	0,5142
15,413	5581,036	25728,701	0,1456
17,440	1006550,500	16903332,000	95,6617
17,957	78358,477	619474,125	3,5058
	1105759,774	17669899,871	100,0000

Figure S38. HPLC-UV chromatogram of complex **1**.

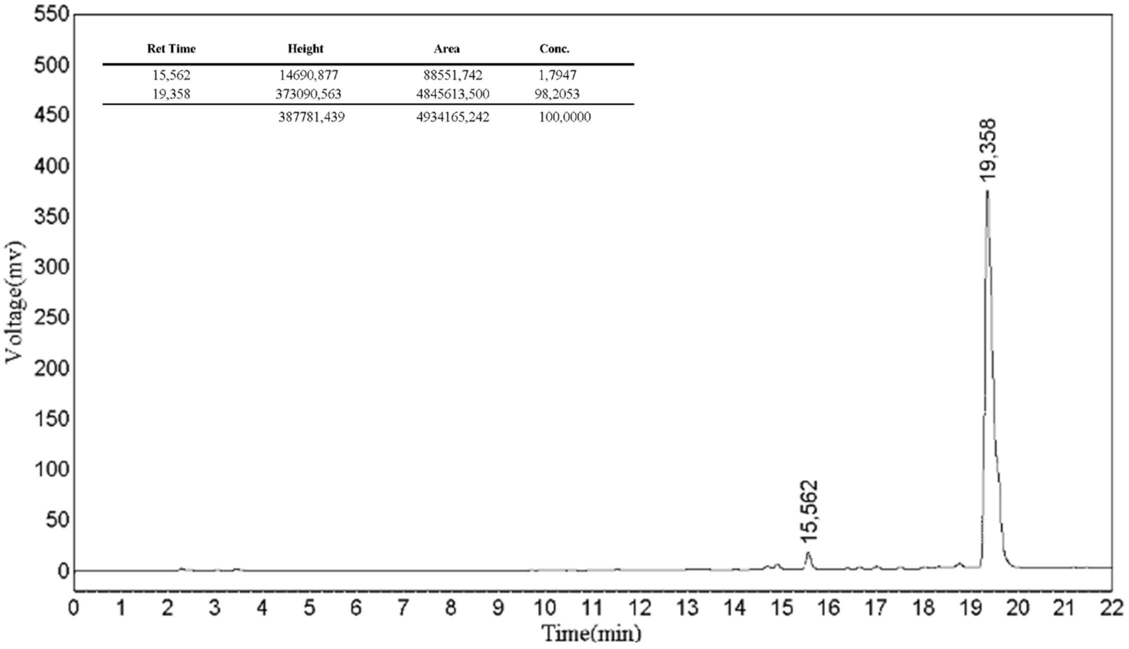

Ret Time	Height	Area	Conc.
15,562	14690,877	88551,742	1,7947
19,358	373090,563	4845613,500	98,2053
	387781,439	4934165,242	100,0000

Figure S39. HPLC-UV chromatogram of complex **2**.

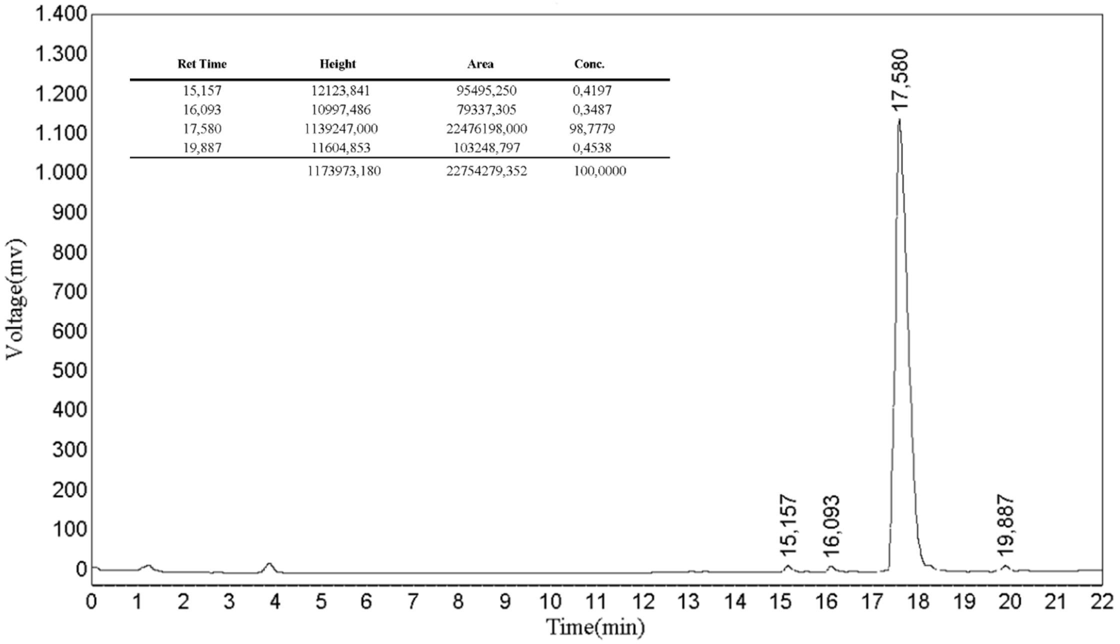

Ret Time	Height	Area	Conc.
15,157	12123,841	95495,250	0,4197
16,093	10997,486	79337,305	0,3487
17,580	1139247,000	22476198,000	98,7779
19,887	11604,853	103248,797	0,4538
	1173973,180	22754279,352	100,0000

Figure S40. HPLC-UV chromatogram of complex **3**.

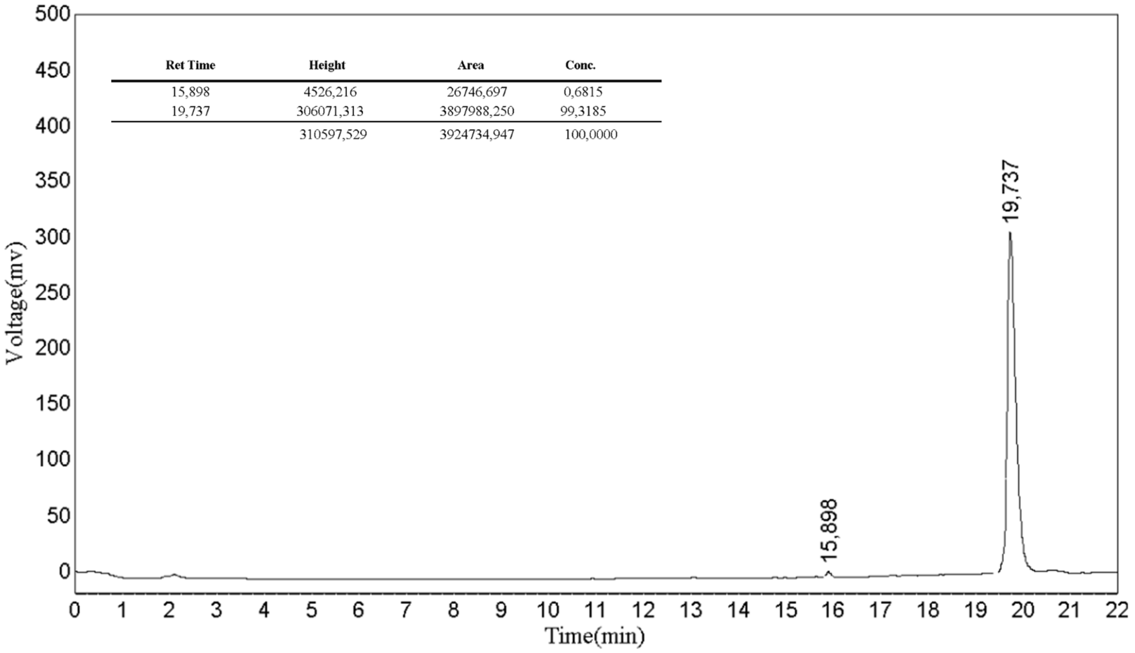

Ret Time	Height	Area	Conc.
15,898	4526,216	26746,697	0,6815
19,737	306071,313	3897988,250	99,3185
	310597,529	3924734,947	100,0000

Figure S41. HPLC-UV chromatogram of complex **4**.

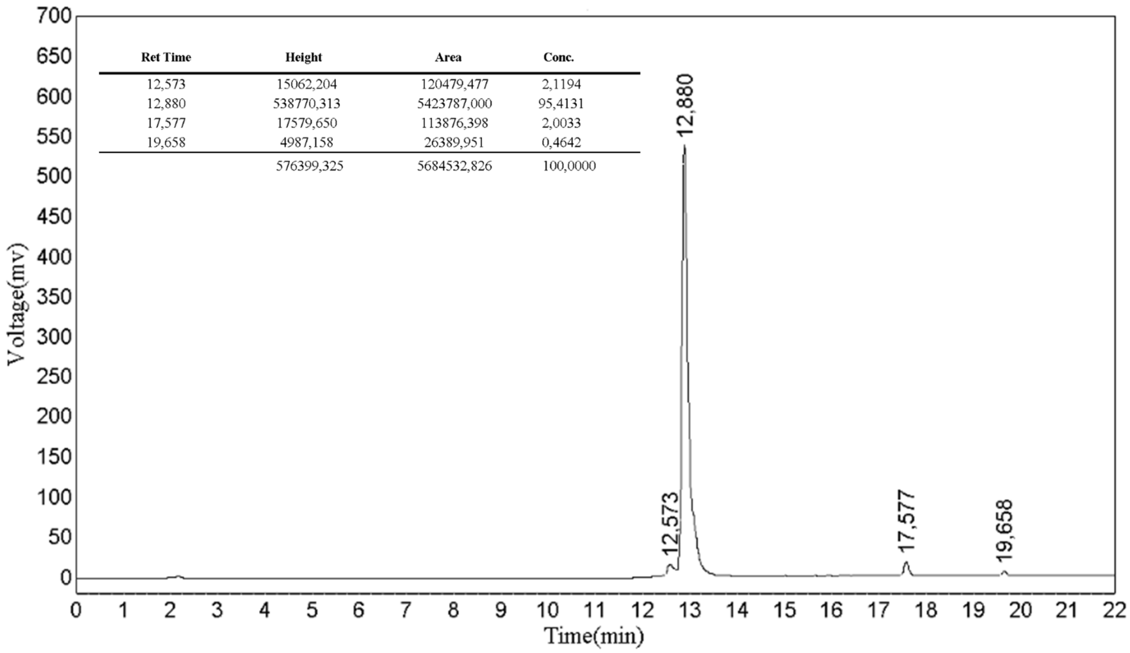

Figure S42. HPLC-UV chromatogram of complex **5**.

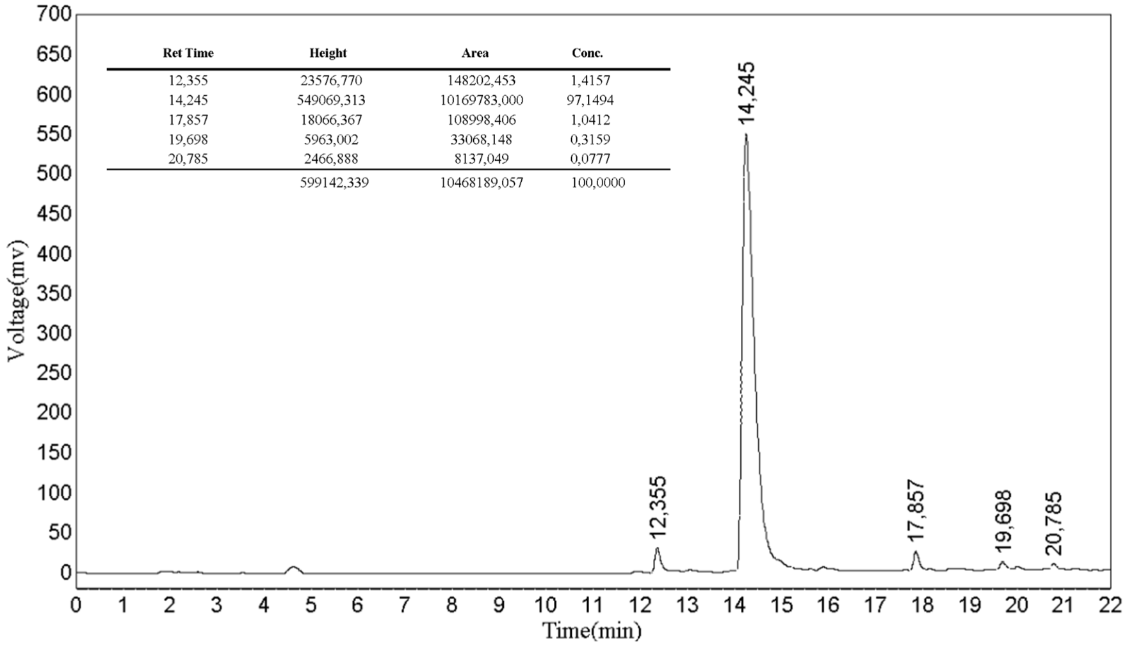

Figure S43. HPLC-UV chromatogram of complex **6**.

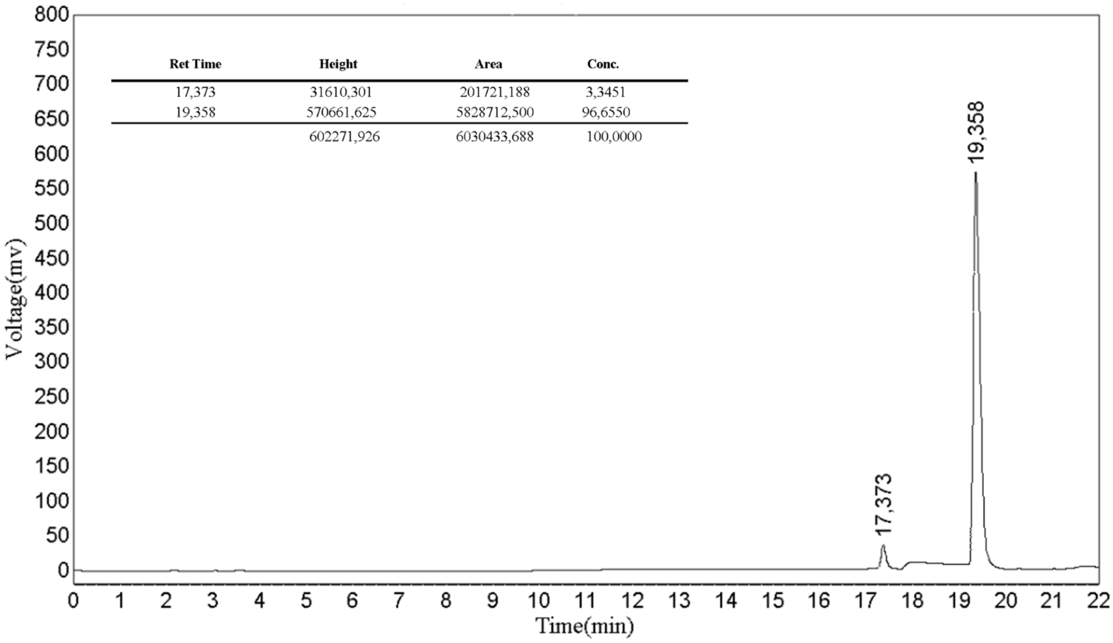

Ret Time	Height	Area	Conc.
17,373	31610,301	201721,188	3,3451
19,358	570661,625	5828712,500	96,6550
	602271,926	6030433,688	100,0000

Figure S44. HPLC-UV chromatogram of complex **7**.

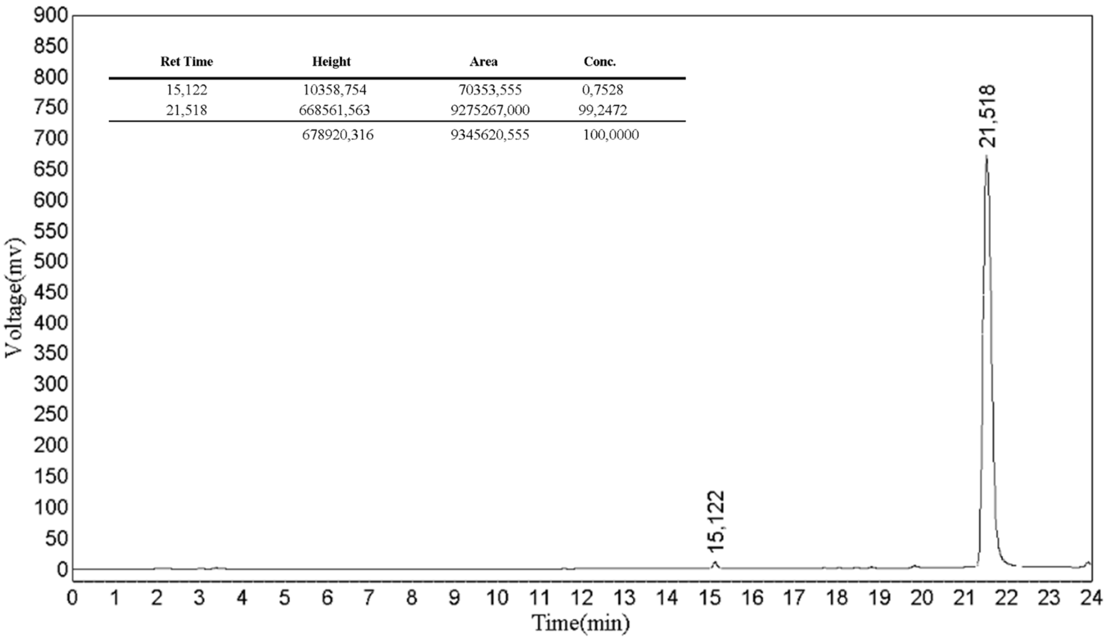

Ret Time	Height	Area	Conc.
15,122	10358,754	70353,555	0,7528
21,518	668561,563	9275267,000	99,2472
	678920,316	9345620,555	100,0000

Figure S45. HPLC-UV chromatogram of complex **8**.

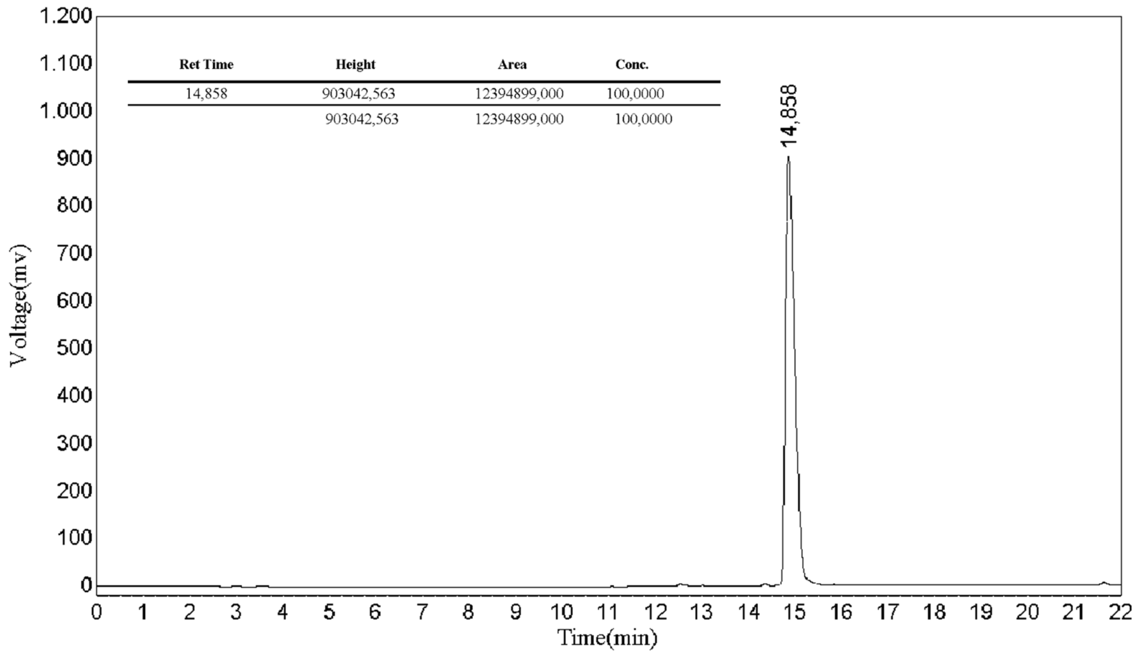

Figure S46. HPLC-UV chromatogram of complex **9**.

pK_a determination

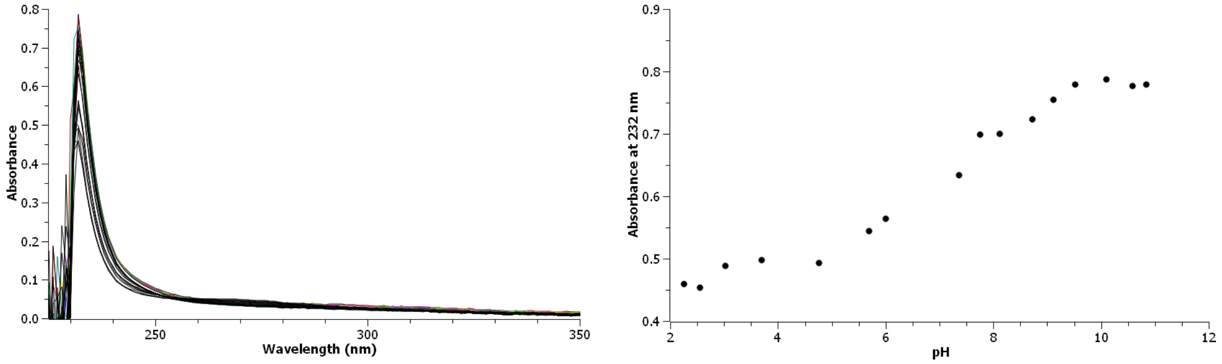

(a) (b)

Figure S47. Absorbance spectra of Imepip ligand recorded at different pH in H_2O/DMSO (98/2, v/v) (a) and pH effect on the ligand absorbance at 232 nm (b).

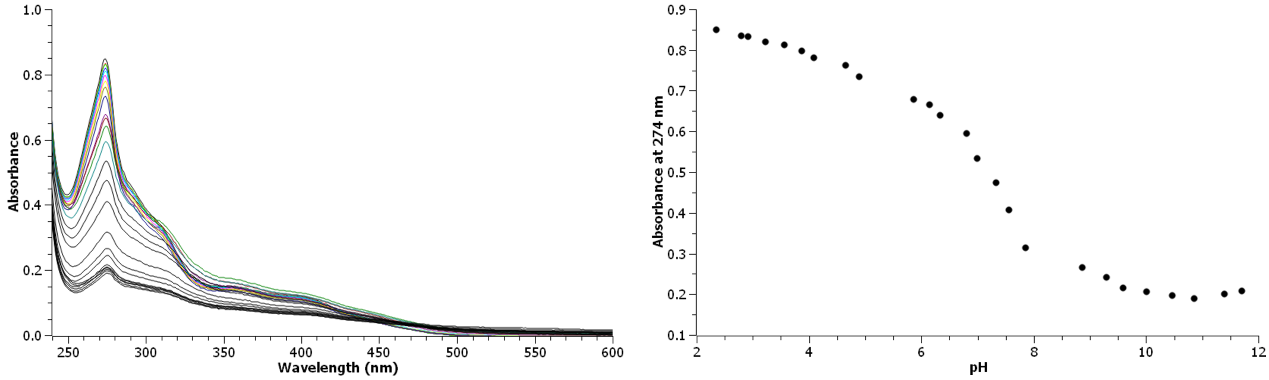

(a) (b)

Figure S48. Absorbance spectra of **5** recorded at different pH in H_2O/DMSO (98/2, v/v) (a) and pH effect on the ligand absorbance at 274 nm (b).

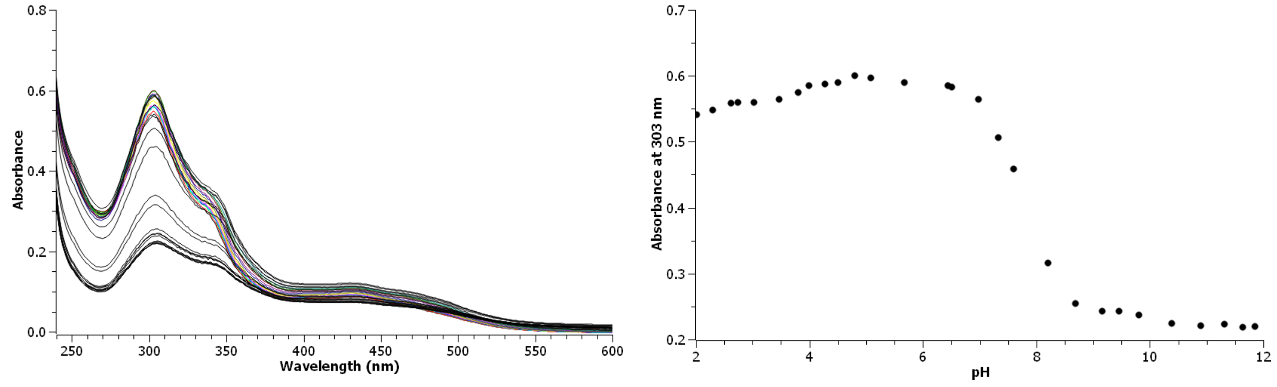

(a) (b)

Figure S49. Absorbance spectra of **6** recorded at different pH in H_2O/DMSO (98/2, v/v) (a) and pH effect on the ligand absorbance at 303 nm (b).

X-ray diffraction

Table S3. Crystal data and structure refinement for **1** and **6 × 0.75C$_3$H$_6$O** and **9 × 0.5C$_4$H$_{10}$O**.

	**1**	**6 × 0.75C$_3$H$_6$O**	**9 × 0.5C$_4$H$_{10}$O**
Empirical formula	$C_{29}H_{25}ClIrN_3$	$C_{45.25}H_{48.5}BF_4IrN_6O_{0.75}$	$C_{27}H_{28}ClIrN_3O_{0.5}$
Formula weight	643.17	967.41	630.17
Temperature (K)	293(2)	100(2)	296.15
Wavelength (Å)	0.71073	0.71073	0.71073
Crystal system	Triclinic	Monoclinic	Triclinic
Space group	P ī	P 2_1/n	P ī
a(Å)	7.4911(3)	12.1003(6)	8.581(2)
b(Å)	8.6903(4)	14.0997(6)	12.589(3)
c(Å)	18.3915(7)	25.9622(13)	12.635(3)
α(°)	97.442(2)	90	61.954(2)
β(°)	93.793(2)	97.923(2)	89.674(3)
γ(°)	97.365(2)	90	88.384(3)
Volume(Å^3)	1173.23(8)	4387.1(4)	1204.2(5)
Z	2	4	2
Density (calculated) (g/cm^3)	1.821	1.465	1.738
Absorption coefficient (mm^{-1})	5.828	3.100	5.677
F(000)	628	1944	618.0
Crystal size (mm^3)	0.15 x 0.07 x 0.01	0.27 x 0.10 x 0.10	0.11 × 0.06 × 0.05
Index ranges	-9 ≤ h ≤ 9<br>-10 ≤ k ≤ 10<br>0 ≤ l ≤ 22	-14 ≤ h ≤ 14<br>-16 ≤ k ≤ 16<br>-24 ≤ l ≤ 30	-10 ≤ h ≤ 9<br>-14 ≤ k ≤ 15<br>-15 ≤ l ≤ 15
Reflections collected	4814	101372	7581
Independent reflections	4814<br>[R(int) = 0.0377]	7703<br>[R(int) = 0.0331]	4754<br>[R_{int} = 0.0317]
Data / restraints / parameters	4814 / 0 / 313	7703 / 84 / 587	4754/19/310
Goodness-of-fit on F^2	1.060	1.148	1.031
Final R indices [$I>2\sigma(I)$]	R1 = 0.0275<br>wR2 = 0.0614	R1 = 0.0319<br>wR2 = 0.0807	R_1 = 0.0418<br>wR_2 = 0.0688
Largest diff. peak / hole, e.Å^{-3}	0.761 / -1.115	0.845 / -1.233	0.82 / -1.14

[a] $R = \Sigma||F_o|-|F_c|/\Sigma|F_o|$. [b] $wR = \{\Sigma w(F_o^2-F_c^2)^2/\Sigma w(F_o^2)^2\}^{1/2}$. [c] GOF = $\{\Sigma [w((F_o^2-F_c^2)^2)/(n-p)\}^{1/2}$, where n = number of reflections and p = total number of parameters refined.

Table S4. Selected bond distances (Å) and angles (°) for complexes **1**, **6** × **0.75**C_3H_6O and **9** × **0.5**$C_4H_{10}O$.

Comp.	C^N				L		Ir-Cp* (centr.)	Bite angle (C-Ir-N)
**1**	Ir(1)-C(1)	2.076(5)	Ir(1)-N(1)	2.079(5)	Ir(1)-Cl(1)	2.395(1)	1.826	79.0(2)
**6**	Ir(1)-C(11)	2.083(4)	Ir(1)-N(4)	2.091(4)	Ir(1)-N(1)	2.085(3)	1.822	78.8(2)
**9**	Ir(1)-C(7)	2.052(6)	Ir(1)-N(1)	2.082(5)	Ir(1)-Cl(1)	2.392(2)	1.821	78.6(2)

Stability and photostability studies

The evolution of **1** and **2** in the dark was studied in a DMSO-d_6/D_2O (9/1) solvent mixture (Figures S50-S52 and Table S5). The appearance of resonances due to a new species was observed in both cases. The new compounds, named **1w** and **2w**, were already present in the freshly prepared solutions but the evolution was clearly faster in the case of **1**. After 30 minutes **1w** was nearly the only compound observed while in the case of **2w** a small amount of the starting chlorido derivative **2** was still visible after 24 hours. We propose that the derivatives formed are the aqua adducts [Cp*Ir(C^N)(D_2O)]$^+$. The formation of the DMSO adducts was ruled out by analysis of the evolution of DMSO-d_6 solutions of **1** and **2** (Figures S53-S56 and Tables S6-S7). In both cases, the chemical shifts were very similar to those of **1w** and **2w** but the transformation occurred to a much lesser extent, thus highlighting the influence of the D_2O concentration (traces of water in the DMSO-d_6 used).

When 100 equivalents of *n*-Bu_4NCl were added to a solution containing **1w**, a 2% of **1** was formed at room temperature after 4 days, indicating the high kinetic stability of **1w**. When the solution was heated at 60 °C for 15 minutes, approximately 15% of the chloride complex **1** was observed.

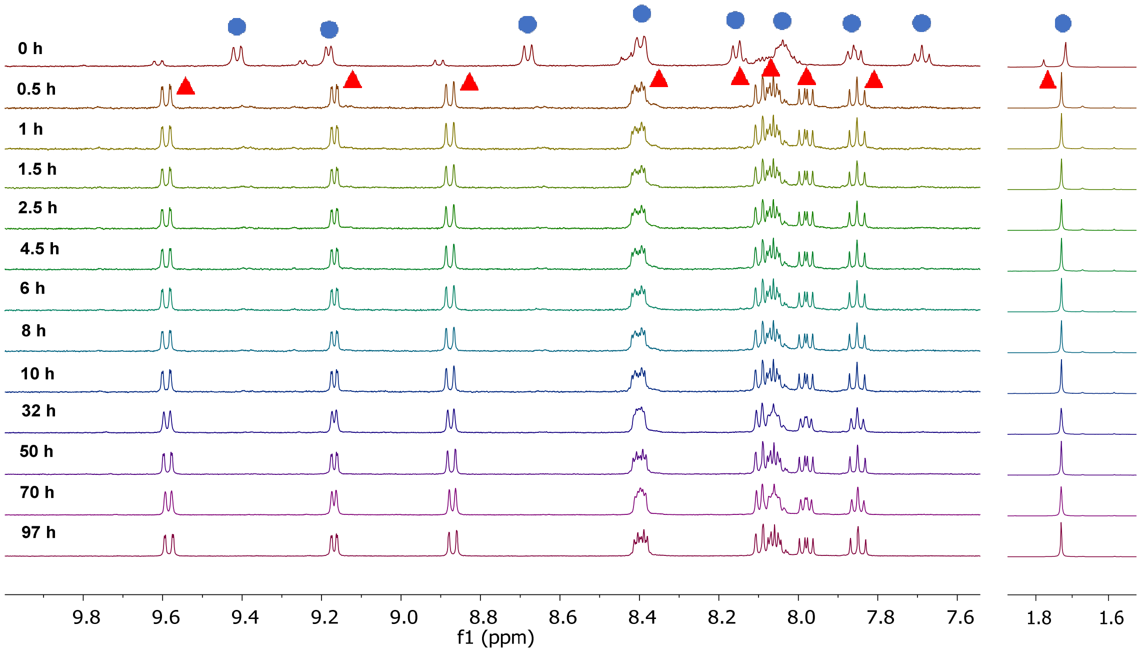

Figure S50. ^{1}H NMR of complex **1** in DMSO-d_6/D_2O (9/1) at different times in the dark. The spectrum indicated with 0 h is complex **1** (•) in DMSO-d_6. Red triangles (▲) correspond to the aqua-complex **1w** in DMSO-d_6/D_2O (9/1).

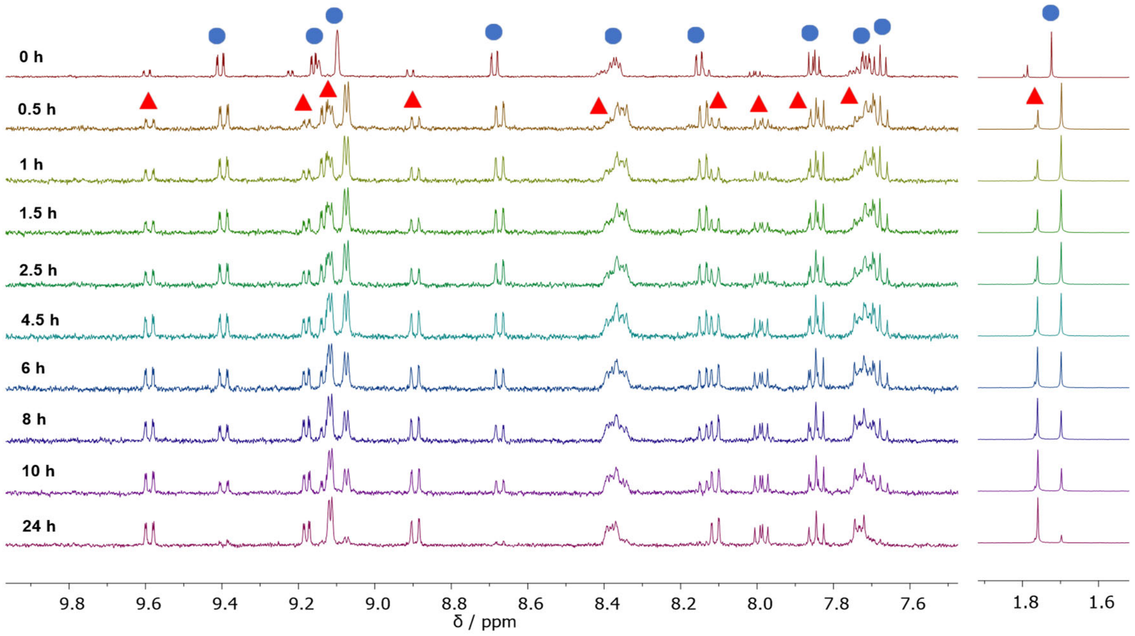

Figure S51. ^{1}H NMR of complex **2** in DMSO-d_6/D_2O (9/1) at different times in the dark. The spectrum indicated with 0 h is complex **1** (•) in DMSO-d_6. Red triangles (▲) correspond to the aqua-complex **2w** in DMSO-d_6/D_2O (9/1). See Table S5 and Figure S52 for the relative concentrations of products.

Table S5. Percentage of **1w** or **2w** formed from **1** or **2**, respectively, in DMSO-d_6/D_2O (9/1) at different times in the dark (see representation of the data in Figure S52).

Time (h)	1w	2w	Time (h)	1w	2w
**0**	0	0	**6**	100	50
**0.2**	-	10	**8**	100	55
**0.5**	96	20	**10**	100	63
**1**	100	28	**24**	100	95
**1.5**	100	33	**32**	100	100
**2.5**	100	37	**48**	100	100
**4.5**	100	47			

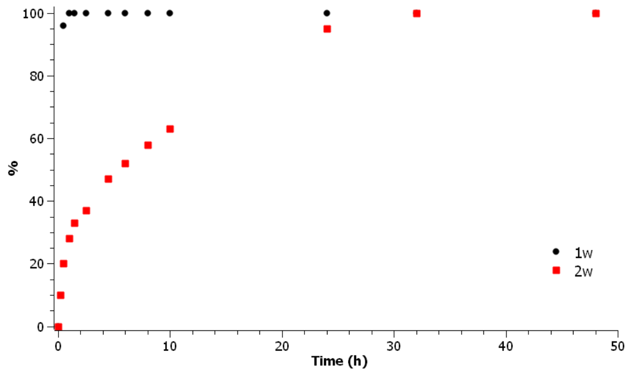

Figure S52. Formation with time of **1w** from **1** and **2w** from **2** as followed by ^{1}H NMR in DMSO-d_6/D_2O (9/1).

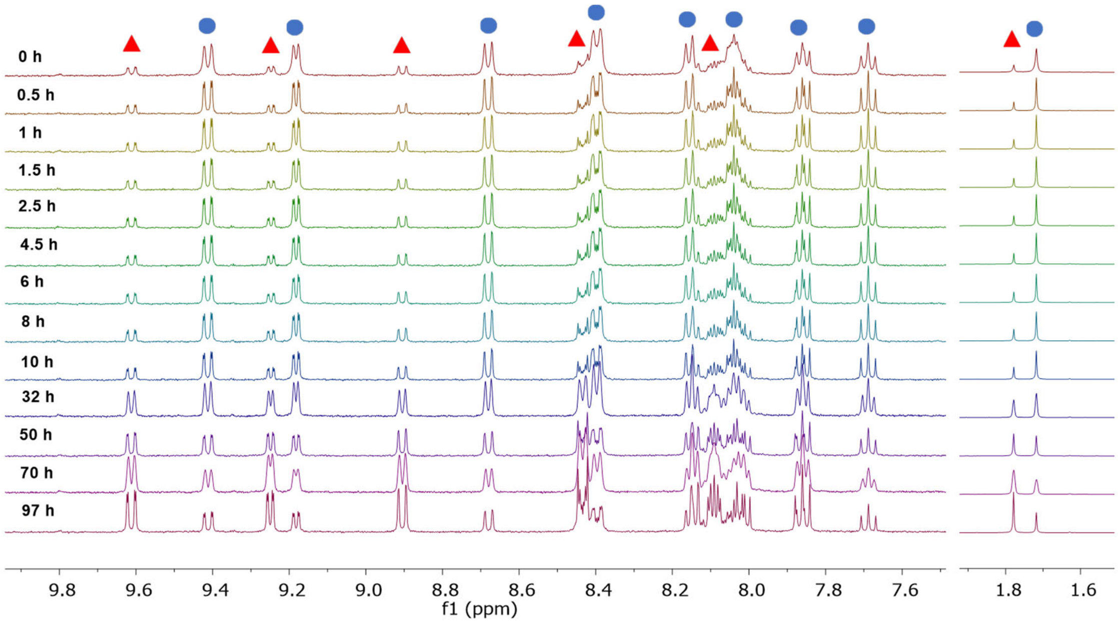

Figure S53. ^{1}H NMR of complex **1** in DMSO-d_6 at different times in the dark. Blue signals (•) correspond to complex **1**. Red triangles (▲) correspond to the aquation product, **1w**. See Table S6 and Figure S54 of relative concentrations of **1** and **1w**.

Table S6. Percentage of **1** and **1w**, formed from **1** in DMSO-d_6 or DMSO-d_6/D_2O (9/1) at different times in the dark (see representation of the data in Figure S54).

	DMSO		DMSO/D_2O			DMSO		DMSO/D_2O	
**Time (h)**	**1**	**1w**	**1**	**1w**	**Time (h)**	**1**	**1w**	**1**	**1w**
**0**	100	0	100	0	**8**	69	31	0	100
**0.5**	78	22	4	96	**10**	68	32	0	100
**1**	76	24	0.5	99.5	**32**	57	43	0	100
**1.5**	75	25	0	100	**50**	49	51	0	100
**2.5**	72	28	0	100	**70**	40	60	0	100
**4.5**	71	29	0	100	**97**	35	65	0	100
**6**	70	30	0	100					

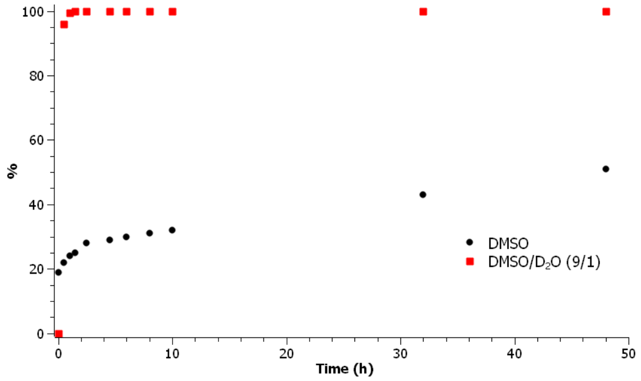

Figure S54. Formation with time of **1w** from **1** as followed by ^{1}H NMR in DMSO-d_6/D_2O (9/1) or DMSO-d_6.

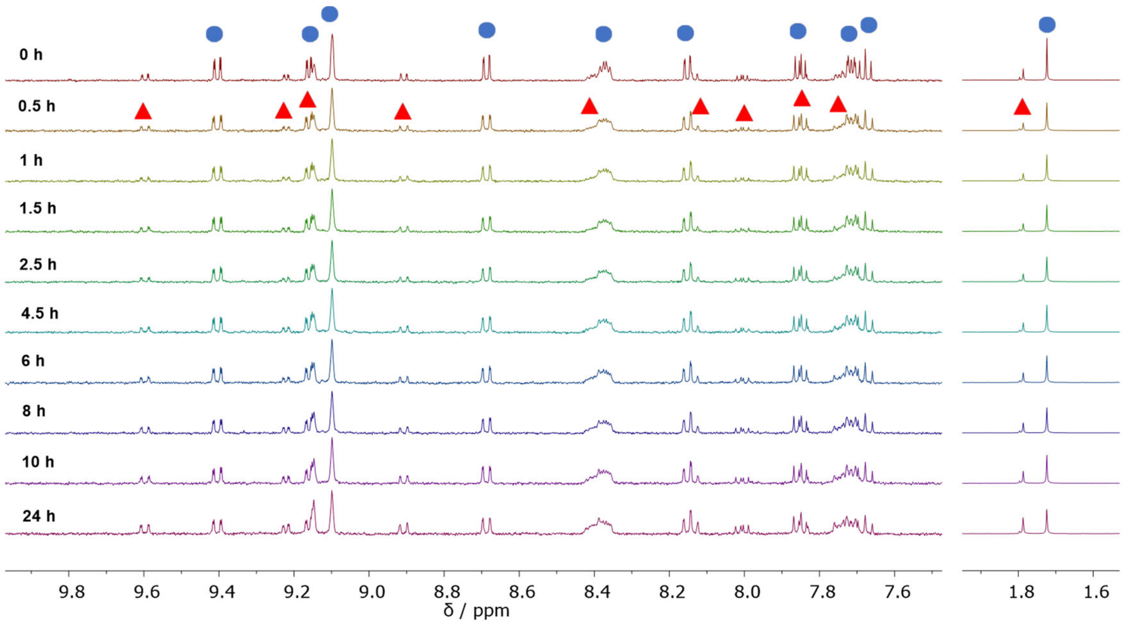

Figure S55. ^{1}H NMR of complex **2** in DMSO-d_6 at different times in the dark. Blue signals (•) correspond to complex **2**. Red triangles (▲) correspond to the aquation product, **2w**. See Table S7 and Figure S56 of relative concentrations of **2** and **2w**.

Table S7. Percentage of **2** and **2w**, formed from **2** in DMSO-d_6 or DMSO-d_6/D_2O (9/1) at different times in the dark (see representation of the data in Figure S56).

	DMSO		DMSO/D_2O			DMSO		DMSO/D_2O	
**Time (h)**	**2**	**2w**	**2**	**2w**	**Time (h)**	**2**	**2w**	**2**	**2w**
**0**	100	0	100	0	**6**	70	30	48	52
**0.5**	78	22	80	20	**8**	69	31	42	58
**1**	76	24	72	28	**10**	68	32	37	63
**1.5**	75	25	67	33	**24**	58	42	5	95
**2.5**	72	28	63	37	**32**	53	47	0	100
**4.5**	71	29	53	47	**48**	48	52	0	100

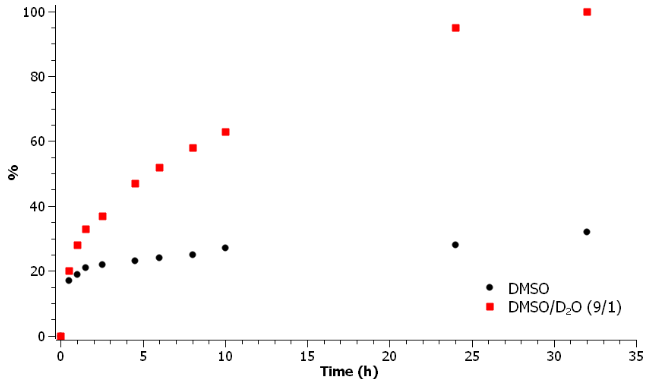

Figure S56. Formation with time of **2w** from **2** as followed by ^{1}H NMR in DMSO-d_6/D_2O (9/1) or DMSO-d_6.

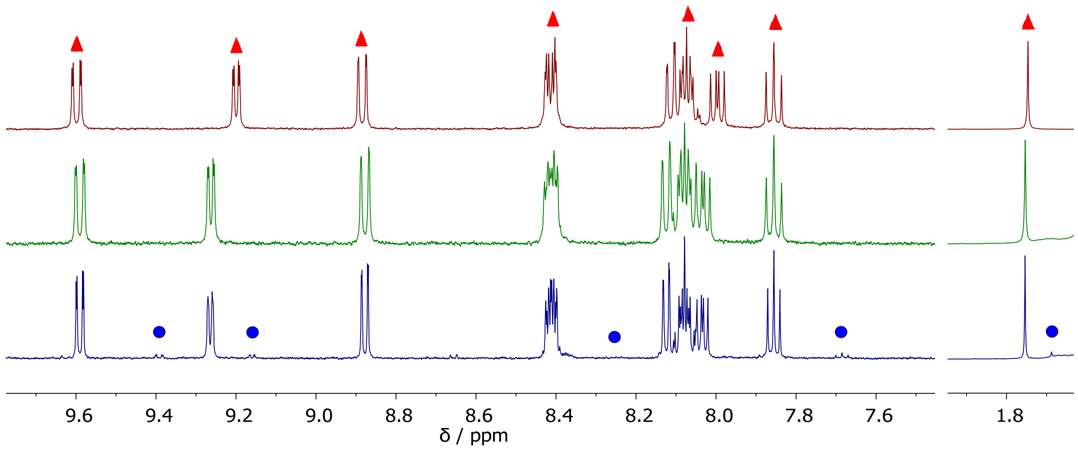

Figure S57. Evolution of **1w** in DMSO/D_2O (9/1, v/v) with 300 mM of *n*-Bu_4NCl (100 equivalents) at t = 0 min (red), t = 15 min (green) and t = 4 days (blue) at room temperature. Red triangles (▲) correspond to the aquation product, **1w**. Blue signals (•) correspond to complex **1**.

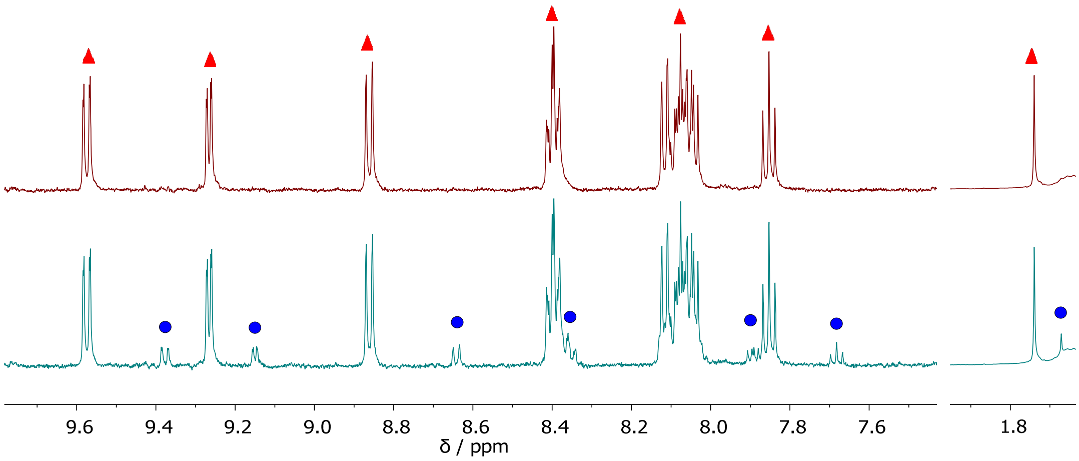

Figure S58. Evolution of **1w** in DMSO/D_2O (9/1, v/v) with 300 mM of *n*-Bu_4NCl (100 equivalents) at t = 0 min (red) and t = 15 min (blue) at 60 °C. Red triangles (▲) correspond to the aquation product, **1w**. Blue signals (•) correspond to complex **1**.

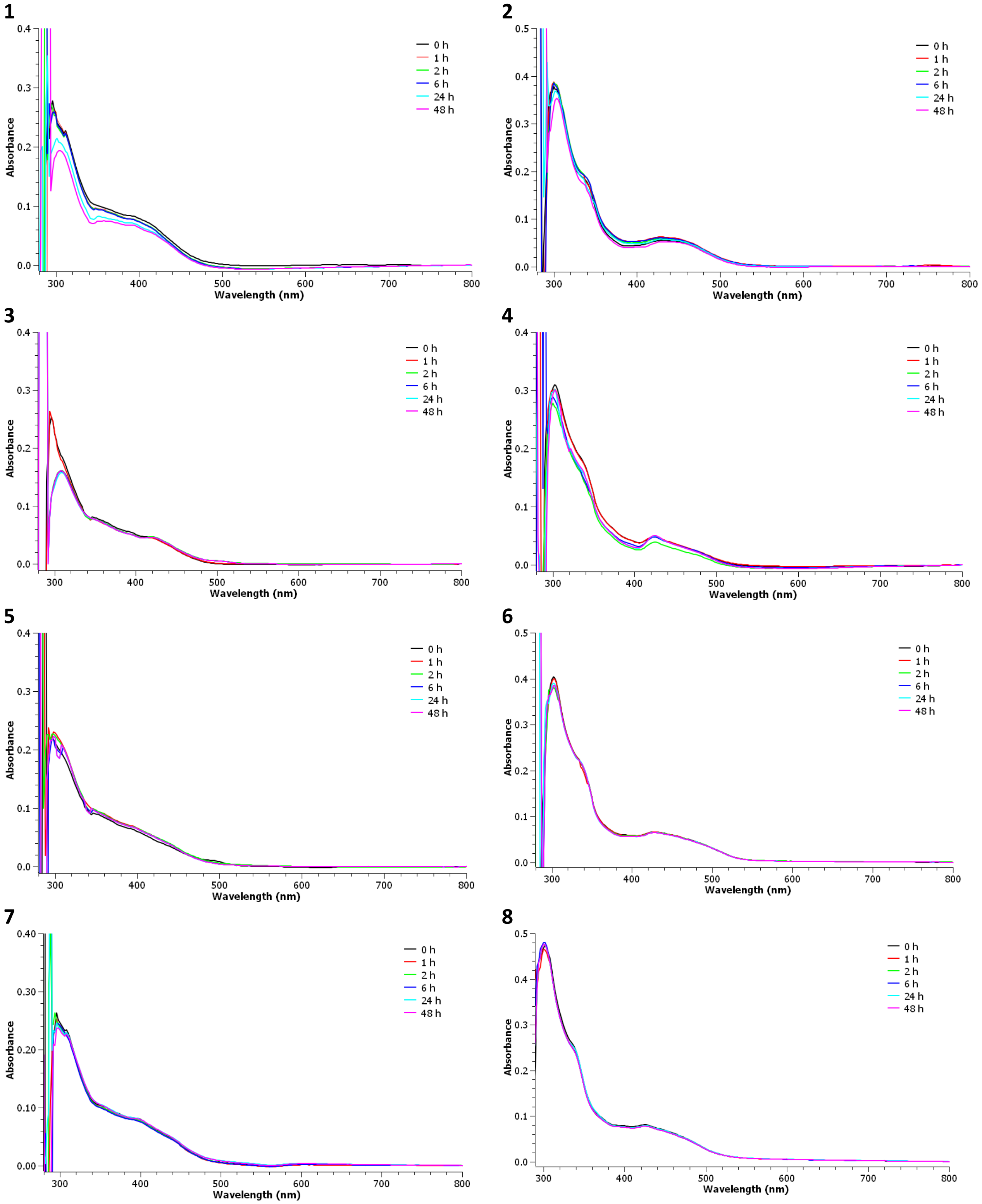

Figure S59. UV-vis spectra of complexes **1-8** in DMEM (1% of MeOH) at different times in the dark at 1.0×10^{-5} M.

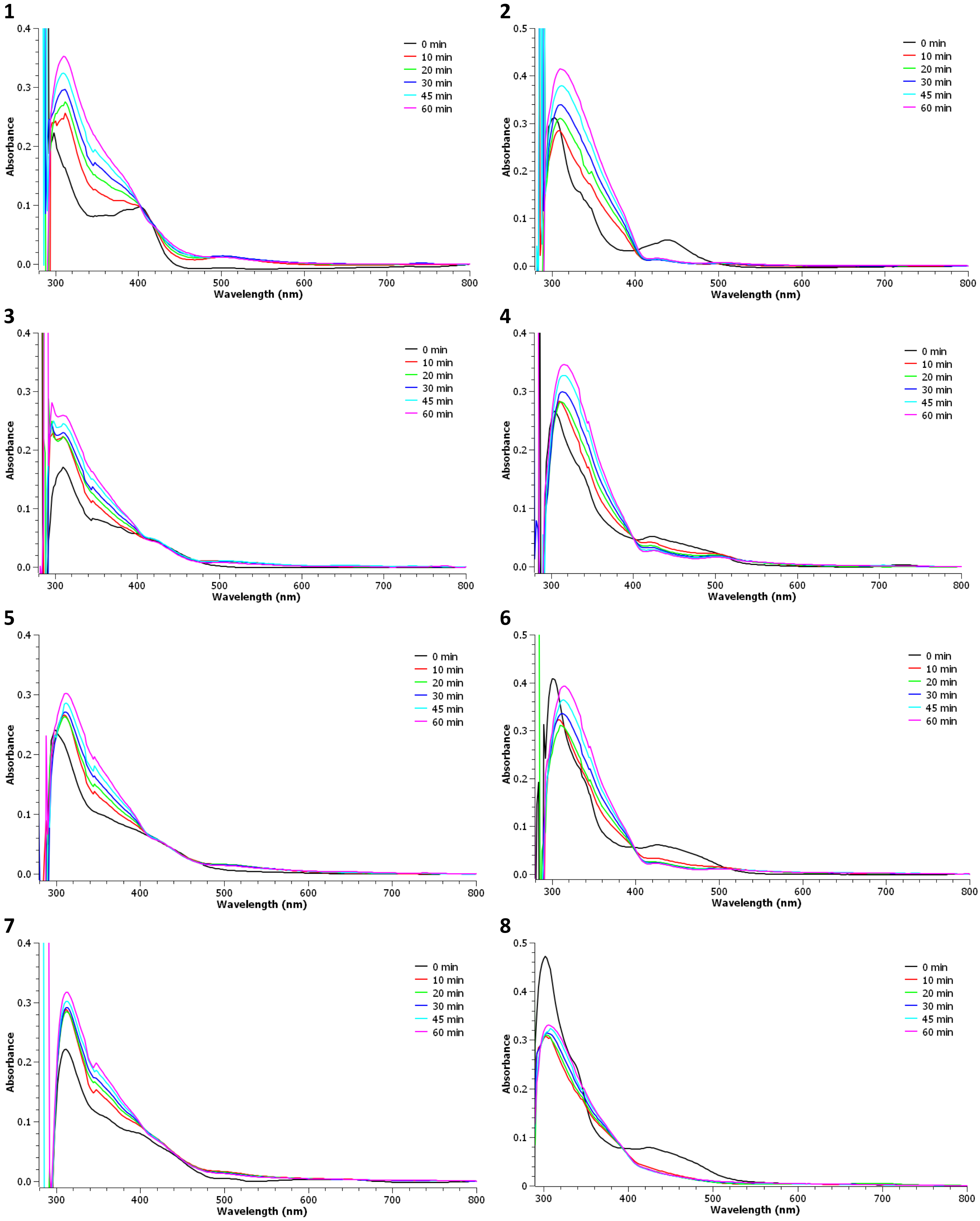

Figure S60. UV-vis spectra of complexes **1-8** in DMEM (1% of MeOH) at different times under blue light irradiation (470 nm) at 1.0×10^{-5} M.

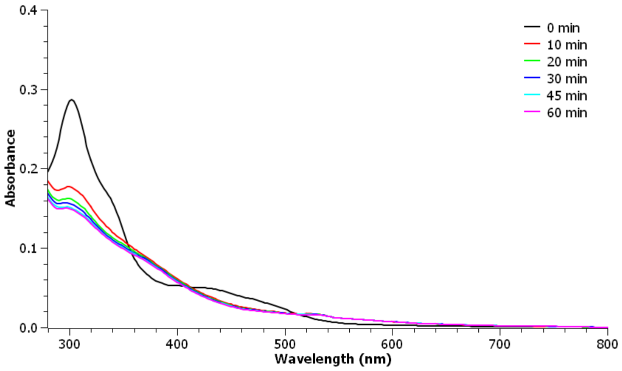

Figure S61. UV-vis spectra of complex **6** in water at different times under blue light irradiation (470 nm) at 1.0×10^{-5} M.

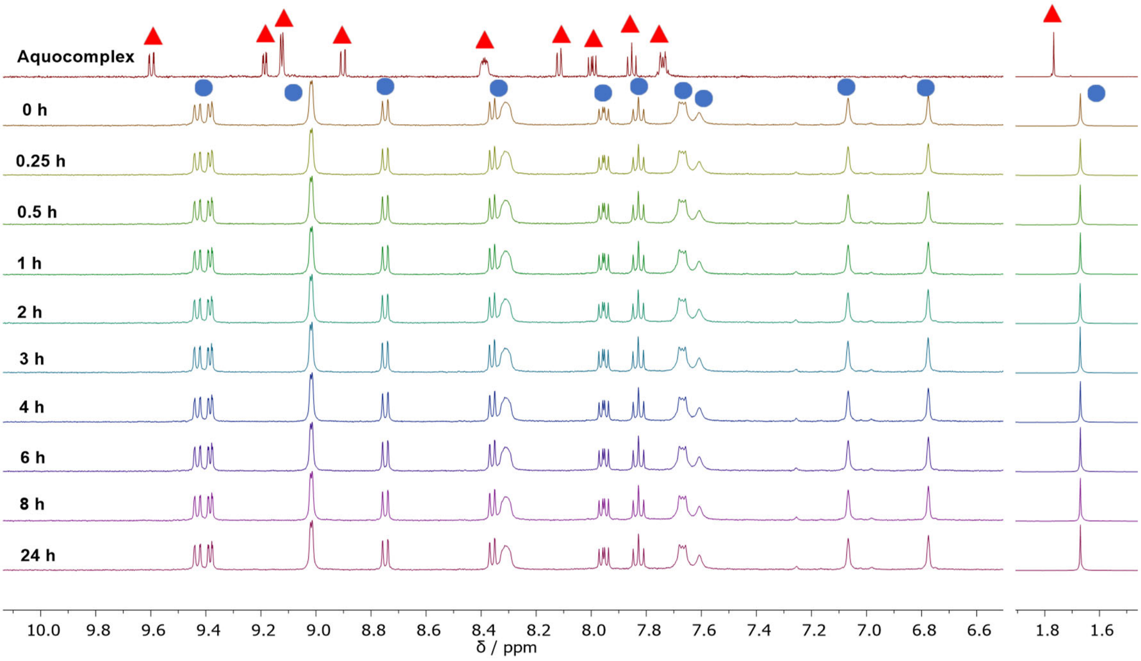

Figure S62. ^{1}H NMR of complex **6** in DMSO-d_6/D_2O (9/1) at different times in the dark. Blue signals (•) correspond to complex **6**. Red triangles correspond to the aquocomplex. The spectrum of the aquocomplex in the same solvent mixture is also included (red triangles, ▲).

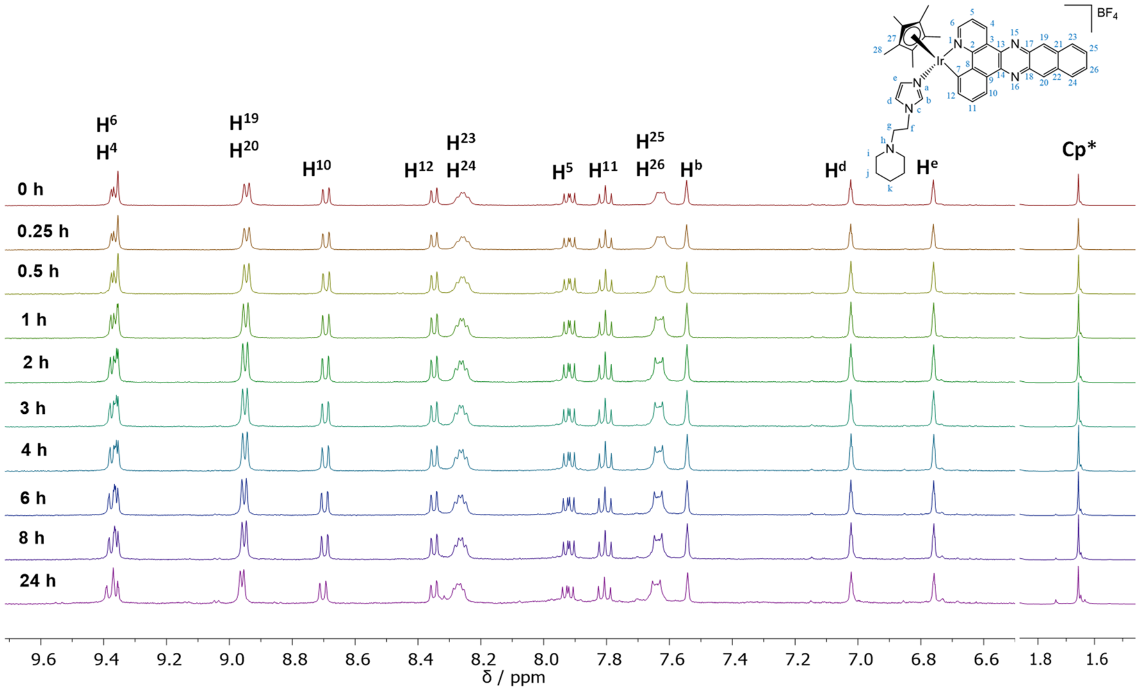

Figure S63. ^{1}H NMR of complex **6** in DMSO-d_6/D_2O (9/1) at different times under blue light irradiation (470 nm).

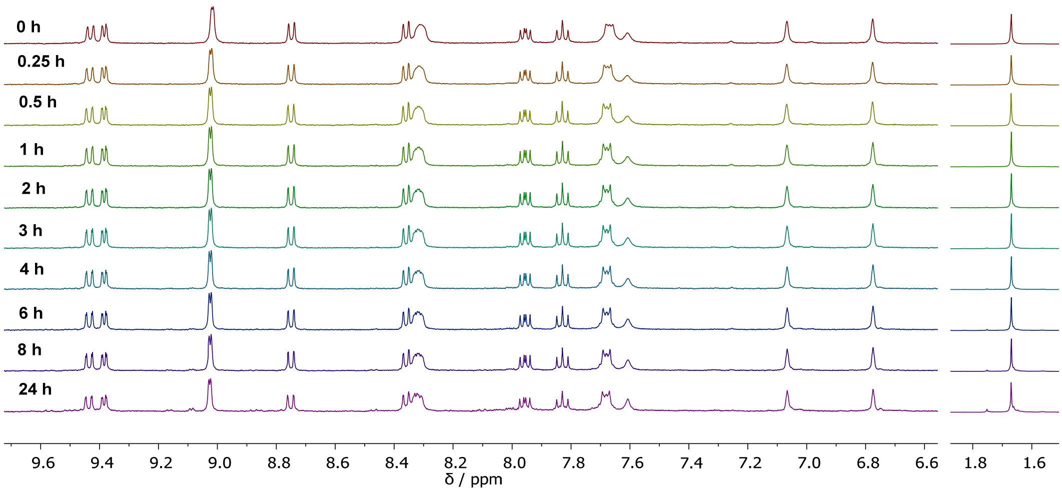

Figure S64. ^{1}H NMR of complex **6** in DMSO-d_6/D_2O (9/1) at different times under green light irradiation (530 nm).

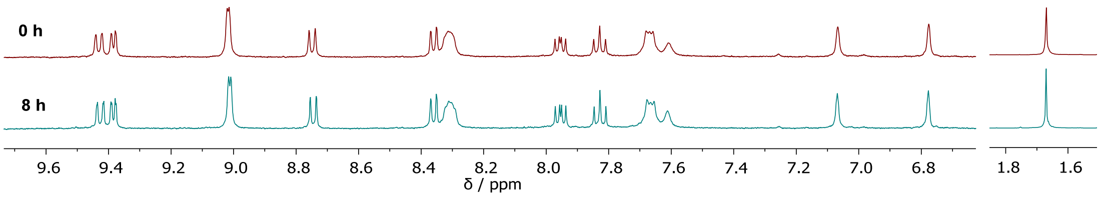

Figure S65. ^{1}H NMR of complex **6** in DMSO-d_6/D_2O (9/1) at different times under red light irradiation (655 nm).

$^{1}O_2$ generation

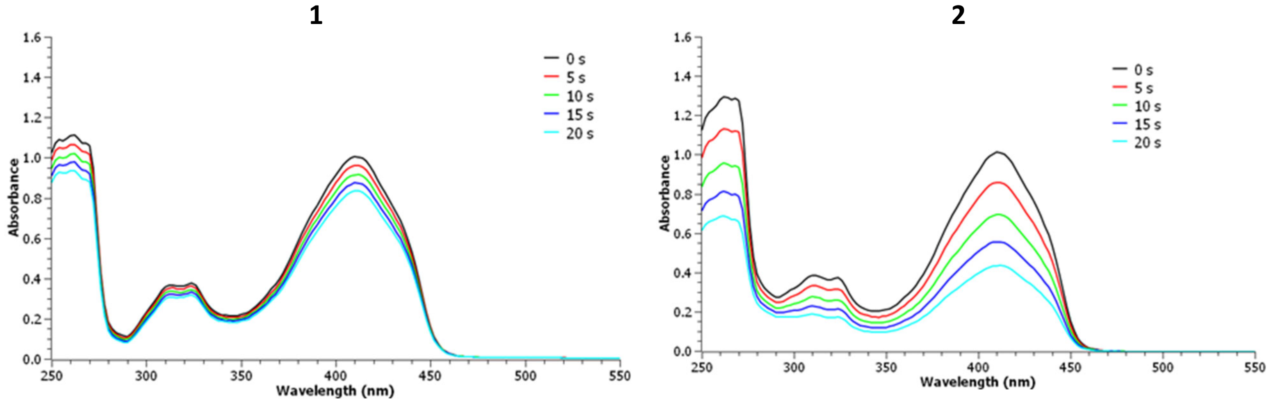

Figure S66. Evolution of the UV-vis spectra of DPBF in the presence of complexes **1** and **2** in acetonitrile under blue light (470 nm) irradiation. The peak situated at 410 nm (characteristic of DPBF) decreased steadily with its oxidation.

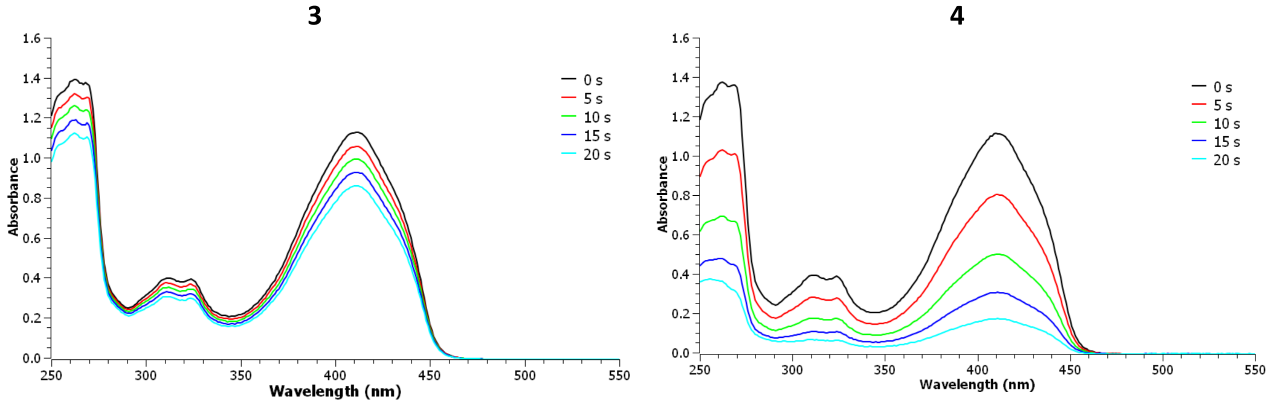

Figure S67. Evolution of the UV-vis spectra of DPBF in the presence of complexes **3** and **4** in acetonitrile under blue light (470 nm) irradiation. The peak situated at 410 nm (characteristic of DPBF) decreased steadily with its oxidation.

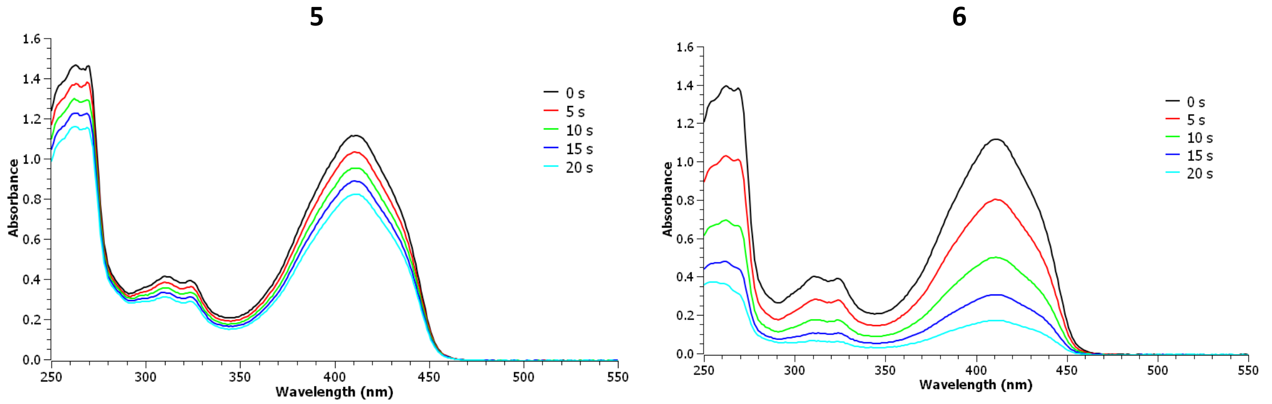

Figure S68. UV-vis spectra of DPBF in the presence of in the presence of complexes **5** and **6** in acetonitrile under blue light (470 nm) irradiation. The peak situated at 410 nm (characteristic of DPBF) decreased steadily with its oxidation.

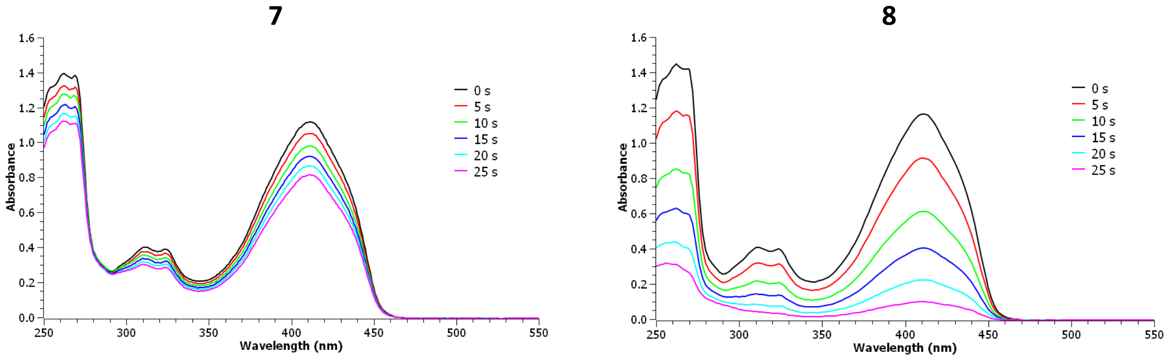

Figure S69. Evolution of the UV-vis spectra of DPBF in the presence of complexes **7** and **8** in acetonitrile under blue light (470 nm) irradiation. The peak situated at 410 nm (characteristic of DPBF) decreased steadily with its oxidation.

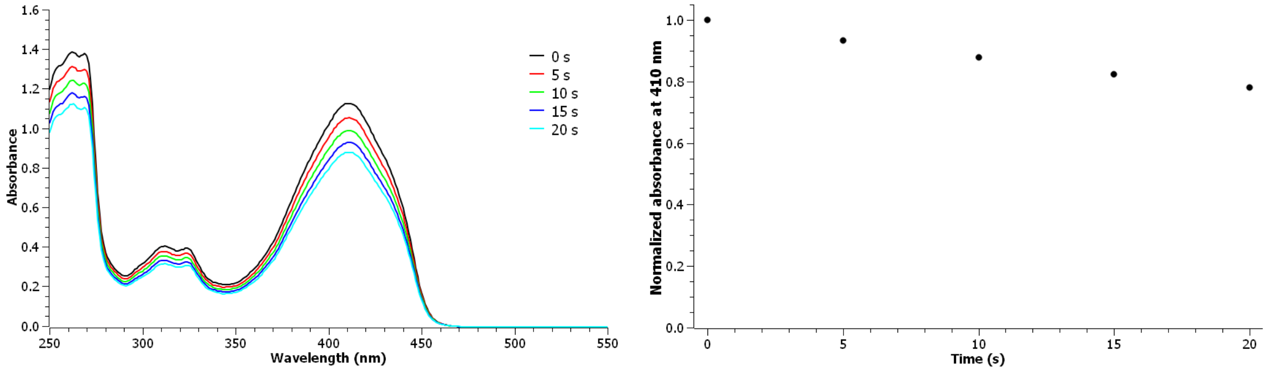

(a) (b)

Figure S70. DPBF photobleaching under blue light irradiation (470 nm). (a) UV-vis absoprtion of DPBF with time. (b) Graph showing the absorption at 410 nm with time.

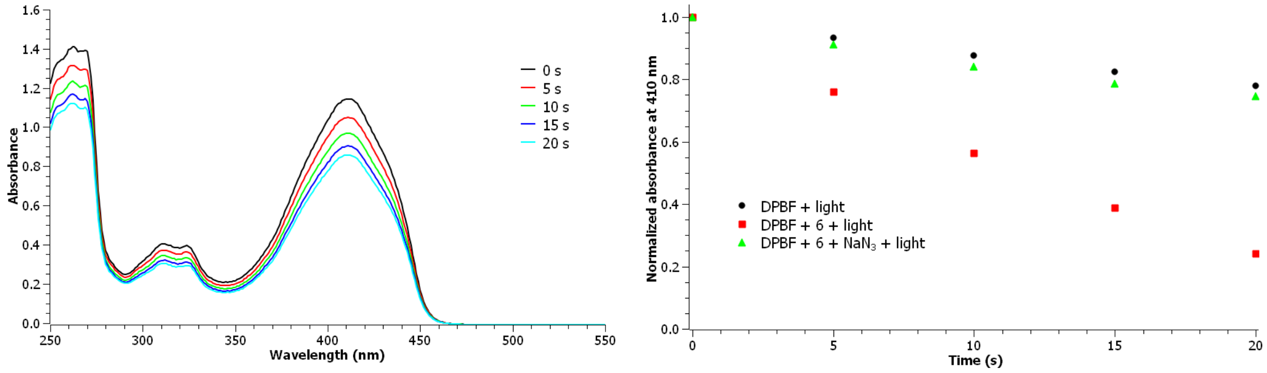

(a) (b)

Figure S71. (a) UV-vis spectra of DPBF in the presence of complex **6** with NaN_3 as $^1O_2$ scavenger under blue light irradiation and (b) comparison of 410 nm absorbance decrease with time.

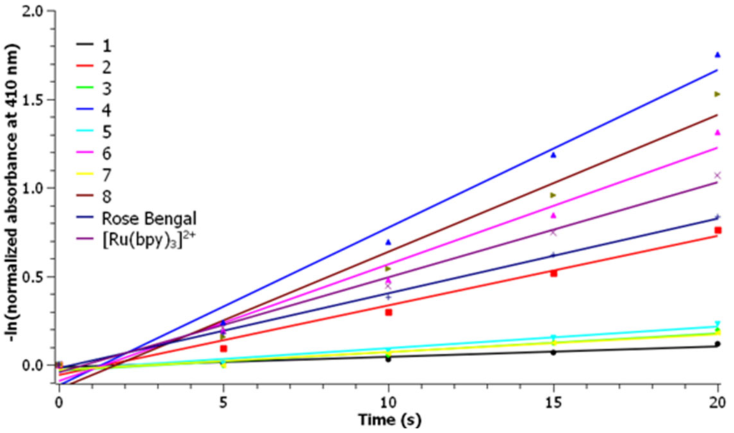

Figure S72. Comparative plots of -ln(normalized Abs at 410 nm) as a function of time for the experiment of photooxidation of DPBF in the presence of complexes **1**-**8** under blue light irradiation (470 nm).

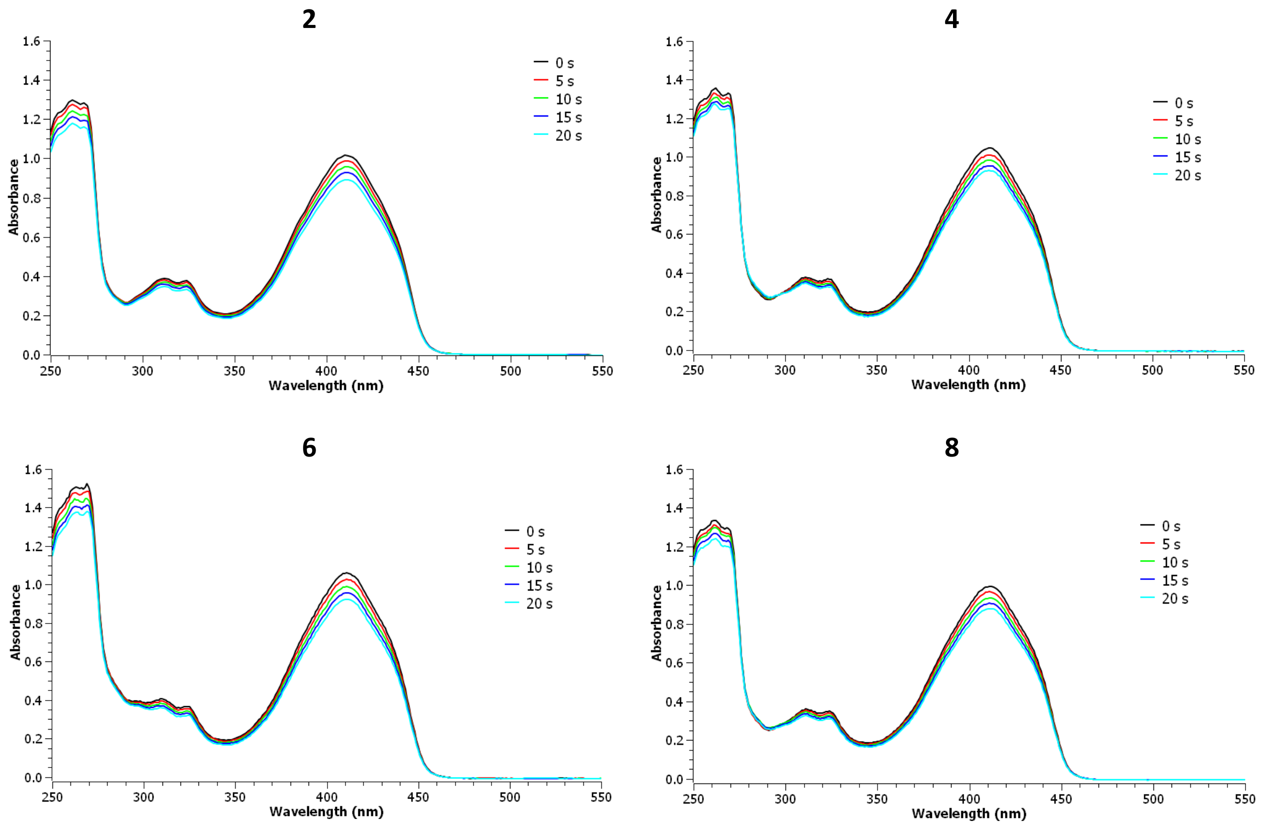

Figure S73. UV-vis spectra of DPBF in the presence of complexes **2**, **4**, **6** and **8** inn acetonitrile under green light irradiation (530 nm). The peak situated at 410 nm (characteristic of DPBF) decreased with its oxidation.

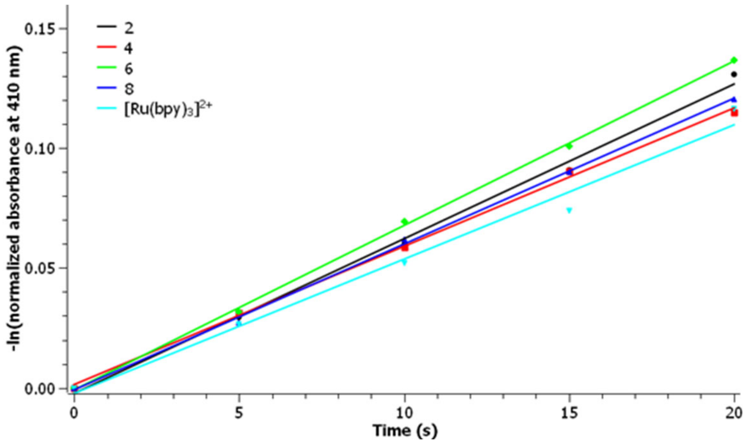

Figure S74. Comparative plots of -ln(normalized Abs at 410 nm) as a function of time for the experiment of photooxidation of DPBF in the presence of complexes **2**, **4**, **6** and **8** in acetonitrile under green light irradiation (530 nm).

Table S8. Slope (S) of the plot of -ln(normalized Abs) vs time for the experiments of photooxidation of DPBF in the presence of complexes **4**, **6** and **8** under blue or green light irradiation.

	**S**	
**Complex**	**λ_{ir} = 470 nm**	**λ_{ir} = 530 nm**
**$[Ru(bpy)_3]^{2+}$**	0.0535	0.0057
**2**	0.0541	0.0065
**4**	0.0890	0.0058
**6**	0.0657	0.0069
**8**	0.0771	0.0061

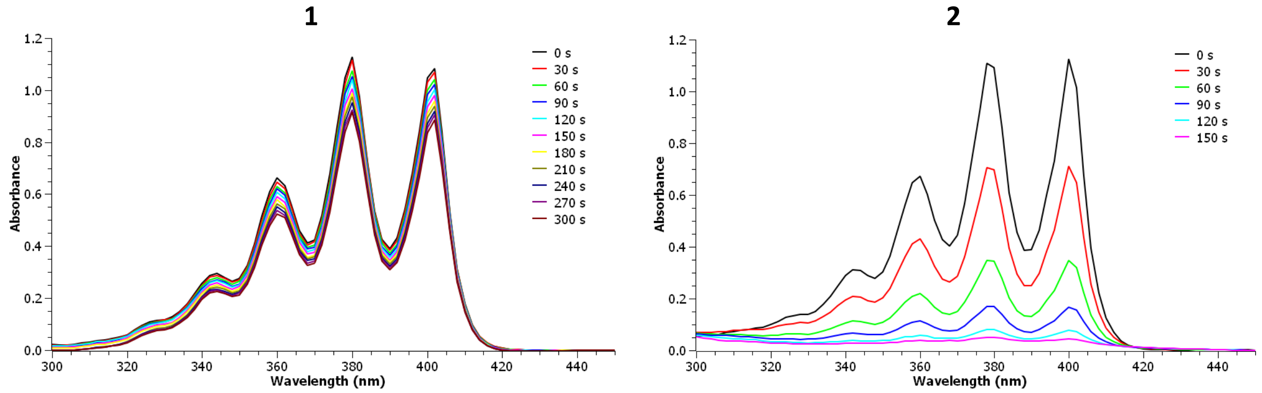

Figure S75. UV-vis spectra of ABDA in the presence of complexes **1** and **2** in H_2O:DMSO (95:5) under blue light (470 nm) irradiation. The peak situated at 402 nm (characteristic of ABDA) decreased steadily with its oxidation.

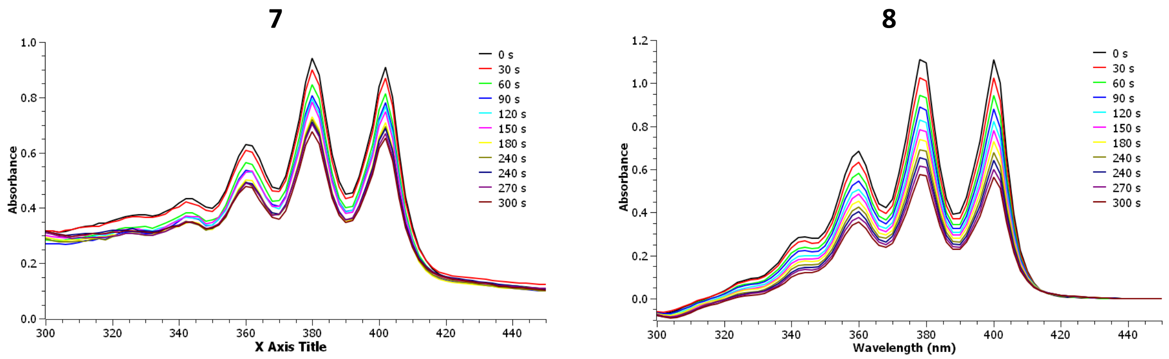

Figure S76. Evolution of the UV-vis spectra of ABDA in the presence of complexes **7** and **8** in H_2O:DMSO (95:5) under blue light (470 nm) irradiation. The peak situated at 402 nm (characteristic of ABDA) decreased steadily with its oxidation.

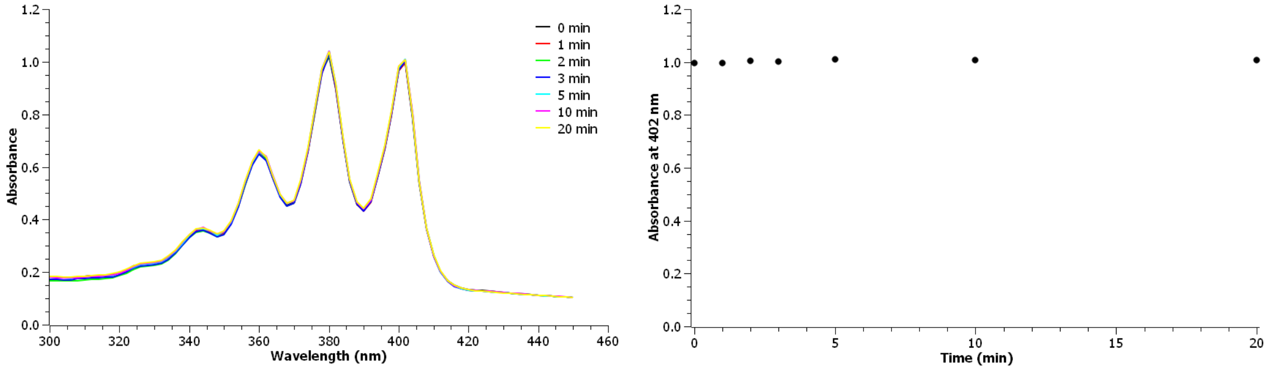

(a) (b)

Figure S77. ABDA photostability under blue light irradiation (470 nm). (a) UV-vis absoprtion of ABDA with time. (b) Graph showing the absorption at 402 nm with time.

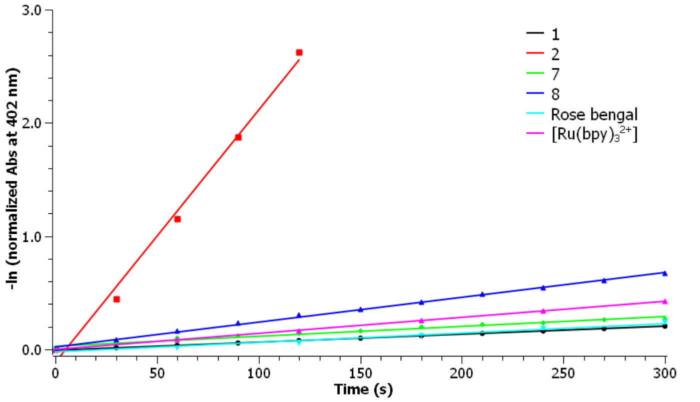

Figure S78. Comparative plots of -ln(normalized Abs at 402 nm) as a function of time for the experiment of photooxidation of ABDA in the presence of complexes **1**-**2** and **7**-**8** under blue light irradiation (470 nm).

Table S9. Singlet oxygen generation quantum yield (ϕ_Δ) of complexes **1**-**2** and **7**-**8** calculated with different probes.

	ϕ_Δ	
**Complex**	**DPBF**	**ABDA**
**1**	0.0780	0.1913
**2**	0.7509	0.8132
**7**	0.0204	0.0526
**8**	0.9900	0.4986

Photophysical properties

UV-vis absorption

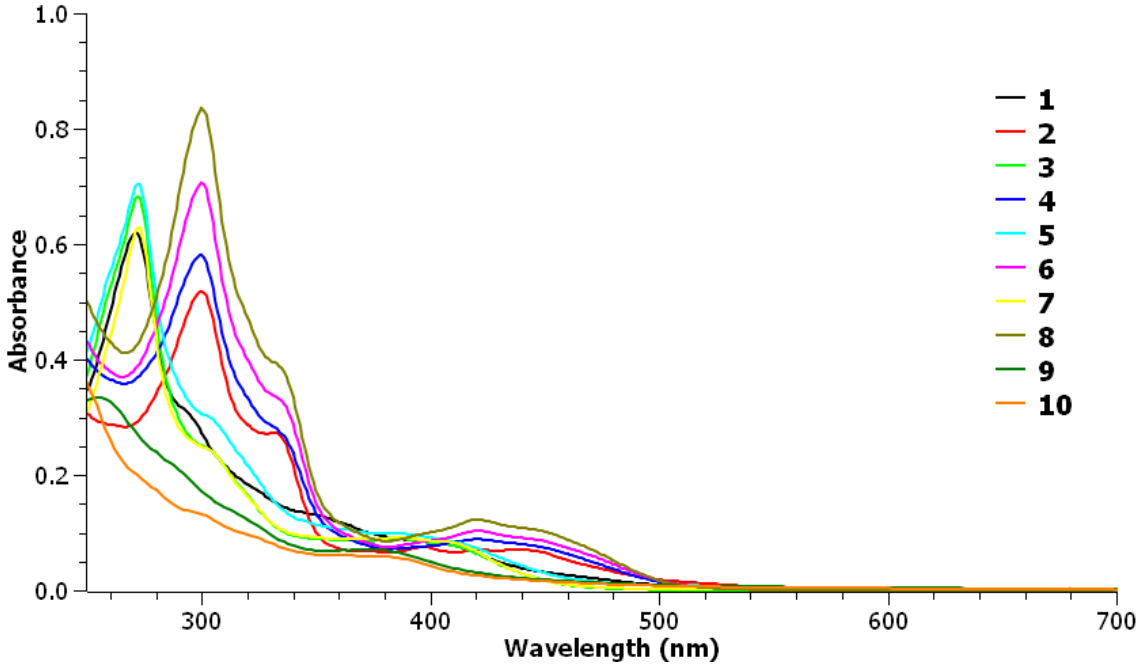

Figure S79. UV-vis absorption spectra of complexes **1–10** at 1.0×10^{-5} M in degassed acetonitrile.

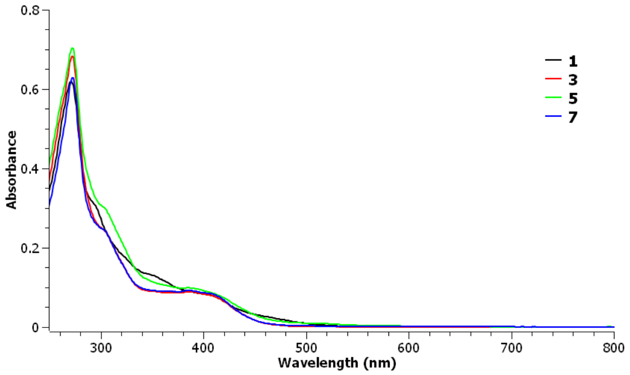

(a)

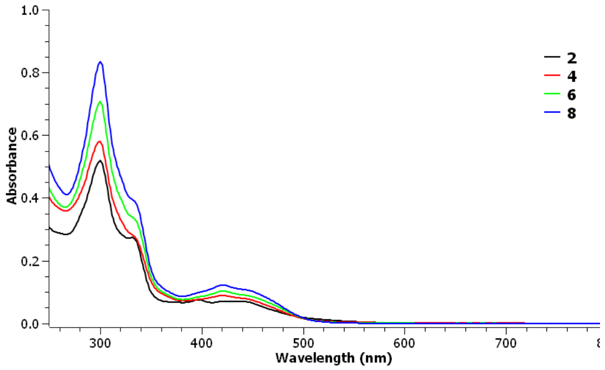

(b)

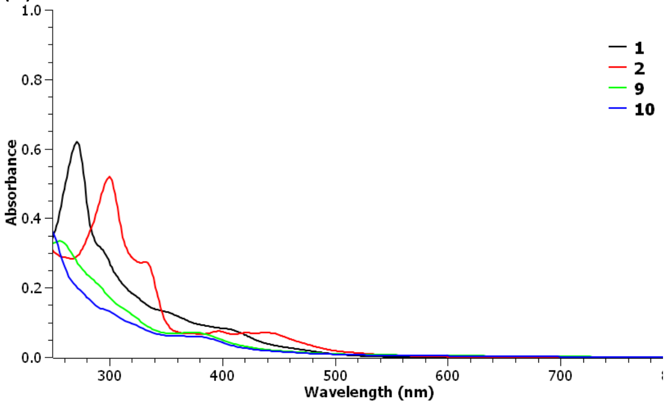

(c)

Figure S80. UV-vis absorption spectra of complexes **1**, **3**, **5** and **7** with C^N = pbpz (a), complexes **2**, **4**, **6** and **8** with C^N = pbpn (b) and complexes **1**, **2**, **9** and **10**, with chloride as monodentate ligand (c) in degassed acetonitrile solutions (1.0×10^{-5} M).

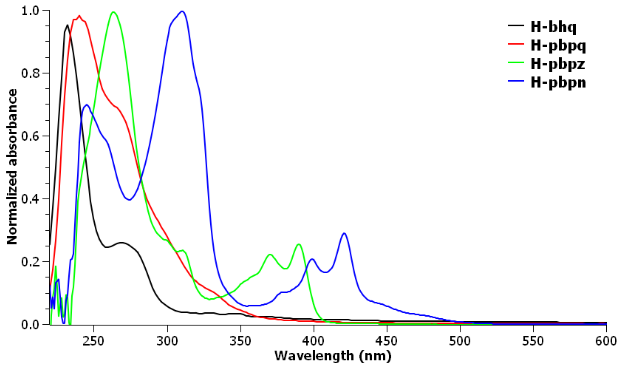

Figure S81. Normalized UV-vis absorption spectra of H-bhq, H-pbpq, H-pbpz and H-pbpn proligands at 1.0 × 10^{-5} M in degassed DCM.

Table S10. Photophysical properties of complexes **1**–**10** and Hpbpz and Hpbpn.

Comp.	**λ / nm (ε / M^{-1}·cm^{-1})**	ε / M^{-1}cm^{-1} λ = 460 nm	ε / M^{-1}cm^{-1} λ = 530 nm	ε / M^{-1}cm^{-1} λ = 605 nm	λ_{em} (λ_{exc}) /nm	ϕ_Δ[a]
1	271 (62400), 303 (25100), 351 (13100), 386 (9000), 409 (7900)	2610	240	0	-	0.0780
2	301 (52300), 332 (27600), 397 (7500), 421 (7100), 448 (6700)	5250	940	80	546 (420)	0.7509
3	272 (69100), 301 (25000), 349 (9100), 387 (8900), 411 (7700)	890	40	0	-	0.0399
4	299 (58400), 333 (27900), 398 (7700), 422 (8900), 448 (7600)	6280	520	210	550 (420)	0.9894
5	273 (71300), 302 (30400), 350 (11400), 386 (10000), 411 (8300)	2130	630	170	-	0.0260
6	300 (71300), 333 (33500), 398 (8600), 420 (10300), 449 (8600)	7400	330	70	546 (420)	0.8118
7	273 (63700), 290 (28400), 302 (24700), 351 (9400), 386 (9200), 411 (8100)	970	190	70	-	0.0204
8	300 (84400), 333 (39400), 398 (9900), 420 (12300), 448 (10400)	8800	200	20	546 (420)	0.9900
9	218 (43000), 256 (33600), 296 (18700), 378 (7200)	1620	710	430	-	n.m.
10	218 (71700), 250 (36100), 300 (13300), 378 (6000)	1390	570	330	.	n.m.
Hpbpz[b]	264 (94800), 299 (25300), 312 (22500), 353 (12900), 370 (21400), 390 (25000)	150	80	45	412 (360)	n.m.
Hpbpn[b]	258 (47400), 304 (77900), 311 (80600), 380 (8200), 399 (17200), 421 (24000)	5050	760	130	540 (422)	n.m.

[a] The ϕ_Δ (quantum yield of $^1O_2$ generation) in CH_3CN solution under blue irradiation was determined using $[Ru(bpy)_3]Cl_2$ (ϕ_Δ = 0.56)[1] for complexes **2**, **4**, **6** and **8** or Rose Bengal (ϕ_Δ = 0.53)[2] for complexes **1**, **3**, **5** and **7**, as references.
[b] In CH_2Cl_2. n.m. = not measured.

(1) Xu, Y.; Wang, X.; Song, K.; Du, J.; Liu, J.; Miao, Y.; Li, Y. BSA-Encapsulated Cyclometalated Iridium Complexes as Nano-Photosensitizers for Photodynamic Therapy of Tumor Cells. *RSC Adv.* **2021**, *11* (25), 15323–15331. https://doi.org/10.1039/d1ra01740c.

(2) Epelde-Elezcano, N.; Martínez-Martínez, V.; Peña-Cabrera, E.; Gómez-Durán, C. F. A.; Arbeloa, I. L.; Lacombe, S. Modulation of Singlet Oxygen Generation in Halogenated BODIPY Dyes by Substitution at Their: Meso Position: Towards a Solvent-Independent Standard in the Vis Region. *RSC Adv.* **2016**, *6* (48), 41991–41998. https://doi.org/10.1039/c6ra05820e.

Emission

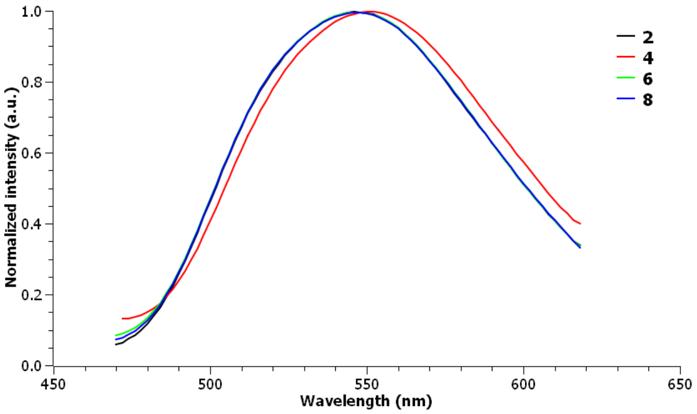

Figure S82. Normalized emission spectra of complexes **2**, **4**, **6** and **8** at 1.0 × 10^{-5} M in degassed acetonitrile (λ_{exc} = 420 nm).

Transient Absorption Spectroscopy (TAS)

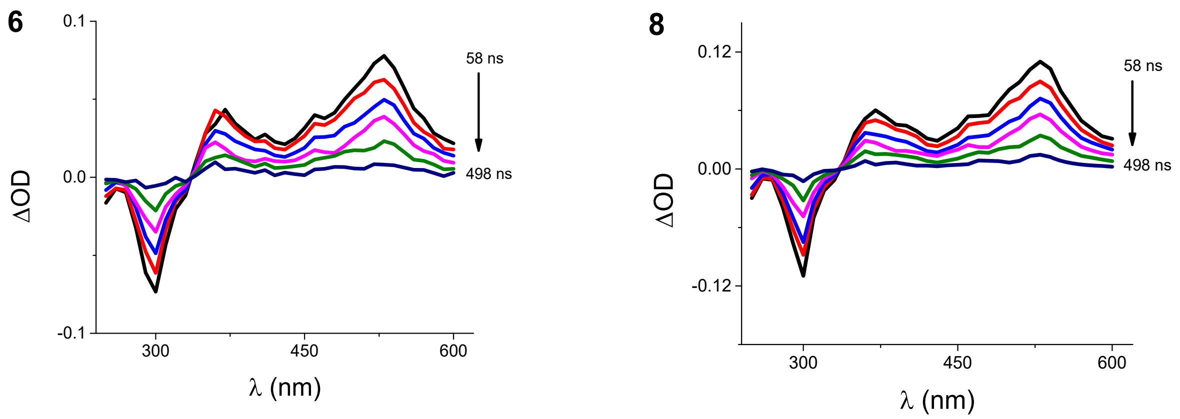

Figure S83. Transient absorption spectra of complexes **6** and **8** in acetonitrile (λ_{exc} = 355 nm). It is observed strong negative ΔOD values at around 300 nm (depletion of the S_0 ground state transition) and positive ΔOD values in the region between 350 and 600 nm.

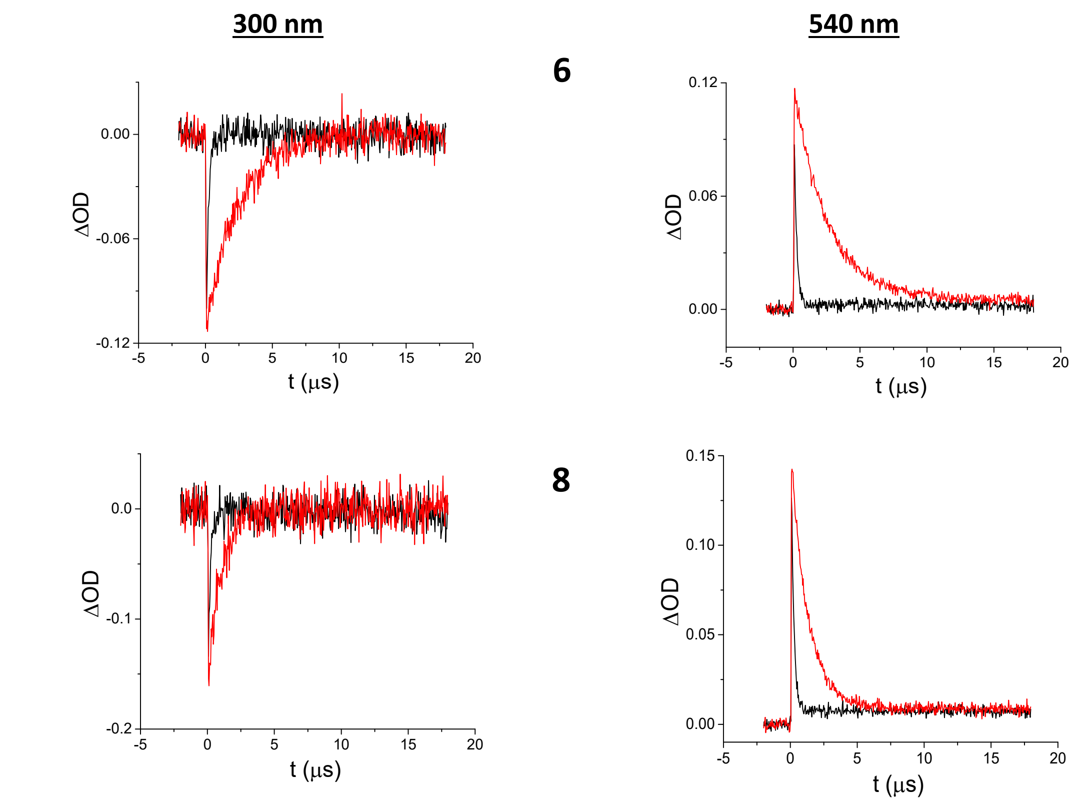

Figure S84. Time-resolved transients of **6** and **8** in acetonitrile recorded at 300 nm (left) and 540 nm (right), in aerated (black) and degassed (red) conditions (λ_{exc} = 355 nm). Lifetimes of around 200 nanoseconds in the presence of oxygen were observed. Degassing (by bubbling argon through the solution for 5–15 minutes) led to a significant increase (by about one order of magnitude) in the respective lifetimes and this gave rise to microsecond decay times (identical at 300 and 540 nm, within the measurement error) that are compatible with a triplet state decay.

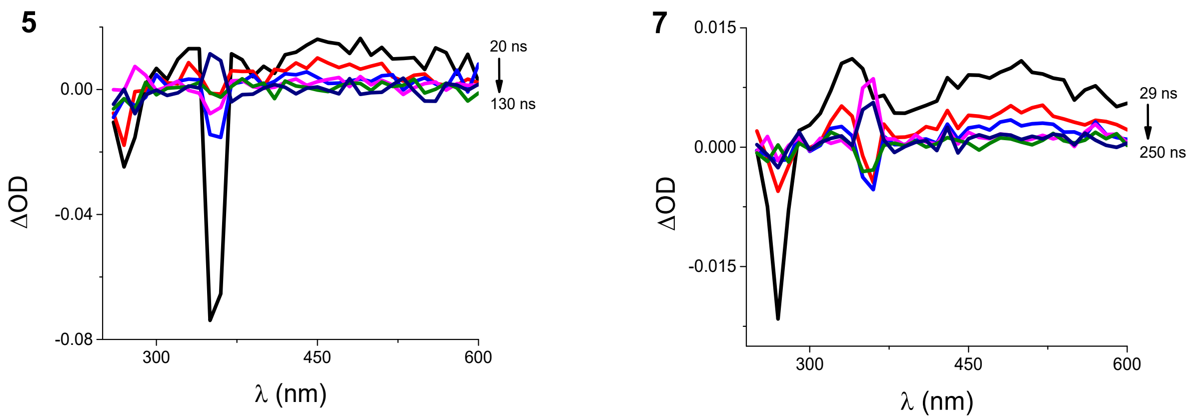

Figure 85. Transient absorption spectra of complexes **5** and **7** in acetonitrile (λ_{exc} = 355 nm). A very low variation in the optical density (ΔOD) with short decay times is observed.

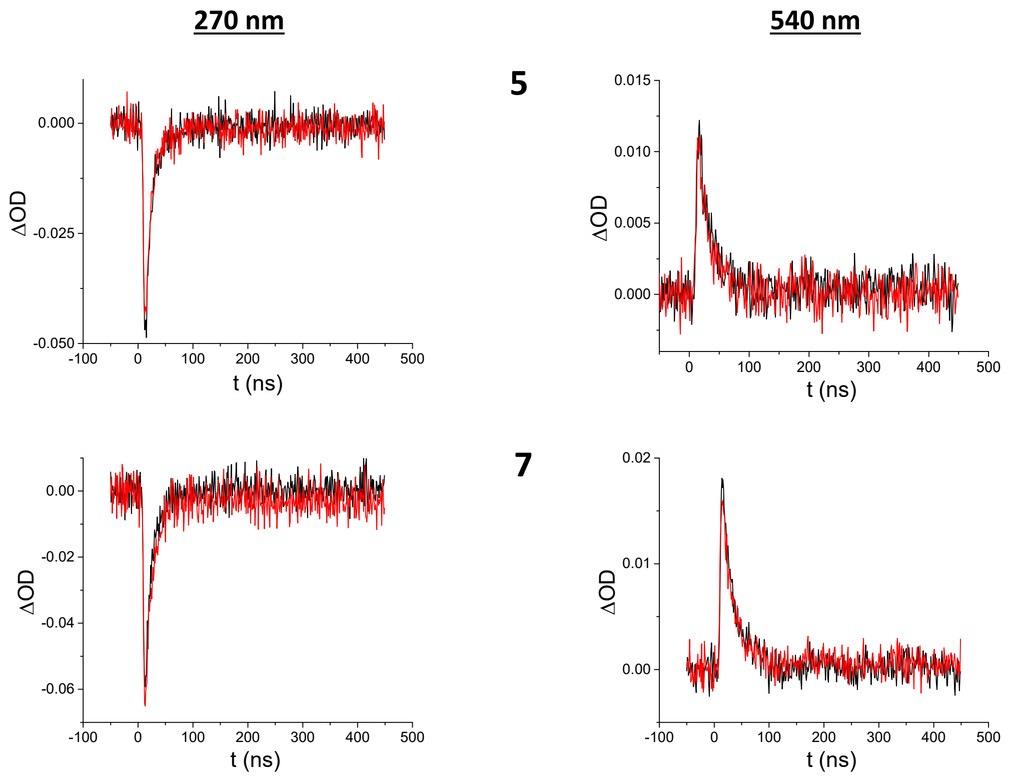

Figure S86. Time-resolved transients of **5** and **7** in acetonitrile recorded at 270 nm (left) and 540 nm (right), in aerated (black) and degassed (red) conditions (λ_{exc} = 355 nm). The decay rates from the depletion and recovery of the S_0 ground state transition (negative transients at 270 nm) best fitted with a single exponential law and a lifetime of around 15 nanoseconds. Analysis of the positive ΔOD transients at 540 nm yielded also monoexponential decays with a similar lifetime (~20 ns). The nanosecond decay times did not change significantly upon degassing the solution, i.e., on this time scale there was no evidence of quenching by oxygen either through energy transfer or another mechanism.

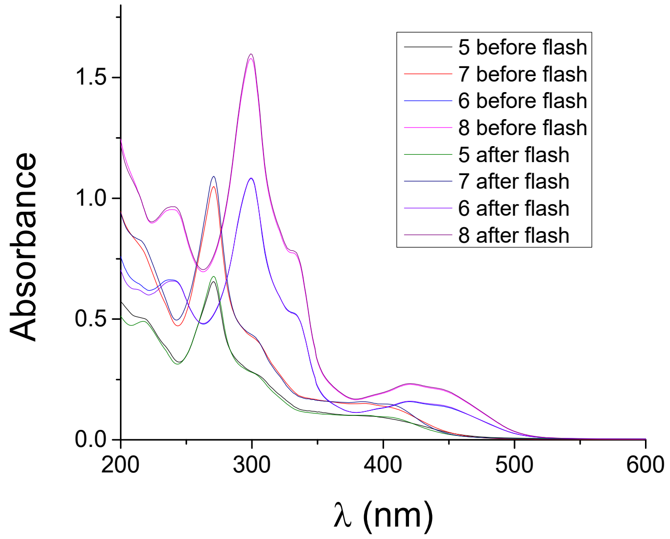

Figure S87. UV-vis absorption spectra of complexes **5**-**8**, before and after flash photolysis experiments.

TD-DTF calculations

Table S11. Energies in electron volts (E/eV) and main fragmental contributions (percentage values within brackets) to some molecular orbitals of **5**, **6**, **7** and **8** obtained at the TD-DFT (SMD, acetonitrile)/6-31G(d,p)//SDD level.

**Orbital**	**E/eV**		**5**	**6**
	**5**	**6**	**Fragmental contributions**	**Fragmental contributions**
**LUMO**	-2.43	-2.71	Q (74) Py (15) Bz (8)	Bq (78) Py (12) Bz (8)
**HOMO**	-5.60	-5.59	Ir (42) Bz (22) Cp* (22) Py (6)	Ir (42) Cp* (24) Bz (21) Py (7)
**HOMO-1**	-5.75	-5.64	Ir (44) Cp*(27) Bz (13) Im (6) py (5)	Bq (87) Bz (8) Py (4)
**HOMO-2**	-5.84	-5.75	Etpi(89)	Ir (45) Cp* (26) Bz* (14) Py (6) Im (6)
**HOMO-3**	-6.27	-5.85	Q (45) Bz (34) Py (17)	Etpi (93)
**Orbital**	**E/eV**		**7**	**8**
	**7**	**8**	**Fragmental contributions**	**Fragmental contributions**
**LUMO**	-2.43	-2.71	Q (74) Py (16) Bz (8)	Bq (77) Py (12) Bz (8)
**HOMO**	-5.60	-5.57	Ir (44) Cp* (23) Bz (21) Py (7) Im (4)	Ir (43) Cp* (27) Bz (18) Py (6)
**HOMO-1**	-5.77	-5.65	Ir (43) Cp*(28) Bz (16) Py (6) Im (5)	Bq (87) Bz (8) Py (4)
**HOMO-2**	-6.27	-5.79	Q (46) Bz (34) Py (17)	Ir (43) Cp* (24) Bz (18) Py (6) Im (6)
**HOMO-3**	-6.40	-6.40	Ir (43) Cp*(18) Im (10) Q (10) Bz (10)	Ir (41) Cp* (17) Bq (15) Im (10) Bz (9)

Cp*: cyclopentadiene, Ir: iridium, Bq: benzo-quinoxaline, Q: quinoxaline, Bz: fused benzene, Py: pyridine, Im: imidazole, Etpi: ethylpiperidine.

Table S12. Main single electronic transitions for **5** obtained at TD-DFT(SMD, acetonitrile)/6-31G(d,p)//SDD level.

**State**	**Wavelength/nm (oscillator strength)**	**Major transitions (contribution percentage)/type of transition**
**S_1**	466 (0.043)	$d_{H}\rightarrow\pi_{L}$ (98) /MLCT
**S_2**	439 (0.027)	$d_{H-1}\rightarrow\pi_{L}$ (98) /MLCT
**S_3**	424 (0.002)	$\pi_{H-2}\rightarrow\pi_{L}$ (100) /(LLCT)
**S_4**	400 (0.007)	$d_{H}\rightarrow\pi_{L+1}$ (92) $d_{H-1}\rightarrow\pi_{L+1}$ (3) /MLCT
**S_5**	376 (0.038)	$\pi_{H-3}\rightarrow\pi_{L}$ (79) $d_{H-1}\rightarrow\pi_{L+1}$ (16)/ LLCT
**S_6**	372 (0.006)	$\pi_{H-8}\rightarrow\pi_{L}$ (76) $d_{H-4}\rightarrow\pi_{L}$(13) $\pi_{H-7}\rightarrow\pi_{L}$(6) / LC
**S_7**	370 (0.070)	$d_{H-1}\rightarrow\pi_{L+1}$ (74) $\pi_{H-3}\rightarrow\pi_{L}$(16) $d_{H}\rightarrow\pi_{L+1}$(3) / MLCT/LLCT
**S_8**	358 (0.081)	$d_{H-4}\rightarrow\pi_{L}$(82) $\pi_{H-8}\rightarrow\pi_{L}$(11) / MLCT/ LC
**S_9**	353 (0.001)	$\pi_{H-2}\rightarrow\pi_{L+1}$ (98) / LLCT
**S_{10}**	339 (0.062)	$d_{H-5}\rightarrow\pi_{L}$ (88) $\pi_{H-8}\rightarrow\pi_{L}$ (3) / MLCT

Table S13. Main single electronic transitions for **6** obtained at TD-DFT(SMD, acetonitrile)/6-31G(d,p)//SDD level.

**State**	**Wavelength/ nm (oscillator strength)**	**Major transitions (contribution percentage)/type of transition**
**S_1**	515 (0.051)	$d_{H}\rightarrow\pi_{L}$ (98) /MLCT
**S_2**	498 (0.019)	$\pi_{H-1}\rightarrow\pi_{L}$ (98) /LC
**S_3**	481 (0.050)	$d_{H-2}\rightarrow\pi_{L}$ (98) /MLCT
**S_4**	462 (0.001)	$\pi_{H-3}\rightarrow\pi_{L}$(100) /LLCT
**S_5**	401 (0.012)	$\pi_{H-8}\rightarrow\pi_{L}$ (68) $d_{H-4}\rightarrow\pi_{L+1}$ (17) $d_{H}\rightarrow\pi_{L+1}$ (8) / LC/MLCT
**S_6**	400 (0.008)	$d_{H}\rightarrow\pi_{L+1}$(87) $\pi_{H-8}\rightarrow\pi_{L}$(7) /MLCT/ LC
**S_7**	388 (0.112)	$d_{H-4}\rightarrow\pi_{L}$ (59) $\pi_{H-8}\rightarrow\pi_{L}$(13) $\pi_{H}\rightarrow\pi_{L+1}$(12) $\pi_{H-6}\rightarrow\pi_{L}$(9) / MLCT/LC/LLCT
**S_8**	380 (0.003)	$\pi_{H-5}\rightarrow\pi_{L}$(81) $d_{H-4}\rightarrow\pi_{L}$(7) / LLCT/ MLCT
**S_9**	372 (0.030)	$d_{H-2}\rightarrow\pi_{L+1}$ (28) $\pi_{H-6}\rightarrow\pi_{L}$(28) $\pi_{H-1}\rightarrow\pi_{L+1}$(24) / MLCT/LLCT
**S_{10}**	369 (0.097)	$d_{H-2}\rightarrow\pi_{L}$ (54) $\pi_{H-6}\rightarrow\pi_{L}$ (21) $\pi_{H-5}\rightarrow\pi_{L}$ (11) / MLCT/LLCT

Table S14. Main single electronic transitions for **7** obtained at TD-DFT(SMD, acetonitrile)/6-31G(d,p)//SDD level.

**State**	**Wavelength / nm (oscillator strength)**	**Major transitions (contribution percentage)/type of transition**
**S_1**	466 (0.042)	$d_{H}\rightarrow\pi_{L}$ (98) /MLCT
**S_2**	436 (0.041)	$d_{H-1}\rightarrow\pi_{L}$ (98) /MLCT
**S_3**	404 (0.007)	$d_{H}\rightarrow\pi_{L+1}$ (94) /(MLCT)
**S_4**	375 (0.042)	$\pi_{H-2}\rightarrow\pi_{L}$ (68) $d_{H}\rightarrow\pi_{L+1}$ (25) /LLCT/MLCT
**S_5**	372 (0.003)	$\pi_{H-8}\rightarrow\pi_{L}$ (29) $\pi_{H-7}\rightarrow\pi_{L}$(27) $\pi_{H-9}\rightarrow\pi_{L}$(19) $d_{H-3}\rightarrow\pi_{L}$(13) / LLCT/MLCT
**S_6**	370 (0.063)	$d_{H-1}\rightarrow\pi_{L+1}$ (63) $\pi_{H-2}\rightarrow\pi_{L}$(27) /MLCT/LLCT
**S_7**	356 (0.087)	$d_{H-3}\rightarrow\pi_{L}$ (82) $\pi_{H-8}\rightarrow\pi_{L}$(4) $\pi_{H-9}\rightarrow\pi_{L}$(4) / MLCT/LLCT
**S_8**	341 (0.000)	$d_{H}\rightarrow\pi_{L+2}$(44) $d_{H}\rightarrow\pi_{L+3}$(27) $\pi_{H-4}\rightarrow\pi_{L}$(8) / MLCT/LLCT
**S_9**	340 (0.012)	$\pi_{H-4}\rightarrow\pi_{L}$ (72) $d_{H-5}\rightarrow\pi_{L}$(10) $\pi_{H-9}\rightarrow\pi_{L}$(5) /MLCT/ LLCT
**S_{10}**	335 (0.009)	$d_{H-5}\rightarrow\pi_{L}$ (78) $\pi_{H-4}\rightarrow\pi_{L}$ (11) /MLCT/LLCT

Table S15. Main single electronic transitions for **8** obtained at TD-DFT(SMD, acetonitrile)/6-31G(d,p)//SDD level.

State	Wavelength/nm (oscillator strength)	Major transitions (contribution percentage)/type of transition
S_1	519 (0.040)	$d_{H} \rightarrow \pi_{L}$ (99) /MLCT
S_2	496 (0.017)	$\pi_{H-1} \rightarrow \pi_{L}$ (99) /LC
S_3	475 (0.079)	$d_{H-2} \rightarrow \pi_{L}$ (98) /MLCT
S_4	407 (0.001)	$d_{H} \rightarrow \pi_{L+1}$(95) /MLCT
S_5	401 (0.019)	$\pi_{H-7} \rightarrow \pi_{L}$ (50) $d_{H-3} \rightarrow \pi_{L}$ (18) $\pi_{H-9} \rightarrow \pi_{L}$ (14) $\pi_{H-8} \rightarrow \pi_{L+1}$ (14) / LLCT/MLCT
S_6	386 (0.105)	$d_{H-3} \rightarrow \pi_{L}$(57) $\pi_{H-1} \rightarrow \pi_{L}$(15) $\pi_{H-7} \rightarrow \pi_{L+1}$(7) /MLCT/ LC/LLCT
S_7	379 (0.001)	$\pi_{H-4} \rightarrow \pi_{L}$ (83) $d_{H-3} \rightarrow \pi_{L}$(8) /LLCT/ MLCT
S_8	369 (0.135)	$d_{H-2} \rightarrow \pi_{L+1}$(75) $\pi_{H-1} \rightarrow \pi_{L+1}$(14) / MLCT/LLCT
S_9	368 (0.004)	$\pi_{H-5} \rightarrow \pi_{L}$ (66) $\pi_{H-1} \rightarrow \pi_{L+1}$(8) $\pi_{H-4} \rightarrow \pi_{L}$(7) /LLCT
S_{10}	361 (0.052)	$d_{H-6} \rightarrow \pi_{L}$ (44) $\pi_{H-1} \rightarrow \pi_{L+1}$ (22) $\pi_{H-5} \rightarrow \pi_{L}$ (13) $d_{H-2} \rightarrow \pi_{L+1}$ (6) / MLCT/LLCT

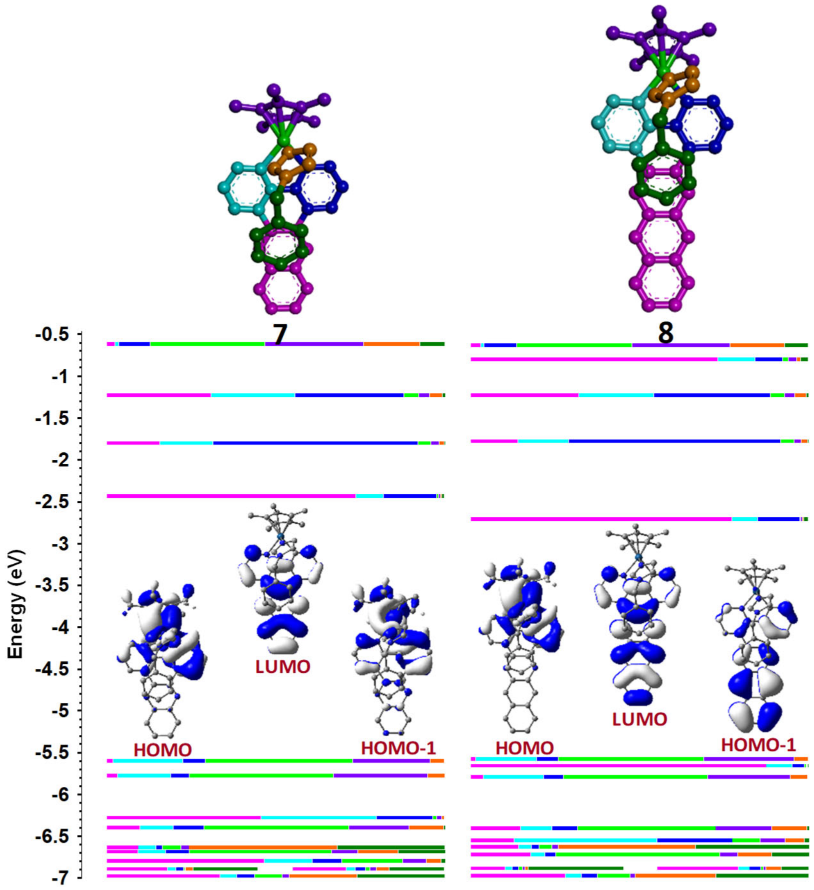

Figure S88. Optimized ground state structures for **7** and **8** obtained from TD-DFT [(B3LYP/SDD for Ir(III)) and (6–31 g** for C,H,N)] with SMD (CH_3CN). For the sake of clarity, the hydrogen atoms are not shown and each molecular fragment has been assigned a color code. Calculated fragmental contributions and energies for some selected molecular orbitals are provided along with the topologies of HOMO-1, HOMO and LUMO for **7** and **8**. The length of each color bar is proportional to the percentage contribution of the corresponding colored moiety to each molecular orbital. Color codes for bars and molecular fragments: Cp* (purple), iridium (light green), quinoxaline or benzoquinoxaline (pink), benzene (blue), pyridine (navy blue), imidazole (brown), benzyl (dark green).

Table S16. Adiabatic excitation energies (eV) calculated at the optimized lowest-lying singlet and triplet excited states of **6**, **5**, **8** and **7** at the TD-DFT (SMD, acetonitrile)/6-31G(d,p)//SDD level.

State	Energy/eV			
	**5**	**6**	**7**	**8**
S_0	0.00	0.00	0.00	0.00
S_1	2.18	1.99	2.16	1.94
S_2	2.19	1.93	2.16	2.25
T_1	1.92	1.44	1.93	1.46

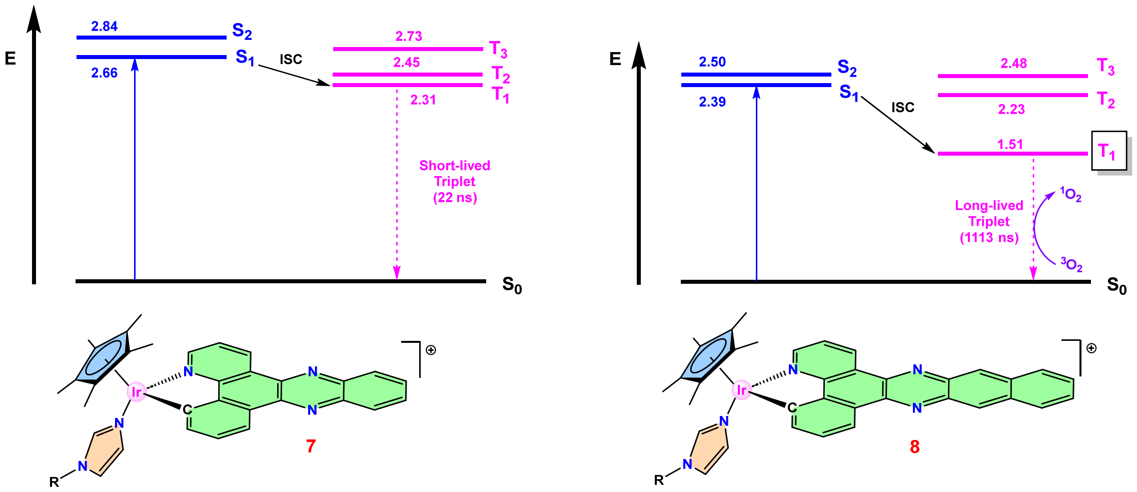

Scheme S2. Diagram showing the energy (eV) values for the lowest vertically excited states (S_n and T_n) calculated by TD-DFT for **7** and **8**. Experimental lifetimes for excited triplet states from TAS studies are included between brackets.

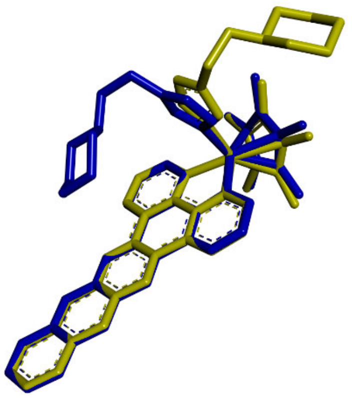

Figure S89. Superposition of the X-ray structure (yellow) and optimized DFT structure (blue) of compound **6**. Hydrogen atoms are hidden for clarity. RMSD = 0.025 Å.

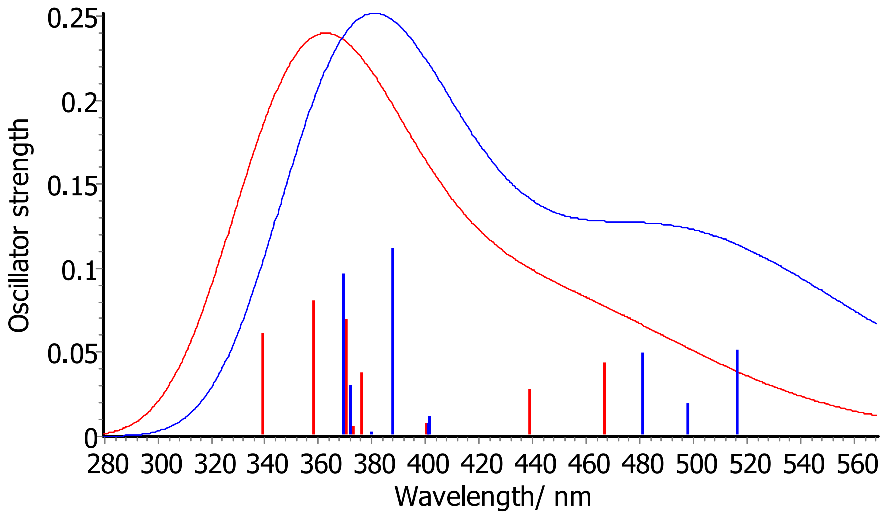

Figure S90. Simulated absorption spectra of compounds **5** (red) and **6** (blue) and their corresponding main transitions obtained from TD-DFT(SMD, acetonitrile)/6-31G(d,p)//SDD level.

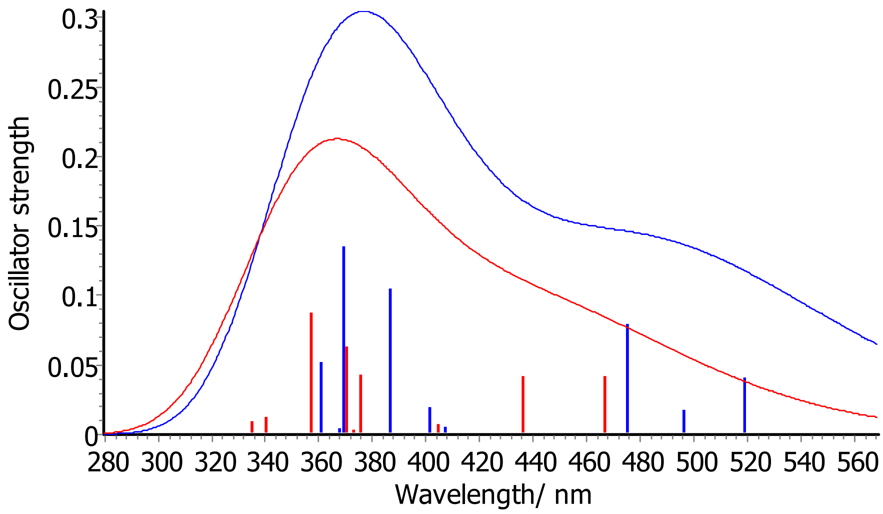

Figure S91. Simulated absorption spectra of compounds **7** (red) and **8** (blue) and their corresponding main transitions obtained from TD-DFT(SMD, acetonitrile)/6-31G(d,p)//SDD level.

Biological properties

Table S17. Lipophilicity of complexes **5**, **6**, **7** and **8** determined by the 'shake flash' method.

Complex	5[a]	6[a]	7[b]	8[b]
$logP/D_{9.2}$	1.10 ± 0.10	1.39 ± 0.07	1.65 ± 0.16	1.13 ± 0.03

[a] $logD_{9.2}$ for complexes **5** and **6**. [b] logP for complexes **7** and **8**.

Cytotoxic activity

Table S18. Above the red line: Data of PDT behavior for iridium complexes with the highest PI values. The results are ordered with decreasing energy of the light used.

Below the red line: PDT behavior for complexes with other transition metals other than Ir. Examples with PI values > 500 have been selected.

Entry	Type of complex	λ (nm)	Cells	PI[I]	Dose (J cm^{-2})	PI/Dose	Refer.
1	$[Ir(C^\wedge N)_2(NHC^\wedge NHC)]^+$	365	A549R	3488	72	48	a
2	$[Ir(C^\wedge N)_2(NHC^\wedge NHC)]^+$	365	A549	239	72	3.3	a
3	$[Ir(C^\wedge N)_2(N^\wedge N)]^+$	405	U2OS	>555	72	>7.7	b
4	$[Ir(C^\wedge N)_2(N^\wedge N)]^+$	405	HCT116	>384	72	>5.3	b
5	$[Ir(C^\wedge N)_2(N^\wedge N)]^+$	405	HeLa	>333	72	>4.6	b
6	$[Ir(C^\wedge N)_2(N^\wedge N)]^+$	405	A549	>959	20	>48	j
7	$[Ir(C^\wedge N)_2(N^\wedge N)]^+$	425	A549	>833	36	>23.1	c
8	$[Ir(C^\wedge N)_2(O^\wedge O)]$	425	A549	>1087	36	>30.2	d
9	$[Ir(C^\wedge N)_2(N^\wedge N)]^+$	425	HeLa	>476	1.2	>397	e
10	$[Ir(C^\wedge N)_2(N^\wedge N)]^+$	425	A549	>322	1.2	>268	e
11	$[IrC^\wedge N)_2(N^\wedge N^+)]^{2+}$	425	HepG2	386.7	40	9.7	r
12	$[IrC^\wedge N)_2(N^\wedge N^+)]^{2+}$	425	CNE-2	257.8	40	6.4	r
13	$[Cp^*Ir(N^\wedge O)(NCN\text{-}R\text{-}pyr)]^+$ [II]	450	HeLa	>417	10	>41.7	f
14	Complex **6**	460	A549	1317	24.1	54.6	This work
15	Complex **6**	460	HeLa	1031	24.1	42.7	This work
16	Complex **8**	460	A549	1260	24.1	52.3	This work
17	Complex **8**	460	PC-3	1179	24.1	48.9	This work
18	Complex **4**	460	A549	2034	24.1	84.4	This work
19	Complex **4**	460	PC-3	2022	24.1	83.9	This work
20	Complex **4**	530	A549	294	24.1	12.2	This work
21	$[Ir(C^\wedge N)_2(N^\wedge N)]^+$	Visible	SK-MEL28	407	123	3.3	g
22	$\{[Ir(N^\wedge C^\wedge N)(N^\wedge N^\wedge N)]_2(\mu\text{-fluo})\}^{4+}$ [III]	Visible	SK-MEL28	288	100	28.8	h
23	$[Ir(C^\wedge N)_2(C^\wedge NHC_{BODIPY})]^+$	Visible	SK-MEL28	135	100	1.35	i
24	$[Ir(C^\wedge N)_2(N^\wedge N)]^+$	625	SK-MEL28	32	105	0.30	g
25	$[Ir(C^\wedge N)_2(C_{BODIPY}{}^\wedge NHC)]^+$	625	SK-MEL28	>6	100	>0.06	i
26	$\{[Ir(N^\wedge C^\wedge N)(N^\wedge N^\wedge N)]_2(\mu\text{-fluo})\}^{4+}$ [III]	625	SK-MEL28	1	100	0.01	h
27	Complex **6**	655	A549	63	24.1	2.6	This work
28	$[Ir(C^\wedge N)_2(N^\wedge N)]+$	808	A375	>369	30	>12.3	k
29	$[Ir(C^\wedge N)_2(N^\wedge N)]+$	808	A375	>885	30	>29.5	k
30	$[Ir(C^\wedge N)_2(N^\wedge N)]+$	808	A375	>377	30	>15.6	k
31	$[Ir(N^\wedge N(pyr)^\wedge N)(N^\wedge N(Ph)^\wedge N)]^{3+}$ [IV]	visible	SK-MEL28	>1657	100	>165.7	l
32	$[Ir(N^\wedge N(pyr)_2{}^\wedge N]^{3+}$ [IV]	visible	SK-MEL28	628	100	62.8	l
33	$[Ru(bpy)_2(pbpn)]^+$	visible	SK-MEL28	>1400	100	>140	m
34	$[Ru(bpy)_2(dppn)]^+$ [V]	visible	SK-MEL28	1500	100	150	m
35	$[Ru(DIP)_2(phen)]^{2+}$ [VI]	540	CT26	1111	9	123.4	p
36	$[Ru(dppn)_2(phen)]^{2+}$	540	CT26	1666	9	185.1	p
37	$[Ru(dppn)_2(phen)]^{2+}$	540	HT29	556	9	61.8	p
38	$[Ru(dppn)_2(phen)]^{2+}$	540	RPE-1	556	9	61.8	p
39	$[Ru(dppn)_2(phen)]^{2+}$	620	RPE-1	333	6.7	49.7	p
40	$[Ru(bpy)_2(phen\text{-}pyr)]^{2+}$	visible	HL60	1747	100	174.7	q
41	$(Prpy)[transPtCl_2(DMSO)]_4$ [VII]	420	HeLa	>680	6.95	>24.5[VIII]	n

42	(Prpy)[transPtCl_2(DMSO)]$_4$ VII	420	CP70	1110	6.95	39.9 VIII	n
43	(Prpy[cisPtCl(NH_3)$_2$]$_4$ VII	420	HeLa	655	6.95	23.6 VIII	n
44	(Prpy)[cisPtCl(NH_3)$_2$]$_4$ VII	420	CP70	1930	6.95	6.4 VIII	n
45	(Prpy)[transPtCl(NH_3)$_2$]$_4$ VII	420	HeLa	1210	6.95	43.5 VIII	n
46	(Prpy)[transPtCl(NH_3)$_2$]$_4$ VII	420	CP70	>5260	6.95	>189.2 VIII	n
47	[Re(CO)$_3$(py)(N^N)]	505	HeLa	555	29.2	19	o

I PI = $IC_{50, dark}/IC_{50, light}$; II pyr = pyrene; III fluo = fluorenyl. IV pyR =Pyrenyl-1-yl; V dppn = benzo[i]dipyrido-[3,2-a:2′,3′-c]phenazine (dppn); VI DIP = 4,7-diphenyl-1,10-phenanthroline; VII Prpy = 5,10,15,20-tetra(4-pyridyl)porphyrin; VIII It has been considered that there are four platinum centers per molecule.

a.- Y. Li, C. P. Tan, W. Zhang, L. He, L. N. Ji and Z. W. Mao, *Biomaterials*, 2015, **39**, 95–104

b.- L. K. McKenzie, I. V. Sazanovich, E. Baggaley, M. Bonneau, V. Guerchais, J. A. G. Williams, J. A. Weinstein and H. E. Bryant, *Chem. - A Eur. J.*, 2017, **23**, 234–238.

c.- L. He, Y. Li, C. P. Tan, R. R. Ye, M. H. Chen, J. J. Cao, L. N. Ji and Z. W. Mao, *Chem. Sci.*, 2015, **6**, 5409–5418.

d.- Z. Y. Pan, W. W. Feng, Q. Y. Liu, L. He, D. H. Yao and Z. D. He, *Dye. Pigment.*, 2022, **203**, 110387.

e.- F. X. Wang, M. H. Chen, Y. N. Lin, H. Zhang, C. P. Tan, L. N. Ji and Z. W. Mao, *ACS Appl. Mater. Interfaces*, 2017, **9**, 42471–42481.

f.- L. Tabrizi, *Dalton Trans.*, 2017, **46**, 7242–7252.

g.- C. Wang, L. Lystrom, H. Yin, M. Hetu, S. Kilina, S. A. McFarland and W. Sun, *Dalt. Trans.*, 2016, **45**, 16366–16378.

h.- B. Liu, S. Monro, L. Lystrom, C. G. Cameron, K. Colón, H. Yin, S. Kilina, S. A. McFarland and W. Sun, *Inorg. Chem.*, 2018, **57**, 9859–9872.

i.- B. Liu, S. Monro, M. A. Jabed, C. G. Cameron, K. L. Colón, W. Xu, S. Kilina, S. A. Mcfarland and W. Sun, *Photochem. Photobiol. Sci.*, 2019, **18**, 2381–2396.

j.- S. Kuang, F. Wei, J. Karges, L. Ke, K. Xiong, X. Liao, G. Gasser, L. Ji, H. Chao, *J. Am. Chem. Soc.* 2022, 144, 4091-4101

k.- X.-L. Li, L.-Z. Zeng, R. Yang, X.-Da. Bi, Y. Zhang, R.-B. Cui, X.-X. Wu, F. Gao, *Inorg. Chem*. 2023, **62**, 16122−16130

l.- B. Liu, S. Monro, Z. Li, M. A. Jabed, D. Ramirez, C. G. Cameron, K. Colón, J. Roque, S. Kilina, J.Tian, S. A. McFarland, W. Sun, *ACS Appl. Bio Mater*. 2019, **2**, 2964.

m.-T. Sainuddin, J. McCain, M. Pinto, H. Yin, J. Gibson, M. Hetu, S. A. McFarland, *Inorg. Chem*. 2016, **55**, 83−95

n.- A. Naik, R. Rubbiani, G. Gasser, B. Spingler, *Angew. Chem. Int. Ed*. 2014, **126**, 7058 –7061.

o.- A. Kastl, S. Dieckmann, K. Whler, T. Vçlker, L. Kastl, A. L. Merkel, A. Vultur, B. Shannan, K. Harms, M. Ocker, W. J. Parak, M. Herlyn, E. Meggers, *ChemMedChem* 2013, **8**, 924 – 927.

p. M. D. Pozza, P. Mesdom, A. Abdullrahman, T.D. Prieto Otoya, P. Arnoux, C. Frochot, G. Niogret, B. Saubaméa, P. Burckel, J. P. Hall, M. Hollenstein, C. J. Cardin, G.Gasser, *Inorg. Chem.* 2023, https://doi.org/10.1021/acs.inorgchem.3c02606.

q.- R. Lincoln, L. Kohler, S. Monro, H. Yin, M. Stephenson, R. Zong, A. Chouai, C. Dorsey, R. Hennigar, R P. Thummel, S. A. McFarland, J. Am. Cghem. Soc. 2013, 135, 17161-17175.

r.- X.-D. Song, B.-B. Chen, S.-F. He, N.-L. Pan, J.-X. Liao, J.-X. Chen, G.-H. Wang, J. Sun, Eur. J. Med. Chem. 2019, **179** , 26-37.

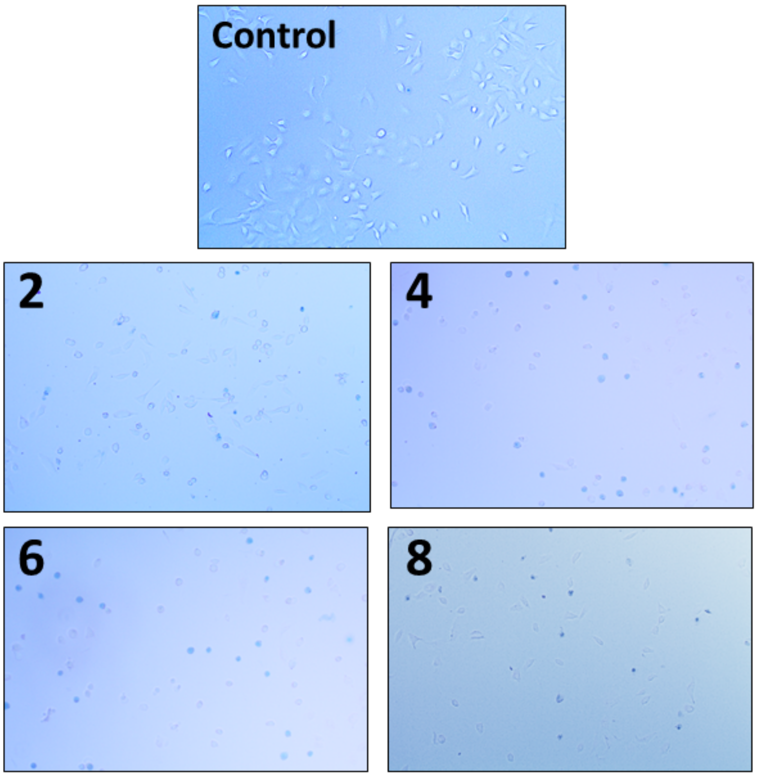

Figure S92. Cytotoxicity of complexes **2**, **4**, **6** and **8**. A549 cells were seeded in 96-well plates and treated with the complexes at a concentration corresponding to the IC_{50} in the dark. After 24 hours of incubation, 10 µl of Trypan Blue (0.4% solution; Sigma) was directly added to the wells to prevent the loss of dead cells during the treatment washes. Images of the cells were captured using an Olympus CKX41 Microscope equipped with LCmicro software (Olympus) after a ten-minute incubation period. Dead cells were identified by their blue coloration.

Hemolytic activity

Table S19. Hemolytic activity of complexes with pbpn ligands.

	**Hemolytic activity (%)***				
Concentration (µM)	**Cisplatin**	**2**	**4**	**6**	**8**
**25**	0.2	7.6	6.7	10.7	16.3
**10**	0	0	0.5	1.0	3.2
**5**	0	0	0	0	1.0
**1**	0	0	0	0	0.2

*Hemolytic activity was determined against porcine red blood cells (RBC). RBC were exposed to the complexes at different concentrations in the dark for 1 hour and to cisplatin as a control. Subsequently, RBC were pelleted by centrifugation and the supernatants were collected. Hemolysis was determined by measuring hemoglobin released in the supernatant. The percentage of hemolysis was obtained from the ratio between the absorbance of each sample and the absorbance of the positive control at 540 nm. Each dilution was assayed in triplicate.

Intracellular ROS production

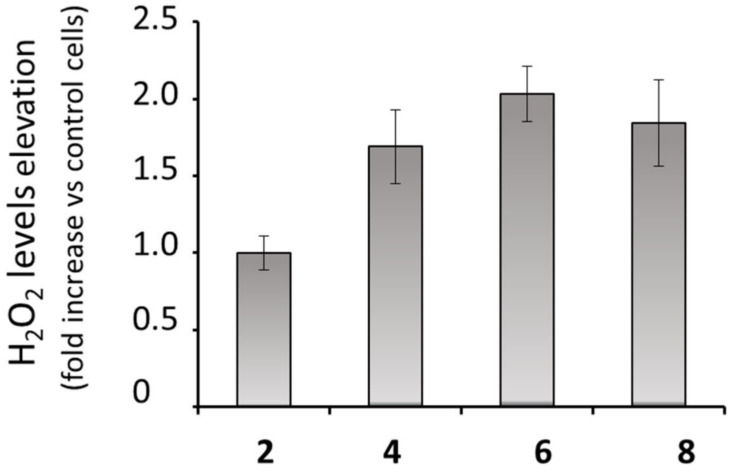

Figure S93. Cellular ROS generation. A549 cells were treated with complexes **2**, **4**, **6** and **8** at a concentration of 10 nM for four hours, followed by exposure to blue light irradiation for one hour (460 nm, 24.1 J cm^{-2}). Intracellular hydrogen peroxide (H_2O_2) levels elevations were determined with a cell-permeant specific probe. The fluorescence was measured by flow cytometry. Barrs represent the mean fold increase (± standard deviation) relative to control untreated cells from three independent experiments. * $p < 0.05$ compared to control cells.

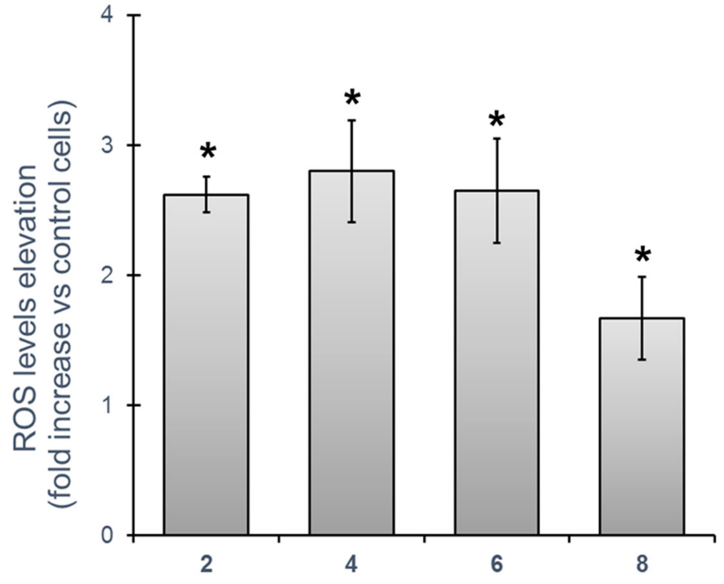

Figure S94. Cellular ROS generation. A549 cells were incubated with complexes **2**, **4**, **6** and **8** at the corresponding $IC_{50, dark}$ for 5 hours. ROS levels were determined with the H_2DCFDA probe. The mean ROS elevations + standard deviation relative to control untreated cells observed in three indepenent experiments are represented. * $p < 0.05$ vs control cells.

Cellular uptake

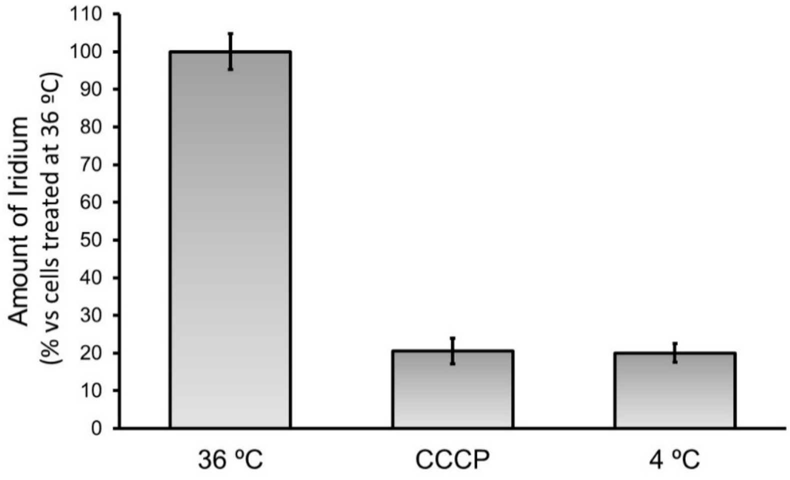

Figure S95. Cellular uptake mechanism of complex **6**. Cells were incubated with **6** at 10 µM for 1 hour at 37 °C alone and in the presence of CCCP (50 µM) or at low temperature (4 °C) to inhibit the active transport. The intracellular iridium content under each set of conditions was measured by ICP-MS and calculated as a % of control cells treated at 37 °C. The mean values and standard deviation of two independent experiments are represented.

NADH oxidation

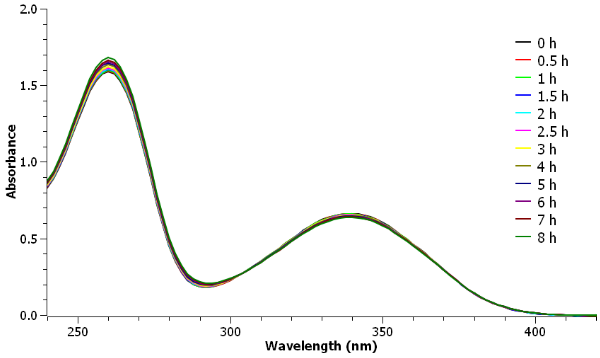

Figure S96. UV–vis spectra for the oxidation of NADH (100 µM) to NAD$^+$ without photosensitizer over a period of 24 hours in MeOH/H_2O (2.5/97.5) at room temperature under blue light irradiation (470 nm).

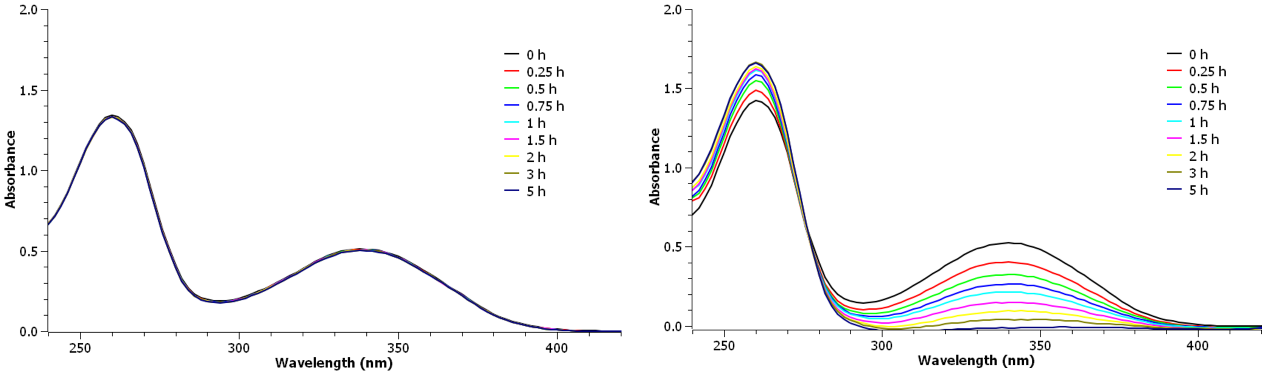

(a) (b)

Figure S97. UV–vis spectra for the catalytic oxidation of NADH (100 µM) to NAD$^+$ in the presence of complex **1** (2.5 µM) over a period of 8 hours in DMSO/H_2O (2.5/97.5) at room temperature, in the dark (a) and under blue light irradiation (470 nm) (b).

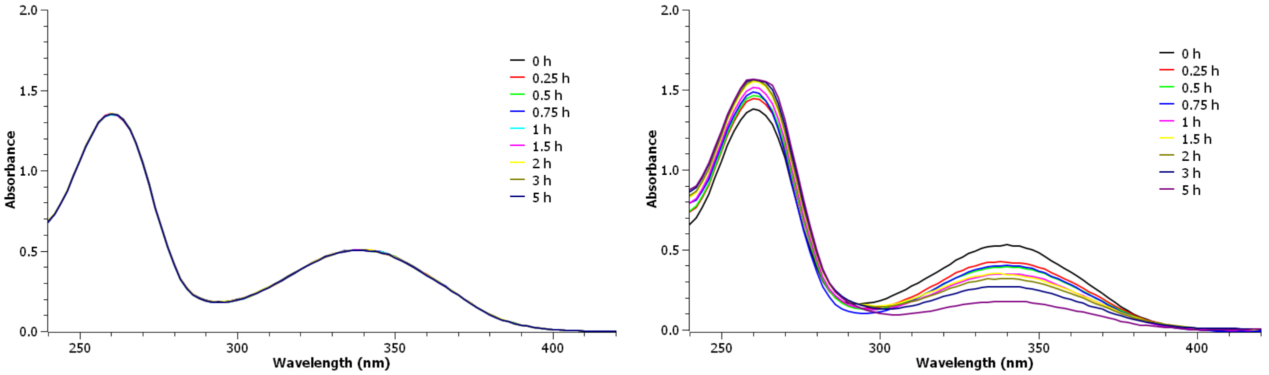

(a) (b)

Figure S98. UV−vis spectra for the catalytic oxidation of NADH (100 μM) to NAD$^+$ in the presence of complex **2** (2.5 μM) over a period of 5 hours in DMSO/H_2O (2.5/97.5) at room temperature, in the dark (a) and under blue light irradiation (470 nm) (b).

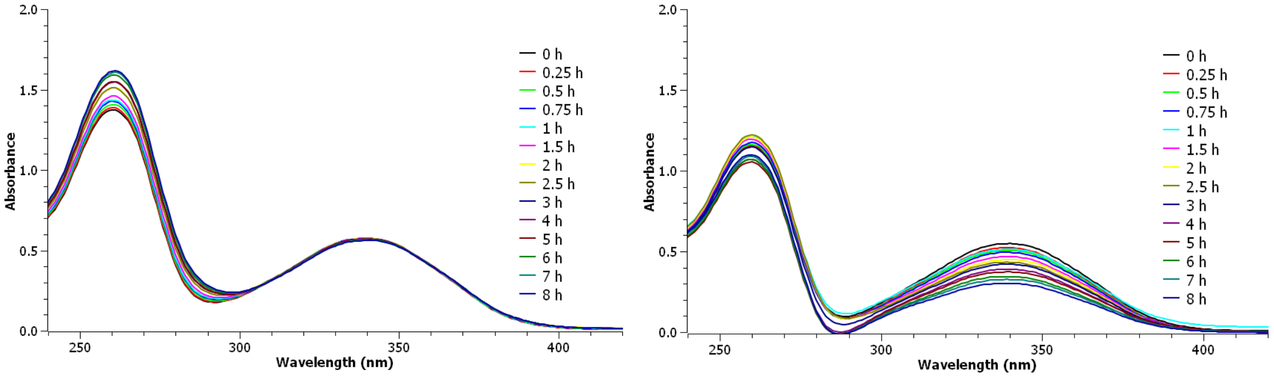

(a) (b)

Figure S99. UV−vis spectra for the catalytic oxidation of NADH (100 μM) to NAD$^+$ in the presence of complex **3** (2.5 μM) over a period of 8 hours in MeOH/H_2O (2.5/97.5) at room temperature, in the dark (a) and under blue light irradiation (470 nm) (b).

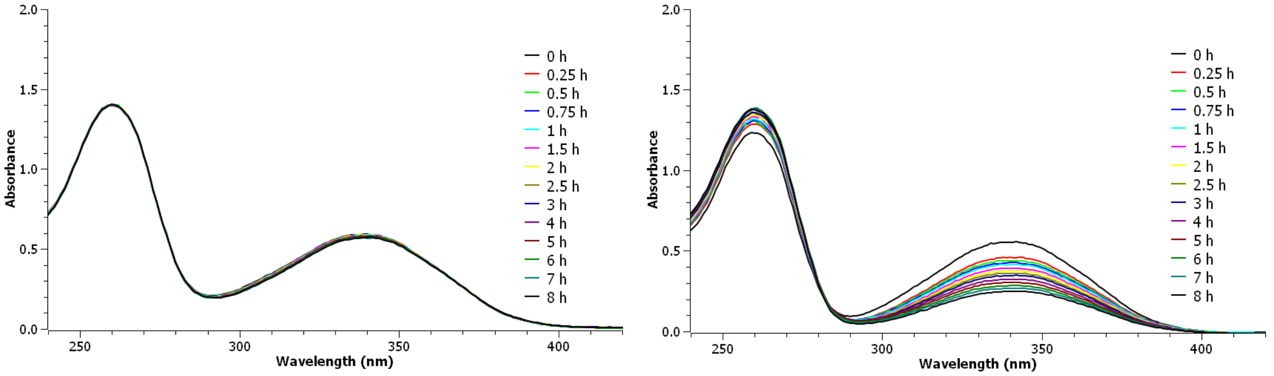

(a) (b)

Figure S100. UV−vis spectra for the catalytic oxidation of NADH (100 μM) to NAD+ in the presence of complex **4** (2.5 μM) over a period of 8 hours in MeOH/H_2O (2.5/97.5) at room temperature, in the dark (a) and under blue light irradiation (470 nm) (b).

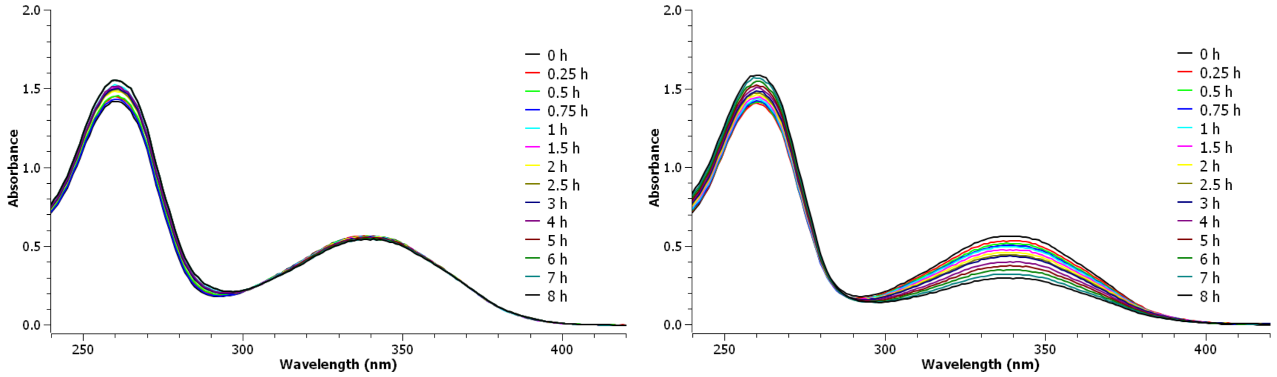

(a) (b)

Figure S101. UV–vis spectra for the catalytic oxidation of NADH (100 μM) to NAD^+ in the presence of complex **5** (2.5 μM) over a period of 8 hours in MeOH/H_2O (2.5/97.5) at room temperature, in the dark (a) and under blue light irradiation (470 nm) (b).

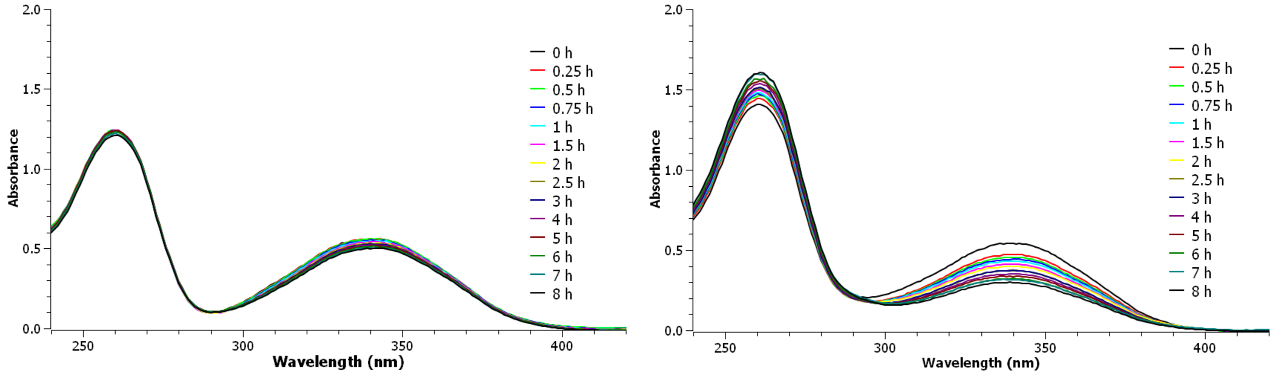

(a) (b)

Figure S102. UV–vis spectra for the catalytic oxidation of NADH (100 μM) to NAD^+ in the presence of complex **6** (2.5 μM) over a period of 8 hours in MeOH/H_2O (2.5/97.5) at room temperature, in the dark (a) and under blue light irradiation (470 nm) (b).

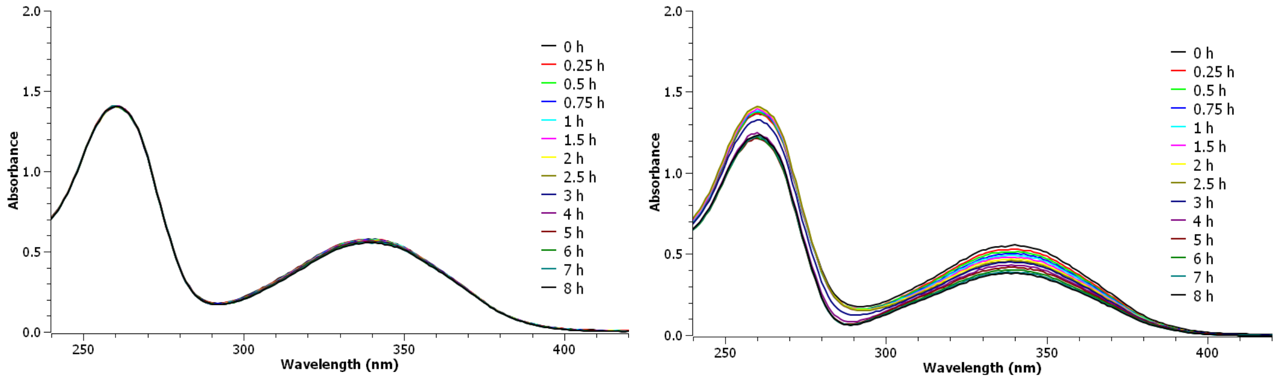

(a) (b)

Figure S103. UV–vis spectra for the catalytic oxidation of NADH (100 μM) to NAD^+ in the presence of complex **7** (2.5 μM) over a period of 8 hours in MeOH/H_2O (2.5/97.5) at room temperature, in the dark (a) and under blue light irradiation (470 nm) (b).

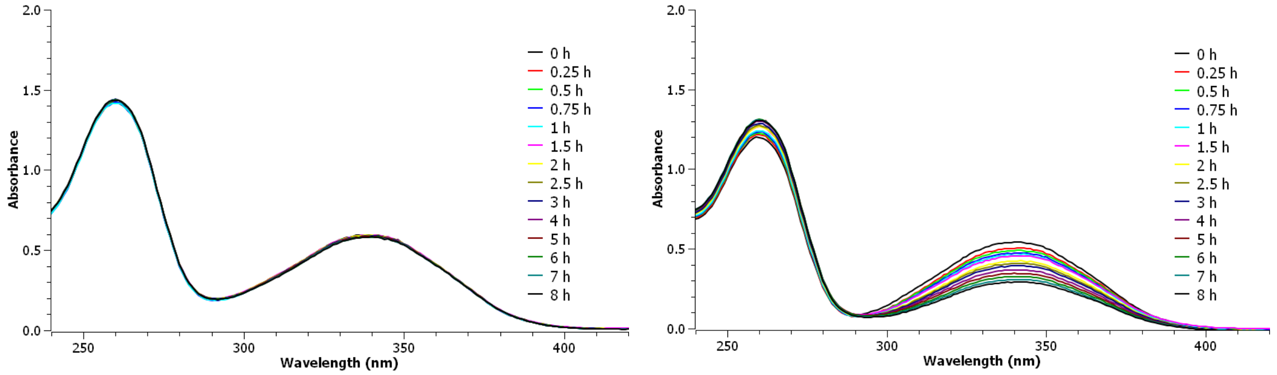

(a) (b)

Figure S104. UV–vis spectra for the catalytic oxidation of NADH (100 µM) to NAD^+ in the presence of complex **8** (2.5 µM) over a period of 8 hours in MeOH/H_2O (2.5/97.5) at room temperature, in the dark (a) and under blue light irradiation (470 nm) (b).

(a)

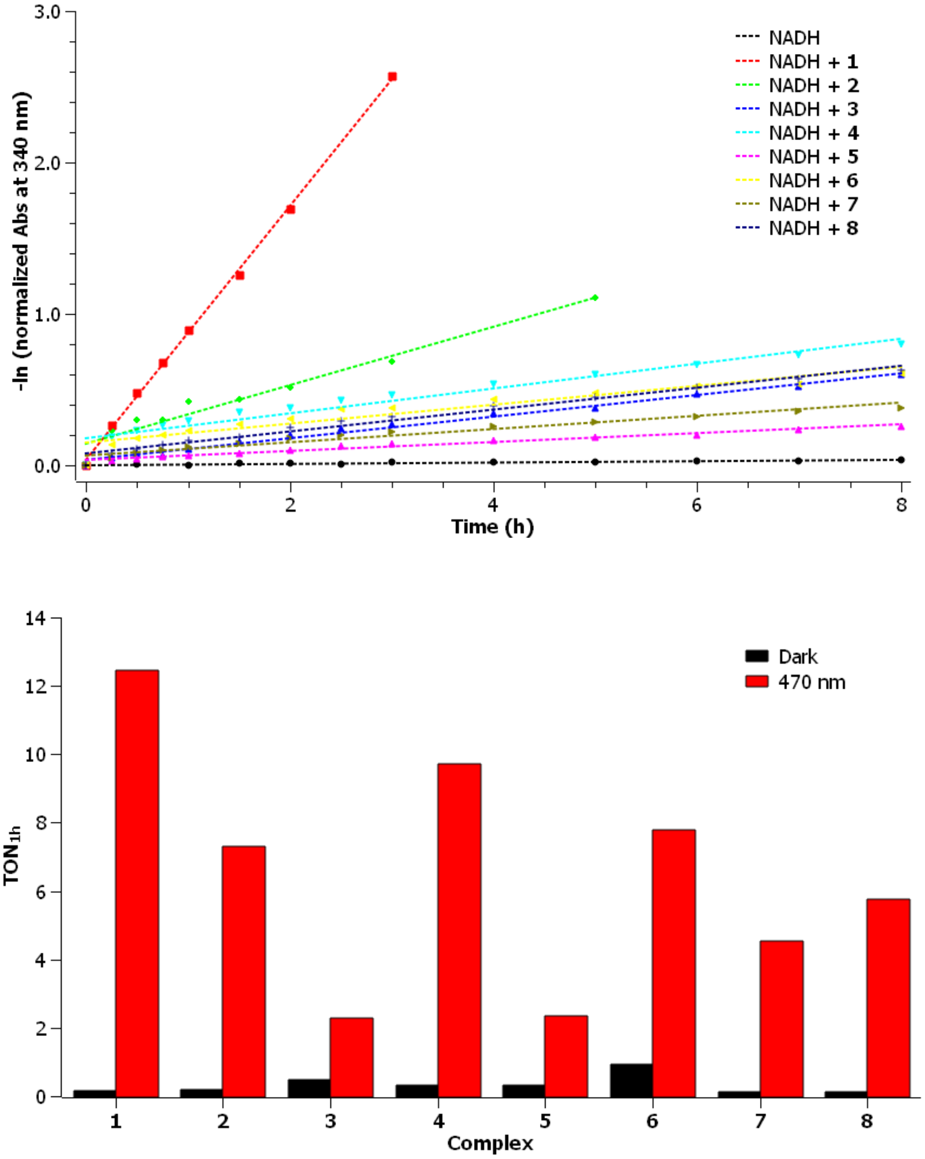

Figure S105. First-order kinetic plot of the photooxidation of NADH with complexes **1**-**8** (a) and TON values (1 hour) for complexes in the dark and under blue light irradiation (470 nm) (b).

Table S20. TON values of complexes **1**–**8** calculated in the first hour of the reaction.

	TON_{1h}	
	**Dark**	**470 nm**
**1**	0.16	12.47
**2**	0.18	7.32
**3**	0.47	2.28
**4**	0.31	9.71
**5**	0.37	2.36
**6**	0.94	7.79
**7**	0.12	4.54
**8**	0.14	5.75

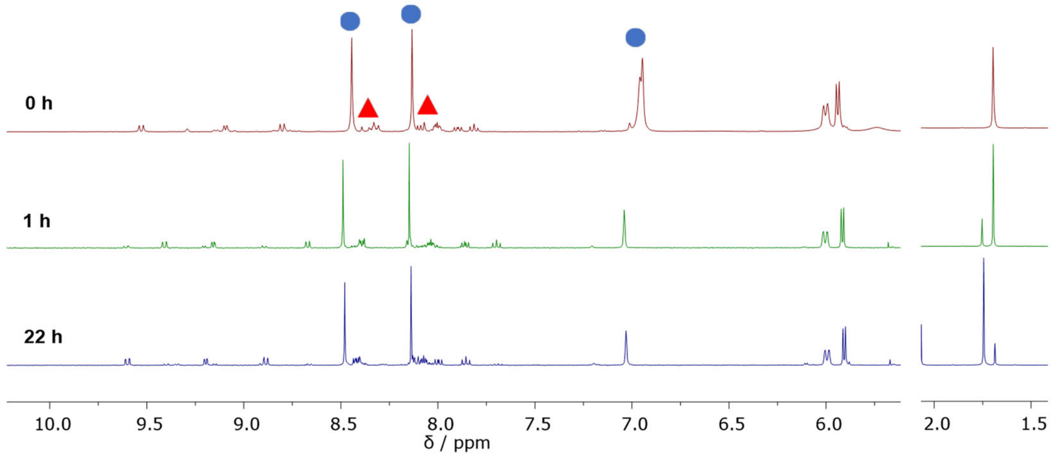

Figure S106. ^{1}H NMR spectra of oxidation of NADH by **1** in dark conditions in DMSO-d_6/D_2O (9/1, v/v). Blue circles (•) correspond to NADH signals and red triangles (▲) correspond to NAD^+ signals.

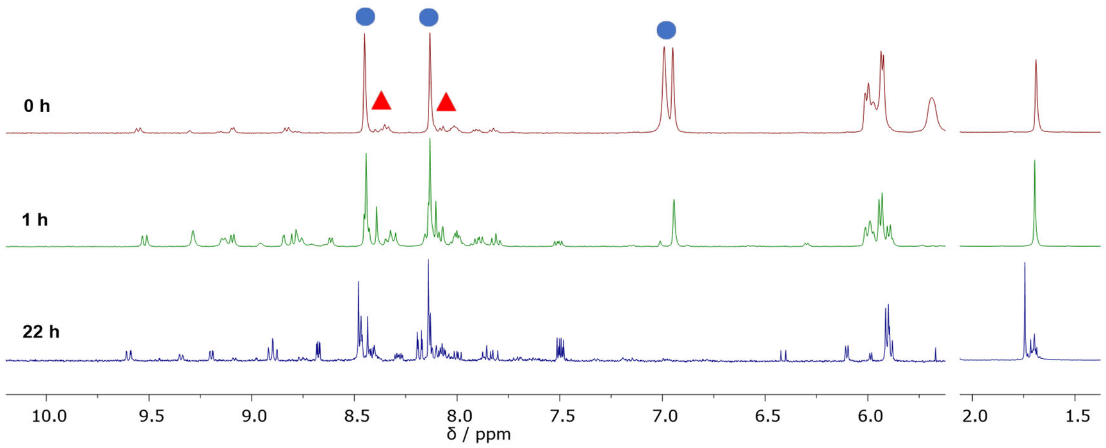

Figure S107. ^{1}H NMR spectra of oxidation of NADH by **1** under blue light irradiation (470 nm) in DMSO-d_6/D_2O (9/1, v/v). Blue circles (•) correspond to NADH signals and red triangles (▲) correspond to NAD^+ signals.

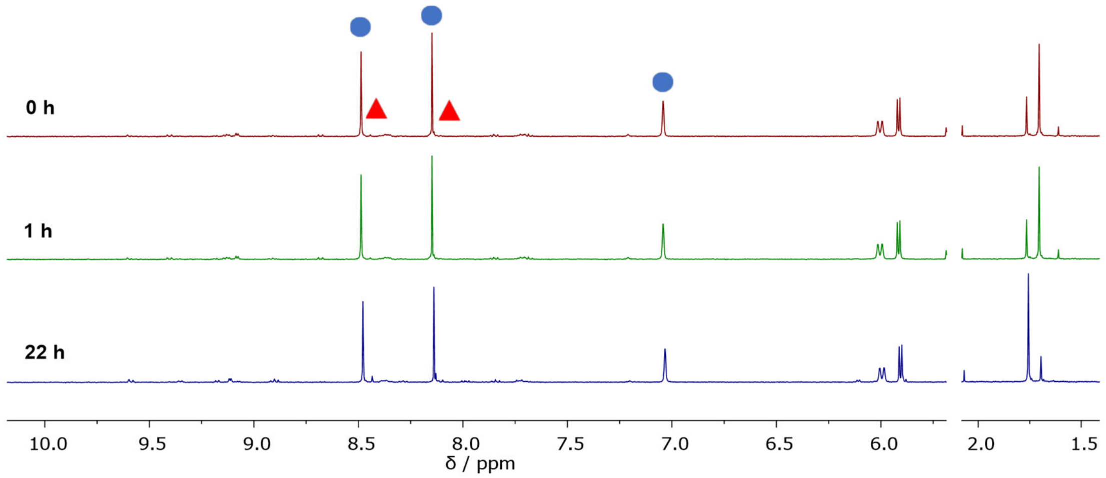

Figure S108. ^{1}H NMR spectra of oxidation of NADH by **2** in dark conditions in DMSO-d_6/D_2O (9/1, v/v). Blue circles (•) correspond to NADH signals and red triangles (▲) correspond to NAD^+ signals.

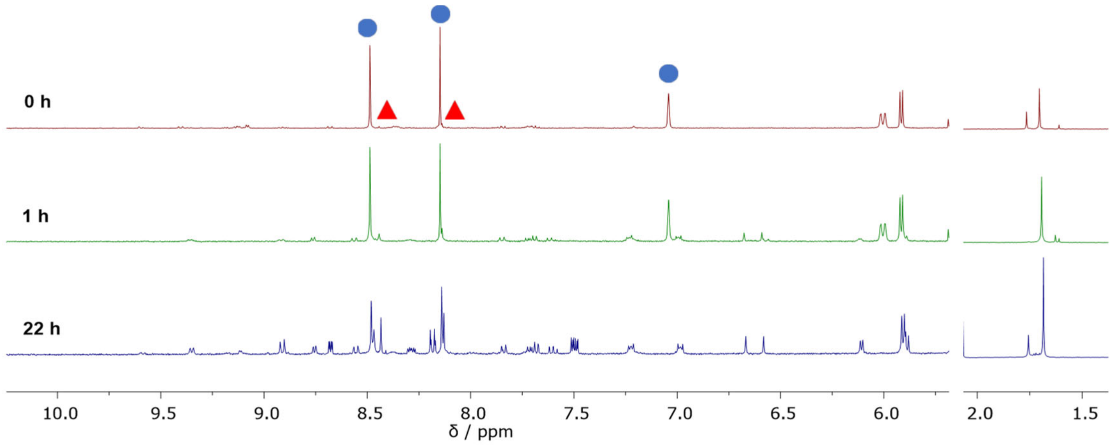

Figure S109. ^{1}H NMR spectra of oxidation of NADH by **2** under blue light irradiation (470 nm) in DMSO-d_6/D_2O (9/1, v/v). Blue circles (•) correspond to NADH signals and red triangles (▲) correspond to NAD^+ signals.

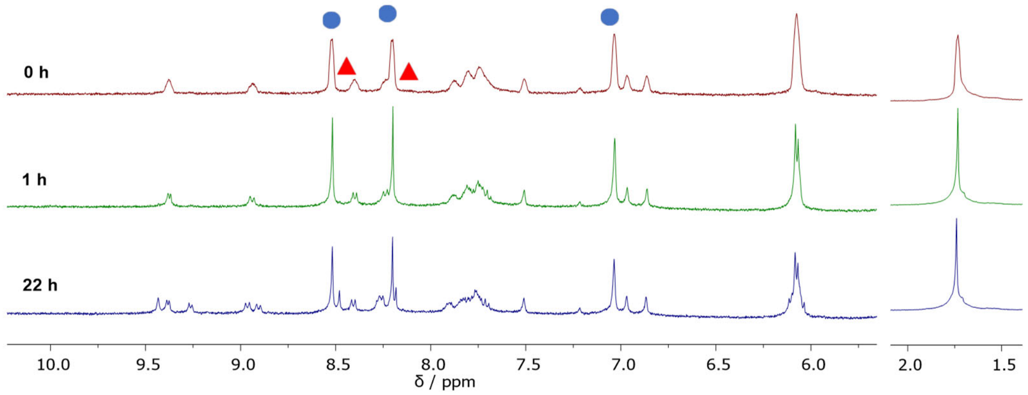

Figure S110. ^{1}H NMR spectra of oxidation of NADH by **5** in dark conditions in DMSO-d_6/D_2O (1/1, v/v). Blue circles (•) correspond to NADH signals and red triangles (▲) correspond to NAD^+ signals.

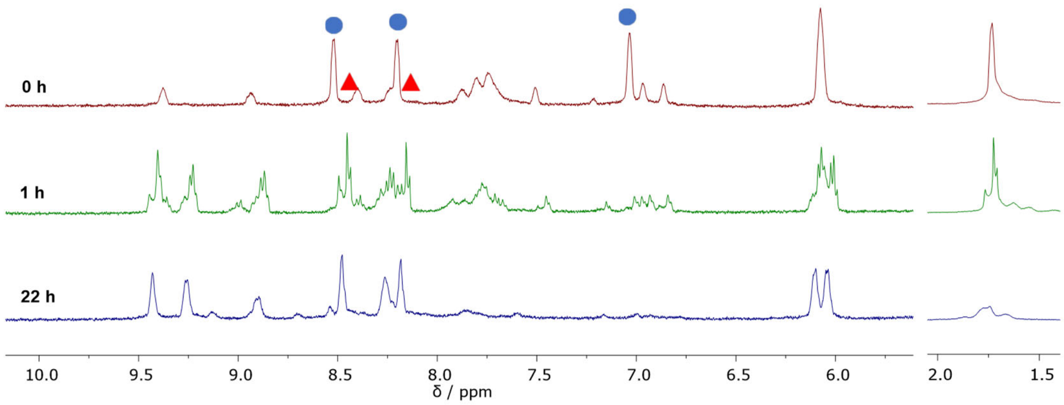

Figure S111. ^{1}H NMR spectra of oxidation of NADH by **5** under blue light irradiation (470 nm) in DMSO-d_6/D_2O (1/1, v/v). Blue circles (•) correspond to NADH signals and red triangles (▲) correspond to NAD^+ signals.

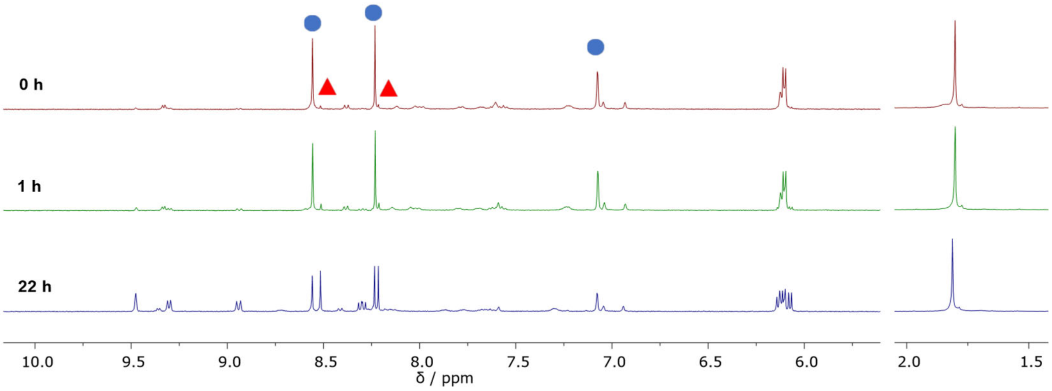

Figure S112. ^{1}H NMR spectra of oxidation of NADH by **6** in dark conditions in DMSO-d_6/D_2O (1/1, v/v). Blue circles (•) correspond to NADH signals and red triangles (▲) correspond to NAD^+ signals.

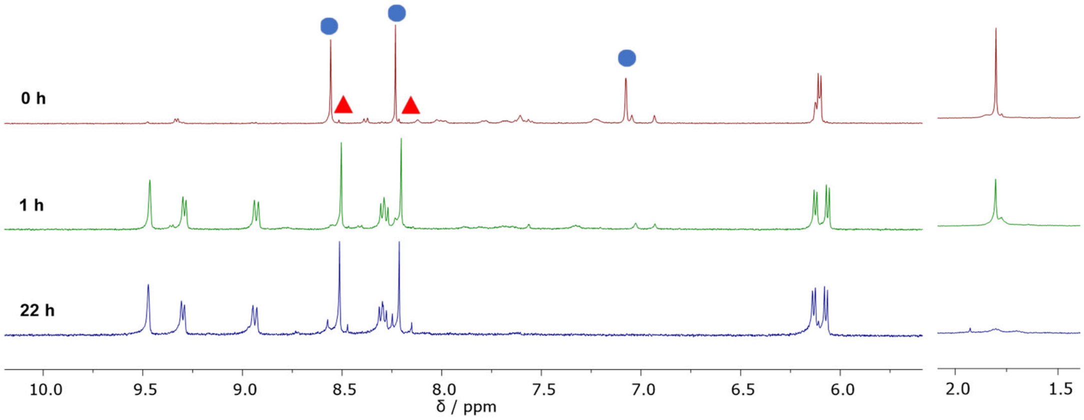

Figure S113. ^{1}H NMR spectra of oxidation of NADH by **6** under blue light irradiation (470 nm) in DMSO-d_6/D_2O (1/1, v/v). Blue circles (•) correspond to NADH signals and red triangles (▲) correspond to NAD^+ signals.

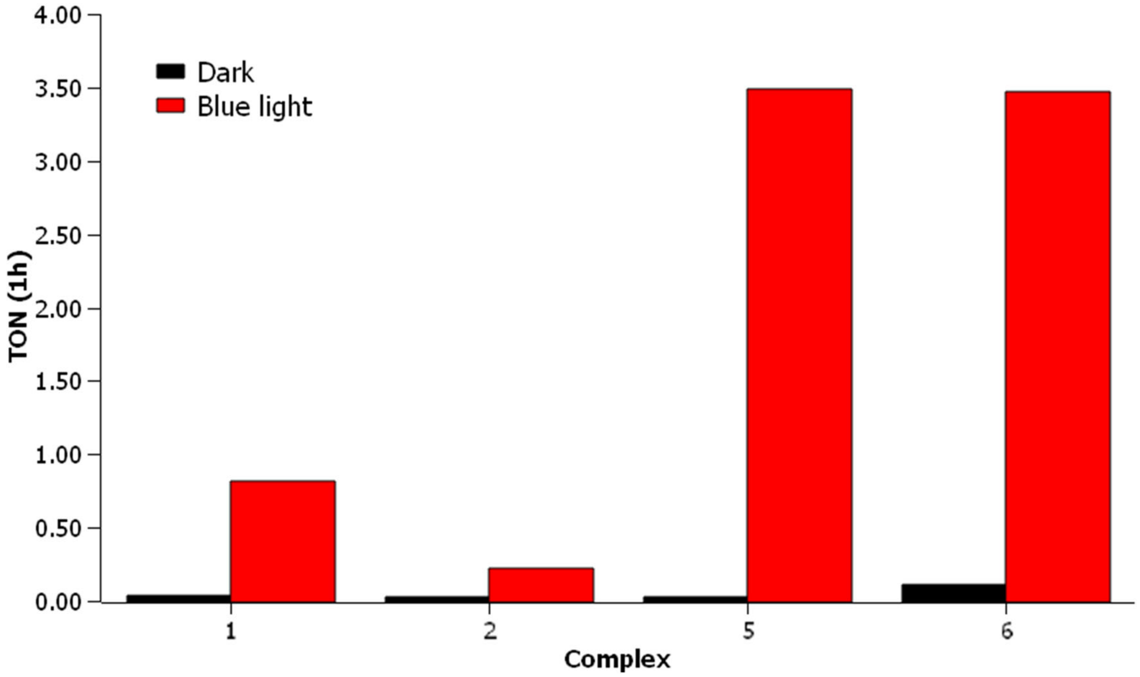

Figure S114. TON values for the oxidation of NADH (3.5 equiv.) with complexes **1**, **2**, **5** and **6** in dark conditions and under blue light irradiation (470 nm) after 1 hour in DMSO-d_6/D_2O (9/1, v/v) for complexes **1** and **2** or DMSO-d_6/D_2O (1/1, v/v) for complexes **5** and **6**.

DNA intercalation

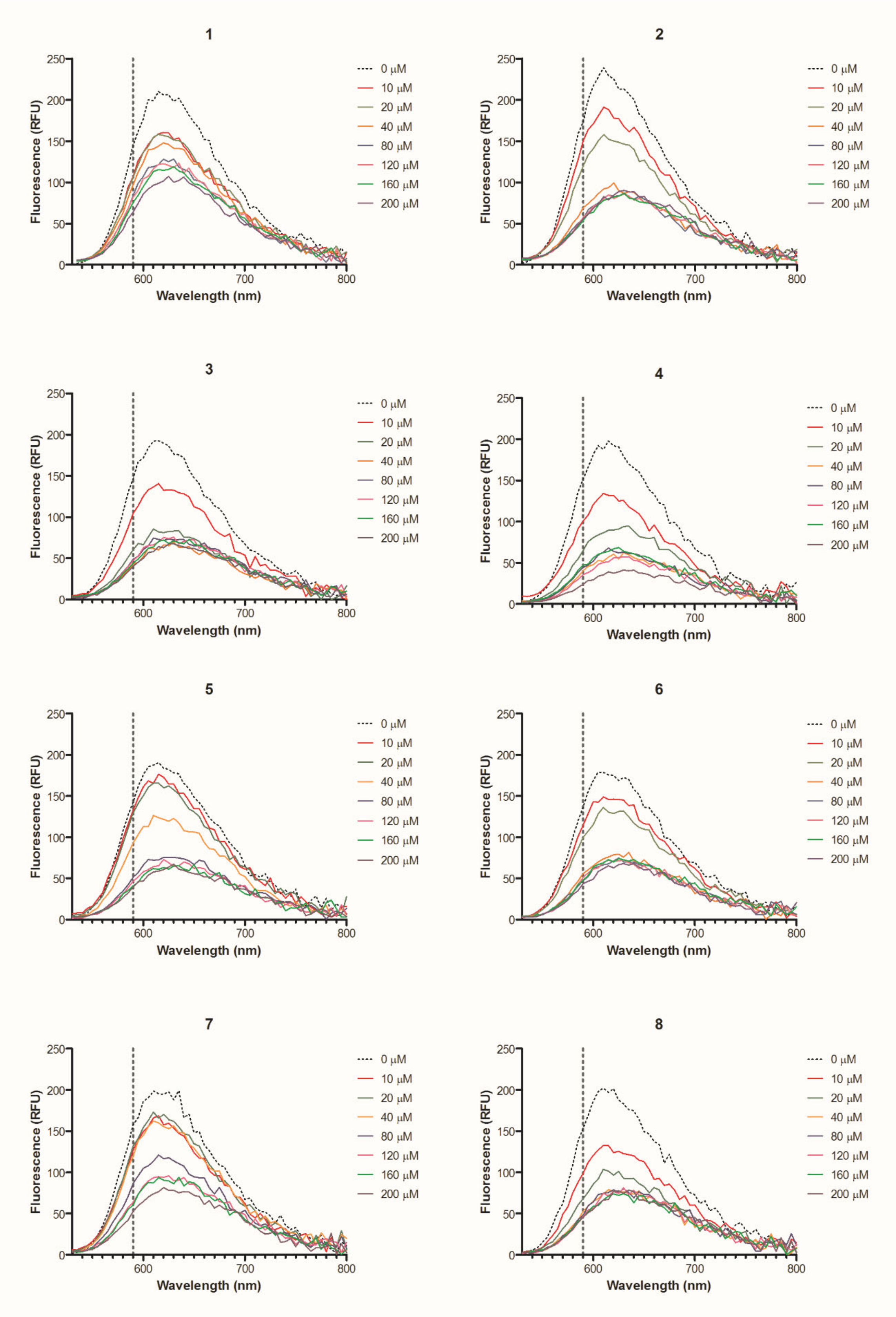

Figure S115. Fluorescence spectra of EtBr bound to CT-DNA at different concentrations of each compound. Spectra (λ_{ex}: 500 nm; λ_{em}: from 530 to 800 nm) were taken after 1 hour incubation. The fluorescence at 590 nm, which corresponds to the λ_{em} of EtBR-CT-DNA, is indicated with a with an λ_{ex} of 500 nm vertical dashed line) and was used to calculate the percentage of fluorescence compared to the untreated sample. These percentages were then used to calculate C_{50} values.

Lysosomal damage

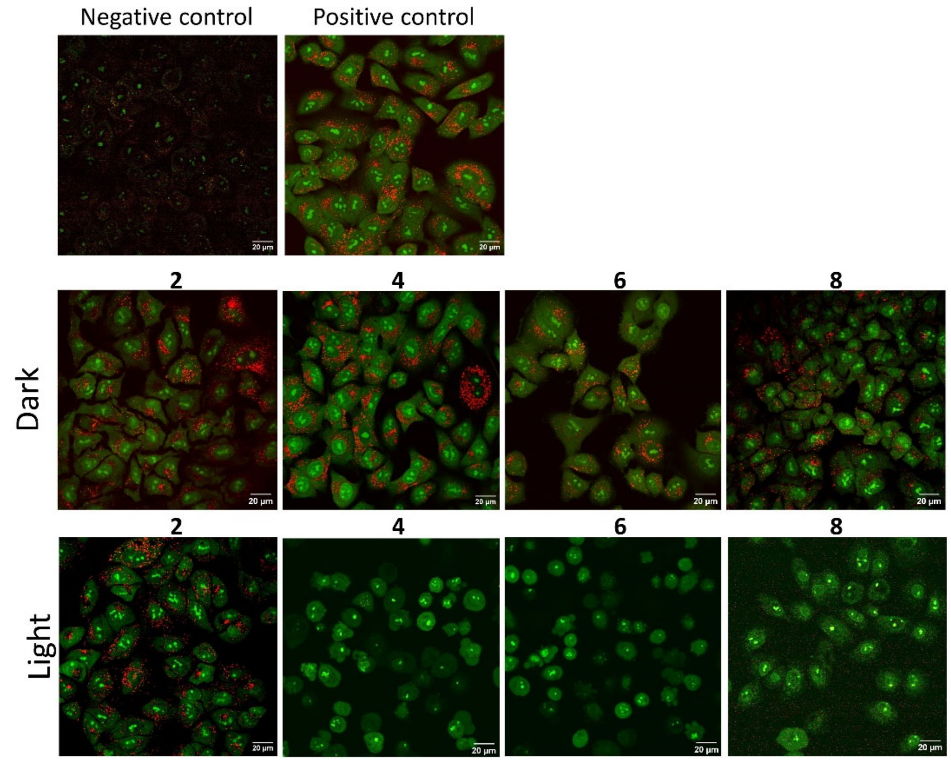

Figure S116. Interaction of the complexes with lysosomes. A549 cells were treated with the complexes **2**, **4**, **6** and **8** at 10 nM in the dark or with light irradiation (1 hour, 460 nm, 24.1 J cm^{-2}). Control cells were incubated with medium alone. Lysosomal damage was evaluated by confocal microscopy using AO staining (λ_{ex} = 488 nm). Cell cytoplasm and nucleoli were visualized in green (λ_{em} = 510 nm) while acidic cellular compartments, such as lysosomes, were visualized in red (λ_{em} = 625 nm).

Reactor for photochemical studies

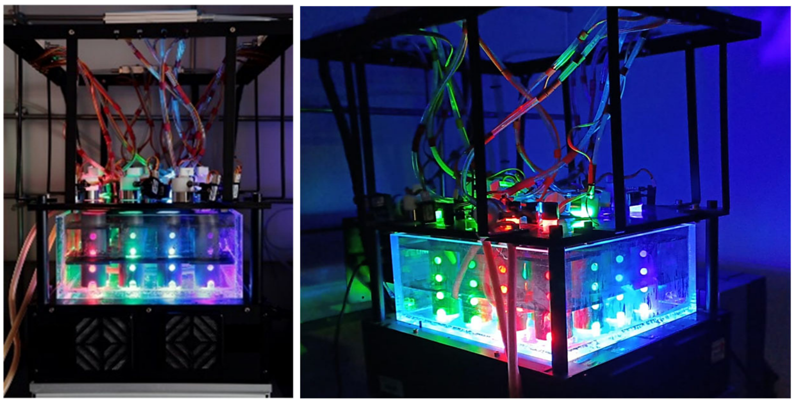

Figure S117. 'Medusa' photoreactor used for the photochemical experiments.
